# Dihedral–torsion model potentials that include angle-damping factors†

**DOI:** 10.1039/d4ra08960j

**Published:** 2025-03-07

**Authors:** Thomas A. Manz

**Affiliations:** a Chemical & Materials Engineering, New Mexico State University Las Cruces NM 88001 USA tmanz@nmsu.edu

## Abstract

This groundbreaking study derives and tests several new dihedral torsion model potentials for constructing classical forcefields for atomistic simulations of materials. (1) The new angle-damped dihedral torsion (ADDT) model potential is preferred when neither contained equilibrium bond angle is linear (*i.e.*, (*θ*^eq^_ABC_ and *θ*^eq^_BCD_) ≠ 180°), at least one of the contained equilibrium bond angles is ≥ 130° (*i.e.*, (*θ*^eq^_ABC_ or *θ*^eq^_BCD_) ≥ 130°), and the dihedral torsion potential contains some odd-function contributions (*i.e.*, *U*[*ϕ*] ≠ *U*[−*ϕ*]). (2) The new angle-damped cosine only (ADCO) model potential is preferred when neither contained equilibrium bond angle is linear (*i.e.*, (*θ*^eq^_ABC_ and *θ*^eq^_BCD_) ≠180°), at least one of the contained equilibrium bond angles is ≥ 130° (*i.e.*, (*θ*^eq^_ABC_ or *θ*^eq^_BCD_) ≥ 130°), and the dihedral torsion potential contains no odd-function contributions (*i.e.*, *U*[*ϕ*] = *U*[−*ϕ*]). (3) The new constant amplitude dihedral torsion (CADT) model potential is preferred when neither contained equilibrium bond angle is linear (*i.e.*, (*θ*^eq^_ABC_ and *θ*^eq^_BCD_) ≠ 180°), both contained equilibrium bond angles are <130° (*i.e.*, (*θ*^eq^_ABC_ and *θ*^eq^_BCD_) < 130°), and the dihedral torsion potential contains some odd-function contributions (*i.e.*, *U*[*ϕ*] ≠ *U*[−*ϕ*]). (4) The constant amplitude cosine only (CACO) model potential is preferred when neither contained equilibrium bond angle is linear (*i.e.*, (*θ*^eq^_ABC_ and *θ*^eq^_BCD_) ≠180°), both contained equilibrium bond angles are <130° (*i.e.*, (*θ*^eq^_ABC_ and *θ*^eq^_BCD_) <130°), and the dihedral torsion potential contains no odd-function contributions (*i.e.*, *U*[*ϕ*] = *U*[−*ϕ*]). (5) The new angle-damped linear dihedral (ADLD) model potential is preferred when at least one contained equilibrium bond angle is linear (*i.e.*, (*θ*^eq^_ABC_ or *θ*^eq^_BCD_) = 180°). Most importantly, this article derives combined angle-dihedral coordinate branch equivalency conditions and angle-damping factors that ensure the angle-damped torsion model potentials (*e.g.*, ADDT, ADCO, and ADLD) are mathematically consistent and continuously differentiable even as at least one contained bond angle approaches linearity (*i.e.*, as (*θ*_ABC_ or *θ*_BCD_) → 180°). This article introduces the torsion offset potential (TOP). I show the TOP gives rise in some materials to the unusual physical phenomenon of slip torsion. For various molecules, extensive quantitative comparisons to high-level quantum chemistry calculations (*e.g.*, CCSD) and experimental vibrational frequencies showed these new dihedral torsion model potentials perform superbly.

## Abstract 2

This article introduces the new angle-damped dihedral torsion (ADDT), angle-damped linear dihedral (ADLD), angle-damped cosine only (ADCO), and constant amplitude dihedral torsion (CADT) model potentials.

## Graphical abstract

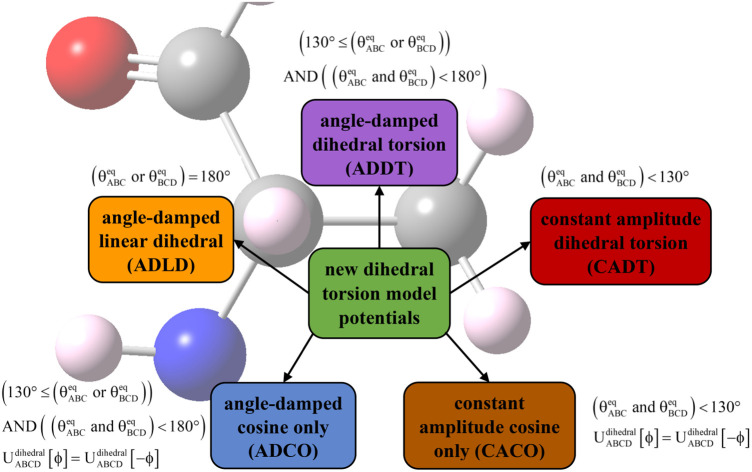

## 1. Introduction

The directed dihedral *ϕ*_ABCD_ measures the directed angle between the plane containing atoms ABC and the plane containing atoms BCD.^1^ The allowed range is:

1 −π < *ϕ*_ABCD_ ≤ π

The directed dihedral is defined for all situations except when atoms ABC or atoms BCD reside on a line, which corresponds to *θ*_ABC_ = π, *θ*_BCD_ = π, *θ*_ABC_ = 0, *θ*_BCD_ = 0, or at least two of the atoms have the same nuclear position. The latter condition of two or more atoms having the same nuclear position is physically prevented by the Pauli exclusion principle.^2^

In a material, a proper dihedral A–B–C–D corresponds to the situation in which bonds A–B, B–C, and C–D all exist.^3^ An improper dihedral corresponds to the situation in which three atoms are directly bonded to a common atom; for example, three hydrogen atoms directly bonded to a carbon atom in a methyl group.^4^ The remainder of this article refers to proper dihedrals. For simplicity, we subsequently refer to a ‘proper dihedral’ as a ‘dihedral’ and an ‘improper dihedral’ as an ‘improper-dihedral’.

Let 
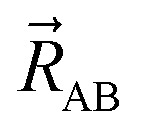
 be the vector from the position of atom A to the position of atom B:2
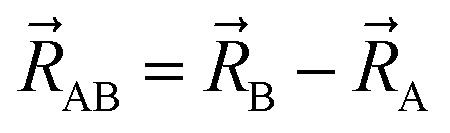


The directed dihedral *ϕ*_ABCD_ is computed as follows:^1,5^3
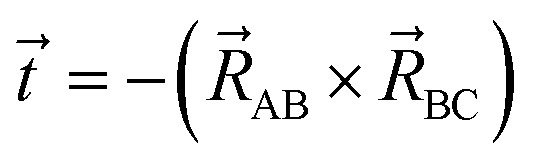
4
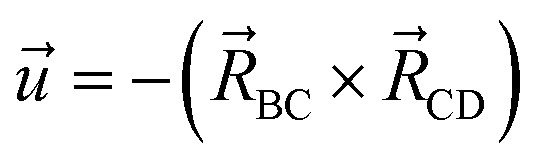
5
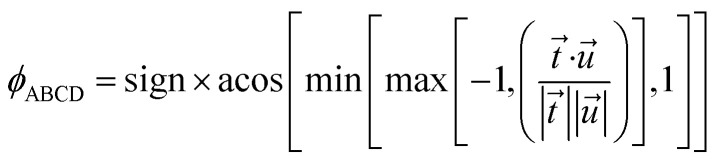


The sign is specified using the following method:6
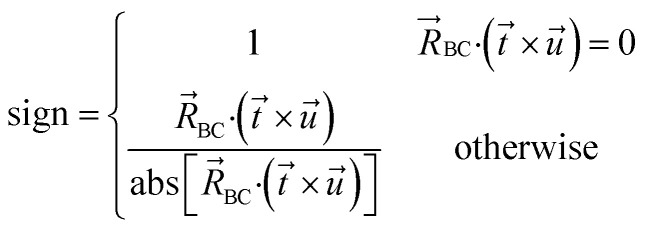
To cancel the effect of roundoff error, we used min and max functions to guarantee the argument of acos is between −1 and 1. When *ϕ*_ABCD_ is 0 or 180°, all four atoms are in the same plane. When *ϕ*_ABCD_ = 0, atoms A and D are located on the same side of the BC bond in the ABCD plane. When *ϕ*_ABCD_ = 180°, atoms A and D are located on opposite sides of the BC bond in the ABCD plane. When *ϕ*_ABCD_ is −90° or 90°, the ABC and BCD planes are perpendicular.

**Fig. 1:**
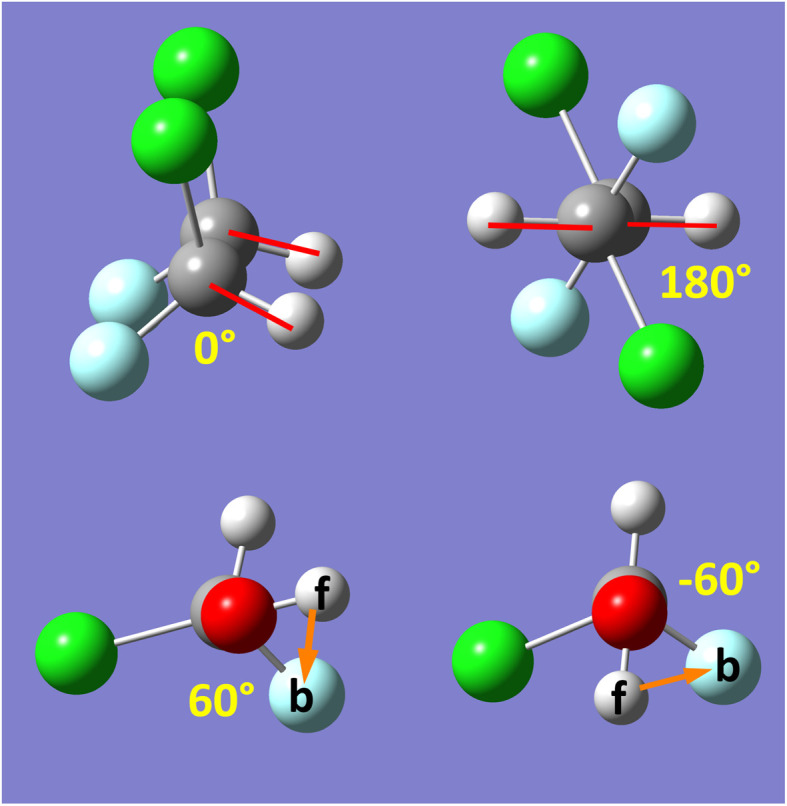
The achiral molecule (CClFH)_2_ (upper panels) and the *S* enantiomer of the chiral molecule C(OH)ClFH (lower panels) with different values of the HCCH or FCOH dihedral angle labeled, respectively. For (CClFH)_2_, the minimum energy geometry corresponds to a HCCH dihedral value of 180°. For C(OH)ClFH, the minimum energy geometry corresponds to a FCOH dihedral value of −64.7°. Atom colors: grey (C), green (Cl), cyan (F), white (H), red (O). The dihedral value is positive (*cf.* negative) if the back substituent is located clockwise (*cf.* counter-clockwise) relative to the front substituent. For clarity, the front substituent is labeled ‘f’, and the back substituent is labeled ‘b’ in the lower panels.

Fig. 1 shows an achiral isomer of (CClFH)_2_ and the *S* enantiomer of the chiral molecule C(OH)ClFH with different values of the HCCH or FCOH dihedral labeled, respectively. For these two molecules, Fig. 1 shows the particular isomers studied in this article.

The torsion potential for dihedral ABCD describes the change in potential energy that results from rotating bonds AB and CD around bond BC. Because this potential energy returns to its initial value when a complete full rotation is performed, the torsion potential is periodic in the dihedral value *ϕ*_ABCD_. A whole number of periods must be traversed when the dihedral changes from *ϕ*_ABCD_ = π to *ϕ*_ABCD_ → −π.

Please note that *U*[*ϕ*_ABCD_] = constant is not considered to be any kind of torsion potential. Rather, it is considered to be the absence of a torsion potential, because it has no dependence on the dihedral's value.

For convenience, we categorize torsion potentials into five classes such that each torsion potential is a member of exactly one of these five classes. (A) ‘Dihedral-only’ torsion potentials depend exclusively on the dihedral value (*e.g.*, *ϕ*_ABCD_) with no explicit dependence on the bond lengths or bond angles. (B) ‘Angle-damped’ torsion potentials depend exclusively on the dihedral value (*e.g.*, *ϕ*_ABCD_) and the two contained bond angle values (*i.e.*, *θ*_ABC_ and *θ*_BCD_) with no explicit dependence on the bond lengths. (C) ‘Distance-damped’ torsion potentials depend exclusively on the dihedral value (*e.g.*, *ϕ*_ABCD_) and the three contained bond lengths (*i.e.*, *R*_AB_, *R*_BC_, and *R*_CD_) with no explicit dependence on the bond angles. (D) ‘Fully-damped’ torsion potentials depend exclusively on the dihedral value (*e.g.*, *ϕ*_ABCD_), the two contained bond angle values (*i.e.*, *θ*_ABC_ and *θ*_BCD_), and the three contained bond lengths (*i.e.*, *R*_AB_, *R*_BC_, and *R*_CD_). (E) The final class contains all of the miscellaneous torsion potentials that do not fit into any of the first four classes.

Class A torsion potentials that depend only on the dihedral's value are abundant in the prior literature.^1,3,5–10^ However, it is straightforward to prove that every Class A torsion potential

7 *U*^dihedral_only^_torsion_[*ϕ*_ABCD_] = function[*ϕ*_ABCD_] ≠ constant

is mathematically and physically inconsistent if one of the contained bond angles approaches linearity.^11–13^ Proof: suppose that π minus the contained bond angle *θ*_ABC_ becomes an infinitesimal positive value

8 (π − *θ*_ABC_) = *ε* > 0

A dihedral scan can be performed by holding the atoms B, C, and D fixed and moving atom A in a circle whose radius is *d*_AB_sin[*ε*], where *d*_AB_ is the distance between atoms A and B. The circumference of this circle is 2π*d*_AB_sin[*ε*]. Since *U*^dihedral_only^_torsion_[*ϕ*_ABCD_] ≠ constant and *U*^dihedral_only^_torsion_[*ϕ*_ABCD_] satisfies the periodic boundary condition

9 *U*^dihedral_only^_torsion_[*ϕ*_ABCD_] = *U*^dihedral_only^_torsion_[*ϕ*_ABCD_ + 2π]

it follows that we can find some dihedral values *ϕ*_ABCD_ = *φ*_2_ and *ϕ*_ABCD_ = *φ*_1_ such that10
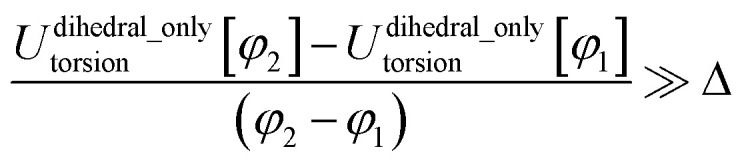
for any infinitesimal positive value *Δ*. For analogous reasons, we can also find some dihedral values *ϕ*_ABCD_ = *φ*_3_ and *ϕ*_ABCD_ = *φ*_4_ such that11
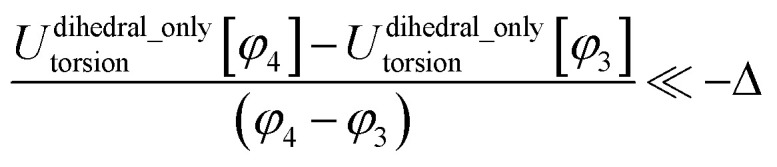
The only way we could not find such values is if *U*^dihedral_only^_torsion_[*ϕ*_ABCD_] = constant, which represents the absence of a torsion potential. The force exerted on atom A during each of these two parts of the dihedral scan is given by12
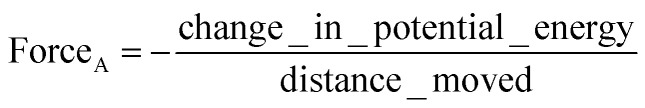
13
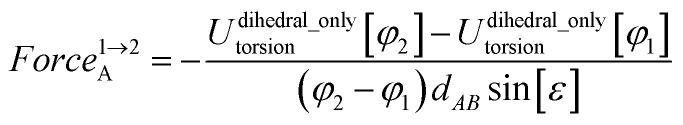
14
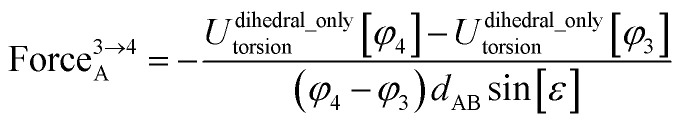
Substituting eqn (10) into (13), substituting eqn (11) into (14), and taking the limit *ε* → 0 gives15
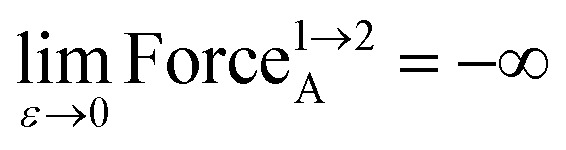
16
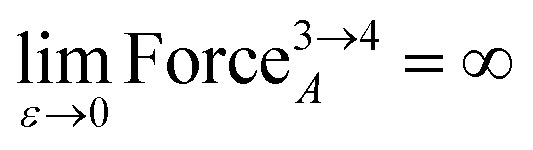
As *ε* → 0, the force on atom A fluctuates from infinitely positive to infinitely negative over an infinitesimally small distance. This extreme behavior is physically inconsistent. Practically, this means that every Class A torsion potential is inapplicable if one of the contained bond angles (*i.e.*, ∢ABC and/or ∢BCD) is statistically likely to approach linearity either (i) during the course of a molecular dynamics simulation or (ii) in thermally accessible conformations during a Monte Carlo simulation.^11,13,14^

Examples of previously published works on damped torsion potentials include the following. Grimme's quantum-mechanically-derived force field (QMDFF) includes a distance-damped torsion potential (*i.e.*, Class C torsion potential).^15^ There have been a few prior studies of angle-damped (*i.e.*, Class B) torsion potentials, but these are not comprehensive or fundamental in nature.^11,12,14,16,17^ Tuzun *et al.* describe a Class D torsion potential that includes both angle damping and distance damping.^13^

Herein, I introduce a comprehensive and fundamental theory of angle-damped dihedral torsion model potentials that are mathematically well-defined for all bond angle values. The remainder of this article is organized as follows. Section 2 introduces the combined angle-dihedral coordinate branch equivalency conditions, mathematical constraints on the angle-damping factors, a specific model function for the angle-damping factors, and principles used to derive the angle-damped torsion model potentials. Section 3 introduces the mirror image parameter *S*_instance_ that allows both mirror images to be classified within the same dihedral type and to be described by the same torsion force constant values. Section 4 derives the angle-damped dihedral torsion (ADDT) model potential as a Class B torsion potential that applies when neither equilibrium bond angle (*i.e.*, *θ*^eq^_ABC_ and *θ*^eq^_BCD_ for dihedral ABCD) is linear. Key components of the ADDT model potential include the torsion offset potential (TOP) and a set of orthonormal rotatable dihedral torsion modes. This ADDT model potential has continuous derivatives as either or both of the contained bond angles (*i.e.*, *θ*_ABC_ and *θ*_BCD_ for dihedral ABCD) approaches linearity. Section 5 introduces the constant-amplitude dihedral torsion (CADT) model potential that is a Class A torsion potential with orthonormal rotatable dihedral torsion modes. The CADT model potential emerges from the ADDT model potential when all of the angle-damping factors are set to a constant value. Section 6 introduces the angle-damped cosine only (ADCO) model potential as a Class B torsion potential that applies when neither equilibrium bond angle (*i.e.*, *θ*^eq^_ABC_ and *θ*^eq^_BCD_ for dihedral ABCD) is linear and the torsion potential contains no odd-function contributions (*i.e.*, *U*[*ϕ*] = *U*[−*ϕ*]). Section 6 also introduces the constant amplitude cosine only (CACO) model potential as a Class A torsion potential that applies when the torsion potential contains no odd-function contributions (*i.e.*, *U*[*ϕ*] = *U*[−*ϕ*]) and all of the angle-damping factors are set to a constant value. Section 7 describes rotatable dihedral mode smart selection using torsion scans. Section 8 discusses how the CADT and CACO model potentials compare to previously published Class A torsion potentials. Section 9 introduces the angle-damped linear dihedral (ADLD) model potential as a Class B torsion potential that applies when one or both of the equilibrium bond angles (*i.e.*, *θ*^eq^_ABC_ and *θ*^eq^_BCD_ for dihedral ABCD) is linear. Section 10 contains an extensive set of computed results for many molecules. These results show my torsion model potentials closely reproduce quantum-mechanically-computed potential energies surfaces for various torsion types. Section 10.8 compares vibrational frequencies computed from flexibility models (which include bond stretches, angle bends, and dihedral torsions) to prior experimentally-measured values for several small molecules. Section 11 concludes.

Note: this article adopts the convention that function arguments are enclosed in square brackets, while parentheses denote multiplication. For example, *y*[*x* + 2] means ‘*y* as a function of (*x* + 2)’ while *y*(*x* + 2) means ‘*y* times (*x* + 2)’.

Throughout this article the *R*-squared statistical descriptor has the definition17
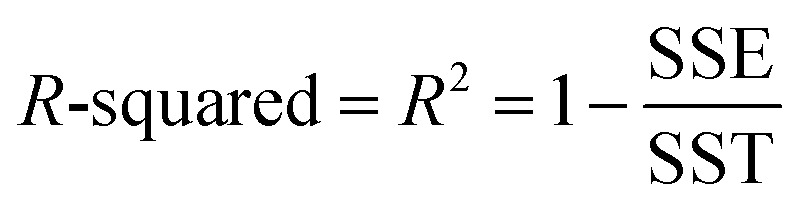
Specific definitions for the sum of squared errors (SSE) and sum of squares total (SST) are presented in each context.

## 2. Mathematical principles governing the angle-dihedral coupling

### 2.1 Combined angle-dihedral coordinate branch equivalency conditions

**Fig. 2:**
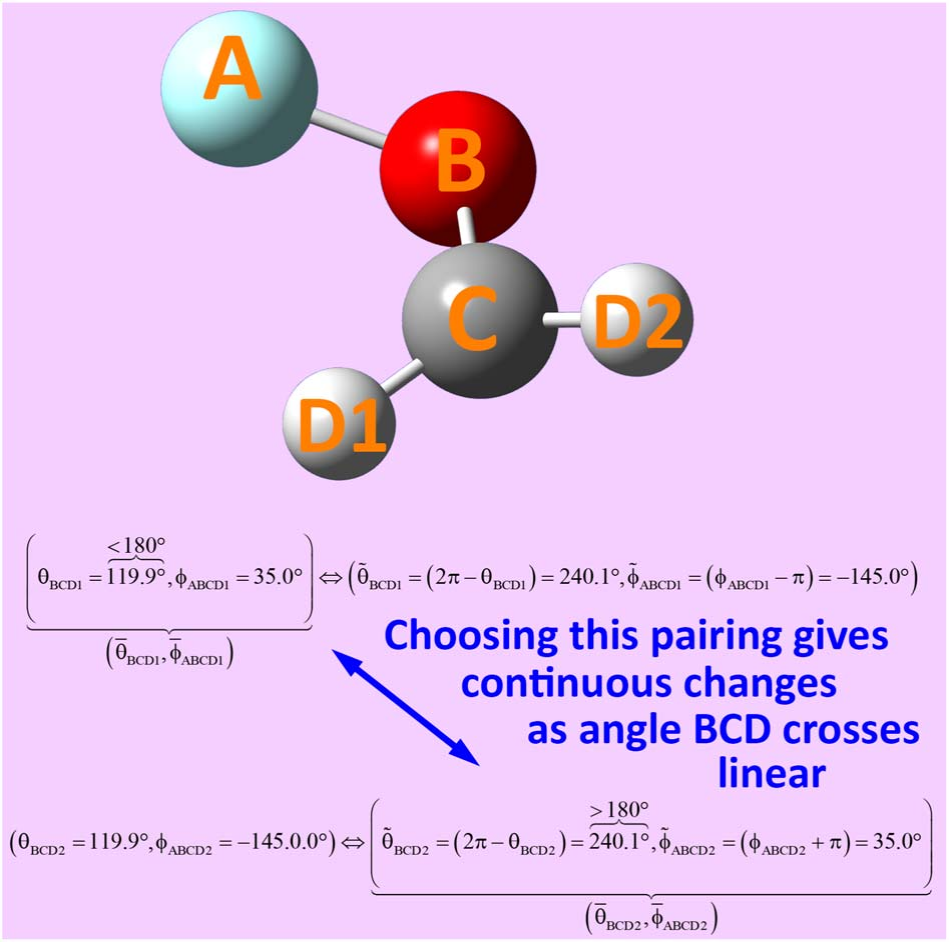
Illustration of angle-dihedral coordinate branch equivalency. Coordinate branches can be selected to yield continuous changes as the adjusted bond angle *θ̄*_BCD_ crosses linear (*i.e.*, crosses 180°).

The concept of combined angle-dihedral coordinate branch equivalency is illustrated in Fig. 2. As the BCD angle sweeps across the *θ*_BCD_ = π line (*e.g.*, from hydrogen atom position D1 to D2 in Fig. 2), the value of *ϕ*_ABCD_ discontinuously jumps from *φ* to (*φ* ± π). Accordingly, this means the coordinate pair (*θ*_BCD_,*ϕ*_ABCD_) = ((π − *Δ*),*φ*) (aka ‘coordinate branch 1’) and the hypothetical coordinate pair *(θ̃*_BCD_,*φ̃*_ABCD_) = ((π + *Δ*),(*φ* ± π)) (aka ‘coordinate branch 2’) describe the same ABCD conformation, where 0 < *Δ* < π. Consequently, the angle-damped dihedral torsion potential must satisfy the following coordinate branch equivalency relations:

18 *U*^angle-damped^_torsion_[*θ*_ABC_,*θ*_BCD_,*ϕ*_ABCD_] = *U*^angle-damped^_torsion_[*θ*_ABC_,(2π− *θ*_BCD_),(*ϕ*_ABCD_±π)] = *U*^angle-damped^_torsion_[(2π − *θ*_ABC_),*θ*_BCD_,(*ϕ*_ABCD_ ± π)]

As the BCD angle sweeps across the *θ*_BCD_ = π line (*e.g.*, from hydrogen atom position D1 to D2 in Fig. 2), this branch equivalency relationship allows us to choose values

19 (*θ̄*_BCD_,*φ̄*_ABCD_) ∈ {(*θ*_BCD_,*ϕ*_ABCD_),(*θ̃*_BCD_,*φ̃*_ABCD_)}

that change continuously. Analogously, we can find a set of coordinates

20 (*θ̄*_ABC_,*φ̄*_ABCD_) ∈ {(*θ*_ABC_,*ϕ*_ABCD_),*(θ̃*_ABC_,*φ̃*_ABCD_)}

that change continuously as the angle ABC angle sweeps across the *θ*_ABC_ = π line.

By constructing *U*^angle-damped^_torsion_ to be an infinitely differentiable function of the continuous coordinates (*θ̄*_ABC_,*θ̄*_BCD_,*φ̄*_ABCD_), this ensures that the derivatives21
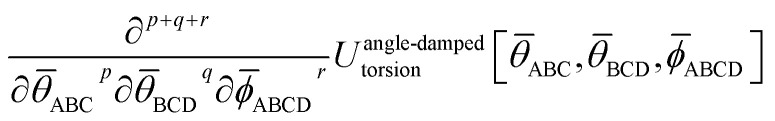
are continuous, where *p*, *q*, and *r* are any combination of non-negative integers. In summary, this provides a notion of continuous derivatives of all orders for *U*^angle-damped^_torsion_.

### 2.2 Mathematical constraints on the angle-damping factors

First, the angle-damping factor must go to zero as the corresponding bond angle approaches linearity. For simplicity, we seek a proxy variable that decreases monotonically from one when *θ*_ABC_ → 0 to zero when *θ*_ABC_ = π. The ‘kangal’22
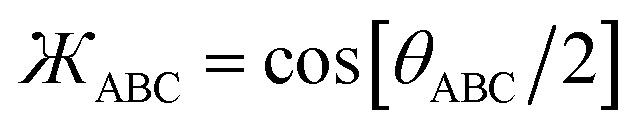
provides an ideal proxy variable. (Kangal is a specific breed of guardian dog. The term ‘kangal’ as used here is a play on words that means ‘keeper (or guardian) of the angle’.)

Consider a spherical coordinate system centered on atom C in Fig. 2, with radius *r* = *R*_CD_, polar angle *θ* = *θ*_BCD_, and azimuthal angle *ϕ* = *ϕ*_ABCD_. For a point within this spherical coordinate system, the corresponding Cartesian coordinates are

23 *x* = *r* sin[*θ*]cos[*ϕ*]

24 *y* = *r* sin[*θ*]sin[*ϕ*]

25 *z* = *r* cos[*θ*]

This satisfies the coordinate branch equivalency condition that (*r*,*θ*,*ϕ*) has the same (*x*, *y*, *z*) coordinates as (*r*,(2π − *θ*),*ϕ* ± π).

Using the standard trigonometric formulas

26 cos[*α* + *β*] = cos[*α*]cos[*β*] − sin[*α*]sin[*β*]

27 sin[*α* + *β*] = sin[*α*]cos[*β*] + cos[*α*]sin[*β*]

angle-damped higher-order torsion multiplicities can be constructed as

28 *x*^2^ − *y*^2^ = *r*^2^ sin^2^[*θ*]cos[2*ϕ*]

29 2*xy* = *r*^2^ sin^2^[*θ*]sin[2*ϕ*]

30 *x*^3^ − 3*xy*^2^ = *r*^3^ sin^3^[*θ*]cos[3*ϕ*]

31 3*x*^2^*y* − *y*^3^ = *r*^3^ sin^3^[*θ*]sin[3*ϕ*]

and so forth. For *n* = 1, 2, 3, 4, … the left-hand side is a *n*th-order polynomial in the *x* and *y* coordinates, while the right-hand side is *r*^*n*^ sin^*n*^[*θ*]cos[*nϕ*] or *r*^*n*^ sin^*n*^[*θ*]sin[*nϕ*].

Combining the trigonometric identities

32 sin[*θ*_BCD_] = 2 cos[*θ*_BCD_/2]sin[*θ*_BCD_/2]

with the definition of the kangal gives33
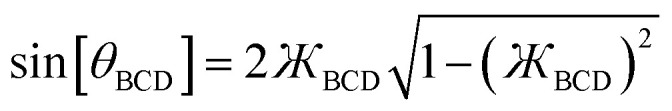
We can re-write eqn (28)–(31) in terms of the kangal as34
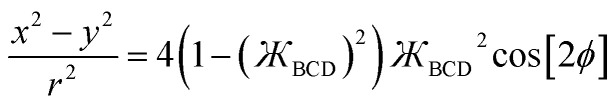
35
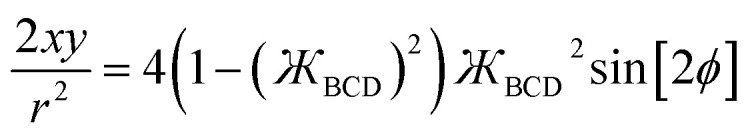
36
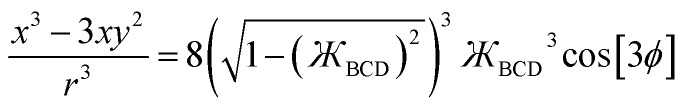
37
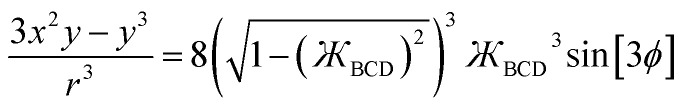


Next, we note that

38 cos[*nϕ*_ABCD_] = (−1)^*n*^cos[*n*(*ϕ*_ABCD_ ± π)]

39 sin[*nϕ*_ABCD_] = (−1)^*n*^sin[*n*(*ϕ*_ABCD_ ± π)]

40
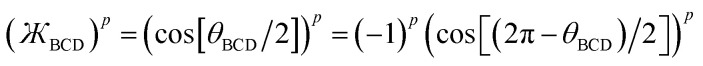
Therefore, to satisfy the coordinate branch equivalency condition that the torsion potential has the same numeric value for (*θ*_ABC_,*θ*_BCD_,*ϕ*_ABCD_) as for (*θ*_ABC_,(2π − *θ*_BCD_),*ϕ*_ABCD_ ± π), cos[2*jϕ*_ABCD_] and sin[2*jϕ*_ABCD_] must be multiplied by even-only powers of 
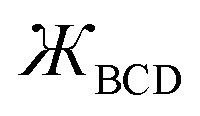
, where *j* is a whole number. Similarly, cos[(2*j* + 1)*ϕ*_ABCD_] and sin[(2*j* + 1)*ϕ*_ABCD_] must be multiplied by odd-only powers of 
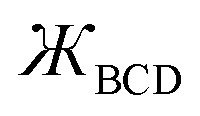
, where *j* is a whole number.

As the BCD bond angle approaches linearity (*i.e.*, 
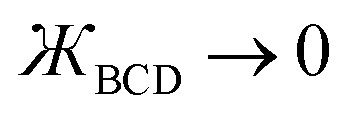
), the angle-damping product on the right-hand side of eqn (34)–(37) have derivatives 
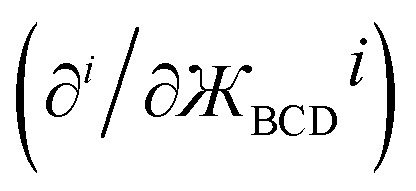
 equal to zero for *i* = 1, 2, 3 … (*n* − 1), where cos[*nϕ*] or sin[*nϕ*] is the dihedral-dependent factor. To preserve this behavior, the ideal angle-damped cos[*nϕ*] term should have the form:41
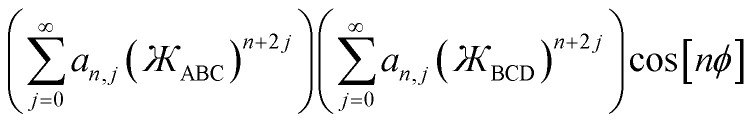
where *a*_*n*,0_ > 0. Note that the form of angle-damping factor for the ABC angle is analogous to that for the BCD angle. To preserve invariance in the choice of spherical coordinate system starting azimuthal angle, the angle-damping factor for sin[*nϕ*] is the same as the angle-damping factor for cos[*nϕ*].

### 2.3 A specific model for the elementary angle-damping functions

The zeroth-order angle-damping factor is a constant:

42 *f*^ABC^_0_ = 1

For *f*^ABC^_*n*≥1_, there are four regimes of bond angles:

• Linear bond angle: when *θ*_ABC_ = 180°, then 
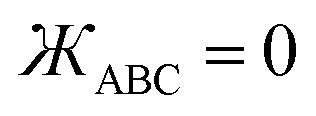
 and *f*^ABC^_*n*≥1_ = 0.

• Wide-angle regime: when 130° ≤ *θ*_ABC_ < 180°, it follows from eqn (41) that the leading-order term in *f*^ABC^_*n*≥1_ is proportional to 
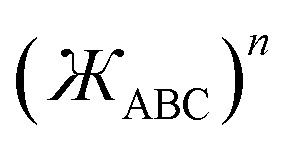
 and forms the dominant contribution.

• Acute angle regime*:* this regime corresponds to 0° < *θ*_ABC_ < 90° and 
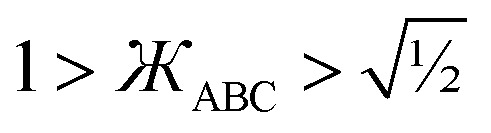
. Since we do not have any *a priori* information about how *f*^ABC^_*n*≥1_ should vary as a function of *θ*_ABC_ in this regime, a reasonable approximation is to mitigate this dependence by setting *f*^ABC^_*n*≥1_ ≈ 1 when 0 < *θ*_ABC_ < π/2. We also require that each *f*^ABC^_*n*≥1_ must be a monotonically increasing function of 
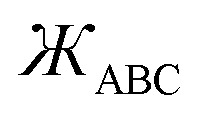
 and satisfy the limit43
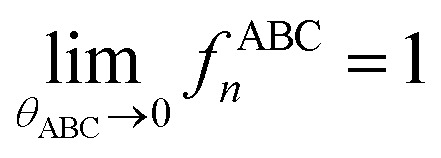


• Intermediate-angle regime: this regime corresponds to 90° ≤ *θ*_ABC_ < 130° and defines the transition regime from perpendicular bond angle (*i.e.*, 90°) to wide bond angle (*i.e.*, 130°). Near the beginning of this transition regime when the bond angle is perpendicular or only slightly obtuse, we expect the different torsion modes to damp similarly as a constrained bond angle changes. In this case, the normalized shape of the angle-constrained torsion scan curve does not change substantially with small changes in the constrained *θ*_ABC_ value; however, the torsion barrier can change with constrained bond-angle changes. This can be accomplished by defining *f*^ABC^_*n*_*via* asymptotic matching such that *f*^ABC^_*n*≥1_ ≈ *f*^ABC^_1_ when *θ*_ABC_ ≲ 90°.

The following unique and parsimonious model achieves these goals. We construct polynomial functions 
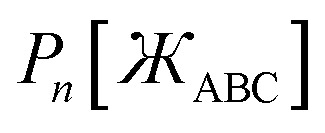
 for *n* = 1, 2, 3, and 4 that are asymptotically matched to each other. Each 
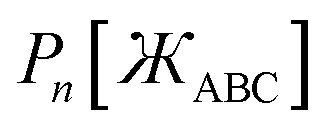
 will be a monotonically increasing function of 
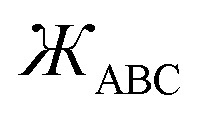
 over the range 
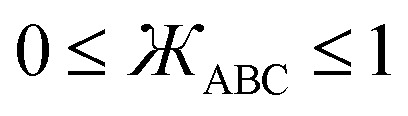
 and satisfy the boundary conditions44
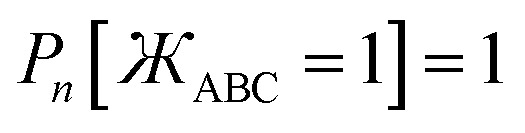

45 *P*_*n*≥1_[0] = 0

46 *P*_*n*≥1_[*ε*] ∝ *ε*^*n*^

where *ε* is positive infinitesimal.

First, we define two-term polynomials 
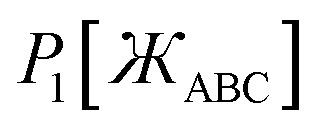
 and 
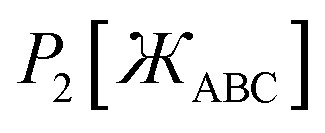
 that are asymptotically matched to each other in the 
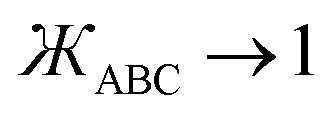
 limits. Define47
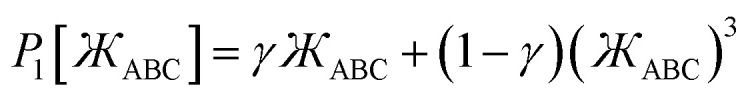
In eqn (47), the two coefficients (*i.e.*, *γ* and (1 − *γ*)) sum to one to ensure eqn (44) is satisfied. To ensure that 
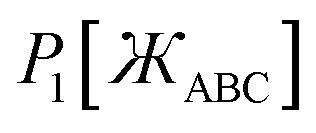
 is an odd function, there is no constant (power zero) and power two terms in eqn (47). Define48
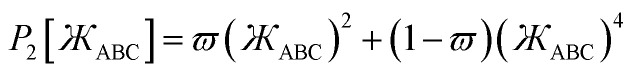
In eqn (48), the two coefficients (*i.e.*, *ϖ* and (1 − *ϖ*)) sum to one to ensure eqn (44) is satisfied. To ensures that *P*_2_[0] = 0, there is no constant (power zero) term in eqn (48). To ensure that 
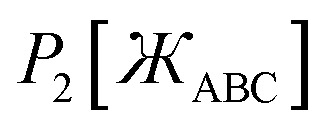
 is an even function, there is no power one or power three terms in eqn (48). Substituting49
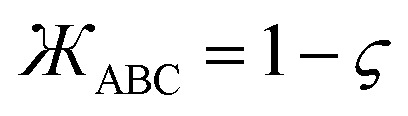
into eqn (47) and (48) gives

50 *P*_1_[1 − *ς*] = *γ*(1 − *ς*) + (1 − *γ*)(1 − *ς*)^3^ = 1 + *ς*(2*γ* − 3) + *ς*^2^(3 − 3*γ*) + …

51 *P*_2_[1 − *ς*] = *ϖ*(1 − *ς*)^2^ + (1 − *ϖ*)(1 − *ς*)^4^ = 1 + *ς*(2*ϖ* − 4) + *ς*^2^(6 − 5*ϖ*) +…

These two can be asymptotically matched by equating coefficients for their first- and second-order terms:

52 2*γ* − 3 = 2*ϖ* − 4

53 3 − 3*γ* = 6 − 5*ϖ*

Eqn (52) and (53) comprise a linear equation system having the unique solution

54 *γ* = (1 − *ϖ*) = ¼

Substituting eqn (54) into (50) gives

55 *P*_1_[1 − *ς*] = 1 − 2.5*ς* + 2.25*ς*^2^ +…

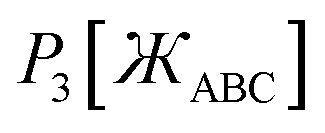
 and 
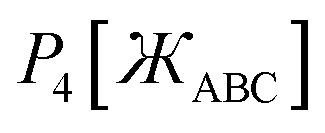
 can be asymptotically matched to 
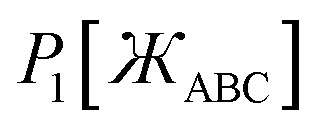
 if we choose56
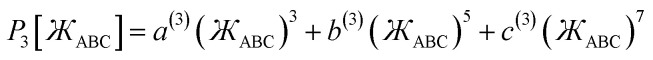
57
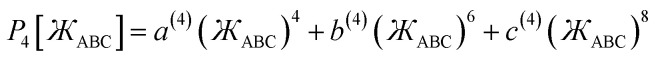
To ensure that 
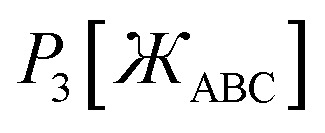
 is an odd function, eqn (56) includes only odd powers of 
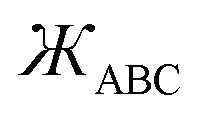
. To ensure that 
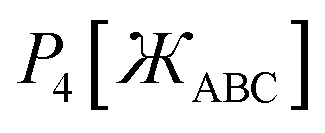
 is an even function, eqn (57) includes only even powers of 
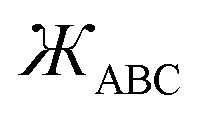
. Substituting eqn (49) into (56) and (57) gives

58 *P*_3_[1 − *ς*] = (*a*^(3)^ + *b*^(3)^ + *c*^(3)^) − (3*a*^(3)^ + 5*b*^(3)^ + 7*c*^(3)^)*ς* + (3*a*^(3)^ + 10*b*^(3)^ + 21*c*^(3)^)*ς*^2^ +…

59 *P*_4_[1 − *ς*] = (*a*^(4)^ + *b*^(4)^ + *c*^(4)^) + (4*a*^(4)^ + 6*b*^(4)^ + 8*c*^(4)^)*ς* + (6*a*^(4)^ + 15*b*^(4)^ + 28*c*^(4)^)*ς*^2^ +…

Asymptotically matching these expansions to eqn (55) gives the following eqn systems:60
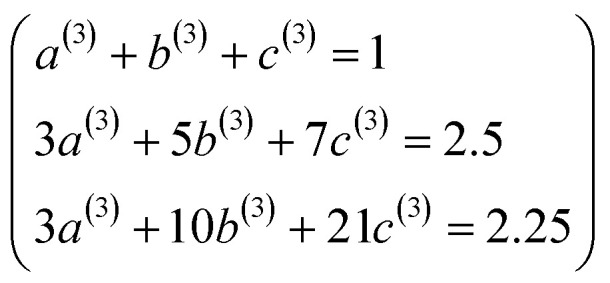
61
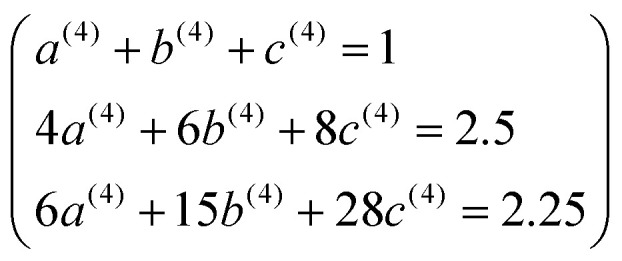
These have the unique solutions

62 (*a*^(3)^,*b*^(3)^,*c*^(3)^) = ((3/2),(−3/4),(1/4))

63 (*a*^(4)^,*b*^(4)^,*c*^(4)^) = ((5/2),(−9/4),(3/4))

In summary, these polynomials have the forms:64
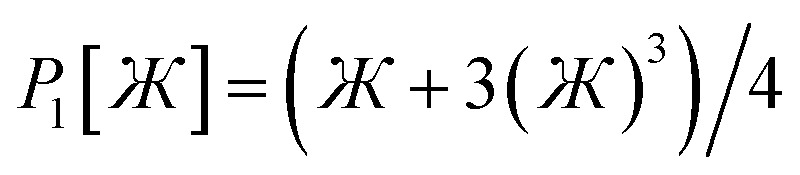
65
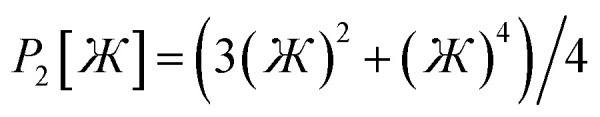
66
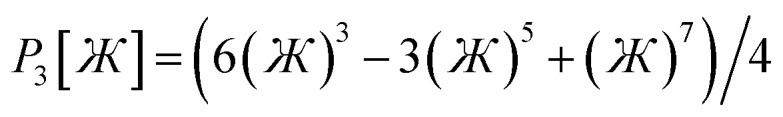
67
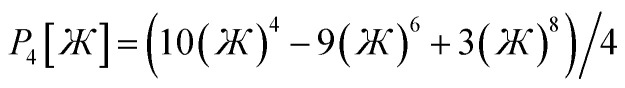


**Fig. 3:**
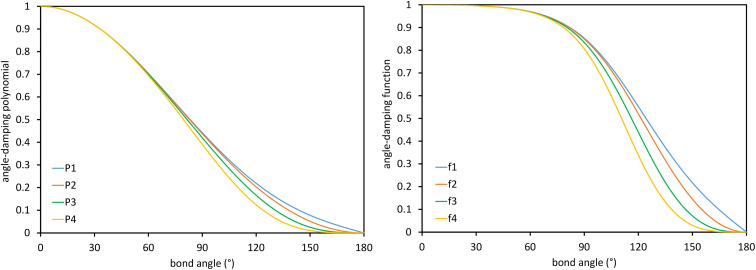
Values of the polynomials *P*_1_, *P*_2_, *P*_3_, and *P*_4_ (left panel) and elementary angle-damping functions *f*_1_, *f*_2_, *f*_3_, and *f*_4_ (right panel) plotted as a function of the bond angle. As the bond angle value gets smaller, all four curves approach the same curve; that is, they are asymptotically matched.

These are plotted in Fig. 3.

This asymptotic matching will ensure that *f*^ABC^_*n*≥1_ ≈ *f*^ABC^_1_ in the acute angle regime if we choose68
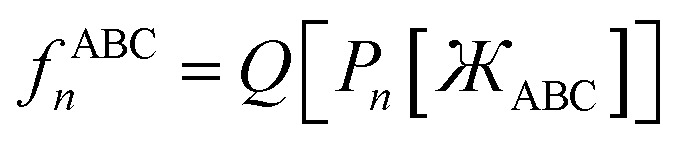
where the function *Q* is yet to be determined. To ensure that 
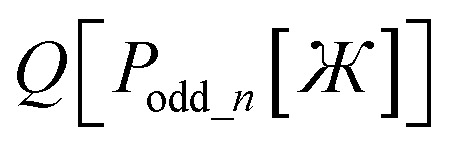
 is an odd function of 
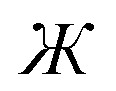
, it manifestly follows that *Q* must be an odd function. To achieve the boundary conditions69
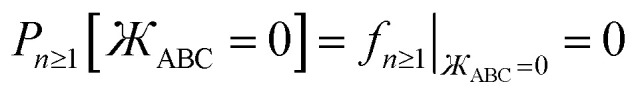
70
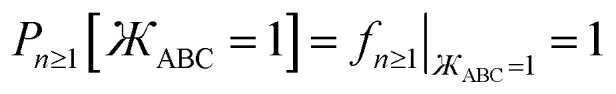
it manifestly follows that

71 *Q*[0] = 0

72 *Q*[1] = 1

Moreover, the function *Q* should monotonically increase from *Q*[0] = 0 to *Q*[1] = 1. Comparing eqn (41)–(46) shows that the leading-order term in the Maclaurin series expansion of *Q*[*x*] should be first-order in *x*. Arguably, the simplest function that meets all of these constraints is73
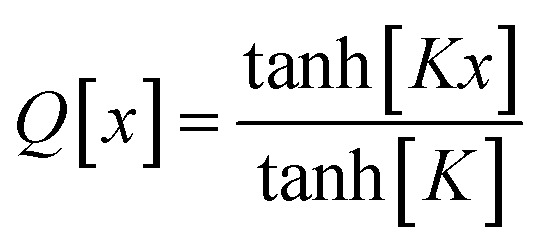


The value of *K* can be assigned by enforcing74
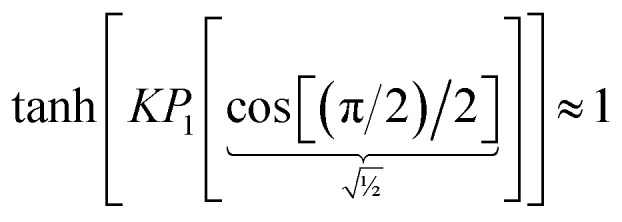
*via* asymptotic matching to reproduce the near-perpendicular-angle behavior described above. We asymptotically match the first three non-zero terms in the Maclaurin series expansion75
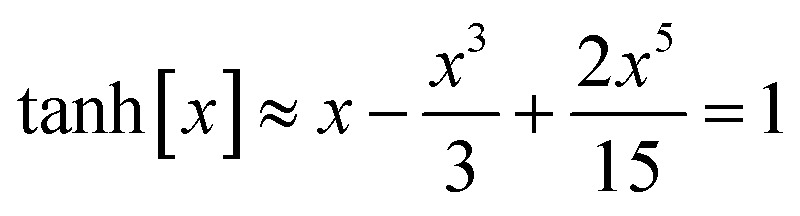
*x*_root_ = 1.244460035526845… is the only real-valued root of eqn (75). The other four roots are complex-valued. Evaluating76
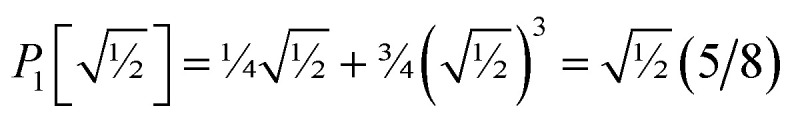
we obtain77
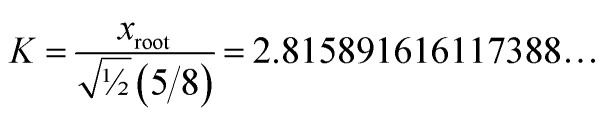


Why asymptotically match the first three non-zero terms (see eqn (75)) instead of one, two, four, or five non-zero terms? Computational tests showed that asymptotically matching an even number of non-zero terms (*i.e.*, 2 or 4 terms) led to the real-valued *x*_root_ < 0 which is not desirable. Therefore, an odd number of non-zero terms must be included in the asymptotic matching. Matching only one non-zero term (which would give *x*_root_ = 1) approximates the tanh[x] function as linear which does not account for any curvature effects of the tanh[*x*] function. Thus, it is desirable to include at least three non-zero terms in the asymptotic matching. Arguable, we should only include the leading-order curvature terms, because we do not want to overfit by including too many terms in the asymptotic matching. Note that the extreme case of overfitting resulting from including an infinitely large odd total number of terms in the asymptotic matching yields *K* → ∞, which is not a workable solution. Including precisely three non-zero terms in the asymptotic matching has the desirable effect of including some curvature in the tanh[*x*] expansion without any overfitting.

Putting this altogether, we finally have78
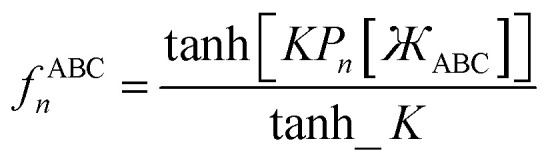
79
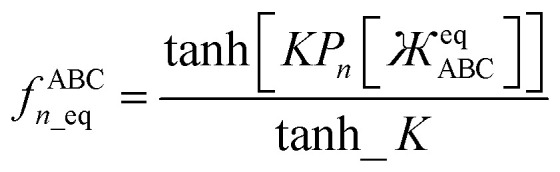
where

80 tanh_*K* = tanh[*K*] = 0.992861208914406…

with the value of *K* given in eqn (77). The expressions for *f*^BCD^_*n*_ and *f*^BCD^_*n*_eq_ are obtained by replacing 
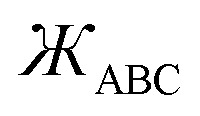
 and 
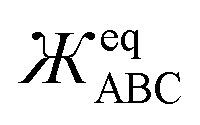
 with 
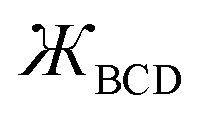
 and 
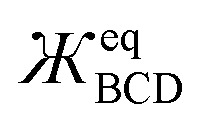
, respectively, in the above equations.

### 2.4 Principles used to derive the angle-damped torsion model potentials

The ADDT, ADCO, and ADLD model potentials describe dihedral torsion with angle damping. The ADDT model potential applies when neither included equilibrium bond angle is linear (*i.e.*, *θ*^eq^_ABC_ ≠ π and *θ*^eq^_BCD_ ≠ π). The ADCO model potential applies when the dihedral torsion potential is an even function of the dihedral value

81 *U*^ADCO^_ABCD_[*ϕ*] = *U*^ADCO^_ABCD_[−*ϕ*]

and neither included equilibrium bond angle is linear (*i.e.*, *θ*^eq^_ABC_ ≠ π and *θ*^eq^_BCD_ ≠ π). The ADLD model potential applies when one or both included equilibrium bond angles is linear (*i.e.*, *θ*^eq^_ABC_ = π or *θ*^eq^_BCD_ = π); in this case, *ϕ*^eq^_ABCD_ cannot be defined.

Before deriving the explicit forms of the ADDT, ADCO, and ADLD model potentials, we must first infer the underlying principles that should govern their forms. I propose the following principles:

(a) To be mathematically and physically self-consistent, every angle-damped dihedral torsion potential should satisfy the combined angle-dihedral coordinate branch equivalency conditions. (See Section 2.1 above.)

(b) The dependence on contained bond angles (*e.g.*, *θ*_ABC_ and *θ*_BCD_ for dihedral ABCD) should be formulated using algebraic combinations of elementary angle-damping functions, where each elementary angle-damping function decreases monotonically with increasing bond angle from a value of 1 (for bond angle = 0) to 0 (for bond angle = π). (See Sections 2.2 and 2.3 above.)

(c) For rigid values of the contained bond angles (*i.e.*, when the ABC and BCD bond angles are held constant), the dependence on dihedral value *ϕ*_ABCD_ should comprise a linear combination of torsion modes that is mathematically equivalent to a Fourier series expansion in *ϕ*_ABCD_.

(d) Due to the trigonometric identities in eqn (26) and (27), a Fourier series in *ϕ*_ABCD_ can be constructed equivalently using basis functions of the form {cos[*nϕ*_ABCD_ − *ψ*^ABCD^_*n*_]} or {cos[*nϕ*_ABCD_],sin[*nϕ*_ABCD_]}. For ADDT but not for ADLD, the Fourier series could also be equivalently constructed using basis functions of the form {cos[*n*(*ϕ*_ABCD_ − *ϕ*^eq^_ABCD_)],sin[*n*(*ϕ*_ABCD_ − *ϕ*^eq^_ABCD_)]}. Expanding82
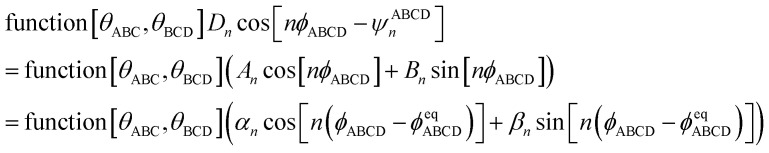
reveals that the combined angle-damping factor for the sine mode of multiplicity *n* must be proportional to the combined angle-damping factor for the cosine mode of multiplicity *n*. Thus, aside from a constant coefficient, sine and cosine modes sharing the same multiplicity *n* must have identical combined angle-damping factors.

(e) Since *ϕ*_ABCD_ is undefined when either *θ*_ABC_ = π or *θ*_BCD_ = π, the combined angle-damping factor multiplying any non-constant function of *ϕ*_ABCD_ must go to zero whenever either bond angle is linear (*i.e.*, whenever *θ*_ABC_ = π or *θ*_BCD_ = π). This effectively zeros out that term whenever either bond angle is linear.

(f) Since the square of a real-valued number is non-negative, and since the potential energy of a displaced geometry should not be lower than the ground-state geometry's potential energy, it follows that a useful potential energy model can be constructed using the ansatz83
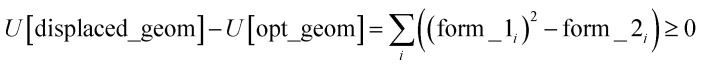
or similar ansatzes that use squared quantities to ensure the relative potential energy is non-negative. An individual term in the summation is non-negative when

84 form_2_*i*_ ≤ (form_1_*i*_)^2^

which is not a strict requirement, because only the combined sum (over *i* values) in eqn (83) must be non-negative. To ensure the right-hand side of eqn (83) is zero at the optimized geometry, each individual term is defined to be zero at the optimized geometry:

85 form_1_*i*_[opt_geom] = 0

86 form_2_*i*_[opt_geom] = 0

I call this extremely useful and powerful approach ‘completing the squares’.

(g) Within the ‘completing the squares’ approach, it is often preferable to construct the individual form_1_*i*_ and form_2_*i*_ terms using the ‘most modest and least biased’ approach. This means that among different options for constructing the model potential, we preferably choose the option that increases the potential energy by the smaller amount and with less correlation bias. (A specific example of this is discussed in Section 4.1 below.)

(h) For the ADDT and ADCO model potentials, it is convenient to express each combined angle-damping factor as a ratio that equals one when both contained bond angles equal their equilibrium values. Practically, this means each combined angle-damping factor in the ADDT and ADCO model potentials is expressed as some algebraic combination of the ratios (*f*^ABC^_*j*_/*f*^ABC^_*j*_eq_) and (*f*^BCD^_*j*_/*f*^BCD^_*j*_eq_). These ratios should not be used in the ADLD model potential, because *f*^ABC^_*j*≥1_eq_ = 0 and/or *f*^BCD^_*j*≥1_eq_ = 0 when at least one of the contained equilibrium bond angles is linear.

Without loss of generality, an angle-damped dihedral torsion potential can be expanded as a multivariate Fourier series of the form87
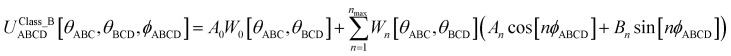
The form shown in eqn (87) spans the entire function space of continuously differentiable Class B torsion model potentials. Note that following point (d) above, the angle-damping function *W*_*n*_[*θ*_ABC_,*θ*_BCD_] appearing before the cos[*nϕ*_ABCD_] term must be the same as the one appearing before the sin[*nϕ*_ABCD_] term. The combined angle-dihedral coordinate branch equivalency condition applied to *W*_*n*_[*θ*_ABC_,*θ*_BCD_]cos[*nϕ*_ABCD_] and the analogous sine term requires that

88 *W*_*n*_[(2π − *θ*_ABC_),*θ*_BCD_] = *W*_*n*_[*θ*_ABC_,(2π − *θ*_BCD_)] = (−1)^*n*^*W*_*n*_[*θ*_ABC_,*θ*_BCD_]

## 3. The mirror image descriptor *S*_instance_

A reflection operation transforms a chemical geometry into its mirror image. For a molecule whose atom-in-material nuclear positions are expressed in Cartesian coordinates89
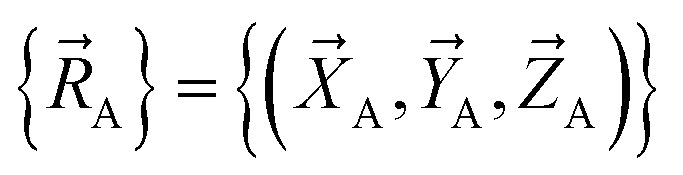
reflection can be achieved by changing the sign of the X-coordinate of each and every atom to give90
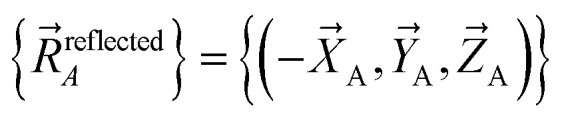
Eqn (90) corresponds to reflection about the *X* = 0 plane. Reflection can be performed about any plane in 3D Cartesian coordinate space.

Values of the dihedral ABCD before and after reflection are related by91
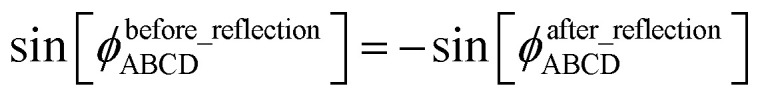
If the dihedral value is zero, then it is mapped onto itself upon refection. If the dihedral value is π (*i.e.*, 180°), then it is mapped onto itself upon reflection. Every other dihedral value is mapped upon reflection onto the dihedral value of the same absolute value but opposite sign. This follows directly from the definition of the directed dihedral given in the Introduction above. In the absence of externally applied fields, mirror image geometries have the same energy.

For this reason, it is convenient to define dihedral types such that all dihedral instances belonging to the same dihedral type: (1) are composed of the same atom types, bond types, and angle types, and (2) have the same absolute value of equilibrium dihedral value (within a tolerance).^18,19^ Dihedral instances belonging to the same dihedral type may have different dihedral value signs. (*i.e.*, they may be local mirror images of each other).^18,19^ Each dihedral type has its own set of torsion force constant values that apply to all dihedral instances in that dihedral type. According to this scheme, dihedral model potentials should be defined in such a way that mirror image dihedral instances belonging to the same dihedral type are accurately described by the same set of torsion force constant values.

This can be accomplished by defining the mirror image descriptor

92 −1 ≤ *S*_instance_ ≤ +1

in such a way that93
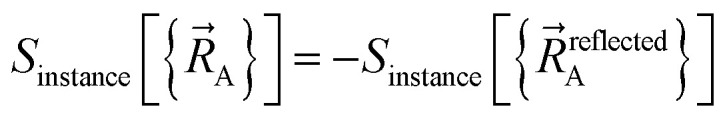


The general strategy is as follows. The torsion potential is decomposed into individual torsion modes such that each torsion mode potential has unchanged absolute value when the geometry is reflected. If the torsion mode would change sign upon reflection, it is multiplied by *S*_instance_ so that the product is invariant when the geometry is reflected. Each of these torsion modes has its own force constant value that is invariant upon reflection.

For example, suppose that *ϕ*_eq_ = −30° in the first instance and *ϕ*_eq_ = +30° in the second instance of two dihedrals of the same type. Then, a dihedral displacement to *ϕ* = 10° in the first instance is chemically equivalent to a dihedral displacement to *ϕ* = −10° in the second instance, and these should produce the same dihedral torsion potential values. Because cosine is an even function, for cosine modes we have

94 cos[*m*(*ϕ* − *ϕ*_eq_)] = cos[*m*(−*ϕ* − (−*ϕ*_eq_))]

which give equal contributions to the torsion potential. In this example, cos[*m*(10°−(−30°))] = cos[*m*(−10°–30°)] = cos[*m*40°]. Because sine is an odd function, we have to multiply it by the corresponding value of *S*_instance_ to recover equal contributions to the torsion potential upon reflection:

95 sin[*m*(*ϕ* − *ϕ*_eq_)] = −sin[*m*(−*ϕ* − (−*ϕ*_eq_))]

In this case, *S*_instance_ = −1 in the first instance and *S*_instance_ = +1 in the second instance to give sine function terms −sin[*m*(10° − (−30°))] = sin[*m*(−10° − 30°)] = −sin[*m*40°].

The case for which sin[*ϕ*_eq_] → 0 deserves some further clarification. In this case, both mirror images have the same equilibrium dihedral value, so the two distinct mirror images cannot be distinguished by the sign of the equilibrium dihedral's value. We are thus faced with several viable alternative choices. One possible solution is to turn off the sine modes by having *S*_instance_ → 0 when sin[*ϕ*_eq_] → 0; this has the effect of making the torsion potential symmetrical about *ϕ* = *ϕ*_eq_. In option # 1, this is done in a discrete way using the signum function:96
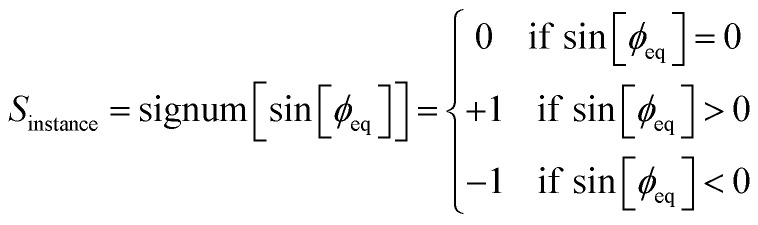
In option # 2, this is done in a smoothed continuous way using

97 *S*_instance_ = tanh[10^*D*^ sin[*ϕ*_eq_]]

where the smoothing exponent *D* controls the decimal place for which the smoothing occurs. For example, setting *D* = 2 means the second decimal place (*i.e.*, 0.01 radians) is where the smoothing occurs, while setting *D* = 1 means the first decimal place (*i.e.*, 0.1 radians) is where the smoothing occurs. Option # 2 has the key advantage that the value of *S*_instance_ is continuously differentiable with respect to continuous changes in *ϕ*_eq_. In option # 3, we retain the value of *S*_instance_ = +1 or −1 to distinguish the two mirror image structures even when sin[*ϕ*_eq_] → 0. This option is the most comprehensive, but it requires developing an alternate method to distinguish the two mirror images. For example, one could use pattern-matching algorithms to distinguish between the two mirror images.

For simplicity, this article used option #1 (see eqn (96)) to compute the value of *S*_instance_ used in the ADDT and CADT model potentials. However, these two model potentials are compatible with any of the three options described in the previous paragraph. For the ADLD model potential, option #3 must be used as explained in Section 9 below, because *ϕ*_eq_ is not defined for linear dihedrals. The ADCO and CACO model potentials do not use any *S*_instance_.

## 4. Angle-damped dihedral torsion (ADDT) model potential

### 4.1 Derivation including torsion offset potential

If neither contained equilibrium bond angle is linear, eqn (87) can be equivalently re-written (with the help of trigonometric identities in eqn (26) and (27)) as98
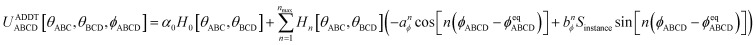
For convenience, we regroup the terms that equal zero when *ϕ*_ABCD_ = *ϕ*^eq^_ABCD_:99
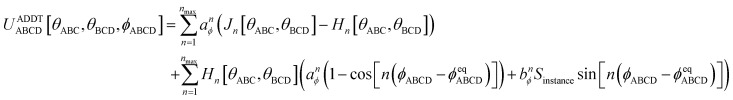


Comparing eqn (98) to (99) shows that100
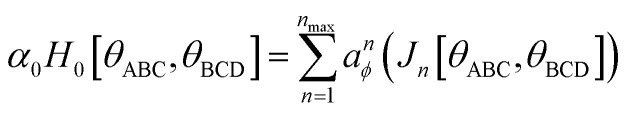


I call *J*_*n*_[*θ*_ABC_,*θ*_BCD_] − *H*_*n*_[*θ*_ABC_,*θ*_BCD_] the ‘torsion offset potential (TOP) for mode *n*’, because it adds a theta-dependent offset potential (aka ‘potential shift’) that is independent of the dihedral's value.

The combined angle-dihedral coordinate branch equivalency condition requires that

101 *H*_*n*_[(2π − *θ*_ABC_),*θ*_BCD_] = *H*_*n*_[*θ*_ABC_,(2π − *θ*_BCD_)] = (−1)^*n*^*H*_*n*_[*θ*_ABC_,*θ*_BCD_]

102 *J*_*n*_[(2π − *θ*_BCD_),*θ*_BCD_] = *J*_*n*_[*θ*_ABC_,(2π − *θ*_BCD_)] = *J*_*n*_[*θ*_ABC_,*θ*_BCD_]

To ensure the potential energy is non-negative for displaced geometries,

103 *J*_*n*_[*θ*_ABC_,*θ*_BCD_] ≥ 0

104 *J*_*n*_[*θ*^eq^_ABC_,*θ*^eq^_BCD_] = 1

will be constructed using a ‘completing the squares approach’. Following principle (*h*) of Section 2.4:

105 *H*_*n*_[*θ*^eq^_ABC_,*θ*^eq^_BCD_] = 1

Explicit formulas for *J*_*n*_ and *H*_*n*_ are derived as follows:

(1) To satisfy the combined angle-dihedral coordinate branch equivalency condition, we could construct a torsion offset potential (TOP) having the following form:

106 TOP_*n*_[*θ*_ABC_,*θ*_BCD_] = *J*_*n*_[*θ*_ABC_,*θ*_BCD_] − *H*_*n*_[*θ*_ABC_,*θ*_BCD_]

107
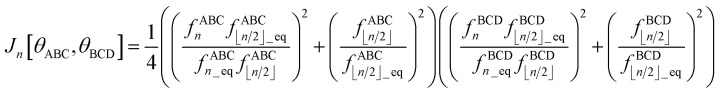
108
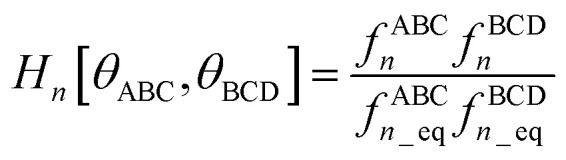
Here, ⌊*x*⌋ denotes floor[*x*], which is the largest integer less than or equal to *x*. Eqn (106)–(108) follows a ‘completing the squares’ strategy that guarantees that

109 TOP_*n*_[*θ*_ABC_,*θ*_BCD_] ≥ 0

*Proof*: Since110
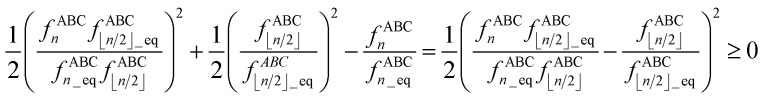
it immediately follows that111
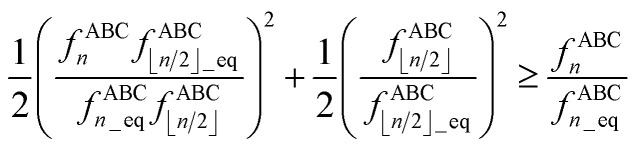
and similarly for the BCD angle. Substituting eqn (111) and the analogous eqn for BCD yields eqn (109).

(2) Consider the following generalization of eqn (110)112
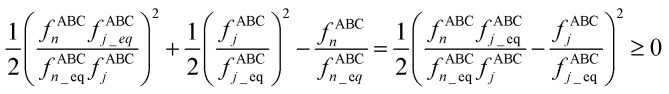
where 0 ≤ *j* ≤ *n*. In the limit 
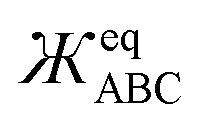
 approaches a small positive number (*i.e.*, 
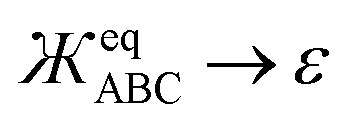
), then 1/*f*^ABC^_*j*_eq_ becomes proportional to *ε*^−*j*^, and *f*^ABC^_*j*_eq_/*f*^ABC^_*n*_eq_ becomes proportional to *ε*^*j*−*n*^. To keep the TOP from becoming excessively large, it is desirable therefore to formulate the TOP such that *max*[*j*,(*n* − *j*)] is as small as feasible. This follows principle (*g*) of Section 2.4 in order to construct the TOP in a way that produces the smallest energy increase. Immediately, this yields *j* = floor[*n*/2] or ceiling[*n*/2]. Due to the asymptotic matching between *f*^ABC^_*j*_eq_ and *f*^ABC^_*n*_eq_, there is a partial cancellation of amplitudes within the *f*^ABC^_*j*_eq_/*f*^ABC^_*n*_eq_. Therefore, to minimize the magnitude of the TOP, for odd *n* the smaller integer (*i.e.*, floor[n/2]) should be assigned to the 1/*f*^ABC^_*j*_eq_ while the larger integer (*i.e.*, ceiling[*n*/2]) should be assigned to the *f*^ABC^_*j*_eq_/*f*^ABC^_*n*_eq_ term. For even *n*, floor[*n*/2] and ceiling[*n*/2] are obviously equal. Thus we have *j* = floor[*n*/2] which yields eqn (107) and (110) above.

(3) The specific form of eqn (107) was chosen to avoid correlation bias. Whenever (*f*^ABC^_*n*_/*f*^ABC^_*n*_eq_) ≠ 1, the ABC bond angle has been displaced away from its equilibrium value; consequently, the TOP should be significantly positive regardless of whether (*f*^BCD^_*n*_/*f*^BCD^_*n*_eq_) is < 1, ∼1, or >1. Similarly, the TOP should be significantly positive whenever (*f*^BCD^_*n*_/*f*^BCD^_*n*_eq_) ≠ 1, regardless of whether (*f*^ABC^_*n*_/*f*^ABC^_*n*_eq_) is <1, ∼1, or >1. The form of eqn (107) satisfies this requirement.

(4) Most importantly, these choices for *J*_*n*_ and *H*_*n*_ guarantee that the minimum TOP_*n*_ value occurs for

113 TOP_*n*_[*θ*^eq^_ABC_,*θ*^eq^_BCD_] = 0

and that the minimum value for (1 − cos[*n*(*ϕ*_ABCD_ − *ϕ*^eq^_ABCD_)]) is 0 and occurs when *ϕ*_ABCD_ = *ϕ*^eq^_ABCD_. If a single torsion mode is active (*i.e.*, smart selected), then it is generally convenient to impose the following constraint to ensure the energy increases for all displacements away from the equilibrium ABC and BCD bond angles and for all displacements away from the equilibrium ABCD dihedral value:

114 *a*^*n*^_*ϕ*_ ≥ 0

What is the physical significance of the TOP? As an example, consider the HNCO molecule. For reasons explained in a companion article, as the NCO bond angle approaches linear (*i.e.*, π, 180°) the force exerted by the angle-bending potential must become zero.^20^ However, Fig. 9 shows the force exerted within the molecule along the angle-scan energy curve is markedly not zero as the NCO bond angle approaches linear (*i.e.*, π, 180°). This is not a paradox but rather a manifestation of the new energy term described by the TOP. Section 10.4 below shows the TOP gives rise to a new and unique physical phenomenon called ‘slip torsion’ that would not exist without it.

The following limits, which retain the leading term in the Taylor series expansion, should be used to avoid division by zero in all equations containing the ratios *f*^ABC^_*n*_/*f*^ABC^_⌊*n*/2⌋_ and/or *f*^BCD^_*n*_/*f*^BCD^_⌊*n*/2⌋_ (*e.g.*, eqn (107) and others) as one or both bond angles approach linearity:115
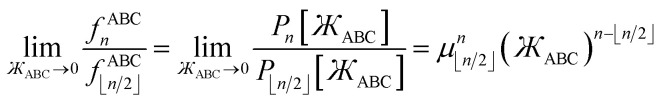
116
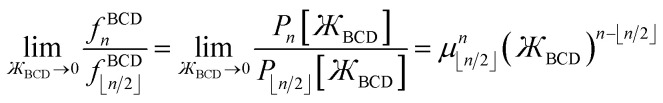

117 *μ*^2^_1_ = 3, *μ*^3^_1_ = 6, *μ*^4^_2_ = 10/3

I recommend using the leading order expansion shown in eqn (115) iff 
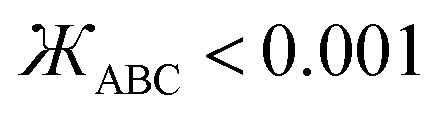
. I recommend using the leading order expansion shown in eqn (116) iff 
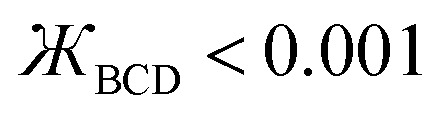
. For example, if 
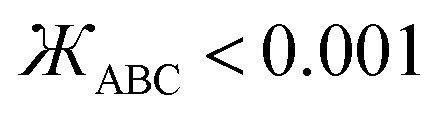
 then *f*^ABC^_4_/*f*^ABC^_*2*_ to leading order equals 
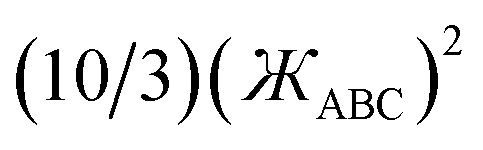
.

### 4.2 Derivation of the first seven independent torsion modes for rotatable dihedrals

To ensure the quantum-mechanically-computed ground-state geometry is an equilibrium geometry of the fitted classical forcefield, the following constraint should be imposed:118
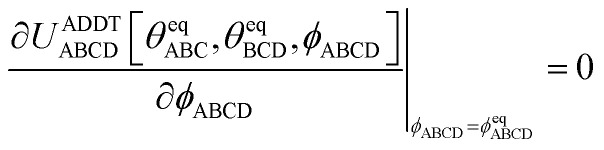


Substituting eqn (99), (104), and (105) into (118) yields the following constraint119
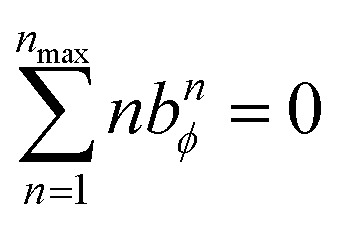


The constraint shown in eqn (119) has the effect of reducing the number of independent sine modes from *n*_max_ to (*n*_max_ − 1), and this reduces the total number of independent rotatable dihedral modes (cosine plus sine) from 2*n*_max_ to (2*n*_max_ − 1). One possible strategy to handle this reduced number of degrees of freedom is to use a linear regression algorithm that can incorporate any form of linear constraints (such as eqn (119)) when optimizing the force constant values. However, the glmnet implementation of the LASSO method handles bounds on the model parameters rather than general linear constraints.^21–23^ Since we use the LASSO method to address the multicollinearity and feature selection problems when optimizing the force constants,^18^ the sine torsion modes should be written in a way that makes them independent of each other without the need for a separate linear constraint.

This can be accomplished as follows. We choose *n*_max_ = 4, because it gives an excellent compromise between accuracy and computational costs. In this case, there are *n*_max_ = 4 independent cosine modes, which we label as modes 1 to 4. There are (*n*_max_ − 1) = 3 independent sine modes, which we label as modes 5, 6, and 7. For the first *n*_max_ = 4 modes, we can use the cosine torsion modes shown in eqn (123), because these already satisfy the zero torsion force condition at the equilibrium geometry. The remaining (*n*_max_ − 1) = 3 independent torsion modes must be constructed as orthogonal linear combinations of the sine torsion modes, where each independent torsion mode has a single independent *k*-value (aka ‘force constant value’).

First, we define

120 *U*^sin^_*n*_ = sin[*n*(*ϕ* − *ϕ*_eq_)]

The linear combinations 3*U*^sin^_1_ − *U*^sin^_3_ and 2*U*^sin^_2_ − *U*^sin^_4_ yield zero torsion force at the equilibrium geometry. Moreover, these two linear combinations are orthogonal to each other; that is, they have zero overlap integral between them. The overlap integral between two real-valued functions *g*_*i*_[*ϕ*_ABCD_] and *g*_*j*_[*ϕ*_ABCD_] is defined as121
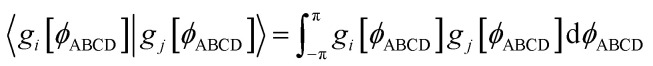


The seventh mode takes a bit more ingenuity to construct. Within the subspace of functions spanned by linear combinations of *U*^sin^_1_ and *U*^sin^_3_, the linear combination *U*^sin^_1_ + 3*U*^sin^_3_ is orthogonal to 3*U*^sin^_1_ − *U*^sin^_3_. Within the subspace of functions spanned by linear combinations of *U*^sin^_2_ and *U*^sin^_4_, the linear combination *U*^sin^_2_ + 2*U*^sin^_4_ is orthogonal to 2*U*^sin^_2_ − *U*^sin^_4_. These can be combined to form the linear combination *U*^sin^_1_ + 3*U*^sin^_3_ − *U*^sin^_2_ − 2*U*^sin^_4_ that yields zero force at the equilibrium geometry and is orthogonal to the other six independent torsion modes.

Finally, it is useful to scale each of the independent torsion modes so that its root-mean-squared deviation from its average value is 
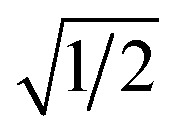
. This puts all of the different independent torsion modes on the same normalization scale. Considering only the dihedral dependence, this gives the first seven independent torsion modes of the rotatable dihedral torsion potential:122
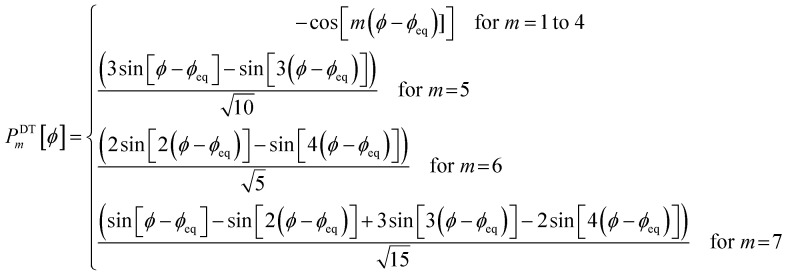
123
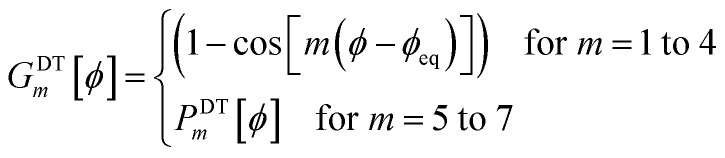
where *ϕ* is the current dihedral angle, and *ϕ*_eq_ is the reference dihedral angle from the equilibrium state of the structure. *P*^DT^_*m*_[*ϕ*] is defined to have an average value of zero:

124 *P*^DT^_*m*_[*ϕ*] = *G*^DT^_*m*_[*ϕ*] − *G*^DT,avg^_*m*_

125
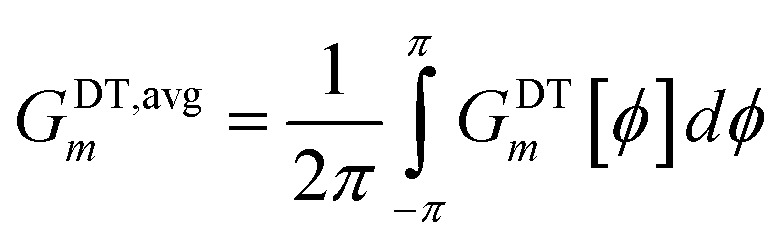


**Fig. 4:**
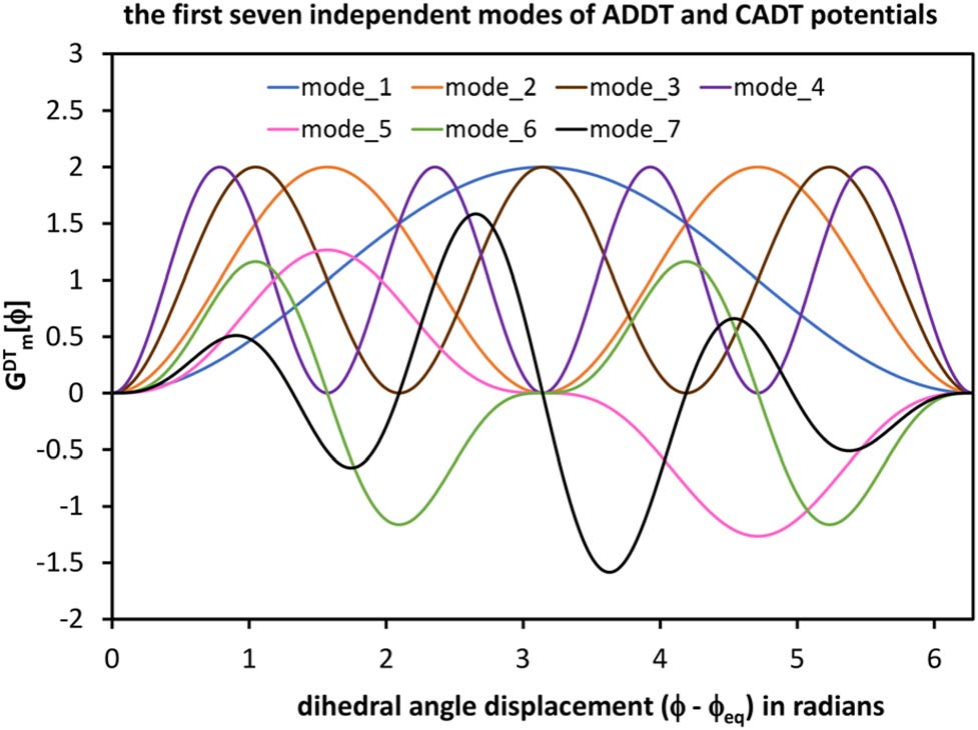
Plot showing the dihedral torsion potential *G*^DT^_*m*_[*ϕ*] for each mode *versus* the dihedral displacement. Modes 1 to 4 are cosine modes, and modes 5 to 7 are sine modes.

Fig. 4 plots each of these seven modes for expanding the dihedral torsion potential function.

Including the torsion offset potential and the angle-damping factors, this yields the following seven ADDT modes:126
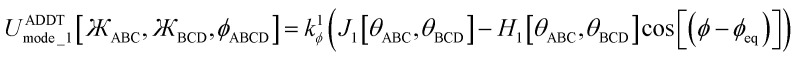
127
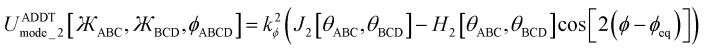
128
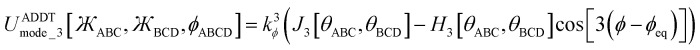
129
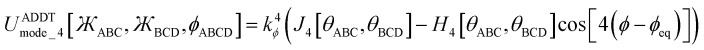
130
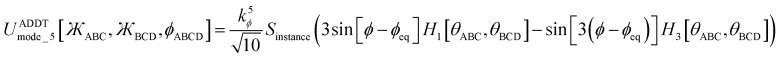
131
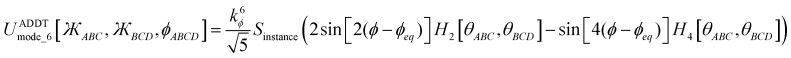
132
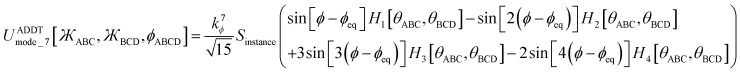


After dihedral mode smart selection (see Section 7), this yields133
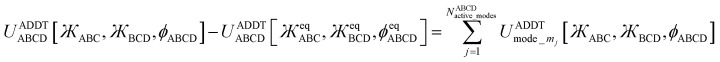
where *N*^ABCD^_active_modes_ is the number of active modes for dihedral ABCD.

## 5. Constant amplitude dihedral torsion (CADT) model potential

As shown in Section 10.2 below, angles with *θ*^eq^_ABC_ < 130° are typically much stiffer than angles with *θ*^eq^_ABC_ > 160°. As a result of this increased stiffness and also that they are farther from 180° to begin with, it is relatively unlikely that angles having *θ*^eq^_ABC_ < 130° will reach 180° during a typical classical molecular dynamics simulation. Also, the relatively large increase in energy from *θ*^eq^_ABC_ < 130° to *θ*^eq^_ABC_ = 180° makes reaching 180° not ‘thermally accessible’ during classical Monte Carlo simulations. Depending on the situation, there may or may not be a ground state crossover as the ABC angle changes from *θ*_ABC_ < 130° to *θ*_ABC_ > 160°. For example, carbon atoms having sp^2^ hybridization typically have bond angles of ∼120° while carbon atoms having sp^1^ hybridization typically have bond angles of ∼180°. Consequently, angles having *θ*^eq^_ABC_ < 130° are in some sense ‘far removed’ from the dynamics near *θ*_ABC_ ≈ 180°.

Why set this angle threshold value at 130°? Since sp^2^ hybridization (which typically yields ∼120° bond angles) is fundamentally different than sp^1^ hybridization (which typically yields ∼180° bond angles), we infer the angle threshold value should be >120° and <180°. Since bond angles >135° are closer to 180° (parallel bonds) than to 90° (perpendicular bonds), it follows that the angle threshold should probably be set to a value ≤ 135°. Now, sp^2^ hybridized angles will exhibit some statistical fluctuations giving some sp^2^ hybridized equilibrium bond angle values slightly larger than 120° and also some others slightly smaller than 120°. Because the angle threshold value should not divide this group, it follows that the angle threshold value should be greater than or equal to approximately 128°. Now, between the feasible range of approximately 128° to 135°, the precise value of the angle threshold value is a judgement call. An angle threshold value of 130° was chosen as a round number that maximizes the size of the region treated by the ADDT potential.

Accordingly, if both *θ*^eq^_ABC_ < 130° and *θ*^eq^_BCD_ < 130°, then it is reasonable to make the simplifications

134 *f*^ABC^_*n*_ ≈ *f*^ABC^_*n*_eq_ ≈ *f*^ABC^_1_ ≈ *f*^ABC^_1_eq_

135 *f*^BCD^_*n*_ ≈ *f*^BCD^_*n*_eq_ ≈ *f*^BCD^_1_ ≈ *f*^BCD^_1_eq_

for *n* = 1, 2, 3, 4. Eqn (134) results both from the asymptotic matching (as described in Section 2.3 above) and from the relatively stiff ABC bond angle which causes *f*^ABC^_*n*_ ≈ *f*^ABC^_*n*_eq_. Eqn (135) results from analogous properties for the BCD bond angle. This yields the approximation

136 *J*_*n*_[*θ*_ABC_,*θ*_BCD_] ≈ *H*_*n*_[*θ*_ABC_,*θ*_BCD_] ≈ 1

These simplifications produce the following constant amplitude dihedral torsion (CADT) potential energy modes:137
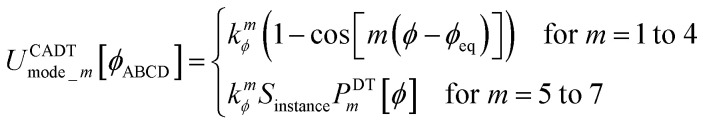


The CADT potential has a force discontinuity if either *θ*_ABC_ = 180° or *θ*_BCD_ = 180°, but those angle values are energetically unlikely to be reached during typical molecular dynamics or Monte Carlo simulations when both *θ*^eq^_ABC_ < 130° and *θ*^eq^_BCD_ < 130°.

As explained in the companion article, dihedral types sharing the same middle bond instances can be ‘pruned’ so that one representative dihedral type is retained per middle bond type in the forcefield.^18^ This dihedral pruning preserves symmetry equivalency.^18^ As explained in the companion article, this should preferably be done so that the retained representative dihedral type simultaneously has a relatively modest number of instances and relatively large values of (180° − *θ*^eq^_ABC_) and (180° − *θ*^eq^_BCD_).^18^ For most materials, the practical consequence is that the vast majority of dihedrals retained after pruning will use the CADT potentials instead of the ADDT potentials.

As explained in Section 7, dihedral mode smart selection for the CADT potential follows exactly the same procedure and equations as for the ADDT potential. This yields138
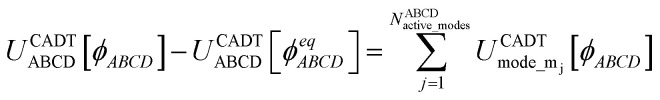
where *N*^ABCD^_active_modes_ is the number of active modes for dihedral ABCD.

After parameterization, the CADT potential can be equivalently rewritten as the dihedral potential Fourier series:139
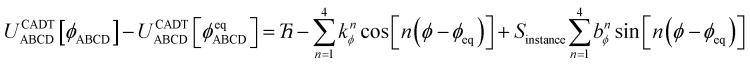
where140
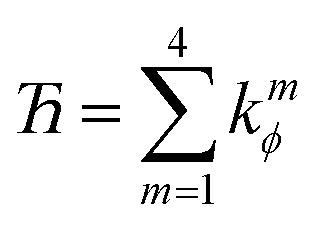
141
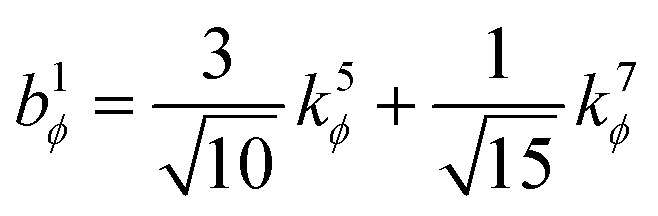
142
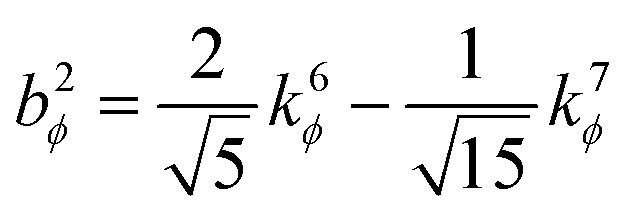
143
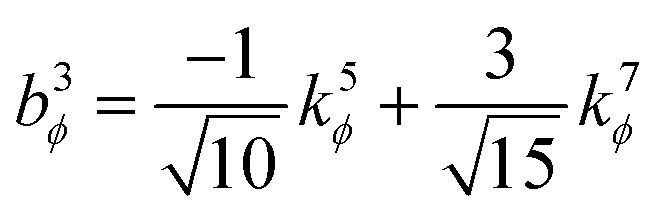
144
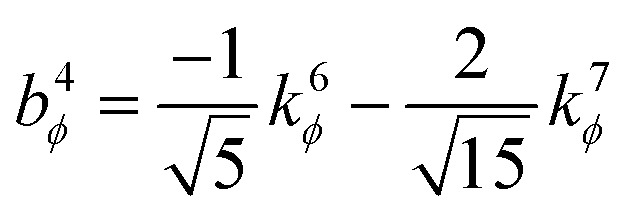
Depending on the particular situation, some terms in eqn (139) may be zero (*i.e.*, omitted) as the consequence of dihedral mode smart selection. In this work, the above series runs up to *n*_ma*x*_ = 4 and spans the full subspace associated with those dihedral potential modes subject to the constraint that *U*^CADT^_ABCD_[*ϕ*^eq^_ABCD_] = 0 is a stationary point (*i.e.*, has first derivative equal to zero). It is possible (albeit not usually necessary) to include higher than *n* = 4 terms in the CADT potential and Fourier series expansion.

## 6. The ADCO and CACO torsion model potentials

Due to symmetry, some dihedrals have a torsion potential that is an even function of the dihedral value as shown in eqn (81). For this dihedral type, it is advantageous to use a model torsion potential that has this same symmetry. Considering only the dihedral dependence, the cosine-only torsion model potentials have the following independent torsion modes:

145 *P*^CO^_*n*_[*ϕ*] = cos[*nϕ*]

146 *G*^CO^_*n*_[*ϕ*] = cos[*nϕ*] − cos[*nϕ*^training^_eq_]

Including the torsion offset potential and the angle-damping factors yields the angle-damped cosine only (ADCO) model potential:147
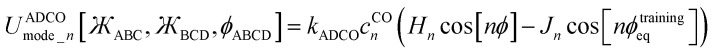
where *k*_ADCO_ is an adjustable force constant and *c*^CO^_*n*_ is the smart selection coefficient for mode *n*. For each ADCO dihedral type, there is one adjustable force constant *k*_ADCO_ irrespective of how many ADCO modes were smart selected; the ratios between the individual ADCO modes are controlled by {*c*^CO^_*n*_}. Here, *ϕ*^training^_eq_ is the equilibrium dihedral value in the QM-optimized low-energy ground-state geometry of the training dataset. After dihedral mode smart selection (see Section 7), this yields148
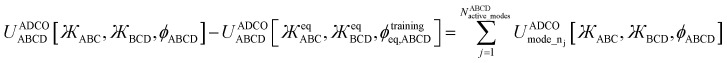
where *N*^ABCD^_active_modes_ is the number of active modes for dihedral ABCD.

Just as described in Section 5 above, if both *θ*^eq^_ABC_ < 130° and *θ*^eq^_BCD_ < 130°, then it is preferable to use the constant amplitude approximation shown in eqn (134)–(136). This simplification yields the constant amplitude cosine only (CACO) model potential:

149 *U*^CACO^_mode_*n*_[*ϕ*_ABCD_] = *k*_CACO_*c*^CO^_*n*_(cos[*nϕ*] − cos[*nϕ*^training^_eq_])

where *k*_CACO_ is an adjustable force constant and *c*^CO^_*n*_ is the smart selection coefficient for mode *n*. For each CACO dihedral type, there is one adjustable force constant *k*_CACO_ irrespective of how many CACO modes were smart selected; the ratios between the individual CACO modes are controlled by {*c*^CO^_*n*_}. After dihedral mode smart selection (see Section 7), this yields150
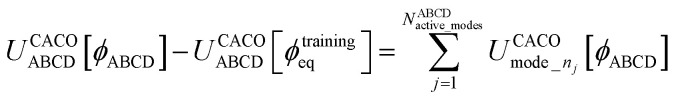
where *N*^ABCD^_active_modes_ is the number of active modes for dihedral ABCD.

If sin[*ϕ*_eq_] ≠ 0, due to series truncation (*i.e.*, only including the most important torsion modes) the ADDT and CADT model potentials typically do not yield strictly even torsion potentials even when the molecule itself has such an underlying molecular symmetry. For example, the CADT model potential does not yield a strictly even torsion potential for the hydrogen peroxide (HOOH) molecule, even though the HOOH molecule itself has such an underlying molecular symmetry. Because the CADT model potential has mismatched symmetry for this molecule, it yields a different position of the equilibrium dihedral value on the positive and negative dihedral sides. (This is explicitly shown in Section 10.8 and Table 16 below.) The ADCO and CACO model potentials address this problem by imposing even function symmetry on the torsion model potential. However, the equilibrium dihedral value of the ADCO and CACO model potentials (aka *ϕ*^FF^_eq_) may not exactly match that of the QM-optimized training geometry:

151 *ϕ*^FF^_eq_ ≈ *ϕ*^training^_eq_

due to truncation of the ADCO or CACO model potential. As more modes are included in the ADCO and CACO model potentials, *ϕ*^FF^_eq_ should asymptotically approach *ϕ*^training^_eq_ when the true dihedral torsion potential is an even function and the training dataset is a complete torsion scan. As more torsion modes are included in the ADDT and CADT model potentials, these should asymptotically approach the same torsion potentials as the ADCO and CACO model potentials, respectively, when the true dihedral torsion potential is an even function.

Why do the ADCO and CACO model potentials include the products *k*_ADCO_*c*^CO^_*n*_ and *k*_CACO_*c*^CO^_*n*_, respectively, instead of having an independently adjustable force constant value for each torsion mode? The specific reason for this is to more accurately reproduce *ϕ*^FF^_eq_ ≈ *ϕ*^training^_eq_. By fitting the {*c*_*n*_} explicitly to a complete torsion scan for a chosen dihedral instance during smart mode selection, this optimizes the ratios between these coefficients to more accurately reproduce *ϕ*^FF^_eq_ ≈ *ϕ*^training^_eq_, because the exact potential energy for such a torsion scan precisely corresponds to a Fourier series expansion. The *k*_ADCO_ or *k*_CACO_ value is subsequently computed during force constant optimization involving the full training dataset. If the force constant for each ADCO or CACO torsion mode were to be optimized independently to the full training dataset, this would likely result in less accurate values for *ϕ*^FF^_eq_ ≈ *ϕ*^training^_eq_, because the exact potential energy for such a training dataset does not necessarily correspond to a Fourier series expansion.

## 7. Selecting torsion model potentials and torsion modes

### 7.1 Selecting the best torsion model potential for each dihedral type

**Fig. 5:**
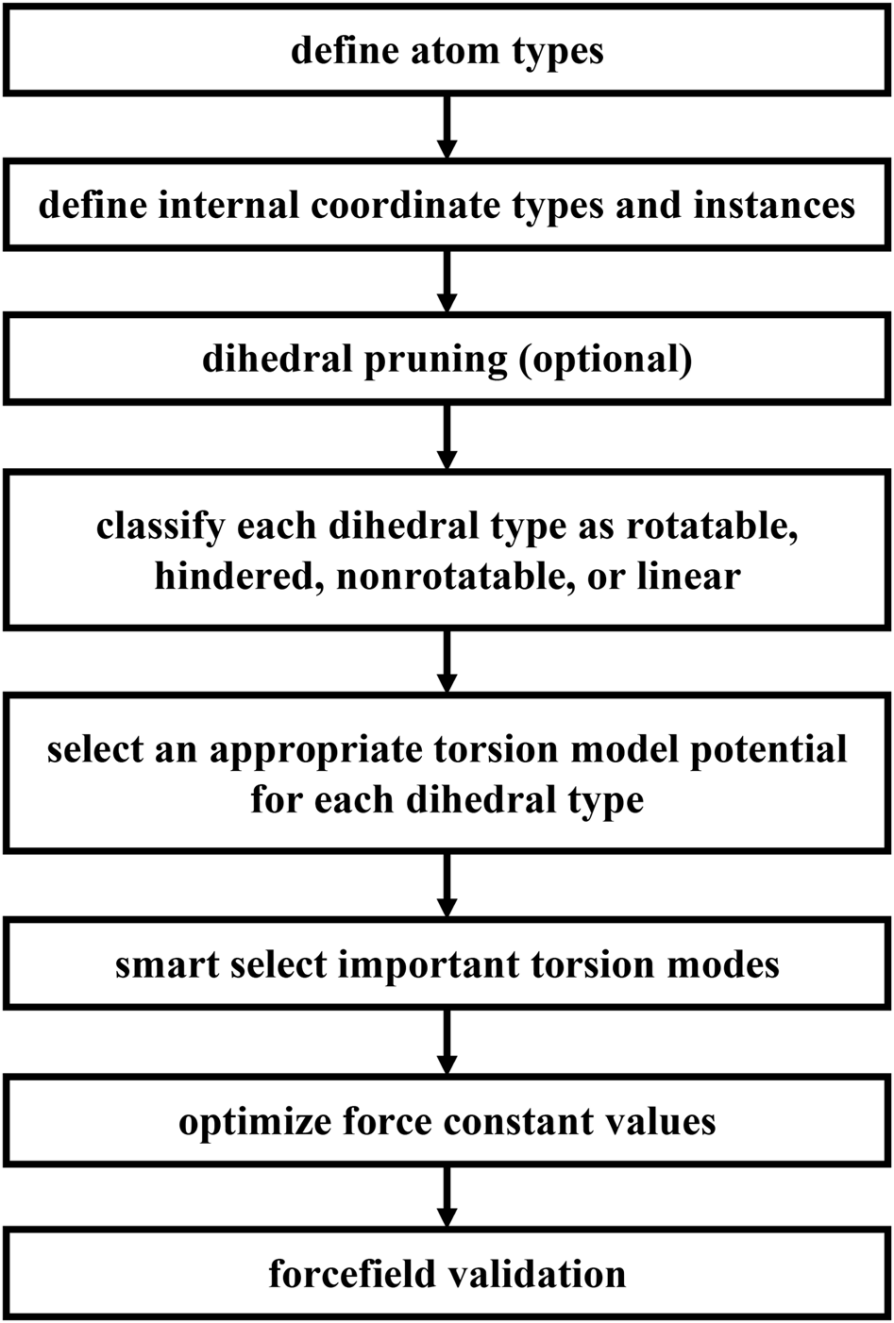
Overall sequence of steps for defining dihedral types, classifying them, and selecting appropriate torsion model potentials and modes to construct and validate a flexible forcefield.

Fig. 5 summarizes the overall process for constructing and validating dihedral torsion potentials. The process begins with defining atom types in the material. The second-neighbor-based atom types of Chen and Manz^24^ are recommended; however, this is not the only feasible choice of atom types. As explained in a companion article, the next step defines the list of active internal coordinate types and instances.^18^ This list of active internal coordinates includes the bond stretches, (optionally) Urey–Bradley stretches, angle bends, dihedral torsions, (optionally) cross-terms, and (optionally) concurrence terms.^18^ Dihedral pruning can be used to reduce excessive internal coordinate redundancy.^18^

Each dihedral type should be classified as rotatable, hindered, nonrotatable, or linear. A companion article gives a particular method to classify each dihedral type as non-rotatable, rotatable, hindered, or linear.^18,19^ According to this particular classification, a non-rotatable dihedral has a middle bond that is part of a bonded ring. The middle bond for a hindered dihedral is not part of any bonded ring; however, a hindered dihedral cannot be rigidly rotated over the entire range −π < *ϕ*_ABCD_ ≤ π without changing the material's bond connectivity.^18,19^ For example, two or more atoms may sterically collide with each other thereby preventing the hindered dihedral from accessing that part of configuration space.^18^

**Fig. 6:**
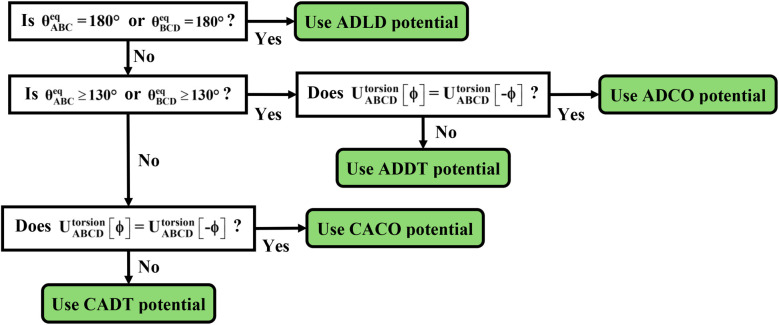
Flowchart illustrating the procedure to select an appropriate torsion model potential for each dihedral type.

The next step is to select an appropriate torsion model potential for each dihedral type. Fig. 6 illustrates the recommended procedure for doing this. Although the ADDT (or ADCO if *U*^torsion^_ABCD_[*ϕ*] = *U*^torsion^_ABCD_[−*ϕ*]) model potential can be applied to dihedral types for which (*θ*^eq^_ABC_ and *θ*^eq^_BCD_) < 130°, the greater simplicity of the CADT (or CACO if *U*^torsion^_ABCD_[*ϕ*] = *U*^torsion^_ABCD_[−*ϕ*]) model potentials favors their use in this case. Although classical nonreactive forcefields employing dihedral torsion model potentials have been used for several decades, the procedure shown in Fig. 6 is a transformative improvement over prior approaches.

After the kind of torsion model potential has been selected for each dihedral type, the next step is to smart select the important torsion modes. Here, the term ‘smart selection’ means the important torsion modes are kept while the unimportant torsion modes are omitted from the torsion model potential. For rotatable dihedral types, Section 7.2 explains a procedure for rotatable dihedral mode smart selection using torsion scans. For linear dihedral types, Section 9.2 explains a procedure for selecting specific modes from the linear dihedral torsion potential. Due to the limited range of motion of nonrotatable dihedral types, these can typically be described by the first cosine mode of the ADDT (if (*θ*^eq^_ABC_ or *θ*^eq^_BCD_) ≥ 130°) or CADT (if (*θ*^eq^_ABC_ and *θ*^eq^_BCD_) < 130°) model potential.^18^

Hindered dihedral types require some special consideration. As explained in a companion article, a dihedral type is classified as ‘hindered’ iff (a) it is not part of a bonded ring and (b) rigid rotation about its middle bond causes one part of the structure to sterically collide with another part of the structure. Closer examination reveals that such collisions may have two limiting cases. In case # 1, the collision is ‘unavoidable’, because the colliding groups are structurally tethered in ways that do not allow them to easily pass each other. In case # 2, the collision is ‘accidental’ and potentially avoidable. Consider long chain-like molecules comprising polymers, large biomolecules (*e.g.*, proteins, RNA, enzymes, fatty acids, polysaccharides, phospholipids, *etc.*), hydrocarbon chains (*e.g.*, petroleum), *etc.* In these cases, one end of the chain may ‘accidently’ collide with another part of the chain when rigidly rotated (about a dihedral middle bond) due to the particular manner in which the flexible chain has been ‘folded’. Let us define a ‘range of dihedral rotation’ as the connected range of *ϕ*_ABCD_ values that are not sterically prevented; this refers to the range of *ϕ*_ABCD_ values that can be reached *via* continuous *relaxed* displacements away from *ϕ*^eq^_ABCD_ avoiding steric collision. If this range of rotation about the hindered dihedral's middle bond is severely limited (such as sometimes occurs in case # 1), then it may be adequate to describe this particular hindered dihedral type by the first cosine mode of the ADDT (if (*θ*^eq^_ABC_ or *θ*^eq^_BCD_) ≥ 130°) or CADT (if (*θ*^eq^_ABC_ and *θ*^eq^_BCD_) < 130°) model potential.^18^ On the other if this range of rotation about the hindered dihedral's middle bond is large (such as sometimes occurs in case # 2), then it may be desirable to retain several torsion modes of the ADDT, CADT, ADCO, or CACO model potential to describe this hindered dihedral.

The next step is to optimize the values of all of the force constants in the flexibility model. For reasons explained in companion articles, these should be optimized simultaneously by fitting to a training dataset, rather than optimized sequentially one-at-a-time.^18,20^ Finally, the parameterized forcefield should be validated using an appropriate validation dataset that is separate from and independent of the training dataset.

### 7.2 Rotatable dihedral mode smart selection using torsion scan curves

A relaxed torsion scan curve changes the value of the dihedral *ϕ*_ABCD_ from −π to π in small increments while allowing all other independent geometric parameters (*e.g.*, bond lengths and bond angles) to reoptimize. Each geometry in a relaxed torsion scan is computed from constrained geometry optimization that produces the lowest energy possible for that particular constrained dihedral value.

A rigid torsion scan curve changes the value of the dihedral *ϕ*_ABCD_ from −π to π in small increments while keeping all of the bond lengths and bond angles fixed at their reference values. Unless otherwise specified, these reference values are the corresponding bond length and bond angle values in the material's optimized (low-energy) ground-state geometry. A rigid torsion scan is a series of single-point energy calculations performed at rigid geometries. There are no changes in the values of the angle-damping factors during rigid torsion scans. In general, a complete rigid torsion scan curve is possible if the dihedral is rotatable, but not if the dihedral is non-rotatable or hindered. We did not use rigid torsion scan energy curves for non-rotatable or hindered dihedrals, because these dihedrals have a limited (aka ‘restricted’) range of motion.^18^

For a rotatable dihedral, the potential energy along a torsion scan curve can be modeled by projecting onto an orthonormal basis of independent torsion modes:152
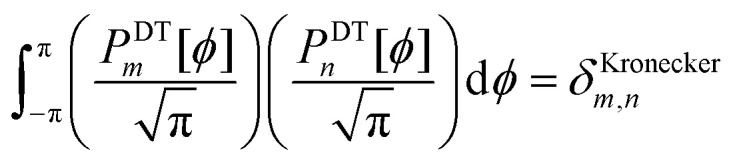
153
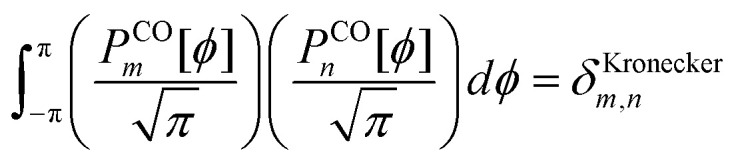


Let *E*^QM^_RTS_[*ϕ*] be the QM-computed energy of the material along the torsion scan curve for rotatable dihedral ABCD. This torsion scan curve is conducted using *T* dihedral values equally spaced over the range (−π,π]. Let *E*^QM_avg^_RTS_ be the average value154
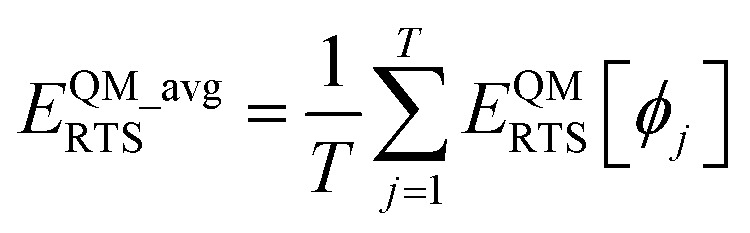
and *w* the self-overlap integral155
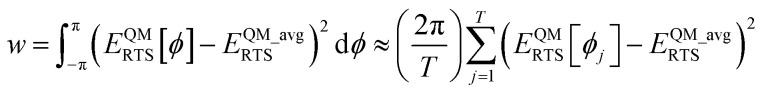


The ‘QM torsion norm’ is the root-mean-squared value of (*E*^QM^_RTS_[*ϕ*] − *E*^QM_avg^_RTS_):156
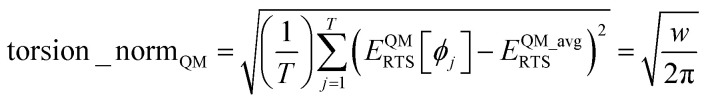


The QM torsion barrier is defined as the maximum energy minus the minimum energy along the torsion scan curve:157
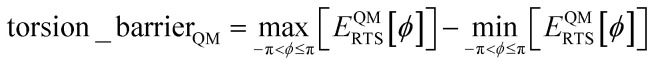


Using a complete set of dihedral-torsion (DT) projectors, the QM potential for the torsion scan curve can be expanded in terms of the orthogonal basis set as158
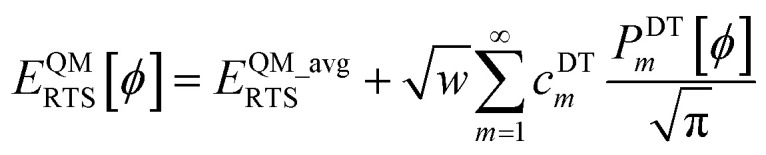


Since the cosine-only (CO) projectors are even functions of *ϕ*, the CO projectors can provide a complete expansion of the QM potential along the torsion scan curve iff the following symmetry descriptor159
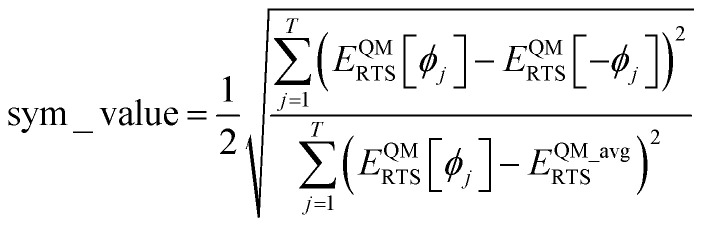
equals zero. Iff sym_value equals zero, then in this case the QM potential can be expanded using the CO projectors as160
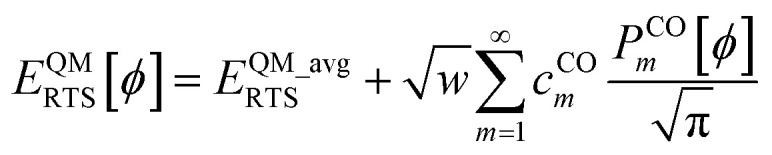


The model potential for the torsion scan curve can be expanded in terms of the orthogonal basis set as161
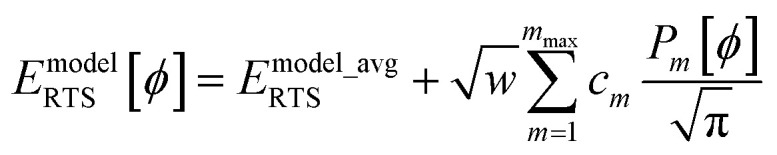
162
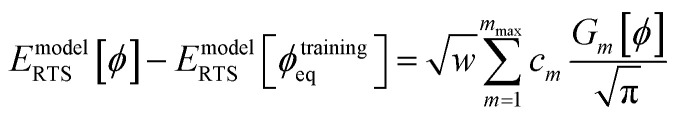
where163
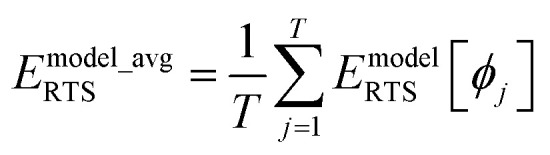
For the ADDT and CADT model potentials, *P*_m_ = *P*^DT^_m_ is used in eqn (161) and (164), and *G*_m_ = *G*^DT^_m_ is used in eqn (162). For the ADCO and CACO model potentials, *P*_m_ = *P*^CO^_m_ is used in eqn (161) and (164), and *G*_m_ = *G*^CO^_m_ is used in eqn (162). The coefficients are given by164
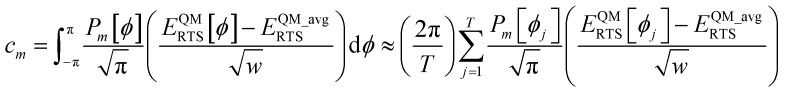


For the torsion modes included in the model potential, the expansion coefficients {*c*_m_} are the same for the QM and model potentials along the torsion scan curve. The model potential neglects some of the less important (*i.e.*, negligible) torsion modes while the QM potential is formally expanded (see eqn (158)) as a nontruncated sum over all possible torsion modes.

The ‘model torsion norm’ is the root-mean-squared value of (*E*^model^_RTS_[*ϕ*_*j*_] − *E*^model_avg^_RTS_):165
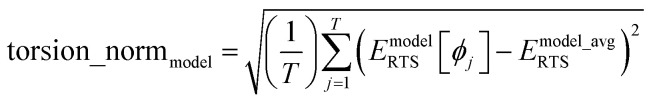


As derived in ESI Section S12,† the QM and model torsion norms are related by166
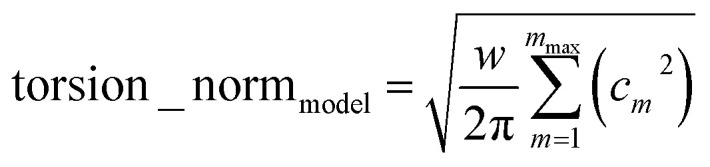
167
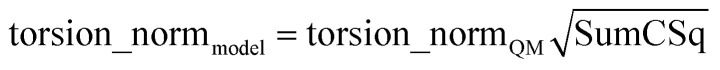
where SumCSq is defined as168
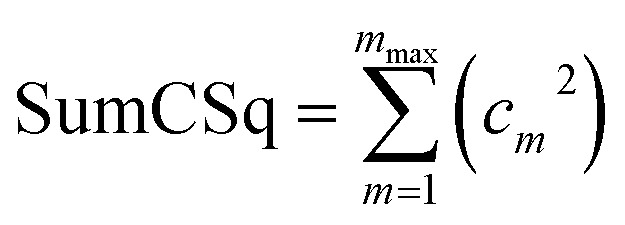


Since {*c*_*m*_} are the coefficients for projection onto an orthonormal basis set, it follows that

169 0 ≤ SumCSq ≤ 1

As derived in ESI Section S12,† the full set of mode coefficients for the normalized QM potential along the torsion scan curve satisfies170
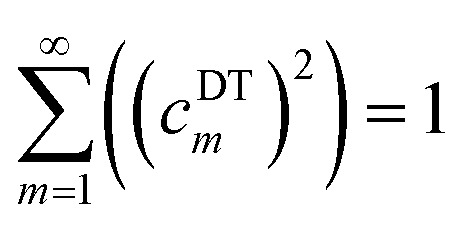
Hence, the SumCSq value can be interpreted as the fraction of the QM torsion scan curve that is recovered by the model potential. Iff SumCSq ≈ 1 and *E*^model_avg^_RTS_ ≈ *E*^QM_avg^_RTS_, then the model potential provides an adequate approximation of the QM potential along the torsion scan curve:

171 *E*^model^_RTS_[*ϕ*] ≈ *E*^QM^_RTS_[*ϕ*] − *E*^QM^_RTS_[*ϕ*^training^_eq_]

Here, we consider the general case in which *E*^model_avg^_RTS_ may potentially be different in value than *E*^QM_avg^_RTS_. This leads to three scenarios. Scenario #1: this scenario chooses the value of *E*^model_avg^_RTS_ such that

172 *E*^model^_RTS_[*ϕ*^training^_eq_] = 0

which makes the left and right sides of eqn (171) exactly equal to each other at the point *ϕ* = *ϕ*^training^_eq_. For the ADDT and CADT model potentials, this scenario corresponds to the choice173
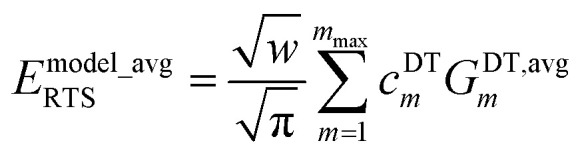
For the ADCO and CACO model potentials, this scenario corresponds to the choice174
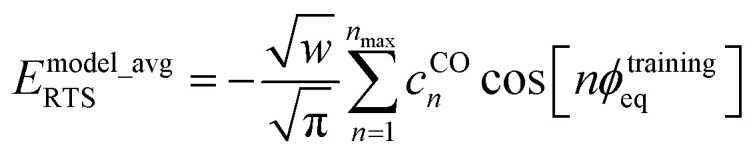


Scenario #2: this scenario chooses the value of *E*^model_avg^_RTS_ such that

175 *E*^model_avg^_RTS_ = *E*^QM_avg^_RTS_ − *E*^QM^_RTS_[*ϕ*^training^_eq_]

which makes the averages of the left and right sides of eqn (171) exactly equal to each other. Using a complete set of orthonormal DT projectors to expand the QM potential along the torsion scan curve yields the untruncated expansion:176
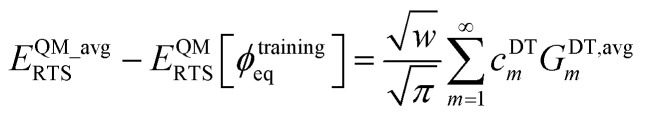


Scenario #3: this scenario (denoted as “other”) encompasses any situations in which *E*^model_avg^_RTS_ is chosen to satisfy any criteria different from Scenarios #1 and #2 described above.

Comparing eqn (173), (175), and (176), the difference in *E*^model_avg^_RTS_ for scenarios #1 and #2 using DT projectors is177
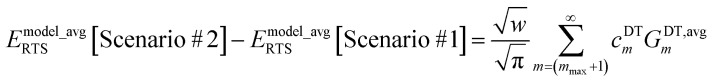
Examining eqn (177), *E*^model_avg^_RTS_ exactly coincides for the two scenarios if the modal coefficients *c*_*m*_ are zero for all values of *m* > *m*_max_. If the modal coefficients *c*^DT^_*m*_ are nearly zero for all values of *m* > *m*_max_, then *E*^model_avg^_RTS_ approximately coincides for the two scenarios.

The situation is slightly more complicated for the CO projectors than for the DT projectors. If *sym*_value (see eqn (159)) equals zero, then in this case the average QM potential can be expanded as178
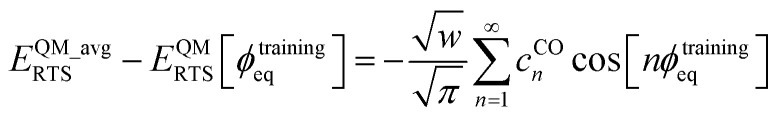
Comparing eqn (174), (175), and (178), the difference in *E*^model_avg^_RTS_ for scenarios #1 and #2 using CO projectors when *sym*_value = 0 is179
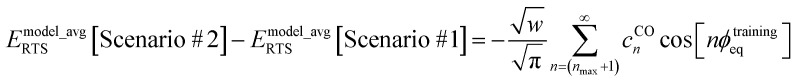


When *sym*_value ≠ 0, eqn (178) and (179) do not apply. If *sym*_value is large, then the CO projectors might give a large difference in *E*^model_avg^_RTS_ value between scenarios #1 and #2.

For a torsion scan curve, the *R*-squared value is computed *via*eqn (17) using the following definitions:180
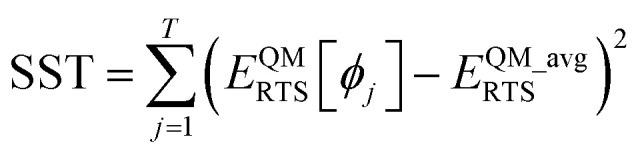
181
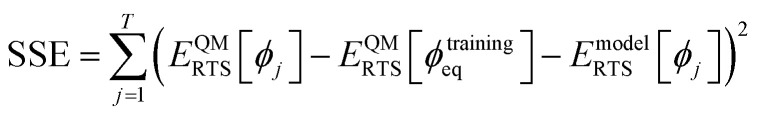
As derived in ESI Section S12,† this *R*-squared value can be equivalently rewritten as182
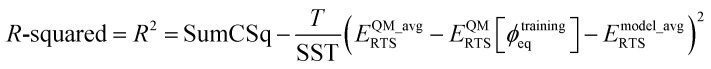
Examining eqn (182),

183 *R*-squared ≤ SumCSq

For Scenario #2, combining eqn (175) and (182) shows that

184 *R*-squared = *R*^2^ = SumCSq

Eqn (184) holds in Scenario #2 irrespective of whether DT or CO projectors are used and irrespective of the value of *sym*_value, but of course the value of SumCSq (and hence of *R*-squared) is impacted by which projectors are used.

As derived in ESI Section S12,† for Scenario #1 eqn (182) is rewritten as185
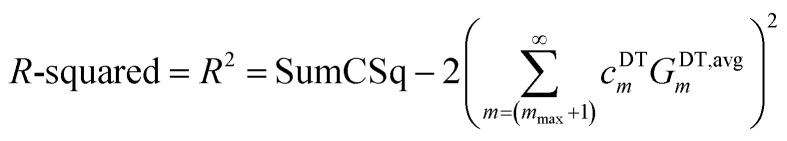
Thus when using Scenario #1, *R*-squared is close to SumCSq when *c*^DT^_*m*_ for every omitted torsion mode is close to zero. Iff *sym*_value = 0, then an analogous derivation using the CO projectors for scenario #1 yields186
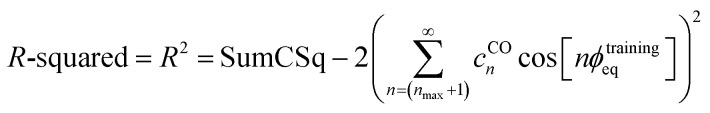


When computing the *R*-squared value as described above, all *T* geometries along the torsion scan curve had equal observation weights. In classical molecular dynamics or Monte Carlo simulations employing the NPT, NVT, μPT, or μVT thermodynamic ensembles, the lower-energy geometries should appear more often (*i.e.*, have higher observation weights) than higher-energy geometries. When every geometry along the torsion scan curve has equal observation weights, scenario #2 described above has a higher *R*-squared value (see eqn (184)) than scenario #1 (see eqn (185)). Scenario #1 (see eqn (172)) exactly matches the relative energy of the QM and model potentials at the training dataset's optimized ground-state geometry. For this reason, scenario #1 typically performs better than scenario #2 when employing the NPT, NVT, μPT, or μVT thermodynamic ensembles, because scenario #1 gives smaller errors than scenario #2 for the lower-energy geometries that receive relatively higher observation weights in such ensembles. For this reason, my ADDT, CADT, ADCO, and CACO model potentials are typically constructed according to scenario #1 rather than according to scenario #2. Except where otherwise indicated, the ADDT, CADT, ADCO, and CACO model potentials were constructed according to scenario #1 throughout this work.

In this work and a companion article,^19^ we used the following smart selection thresholds. If *sym*_value ≤ 0.01, this means *U*^torsion^_ABCD_[*ϕ*] = *U*^torsion^_ABCD_[−*ϕ*] within the tolerance, so the ADCO or CACO model potential was used. In this case, an ADCO or CACO mode was kept if ab*s*[*c*_*m*_] > 0.001. Keeping the ADCO or CACO coefficients greater than this ‘very tight’ cutoff helps *ϕ*^FF^_eq_ more closely approach *ϕ*^training^_eq_. If 0.01 < *sym*_value ≤ 0.1, the ADDT or CADT model potential was used, and an ADDT or CADT mode was kept if abs[*c*_*m*_] > 0.01. This case corresponds to the situation in which *U*^torsion^_ABCD_[*ϕ*] is approximately but not strictly equal to *U*^torsion^_ABCD_[−*ϕ*], so it is beneficial to use a ‘tight’ cutoff (*i.e.*, abs[*c*_*m*_] > 0.01) for retaining torsion modes to achieve a balance between accuracy and conciseness. This ‘tight’ cutoff helps the ADDT or CADT model potential to more accurately reproduce the position of the alternate local energy minimum *ϕ*^alternate^_eq_ ≈ −*ϕ*_eq_. If 0.1 < *sym*_value, the ADDT or CADT model potential was used, and an ADDT or CADT mode was kept if abs[*c*_*m*_] > 0.1. This case corresponds to the situation in which *U*^torsion^_ABCD_[*ϕ*] is not approximately equal to *U*^torsion^_ABCD_[−*ϕ*], so conciseness of the torsion modes is preferred. Examining eqn (168), this ‘normal’ cutoff (*i.e.*, *abs*[*c*_*m*_] > 0.1) neglects a torsion mode if it affects the SumCSq value by ≤ 0.01.

The coefficients *k*^*m*^_*ϕ*_, *c*_*m*_, and {*b*^*n*^_*ϕ*_} have the following distinct meanings. *k*^*m*^_*ϕ*_ is the force constant for torsion mode *m*. 
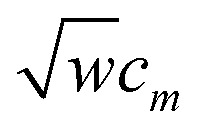
 is the projection coefficient of mode *m* for a torsion scan of one dihedral instance, where 
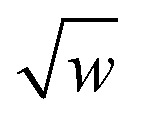
 is the torsion scan's normalization coefficient and *c*_*m*_ is the normalized function's projection onto torsion mode *m*. In contrast to the coefficients {*c*_*m*_} which refer to an individual torsion scan, the force constants {*k*^*m*^_*ϕ*_} apply to all motions of the system. Because of multicollinearity between some flexibility terms, there is no universal relationship between 
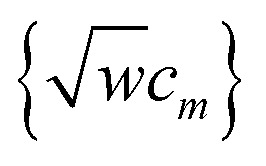
 and {*k*^*m*^_*ϕ*_}. As shown above, ADDT and CADT torsion mode 5 is composed of two sine functions, torsion mode 6 is composed of two sine functions, and torsion mode 7 is composed of four sine functions. The coefficients {*b*^*n*^_*ϕ*_} are the coefficients for individual sine functions, and these {*b*^*n*^_*ϕ*_} are computed from the force constants {*k*^*m*^_*ϕ*_} of ADDT or CADT torsion modes *m* = 5, 6, and 7 using the formulas shown in eqn (141)–(144).

## 8. How do the CADT and CACO model potentials compare to previously published class A torsion potentials?

The Class A (*i.e.*, ‘dihedral-only’) torsion potential of dihedral *ϕ*_ABCD_ can be expanded as a Fourier series expressed in either of two equivalent forms:187
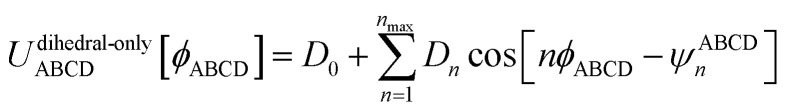
188
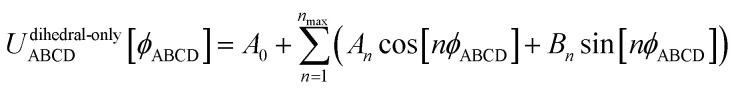
where *D*_0_, *D*_n_ (for *n* = 1, 2, 3, …), and *ψ*^ABC*D*^_*n*_ are adjustable (aka ‘fitted’) parameters that define the potential. Here, *n*_ma*x*_ is the maximum value of *n* that is considered in the model. This Fourier series becomes formally complete in the limit *n*_max_ → ∞. Comparing eqn (26), (187), and (188) confirms that

189 *A*_0_ = *D*_0_

190 *A*_*n*≥1_ = *D*_*n*_cos[*ψ*^ABCD^_*n*_]

191 *B*_*n*_ = *D*_*n*_sin[*ψ*^ABCD^_*n*_]

If neither contained equilibrium bond angle is linear (*i.e.*, *θ*^eq^_ABC_ ≠ π and *θ*^eq^_BCD_ ≠ π), then it is possible to define the equilibrium dihedral value (*ϕ*^eq^_ABCD_), which is the value of *ϕ*_ABCD_ in the material's optimized ground-state (*i.e.*, low-energy) geometry. In this case, the Fourier series can be re-written as192
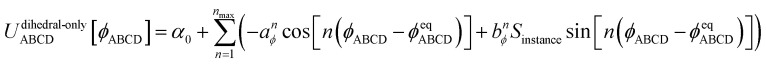
which is equivalent to eqn (187) and (188). Comparing eqn (26), (187), and (192) confirms that

193 *α*_0_ = *D*_0_

194 *a*^*n*^_*ϕ*_ = −*D*_*n*_ cos[*ψ*^ABCD^_*n*_ − *nϕ*^eq^_ABCD_]

195 *b*^*n*^_*ϕ*_*S*_instance_ = *D*_*n*_ sin[*ψ*^ABCD^_*n*_ − *nϕ*^eq^_ABCD_]

Defining the variable substitutions

196 *n*_max_ = 4

197 *k*^*n*^_*ϕ*_ = *a*^*n*^_*ϕ*_ for *n* = 1,2,3,4

198
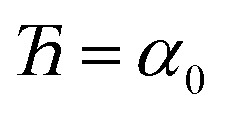
and substituting these into eqn (192) yields the Fourier series expansion shown in eqn (139). Applying the boundary condition199
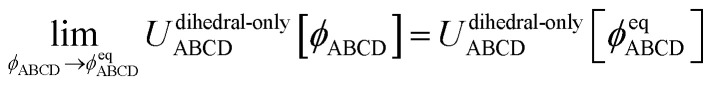
yields eqn (140). The boundary condition200
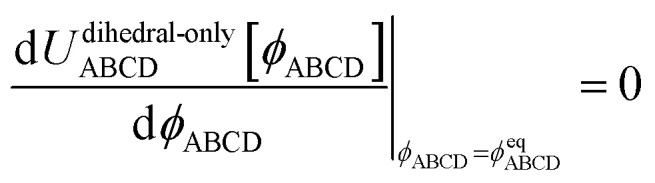
applies, because all of the atom-in-material forces are zero at the material's optimized ground-state geometry. This boundary condition is automatically satisfied for the constant 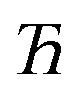 and the cosine modes in eqn (139). Applying the boundary condition of eqn (200) to the Fourier series shown in eqn (139) yields eqn (119). The constraint shown in eqn (119) has the effect of reducing the number of independent sine modes from *n*_max_ to (*n*_max_ − 1). Consider *n*_max_ = 4 as an example. In this case, there are *n*_max_ = 4 independent cosine modes and (*n*_max_ − 1) = 3 independent sine modes, which totals to 7 seven rotatable dihedral torsion modes.

From this, we conclude that the CADT potential described in Section 5 completely spans the degrees of freedom associated with the Fourier series expansion, which can be equivalently written in any of the following forms:

(i) Eqn (137) and (138)

(ii) Eqn (139)–(144), which is the same as eqn (192) with constraint (200),

(iii) Eqn (187) with constraint (200)

(iv) Eqn (188) with constraint (200)

The Fourier expansion form shown in eqn (187) is extremely inconvenient, because it requires non-linear regression to optimize the *ψ*^ABCD^_*n*_ parameters.^25^ Rewriting the Fourier expansion as shown in eqn (137), (138) or eqn (188) or eqn (192) avoids this problem.

Which parts of this Fourier series expansion were not captured by specific Class A torsion potentials used in the prior literature? Some prior literature^7,26,27^ used an expansion of the form

201 *U*^cos_pwrs^_ABCD_[*ϕ*_ABCD_] = *Λ*_0_ + *Λ*_1_ cos[*ϕ*_ABCD_] + *Λ*_2_ cos^2^[*ϕ*_ABCD_] + *Λ*_3_ cos^3^[*ϕ*_ABCD_] +…

which is an even function of *ϕ*_ABCD_. An expansion of powers of cosine is equivalent to an expansion involving various cosine multiplicities:

202 *U*^even^_ABCD_[*ϕ*_ABCD_] = *Γ*_0_ + *Γ*_1_ cos[*ϕ*_ABCD_] + *Γ*_2_ cos[2*ϕ*_ABCD_] + *Γ*_3_ cos[3*ϕ*_ABCD_] +…

which is functionally equivalent to the torsion potential form used in some common implementations of the OPLS-AA,^28^ AMBER,^8,29^ CHARMM^30^ and some other^5^ forcefields. For example, the OPLS-AA forcefield uses the following ‘cosine only’ torsion potential:^28^203
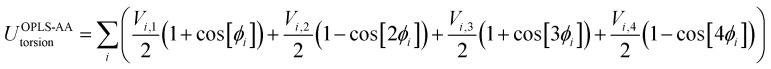
These ‘cosine only’ torsion model potentials are manifestly incomplete, because they omit all of the odd-function contributions to the torsion potential.

Burger *et al.*^6^ used a dihedral potential of the form204
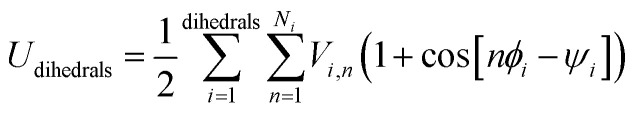
This type of model potential is incomplete and overly restrictive, because it uses the same *ψ*_*i*_ value for all *N*_i_ modes of dihedral *ϕ*_*i*_. Hopkins and Roitberg^25^ used a dihedral model potential of the form:205
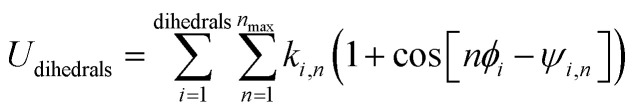
They also rewrote this in the form of eqn (188) except that they omitted the constant (*A*_0_) term. Comparing eqn (187) to (205) shows that eqn (205) provides a nearly complete Fourier series expansion of the torsion potential, except that eqn (205) is missing one degree of freedom in the constant part of the potential. This does not affect the forces.^25^ Radom *et al.*^3^ investigated a torsion potential that was a truncation of the Fourier series shown in eqn (188).

Some prior literature appears at first glance to use dihedral model potentials containing cos[*nϕ*_*i*_ − *ψ*_*i*,*n*_] terms. However, some of these forcefields impose the too-severe restriction that *ψ*_*i*,*n*_∈{0,180°}, which means that cos[*nϕ*_*i*_ − *ψ*_*i*,*n*_] → ±cos[*nϕ*_*i*_].^5^ In this case, the expansion is actually equivalent to the form shown in eqn (202), which is not a Fourier series expansion because it omits the odd-function contributions.

Some forcefields recommend using *ψ*_*i*,*n*_ ∈ {0,180°} even if they do not strictly impose it. “In CHARMM it is possible to use any value for the phase;^31^ however, it is strongly suggested that values of 0 and 180° be used as the parameters are then appropriate for different stereoisomers associated with a given dihedral.”^32^ However, this too-severe restriction often prevents the torsion model from accurately reproducing the torsion scan curve. My ADDT and CADT model potentials completely avoid this problem, because they allow different stereoisomers to be described by the same ADDT and CADT force constant values without omitting the odd-function contributions.

The CACO torsion model potential is functionally equivalent to eqn (202) with the following caveats. A least-squares fit of eqn (202) to a torsion scan curve optimizes the constant potential intercept *Γ*_0_ to match the average dihedral potential over the dihedral scan curve. The CACO torsion model potential adjusts the constant offset such that the model potential is zero when *ϕ* = *ϕ*^training^_eq_:

206 *U*^CACO^_ABCD_[*ϕ*^training^_eq_] = 0

Functionally, the CACO torsion model potential can be derived from eqn (202) as

207 *U*^CACO^_ABCD_[*ϕ*] = *U*^even^_ABCD_[*ϕ*] − *U*^even^_ABCD_[*ϕ*^training^_eq_]

with the additional caveat that the force constant for each CACO mode is expressed as *k*_CACO_*c*_*n*_. These particular choices ensure that CACO reproduces relative energies more accurately for the lower energy geometries (which appear more frequently during classical molecular dynamics and Monte Carlo simulations) and that *ϕ*^FF^_eq_ ≈ *ϕ*^training^_eq_.

## 9. Angle-damped linear dihedral (ADLD) model potential

### 9.1 Model potential for dihedrals containing at least one linear equilibrium bond angle

A dihedral containing one or two linear (or close to linear) equilibrium bond angles is called a ‘linear dihedral’. In this context, ‘close to linear’ means π − *θ*^eq^_ABC_ < *ε* or π − *θ*^eq^_BCD_ < *ε*, where *ε* is a tolerance (*e.g.*, *ε* = 0.03 radians was used in this work and the companion article^18,19^). Please note that the classification of whether or not a dihedral is a ‘linear dihedral’ is based on the values of *θ*^eq^_ABC_ and *θ*^eq^_BCD_ not the values of *θ*_ABC_ and *θ*_BCD_. For a linear dihedral, *ϕ*^eq^_ABCD_ is undefined and *f*^∢^_*n*≥1_eq_ = 0 for whichever equilibrium bond angle is linear.

The new ADLD model potential is derived starting from my general Class B torsion potential shown in eqn (87). The combined angle-dihedral coordinate branch equivalency condition applied to *H*_*n*_[*θ*_ABC_,*θ*_BCD_](*A*_*n*_cos[*nϕ*_ABCD_] + *B*_*n*_sin[*nϕ*_ABCD_]) requires that eqn (88) be satisfied. This can be accomplished by choosing the angle-damping functions multiplying cosine and sine terms in the ADLD potential to be

208 *Q*_*n*_[*θ*_ABC_,*θ*_BCD_] = *f*^ABC^_⌊*n*/2⌋_*f*^ABC^_⌈*n*/2⌉_*f*^BCD^_⌊*n*/2⌋_*f*^BCD^_⌈*n*/2⌉_

Note that these formulas involve no division by the equilibrium values. Here, ⌈*x*⌉ denotes ceiling[*x*], which is the smallest integer greater than or equal to *x*. As explained in a previous section, ⌊*x*⌋ denotes floor[*x*], which is the largest integer less than or equal to *x*.

For the ADLD model potential, an angle-dependent offset potential is required to ensure that the energy increases as the structure is displaced from its ground-state geometry. For an even mode, one could envision something like209
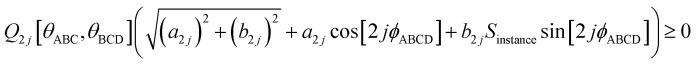
to ensure that the energy contribution is always non-negative. In eqn (209), the term that does not depend on *ϕ*_ABCD_ is the angle-dependent offset potential.

Because they are odd functions of *ϕ*_ABCD_, the sin[*nϕ*_ABCD_] terms involve the sign *S*_instance_ ∈ {0,−1,+1}. Different dihedral instances of the same dihedral type can have different values of *S*_instance_. Because *ϕ*_eq_ is undefined for linear dihedrals, the value of *S*_instance_ cannot be assigned using the sign of *ϕ*_eq_ for linear dihedrals. For linear dihedrals, the value of *S*_instance_ must be determined by detecting local mirror-image environments (*i.e.*, local chiral enantiomer environments) for different dihedral instances of the same dihedral type. Note that *S*_instance_ = 0 implies a mirror-image (*i.e.*, reflection) symmetry of the torsion potential.

Eqn (209) is not convenient, because it requires nonlinear regression to optimize the *a*_2*j*_ and *b*_2*j*_ values. To facilitate linear regression, it is convenient to instead expand each even mode as

210 *U*^ADLD^_2*j*_ = (*f*^ABC^_*j*_*f*^BCD^_*j*_)^2^(*k*^*j*^_LD1_(1 − cos[2*jϕ*_ABCD_]) + *k*^*j*^_LD2_(1 + cos[2*jϕ*_ABCD_]) + *k*^*j*^_LD3_*S*_instance_sin[2*jϕ*_ABCD_])

The coefficients *k*^*j*^_LD1_, *k*^*j*^_LD2_, and *k*^*j*^_LD3_ in eqn (210) can be optimized using linear regression, and the constraints

211 *k*^*j*^_LD1_ ≥ 0

212 *k*^*j*^_LD2_ ≥ 0

should be imposed during this linear regression. Eqn (209) can always be represented as a special case of eqn (210) by setting

213 *k*^*j*^_LD3_ = *b*_2*j*_

214
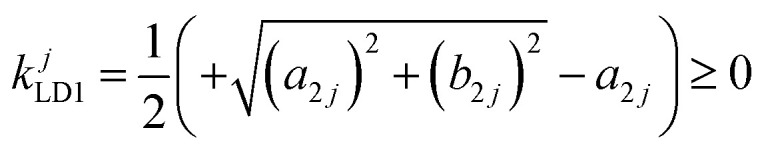
215
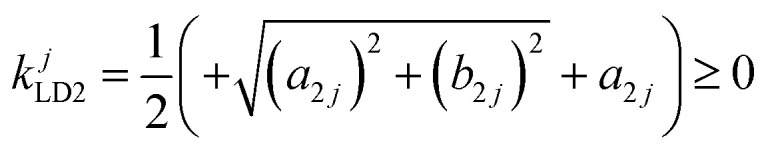


For odd modes, *Q*_2*j*−1_[*θ*_ABC_,*θ*_BCD_] multiplied by only a constant does not satisfy the combined angle-dihedral coordinate branch equivalency condition. To ensure that the energy increases as the structure is displaced from its ground-state geometry, instead of *Q*_2*j*−1_[*θ*_ABC_,*θ*_BCD_] we can use an angle-dependent offset potential of the form216
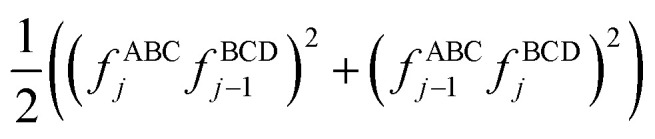
which does satisfy the combined angle-dihedral coordinate branch equivalency condition. The offset in eqn (216) arises from the following ‘completing the squares’:217
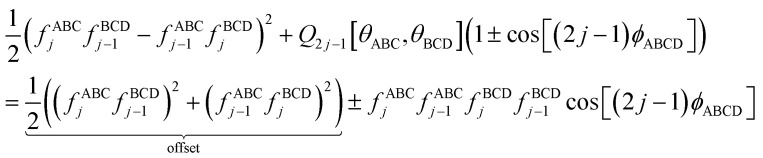
Because

218 0 ≤ |cos[(2*j* − 1)*ϕ*_ABCD_]| ≤ 1

it directly follows that219
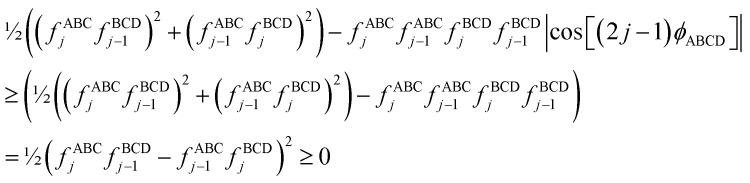


Somewhat analogous to the even modes, each odd mode is expanded using three force constants amenable to linear regression:220
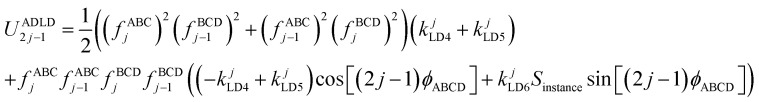


To ensure that the *k*^*j*^_LD4_ and *k*^*j*^_LD5_ terms never decrease in energy as the geometry undergoes an infinitesimal displacement away from the optimized ground-state geometry, combining eqn (219) and (220) shows the following constraints should be enforced during linear regression

221 *k*^*j*^_LD4_ ≥ 0

222 *k*^*j*^_LD5_ ≥ 0

Each sin[2*jϕ*_ABCD_] and sin[(2*j* − 1)*ϕ*_ABCD_] term decreases in energy as the dihedral undergoes an infinitesimal displacement away from the optimized ground-state geometry in one direction and increases in energy as the dihedral is displaced away from the optimized ground-state geometry in the opposite direction. Because the optimized ground-state geometry is an energy minimum, this means the sin[2*jϕ*_ABCD_] and sin[(2*j* − 1)*ϕ*_ABCD_] terms cannot appear by themselves in the optimized forcefield, but rather they can only occur if a force constant for one or more cos[2*jϕ*_ABCD_] or cos[(2*j* − 1)*ϕ*_ABCD_] modes is non-zero. In this case, the force constant(s) for the cosine mode(s) must be large enough to ensure that any active sine modes cannot lower the isolated bonded cluster's energy below that of its optimized ground-state geometry. Instead of enforcing this as an explicit constraint on the force constants, we rely on the training dataset fitting to obtain (asymptotically close to) this behavior during linear regression that optimizes the values of force constants.

Putting this altogether gives223
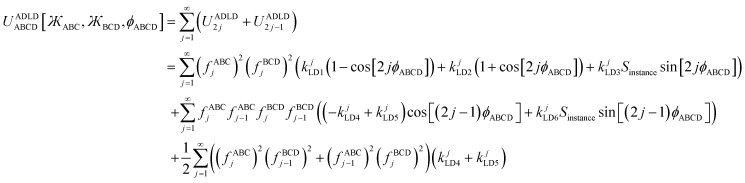
subject to the constraints

224 *k*^*j*^_LD1_,*k*^*j*^_LD2_,*k*^*j*^_LD4_,*k*^*j*^_LD5_ ≥ 0

This can also be re-written in the equivalent form225
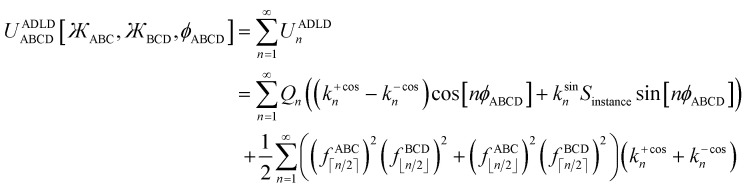
subject to the constraints

226 *k*^+cos^_*n*_,*k*^−cos^_*n*_ ≥ 0

### 9.2 Selecting specific modes from the linear dihedral torsion potential

A particular linear dihedral normally involves only a small subset of the terms shown in eqn (223). The particular terms involved depend on the type of linear dihedral. A single-linear dihedral contains exactly one linear (or close to linear) equilibrium bond angle: (π − *θ*^eq^_ABC_ < *ε*) xor (π − *θ*^eq^_BCD_ < *ε*). A double-linear dihedral contains two linear (or close to linear) equilibrium bond angles: (π − *θ*^eq^_ABC_ < *ε*) and (π − *θ*^eq^_BCD_ < *ε*). Here, we consider four cases.

*Case # 1 is a symmetry-induced single-linear dihedral.* Case # 1 corresponds to the situation in which the linear equilibrium bond angle is a ω-fold axis of rotational symmetry for some whole number 1 < *ω* < ∞. This symmetry eliminates all of the terms in eqn (223) except those for which *n* is wholly divisible by *ω*. For example, if *ω* = 2 then only the *n* = even (*i.e.*, the *n* = 2*j* terms) survive and all of the odd terms (*i.e.*, the *n* = (2*j* − 1) terms) are eliminated. If *n* = 3, then only the terms for which *n* (which is 2*j* or (2*j* − 1)) is wholly divisible by 3 (*i.e.*, *n* = 3, 6, 9, …) survive. Depending on the situation, the bent equilibrium bond angle may be contained in a mirror plane (Case 1a) or not (Case 1b). If the bent equilibrium bond angle is contained within a mirror plane (Case 1a), then this mirror symmetry eliminates all of the odd functions (*i.e.*, the sine terms) in eqn (223). Examples of Case 1a include boranecarbonitrile (H_2_B–C≡N) and isocyanoborane (H_2_B–N≡C) which have a 2-fold axis of rotational symmetry and acetonitrile (H_3_C–C≡N) which has a 3-fold axis of rotational symmetry. (Since *n* = 1 is not wholly divisible by any number *ω* > 1, it follows that the *n* = (2(*j* = 1) − 1) = 1 terms are always eliminated in Cases 1a and 1b.)

*Case # 2 is a symmetry-induced double-linear dihedral.* Case # 2 corresponds to the situation in which the linear ABCD equilibrium axis (along which the two linear equilibrium bond angles ABC and BCD reside) is either contained within two mirror planes (Case 2a) or a ω-fold axis of rotational symmetry (Case 2b). Either of these two symmetries (*i.e.*, Case 2a or Case 2b) ensures that the atom-in-material forces are zero on atoms A, B, C, and D when they are arranged along this linear axis. Case 2a: If two mirror planes contain the linear ABCD equilibrium axis, then this mirror symmetry eliminates all of the odd functions (*i.e.*, the sine terms) in eqn (223). Examples of this case include the acetylene (H–C≡C–H) and fulminic acid (H–C≡N–O) molecules. In this case, the torsion potential expands as227
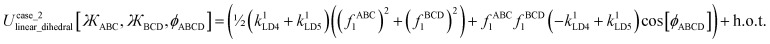
where h.o.t. stands for ‘higher-order terms’. In practice, only the leading-order terms explicitly written in eqn (227), which are the *n* = (2(*j* = 1) − 1) = 1 terms, are needed if the non-equilibrium bond angle fluctuations are modest in amplitude. This simplification arises because for small 
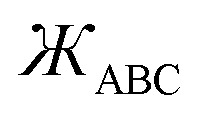
 the angle-damping factor *f*^ABC^_*j*_ is proportional to 
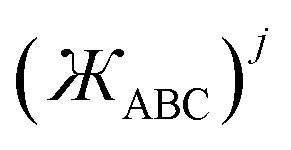
. In Case 2b, none of the terms in eqn (223) are necessarily automatically eliminated by symmetry; however, depending on the circumstances some of the terms in eqn (223) may turn out not to be significant. The C–C–N–O double-linear dihedral in the acetonitrile N-oxide (H_3_C–C≡N–O) molecule is an example of Case 2b.

*Case # 3 is an accidental single-linear dihedral.* In this context, the term ‘accidental’ means that the single-linear dihedral arises due to a close to linear equilibrium bond angle that is not caused by any intrinsic symmetry. Depending on one's perspective, this case could be regarded as either ‘rare’ or ‘not rare’. Specifically, when examining a single material, it is comparatively ‘rare’ that the balancing of forces would fortuitously lead to an after-dihedral-pruning close to linear equilibrium bond angle not caused by any intrinsic symmetry. (Before dihedral pruning, close to linear equilibrium bond angles would occur frequently. The vast majority of these would be removed from the active dihedrals list by dihedral pruning.) However, when examining a large database of materials, the sheer large number of materials investigated makes it probable that a few of these materials will contain an accidental single-linear dihedral after dihedral pruning. Since the force for small (*i.e.*, infinitesimal) displacements of the linear bond angle must be zero, it follows (see ESI† Section S8) that the *n* = (2(*j* = 1) − 1) = 1 terms are eliminated in eqn (223), while higher-order terms can potentially contribute to the torsion potential.

*Case # 4 is an accidental double-linear dihedral.* This case corresponds to the situation in which a double-linear dihedral arises due to a balance of forces that causes a linear ABCD moiety not caused by intrinsic symmetry. In this case, none of the terms in eqn (223) are necessarily automatically eliminated by symmetry; however, depending on the circumstances some of the terms in eqn (223) may turn out not to be significant. If considering relatively local symmetries of the chemical group rather than global symmetries of the entire chemical system, this case would be rare after dihedral pruning.

Depending on one's perspective, an acetylene molecule adsorbed in a MOF could be considered either as Case #2 or Case #4 if it has a double-linear dihedral. If one considers an acetylene molecule's symmetry without including the MOF adsorbent, this double-linear dihedral would be classified as Case #2. If one considers the global symmetry of the MOF plus adsorbed acetylene molecule, the lower symmetry of this combined system could result in classifying this double-linear dihedral as Case #4. Ultimately, this demonstrates that the classification into various cases is a judgement call that depends on how large a chemical subunit is chosen when evaluating the symmetry properties. A reasonable compromise would be to consider a local region that extends ∼3 bonds in all directions around the edges of the dihedral being considered.

## 10. Results and discussion

### 10.1 CCSD quantum chemistry calculations of small molecules

All quantum chemistry calculations in this section were performed in Gaussian16 (ref. 33) using the CCSD method with def2-TZVPD^34^ basis sets. (In this article, CCSD not CCSD(T) calculations were used.) For molecules containing no elements heavier than neon, all electrons were correlated in the coupled-cluster calculation. For molecules containing one or more elements heavier than neon, the FreezeNobleGasCore keyword was used, which applies the coupled-cluster correlation to the valence shell electrons only on all atoms. Geometries were optimized to the following convergence criteria: (1) the maximum force is less than 0.00045 hartrees/bohr; (2) the root-mean squared (RMS) force is less than 0.0003 hartrees/bohr; (3) the maximum displacement is less than 0.0018 bohr; and (4) the RMS displacement is less than 0.0012 bohr. As described below, some of the calculations constrained one or more internal coordinates to generate energy scans.

**Fig. 7:**
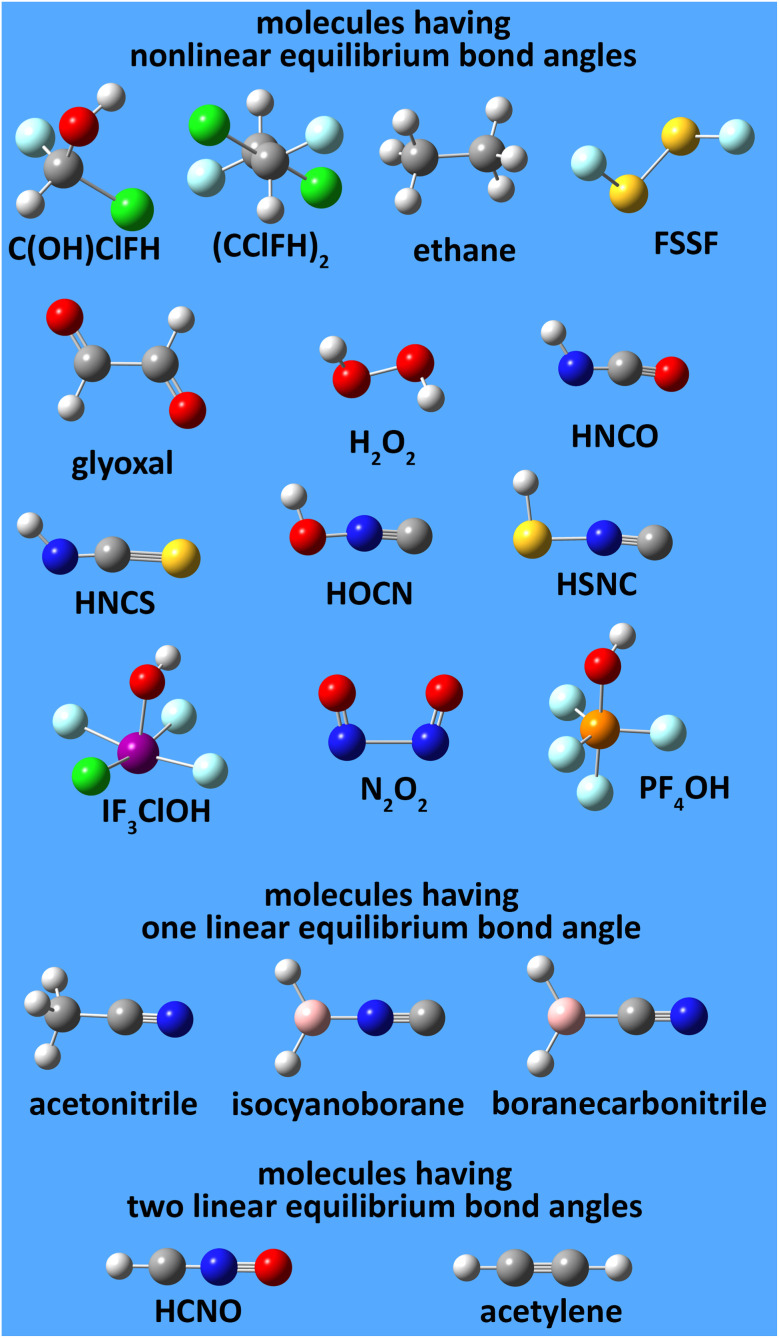
Optimized geometries of molecules used to study dihedral torsion.

Values of calculated geometric parameters in the CCSD/def2-TZVPD optimized ground-state structures of 13 molecules containing no linear bond angles are shown in Table 1 and of five molecules containing one or more linear bond angles are shown in Table 2. For each molecule, optimized bond lengths and angles are listed for the particular dihedral that was subsequently studied in detail as described below. Fig. 7 shows the optimized geometries.

**Table 1:** Key values of calculated geometric parameters in the CCSD/def2-TZVPD optimized ground-state structure of each molecule. These dihedrals contain two nonlinear equilibrium bond angles

Molecule	Atoms in dihedral	*ϕ*_eq_ (°)	Bond 1 (Å)	Bond 2 (Å)	Bond 3 (Å)	Angle 1 (°)	Angle 2 (°)
(CClFH)_2_	HCCH	180.0	1.090 (HC)	1.524 (CC)	1.090 (CH)	111.2 (HCC)	111.2 (CCH)
C(OH)ClFH	FCOH	−64.70	1.358 (FC)	1.355 (CO)	0.967 (OH)	111.1 (FCO)	109.0 (COH)
Ethane	HCCH	180.0	1.093 (HC)	1.525 (CC)	1.093 (CH)	111.3 (HCC)	111.3 (CCH)
FSSF	FSSF	87.42	1.625 (FS)	1.913 (SS)	1.625 (SF)	107.2 (FSS)	107.2 (SSF)
Glyoxal	OCCO	180.0	1.201 (OC)	1.520 (CC)	1.201 (CO)	121.3 (OCC)	121.3 (CCO)
H_2_O_2_	HOOH	111.1	0.967 (HO)	1.438 (OO)	0.967 (OH)	100.8 (HOO)	100.8 (OOH)
HNCO	HNCO	180.0	1.006 (HN)	1.211 (NC)	1.160 (CO)	123.6 (HNC)	173.0 (NCO)
HNCS	HNCS	180.0	1.005 (HN)	1.199 (NC)	1.572 (CS)	131.7 (HNC)	174.3 (NCS)
HONC	HONC	180.0	0.969 (HO)	1.327 (ON)	1.168 (NC)	105.1 (HON)	173.3 (ONC)
HSNC	HSNC	180.0	1.343 (HS)	1.665 (SN)	1.175 (NC)	95.4 (HSN)	173.8 (SNC)
IF_3_ClOH	ClIOH	180.0	2.343 (ClI)	1.861 (IO)	0.972 (OH)	83.5 (ClIO)	107.1 (IOH)
N_2_O_2_	ONNO	0.00	1.151 (ON)	1.872 (NN)	1.151 (NO)	103.4 (ONN)	103.4 (NNO)
PF_4_OH	FPOH	180.0	1.543 (FP)	1.617 (PO)	0.963 (OH)	88.0 (FPO)	113.1 (POH)

**Table 2:** Key values of calculated geometric parameters in the CCSD/def2-TZVPD optimized ground-state structure of each molecule. These dihedrals contain one or two linear equilibrium bond angles

Molecule	Atoms in dihedral	Bond 1 (Å)	Bond 2 (Å)	Bond 3 (Å)	Angle 1 (°)	Angle 2 (°)
Acetonitrile	HCCN	1.090 (HC)	1.462 (CC)	1.152 (CN)	109.9 (HCC)	180.0 (CCN)
Acetylene	HCCH	1.064 (HC)	1.202 (CC)	1.064 (CH)	180.0 (HCC)	180.0 (CCH)
H_2_BCN	HBCN	1.185 (HB)	1.533 (BC)	1.156 (CN)	118.8 (HBC)	180.0 (BCN)
H_2_BNC	HBNC	1.186 (HB)	1.432 (BN)	1.175 (NC)	118.3 (HBN)	180.0 (BNC)
HCNO	HCNO	1.062 (HC)	1.152 (CN)	1.202 (NO)	180.0 (HCN)	180.0 (CNO)

For the ONNO molecule, CCSD/def2-TZVPD geometry optimization was performed for both the *cis* and *trans* conformations in both the singlet and triplet spin states. The computed relative energies (in eV) were 0.00 (*cis*, singlet), 0.11 (*trans*, singlet), 0.48 (*cis*, triplet) and 0.53 (*trans*, triplet). East previously studied the electronic states of this molecule in cis and trans geometries with complete active space self-consistent field (CAS-SCF), multireference configuration (MRCISD), and other electronic structure methods.^35^ Those computations indicated the cis geometry with singlet spin state to be the ONNO molecule's ground state.^35^

### 10.2 Detailed torsion analysis for rotatable dihedrals using ADDT and CADT model potentials

Table 3 summarizes torsion mode analysis for these 13 molecules containing nonlinear dihedrals. Both rigid and (fully or partly) relaxed torsion scans are presented. For HNCO, HNCS, HONC, and HSNC, fully relaxed torsion scans could not be computed, because due to ‘slip torsion’ (see Section 10.4) some constrained dihedral values yielded a relaxed bond angle that was too close to linear for the optimizer to converge. (The dihedral value becomes indeterminate when the bond angle becomes linear.) For each of these four molecules, a partly relaxed torsion scan was thus performed holding the two bond angles rigid but allowing the bond lengths to relax as the constrained dihedral was scanned. As shown in Table 3, these partly relaxed torsion scans yielded results virtually equal to the rigid torsion scans. Fully relaxed dihedral scans were performed for the other nine molecules. In Table 3, the columns labeled ‘mode 1’, ‘mode 2’, *etc.* display the coefficients (*i.e.*, *c*_*i*_ values) for these individual modes. As demonstrated by the SumCSq ≈ 1.00 values in Table 3, my new seven-mode dihedral torsion model potential yielded superb fits for all of these molecules.

**Table 3:** Torsion mode analysis using the DT projectors for 13 molecules at the CCSD/def2-TZVPD level of theory. For the results without parentheses, all bond angles and bond lengths were held fixed at values taken from the ground-state structure, and the dihedral value was rigidly scanned to generate the energy curves. Where available, results shown inside parentheses allowed the bond angles and bond lengths to relax as the constrained dihedral value was scanned to generate the energy curves. For HNCO, HNCS, HONC, and HSNC, results in square brackets allowed the bond lengths to relax while the bond angles were held rigid

Molecule	Atoms in dihedral	*ϕ*_eq_ (°)	QM torsion barrier (kJ mol^−1^)	QM torsion norm (kJ mol^−1^)	*c*_1_	*c*_2_	*c*_3_	*c*_4_	*c*_5_	*c*_6_	*c*_7_	SumCSq
(CClFH)_2_	HCCH	180.0 (180.0)	73.15 (39.98)	19.40 (10.42)	0.5943 (0.5058)	−0.3795 (−0.2707)	0.7048 (0.8123)	−0.0690 (−0.0807)	0.0000 (0.0000)	0.0000 (0.0000)	0.0003 (0.0002)	0.9987 (0.9955)
C(OH)ClFH	FCOH	−64.70 (−64.70)	19.39 (15.78)	6.55 (4.72)	0.8513 (0.8028)	0.4885 (0.4386)	0.1801 (0.3742)	−0.0096 (−0.0092)	0.0197 (0.0899)	0.0400 (0.0856)	−0.0470 (−0.0863)	1.0000 (0.9999)
Ethane	HCCH	180.0 (180.0)	12.65 (12.10)	4.47 (4.28)	0.0000 (0.0000)	0.0000 (0.0000)	1.0000 (0.9996)	0.0000 (0.0000)	0.0000 (0.0000)	0.0000 (0.0000)	−0.0001 (−0.0001)	1.0000 (0.9992)
FSSF	FSSF	87.42 (87.42)	212.0 (129.6)	69.23 (43.80)	0.0041 (−0.0005)	0.9731 (0.9902)	0.0014 (0.0136)	−0.1768 (0.0035)	0.0829 (−0.0414)	−0.0932 (−0.0798)	0.0372 (0.0984)	0.9951 (0.9986)
Glyoxal	OCCO	180.0 (180.0)	25.41 (24.86)	8.48 (8.52)	0.8090 (0.8215)	0.5842 (0.5658)	0.0599 (0.0033)	−0.0255 (−0.0694)	0.0000 (0.0000)	0.0000 (0.0000)	0.0000 (0.0000)	0.9999 (0.9999)
H_2_O_2_	HOOH	111.1 (111.1)	35.75 (31.56)	11.99 (10.68)	0.2996 (0.3014)	0.4077 (0.4028)	−0.0446 (−0.0322)	−0.0009 (0.0000)	−0.7454 (−0.7479)	0.3338 (0.3257)	−0.2738 (−0.2835)	1.0000 (1.0000)
HNCO	HNCO	180.0 [180.0]	14.86 [14.78]	5.25 [5.23]	1.0000 [1.0000]	−0.0011 [0.0004]	0.0004 [0.0003]	0.0000 [0.0000]	0.0000 [0.0000]	0.0000 [0.0000]	0.0000 [0.0000]	1.0000 [1.0000]
HNCS	HNCS	180.0 [180.0]	8.19 [8.15]	2.90 [2.88]	1.0000 [1.0000]	−0.0043 [-0.0030]	0.0002 [0.0002]	0.0000 [0.0000]	0.0000 [0.0000]	0.0000 [0.0000]	0.0000 [0.0000]	1.0000 [1.0000]
HONC	HONC	180.0 [180.0]	2.54 [2.54]	0.903 [0.902]	0.9950 [0.9950]	0.1001 [0.1003]	0.0001 [0.0002]	0.0000 [0.0000]	0.0000 [0.0000]	0.0000 [0.0000]	0.0000 [0.0000]	1.0000 [1.0000]
HSNC	HSNC	180.0 [180.0]	1.89 [1.89]	0.675 [0.673]	0.9914 [0.9914]	0.1307 [0.1310]	0.0000 [0.0002]	0.0000 [0.0001]	0.0000 [0.0000]	0.0000 [0.0000]	0.0000 [0.0000]	1.0000 [1.0000]
IF_3_ClOH	ClIOH	180.0 (180.0)	8.95 (2.70)	2.91 (0.70)	0.7753 (0.4091)	0.6228 (0.2565)	0.0930 (0.2593)	0.0488 (0.8356)	0.0001 (0.0000)	0.0001 (−0.0001)	0.0000 (−0.0001)	0.9999 (0.9987)
N_2_O_2_	ONNO	0.00 (0.00)	26.13 (18.99)	8.40 (5.38)	0.7431 (0.3967)	0.5981 (0.8543)	0.2981 (0.3347)	−0.0254 (0.0272)	0.0000 (0.0000)	0.0000 (0.0000)	0.0000 (0.0000)	0.9994 (1.0000)
PF_4_OH	FPOH	180.0 (180.0)	10.20 (4.46)	3.34 (1.69)	0.0000 (0.0000)	0.0000 (0.0000)	0.9862 (0.9922)	0.0000 (0.0000)	0.0007 (0.0007)	0.0000 (0.0000)	−0.0018 (−0.0018)	0.9727 (0.9844)

**Fig. 8:**
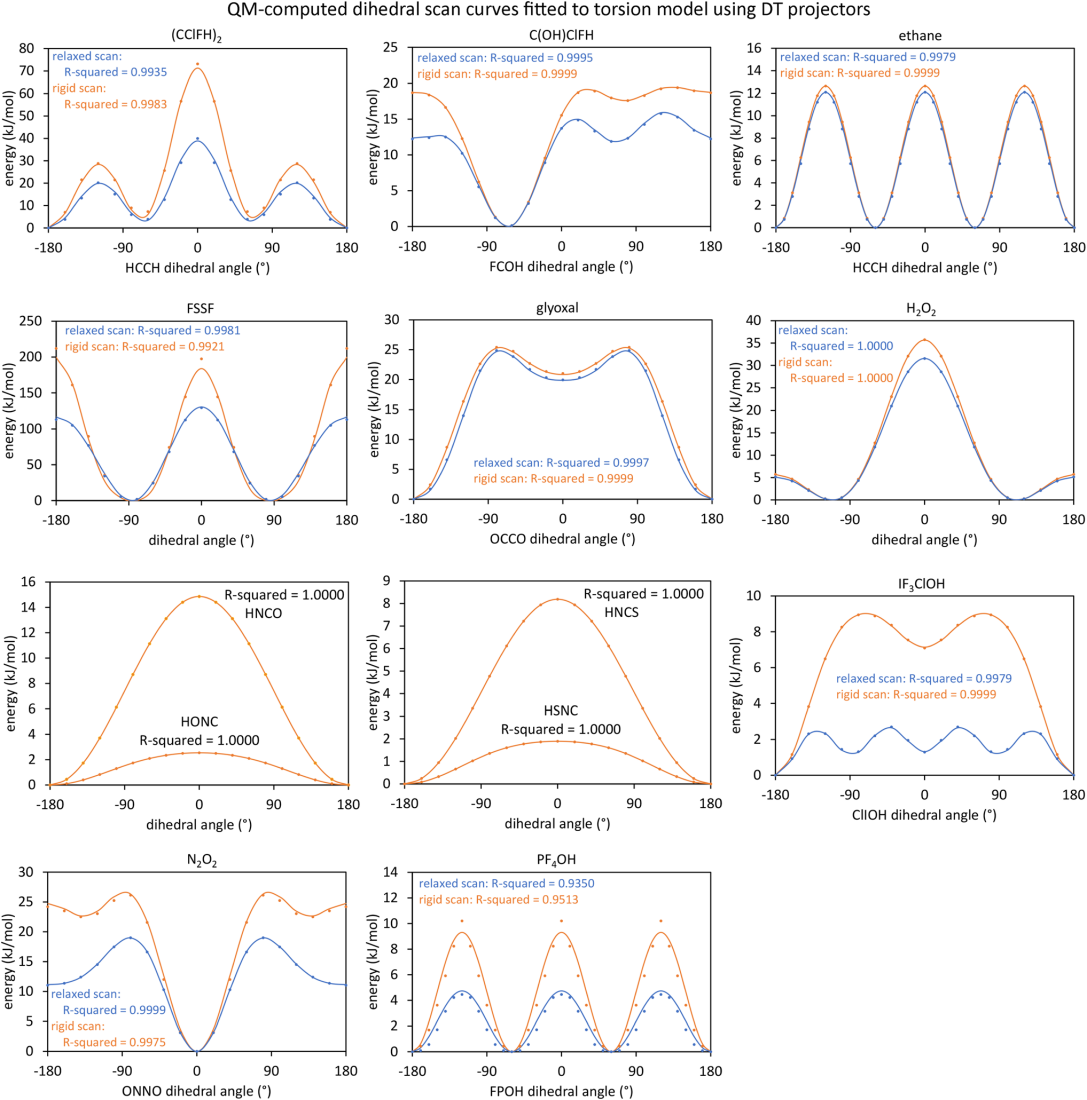
Example dihedral scan energy curves for several molecules. The *y*-axis plots the energy relative to the low energy conformation. The filled circles show the QM-computed (CCSD/def2-TZVPD) values. The solid lines show the fitted model potential of eqn (161) and (162) using the DT projectors (eqn (122) and (123)) with the parameters from Table 3. Bond angles and bond lengths were held fixed to generate the results shown in orange. Bond angles and bond lengths were relaxed to generate the results (where available) shown in blue. The SumCSq values are listed in Table 3.

Fig. 8 plots the quantum chemistry results and model fits (including all seven torsion modes) for the rigid and fully relaxed torsion scans. In some cases (*e.g.*, ethane, glyoxal, and H_2_O_2_), the relaxed torsion scan gave nearly the same results as the rigid torsion scan. In other cases (*e.g.*, (CClFH)_2_, IF_3_ClOH, N_2_O_2_, and PF_4_OH), there was a huge difference between the relaxed and rigid torsion scans. FSSF and C(OH)ClFH showed modest non-negligible differences between relaxed and rigid torsion scans.

**Fig. 9:**
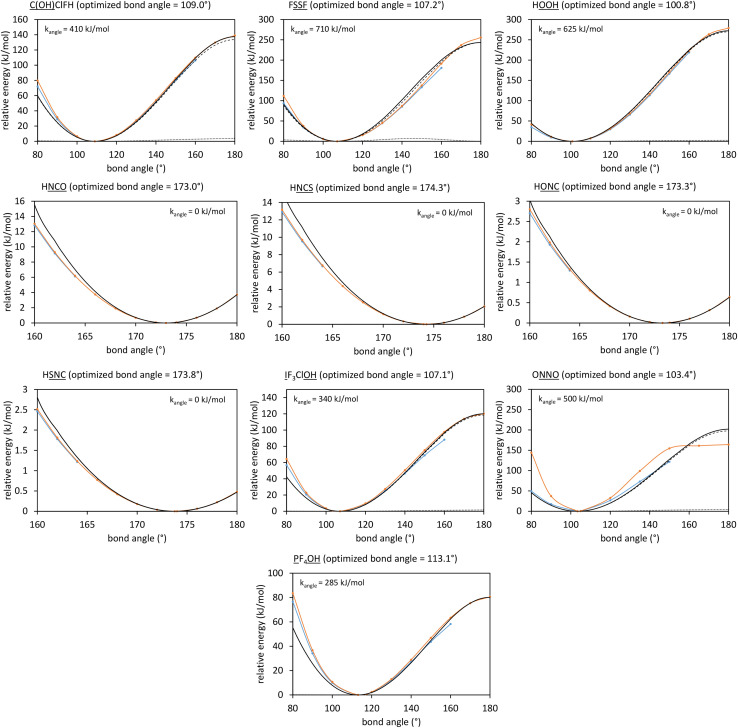
Angle-scan energy curves for several polyatomic molecules. The orange curves show the QM-computed (CCSD/def2-TZVPD) values holding the bond lengths (and other geometric parameters) fixed as the constrained angle varied, while the blue points (CCSD/def2-TZVPD) relaxed all geometric parameters except the constrained angle. (Blue points were not available in some instances due to the bond angle being too close to linear for either the relaxed torsion scan or the relaxed angle-bending energy to be computed.) The model potential (solid black line) is the sum of the angle-bending model potential (dashed black line) and the ADDT torsion offset potential (dotted black line). The angle-bending force constant is displayed treating radians as dimensionless units. The ADDT torsion offset potential was computed using the rigid torsion parameters from Table 3.

Angle-scan energy curves were QM-computed for the 10 molecules from this set that contained at least one atom with a coordination number equal to 2. Fig. 9 compares these QM-computed results to a model potential. The model potential was the sum of the ADDT torsion offset potential (computed using the rigid torsion parameters from Table 3) and the following angle-bending model potential^20^ with the k_angle_ value displayed on each graph:228
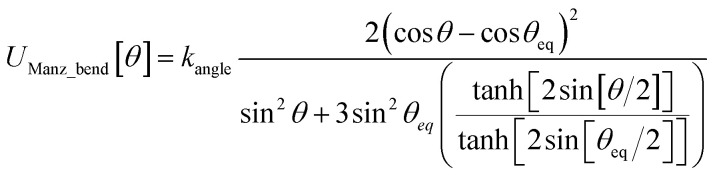
In this model potential, the torsion offset potential and angle-bending potential were only included for the one angle of interest and not for any other angles. Since the torsion parameters (listed in Table 3) were already computed from the torsion mode analysis, k_angle_ was the only parameter freely adjusted to generate the model curves displayed in Fig. 9. Agreement between the QM-computed results and the model potential was generally good; however, as the bond angle became acute (<90°) steric repulsion often caused the quantum-mechanically-computed results to rise higher in energy than the model potential. For 
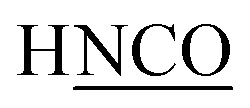
, 
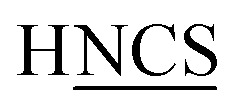
, 
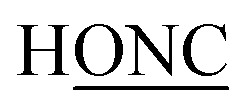
, and 
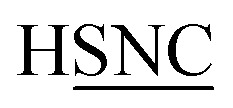
, the angle-scan energy curve was completely described by the ADDT torsion offset potential with negligible contribution (*i.e.*, *k*_angle_ = 0) from the angle-bending model potential. For 
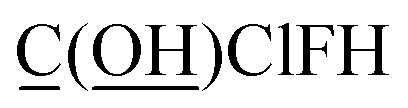
, 
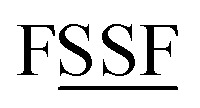
, 
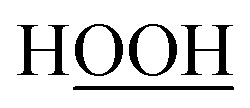
, 
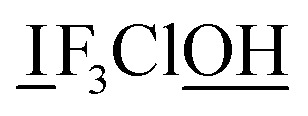
, 
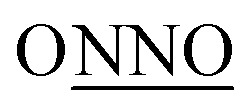
, and 
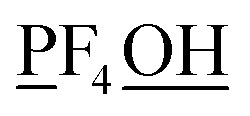
, the angle-scan energy curve was almost completely described by the angle-bending model potential with only a tiny contribution from the torsion offset potential. In summary, these results show the angle-bending model potential is typically much more significant than the torsion offset potential when the equilibrium bond angle is highly bent (*i.e.*, (π − *θ*_eq_) is large).

The torsion offset potential occurs for the cosine modes (*e.g.*, modes 1 to 4) of the ADDT model potential. There is no torsion offset potential for the sine modes of the ADDT and CADT model potentials. The torsion offset potential is also zero for the CADT cosine modes, because TOP_*n*_ = *J*_*n*_ − *H*_*n*_ (see eqn (106)) and *J*_*n*_ = *H*_*n*_ = 1 (see eqn (136)) for the CADT model potential.

An angle-bending model potential must have a slope of zero at *θ* = π in order for its derivative (and hence force) to be continuous at *θ* = π. As explained in prior literature, this restriction arises from the reflection symmetry of cos[*θ*] about *θ* = π.^10,20^ In contrast to the bond angle, the directed dihedral is not required to have reflection symmetry. An angle-scan energy curve for a subgroup of 3 atoms within a larger bonded cluster is not required to have a slope of zero at *θ* = π for its derivative (and hence force) to be continuous at *θ* = π, because this energy curve is the sum of an angle-bending potential and a torsion offset potential. Accordingly, any non-zero slope of the angle-scan energy curve at *θ* = π is assigned to the torsion offset potential and not to the angle-bending model potential. In the examples studied here, the QM-computed angle-scan energy curves have highly non-zero slopes at *θ* = π for 
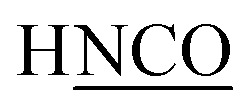
, 
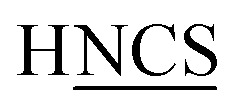
, 
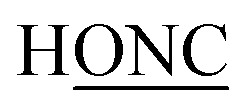
, and 
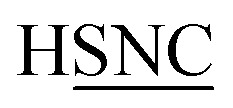
. As shown in Fig. 9, these nonzero slopes were practically perfectly described by the torsion offset potentials. Remarkably, this practically perfect agreement in slopes at *θ* = π occurred without using any freely adjustable parameters, because the torsion parameters had already been obtained from the torsion mode analysis as listed in Table 3.

Why is it useful to compute both rigid and relaxed angle-bending and dihedral–torsion scans? Comparing the rigid scan energy curve to the relaxed scan energy curve provides extremely valuable insights into the relative importance of some cross terms. First, consider the angle-scan curves shown in Fig. 9. Iff the relaxed scan curve is greatly below the rigid scan curve, then this indicates that changing bond lengths (or other geometric parameters) substantially lowers the energy at non-equilibrium angle values, and in this case bond-bend (or other) cross terms may be needed to construct an accurate forcefield. Iff the relaxed and rigid angle-scan curves are nearly identical, this suggests bond-bend cross terms are not required to construct an accurate forcefield model. Second, consider the dihedral-scan energy curves shown in Fig. 8. Iff the rigid and relaxed torsion scans have nearly identical energy profiles, then this suggests that bond-torsion and bend-torsion cross terms are not required to construct an accurate forcefield model. Iff the relaxed torsion scan curve is greatly below the rigid torsion scan curve, then this indicates that changing bond lengths, angle values, or other geometric parameters substantially lowers the energy at non-equilibrium dihedral values, and in this case bond-torsion, bend-torsion, and/or torsion–torsion cross terms may be needed to construct an accurate forcefield.

For purposes of optimizing flexibility model parameter values, one can choose to use either rigid torsion scans or relaxed torsion scans in the training dataset for rotatable dihedrals. Some quantum-chemistry software packages do not have built-in constrained geometry optimization algorithms that facilitate holding one dihedral's value constant while relaxing all other internal coordinates. For practical reasons, relaxed torsion scans would be extremely difficult to perform using those software packages; consequently, rigid torsion scans would be preferred in those cases. Because rigid torsion scans do not require constrained geometry optimization, they are computationally cheaper and easier to implement than relaxed torsion scans. For slip torsions, either rigid or ‘partly relaxed’ (*i.e.*, with at least one bond angle's value constrained) scans are required, because this allows sampling the full range of dihedral values. On the other hand, relaxed torsion scans may be preferred in some cases when constructing the training dataset for rotatable dihedrals, because relaxed torsion scans can provide a more accurate and realistic estimate of the torsion energy barrier than rigid torsion scans.

How accurate is the ADDT model potential for predicting changes in the torsion norm due to non-equilibrium bond angle values? Two datasets were studied to explore this question. The first dataset contained dihedrals for which at least one of the contained equilibrium bond angles was ≥130°. The second dataset contained dihedrals for which both contained equilibrium bond angles were <130°. To generate these two datasets, one bond angle was constrained while the corresponding dihedral was scanned and all other geometric parameters were relaxed. These constrained CCSD/def2-TZVPD calculations were performed in Gaussian 16 using the opt = modredundant method.

For the first dataset, the bond angle chosen for study had an equilibrium value ≥130° in the optimized ground-state structure. Dihedral scans were attempted for 
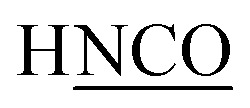
, 
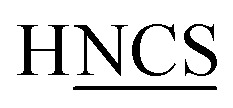
, 
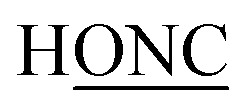
, and 
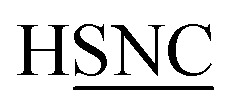
 at constrained values of 125°, 140°, 155°, and 165° for the underlined bond angle. In some of these calculations, the other (*i.e.*, unconstrained) bond angle became too close to linear for the default geometry optimizer in Gaussian16 (ref. 33) to converge.

**Fig. 10:**
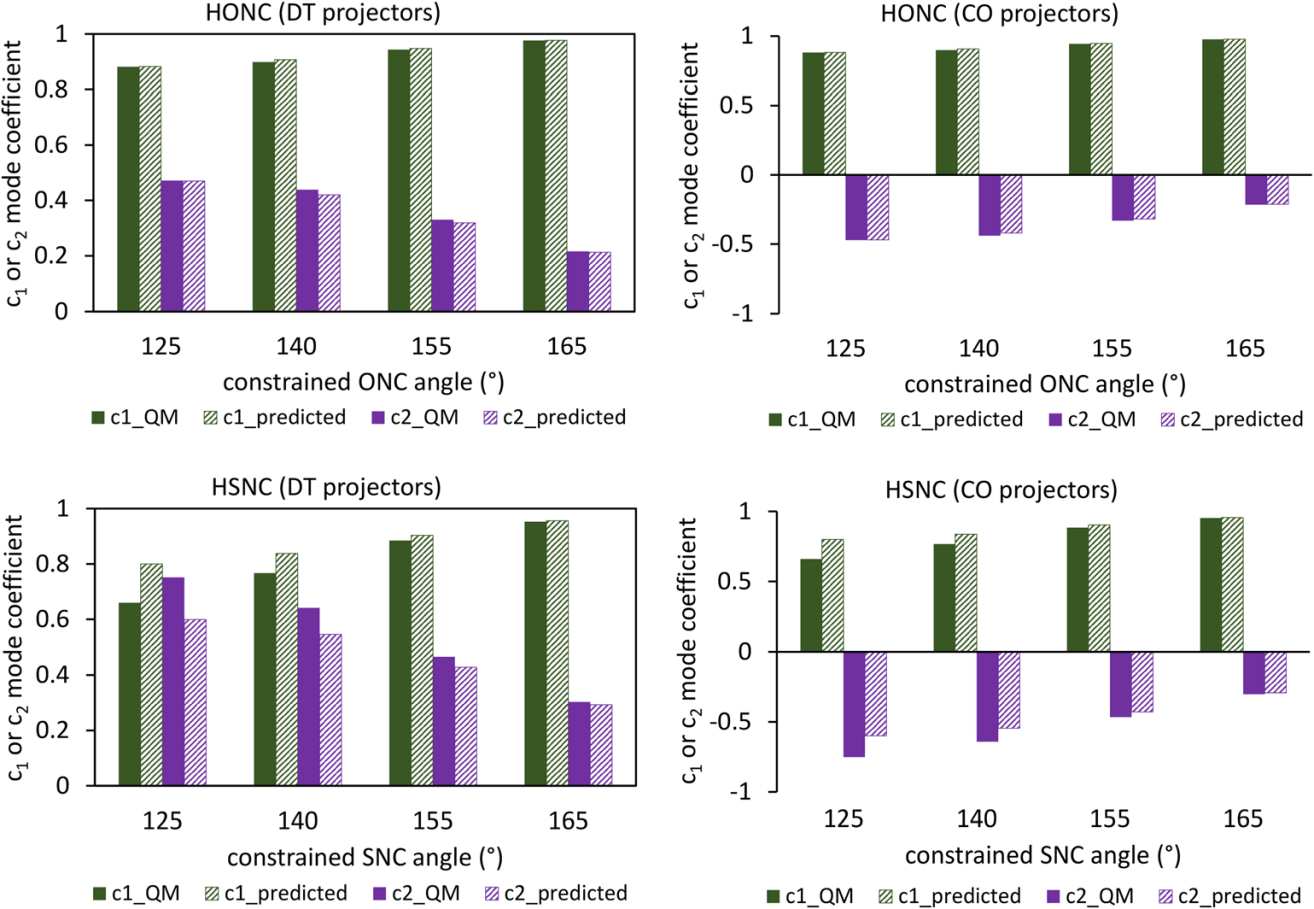
Comparison of *c*_1_ and *c*_2_ mode coefficients for HONC (top panels) and HSNC (bottom panels) molecules at different constrained values of the ONC or SNC bond angle. Solid bars show the QM-extracted values using the DT projectors (left panels) and CO projectors (right panels). Diagonally-hashed bars show the values predicted using the ADDT model (left panels) and ADCO model (right panels).

The ADDT model predictions shown in Tables 4, 6 and Fig. 10 were made using the following steps:

**Table 4:** Comparison of QM-computed (CCSD/def2-TZVPD) and ADDT model-predicted torsion norms for several molecules having at least one included equilibrium bond angle ≥ 130°. For each geometry, one bond angle and one dihedral were constrained with the other geometric parameters relaxed. The dihedral was constrained to a series of different values to generate the torsion curve at the constrained bond angle. *ϕ*_min_ is the dihedral value that has the lowest energy. The column labeled ‘QM norm ratio’ shows the ratio of the QM torsion norm for the constrained bond angle to the partly relaxed torsion norm displayed in Table 3 for the optimized bond angle (the ‘optimized norm’). ADDT and CADT model predictions are listed in the last four columns

Molecule	*θ*_constr_ (°)	*θ*_other_ (°) at *ϕ*_min_	*ϕ*_min_ (°)	QM torsion norm (kJ mol^−1^)	QM norm ratio	ADDT predicted *ϕ*_min_ (°)	ADDT predicted norm (kJ mol^−1^)	ADDT predicted norm ratio	CADT prediction equal to optimized norm (kJ mol^−1^)
HNCO	125.0 (NCO)	120.0 (HNC)	180.0	37.84	7.24	180.0	63.59	12.17	5.23
HNCO	140.0 (NCO)	122.2 (HNC)	180.0	28.99	5.55	180.0	38.98	7.46	5.23
HNCO	155.0 (NCO)	123.4 (HNC)	180.0	18.46	3.53	180.0	20.77	3.98	5.23
HNCO	165.0 (NCO)	123.9 (HNC)	180.0	11.13	2.13	180.0	11.47	2.20	5.23
HNCS	165.0 (NCS)	130.5 (HNC)	180.0	7.49	2.60	180.0	8.11	2.81	2.88
HONC	125.0 (ONC)	104.4 (HON)	180.0	8.10	8.98	180.0	12.04	13.35	0.90
HONC	140.0 (ONC)	105.1 (HON)	180.0	6.08	6.74	180.0	7.47	8.29	0.90
HONC	155.0 (ONC)	105.3 (HON)	180.0	3.58	3.97	180.0	3.91	4.33	0.90
HONC	165.0 (ONC)	105.2 (HON)	180.0	2.06	2.28	180.0	2.12	2.35	0.90
HSNC	125.0 (SNC)	95.7 (HSN)	180.0	5.46	8.12	180.0	10.48	15.58	0.67
HSNC	140.0 (SNC)	96.0 (HSN)	180.0	4.65	6.91	180.0	6.44	9.57	0.67
HSNC	155.0 (SNC)	95.8 (HSN)	180.0	2.86	4.25	180.0	3.27	4.87	0.67
HSNC	165.0 (SNC)	95.6 (HSN)	180.0	1.65	2.46	180.0	1.74	2.58	0.67

(1) (Partly or fully) relaxed torsion mode analysis was performed as shown in Table 3. This yielded the ‘optimized’ torsion norm and the ‘optimized’ *c*_1_ to *c*_7_ for each dihedral studied.

(2) CCSD/def2-TZVPD energies were computed at each constrained bond angle value for a series of uniformly spaced dihedral values over the range −π < *ϕ* ≤ π. In each of these calculations, one bond angle and one dihedral were held rigid while all other geometric parameters were relaxed. In some cases, molecular symmetry could be exploited. Taking HSNC as an example, QM calculations were performed for HSNC dihedral values of 0°, 20°, 40°, 60°, 80°, 100°, 120°, 140°, 160°, and 180° for each constrained SNC angle value of 125°, 140°, 155°, and 165°. The HSNC molecular symmetry shows the energy at a HSNC dihedral value of *ϕ* = *γ* equals that at a value of *ϕ* = −*γ*.

(3) For each constrained bond angle value, *ϕ*_min_ was identified as the *ϕ* value having the lowest QM-computed energy. In some cases, this occurred at a symmetry plane such as *ϕ* = 180° (for HSNC) or 0° (for N_2_O_2_). Otherwise, *ϕ*_min_ was computed *via* a CCSD/def2-TZVPD calculation that held one bond angle rigid (at the constrained value) while all other geometric parameters were relaxed.

(4) Using results from (2), torsion mode analysis was then performed at each of the constrained bond angle values. This yielded the QM torsion norm and QM torsion mode coefficients for each constrained bond angle value.

(5) The ADDT model predictions were made as follows. Starting with the optimized torsion norm and coefficients as computed in 1), the potential was then rewritten as:229
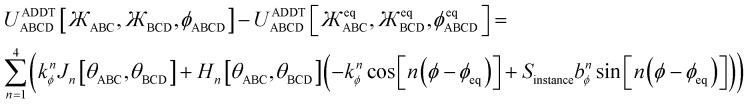
The *f*^ABC^_*n*_, *f*^BCD^_*n*_, *f*^ABC^_*n*_eq_, and *f*^BCD^_*n*_eq_ values were computed using the *θ*_constr_, *θ*_other_, angle_1, and angle_2 values listed in Tables 1 and 4 or 6. The predicted norm and mode coefficients were then computed as230
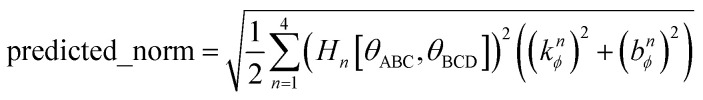
231
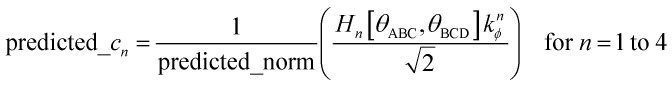
The factors of 
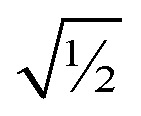
 appear in eqn (230) and (231), because the root-mean-squared values of cosine and sine functions is 
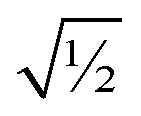
. *Caution:* The relationship between the predicted torsion norm and the torsion force constants as shown in eqn (230) and (231) holds only when the relevant internal coordinates are non-redundant and there are no multicollinearity issues of the related flexibility terms. This condition was clearly met for the dihedral torsions studied here.

(6) For each value of the constrained bond angle, the ADDT predicted *ϕ*_min_ value was computed by calculating the full 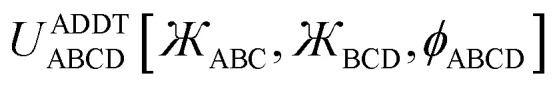 curve over the range −180° < *ϕ*_ABCD_ ≤ 180° in 0.01° increments and identifying which value of *ϕ*_ABCD_ produced the minimum value of 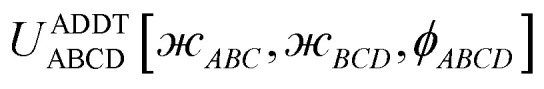.

For the first dataset, QM torsion norms for all of the converged calculations are listed in Table 4 and compared to predictions of the ADDT and CADT models. Here, the CADT model approximated the torsion norm as equal to the partly relaxed torsion norm listed in Table 3. As shown in Table 4, for these systems the QM torsion norm systematically increased as the constrained bond angle decreased in value. My ADDT model reproduced this trend, while the CADT model did not.

Table 5 summarizes the torsion mode coefficients extracted from these QM results. For HNCO and HNCS, only *c*_1_ was larger in absolute value than 0.1. For HONC and HSNC, both *c*_1_ and *c*_2_ were larger in absolute value than 0.1, while *c*_3_ to *c*_7_ were smaller than this. For HONC and HSNC, *c*_2_ systematically increased in magnitude as the constrained bond angle decreased. As shown in Fig. 10, my ADDT model predicted this trend with good quantitative accuracy.

**Table 5:** QM-computed torsion mode coefficients using the DT projectors for the same systems and constrained bond angles as analyzed in Table 4

Molecule	*θ*_constr_ (°)	*c*_1_	*c*_2_	*c*_3_	*c*_4_	*c*_5_, *c*_6_, *c*_7_	SumCSq
HNCO	125.0	0.9994	0.0143	0.0308	−0.0011	0.0000	1.0000
HNCO	140.0	0.9999	0.0070	0.0119	−0.0012	0.0000	1.0000
HNCO	155.0	1.0000	0.0035	0.0035	0.0000	0.0000	1.0000
HNCO	165.0	1.0000	0.0019	0.0012	0.0001	0.0000	1.0000
HNCS	165.0	1.0000	−0.0040	−0.0004	0.0005	0.0000	1.0000
HONC	125.0	0.8816	0.4718	0.0089	0.0022	0.0000	1.0000
HONC	140.0	0.8985	0.4389	0.0072	0.0003	0.0000	1.0000
HONC	155.0	0.9438	0.3304	0.0026	0.0003	0.0000	1.0000
HONC	165.0	0.9764	0.2160	0.0010	0.0002	0.0000	1.0000
HSNC	125.0	0.6597	0.7514	−0.0107	0.0059	0.0000	1.0000
HSNC	140.0	0.7672	0.6414	0.0039	0.0013	0.0000	1.0000
HSNC	155.0	0.8847	0.4661	0.0031	0.0001	0.0000	1.0000
HSNC	165.0	0.9530	0.3029	0.0014	0.0000	0.0000	1.0000

The second dataset contained six molecules. In each molecule, the selected dihedral had both included equilibrium bond angles <130° in its optimized ground-state structure. For the same three bonded atoms as studied in Fig. 9, torsion mode analysis was performed at constrained bond angle values 10° larger and 10° smaller than the equilibrium value. As shown in Fig. 9, the energy penalty for changing the bond angle is much larger for the bond angles studied in dataset 2 compared to those in dataset 1. In other words, the bond angles in dataset 2 were much stiffer than those in dataset 1. Accordingly, during a molecular dynamics or Monte Carlo simulation, the bond angles in dataset 2 would be expected to change less compared to those in dataset 1. This explains why Table 6 for dataset 2 studied smaller changes in the constrained bond angle compared to Table 4 for dataset 1. As shown in Table 6, the QM torsion norm increased more often (∼2/3 of the time) than decreased (∼1/3 of the time) as the constrained bond angle decreased. The ADDT model predicted a systematic increase in the torsion norm as the constrained bond angle decreased, while the CADT model predicted unchanged amplitudes.

**Table 6:** Comparison of QM-computed (CCSD/def2-TZVPD) and ADDT model-predicted torsion norms for several molecules having both included equilibrium bond angles < 130°. For each geometry, one bond angle and one dihedral were constrained with the other geometric parameters relaxed. The dihedral was constrained to a series of different values to generate the torsion curve at the constrained bond angle. *ϕ*_min_ is the dihedral value that has the lowest energy. The column labeled ‘QM norm ratio’ shows the ratio of the QM torsion norm for the constrained bond angle to the fully relaxed torsion norm (the ‘optimized norm’) displayed in Table 3. ADDT and CADT model predictions are listed in the last four columns

Molecule	*θ*_constr_ (°)	*θ*_other_ (°) at *ϕ*_min_	*ϕ*_min_ (°)	QM torsion norm (kJ mol^−1^)	QM norm ratio	ADDT predicted *ϕ*_min_ (°)	ADDT predicted norm (kJ mol^−1^)	ADDT predicted norm ratio	CADT prediction equal to optimized norm (kJ mol^−1^)
C(OH)ClFH	99.0 (COH)	110.5 (FCO)	−60	4.61	0.98	−65	5.55	1.18	4.72
C(OH)ClFH	119.0 (COH)	111.5 (FCO)	−68	4.86	1.03	−65	3.80	0.81	4.72
FSSF	97.2 (SSF)	106.0 (FSS)	±87^a^	39.83	0.91	±∼85^a^	51.4	1.17	43.8
FSSF	117.2 (SSF)	108.2 (FSS)	±88^a^	60.04	1.37	±∼86^a^	35.0	0.80	43.8
H_2_O_2_	90.8 (OOH)	100.6 (HOO)	±121^a^	14.45	1.35	±∼111^a^	11.9	1.11	10.7
H_2_O_2_	110.8 (OOH)	101.0 (HOO)	±103^a^	8.41	0.79	±∼112^a^	9.15	0.86	10.7
IF_3_ClOH	97.1 (IOH)	84.4 (ClIO)	180	1.21	1.74	180	0.86	1.23	0.70
IF_3_ClOH	117.1 (IOH)	83.0 (ClIO)	180	0.55	0.78	180	0.51	0.73	0.70
N_2_O_2_	93.4 (NNO)	98.7 (ONN)	0	7.79	1.45	0	6.50	1.21	5.38
N_2_O_2_	113.4 (NNO)	107.5 (ONN)	0	3.55	0.66	0	4.20	0.78	5.38
PF_4_OH	103.1 (POH)	88.3 (FPO)	180 (−60, 60)^b^	2.04	1.21	60 (−60, 180)^b^	2.11	1.26	1.67^c^
PF_4_OH	123.1 (POH)	88.0 (FPO)	180 (−60, 60)^b^	1.33	0.79	60 (−60, 180)^b^	1.19	0.71	1.67^c^

a This molecule's QM energy is unchanged upon mirror reflection which changes the dihedral's sign. For the FSSF and HOOH molecules, the ADDT model only approximately (not exactly) captures this reflection symmetry.

b Due to the 3-fold symmetry of this molecule, there are chemically equivalent minima at *ϕ* = 60°, 180°, and −60°.

c For PF_4_OH, the model-fitted fully relaxed norm from the seven fitted dihedral modes is 1.67 kJ mol^−1^ compared to 1.69 kJ mol^−1^ for a complete modal expansion; this reflects the SumCSq value of 0.98 as listed in Table 3.

Table 7 lists summary statistics for the ADDT and CADT models. The mean log_10_ error (MLE) was defined as the average value of log_10_[predicted_norm/QM_norm]. The mean unsigned log_10_ error (MULE) was defined as the average value of abs[log_10_[predicted_norm/QM_norm]]. For dataset 1, the ADDT model performed much better than the CADT model. For dataset 2, both models performed acceptably and neither was substantially more accurate than the other. Given the much lower computational cost of the CADT model compared to the ADDT model, this clearly leads to the following conclusions. If the dihedral contains a bond angle whose equilibrium value is ≥ 130°, the ADDT model is preferred both due to its higher accuracy and also due to its higher stability, because it yields continuous derivatives even if the perturbed bond angle reaches linearity (*i.e.*, 180°) during a molecular dynamics or Monte Carlo simulation. If both contained bond angles in the dihedral have equilibrium values < 130°, then these bond angles are likely to be sufficiently stiff that it will be extremely rare for the perturbed bond angle to reach linearity (*i.e.*, 180°) during a molecular dynamics or Monte Carlo simulation. In this case, the CADT model is preferred, because it is computationally cheaper than the ADDT model yet of comparable accuracy for these dihedrals.

**Table 7:** Summary statistics comparing the ADDT model potential to the CADT model potential. MLE = mean log_10_ error; MULE = mean unsigned log_10_ error

Dataset	ADDT model		CADT model	
	MLE	MULE	MLE	MULE
An equil. angle ≥ 130°	0.098	0.098	−0.647	0.647
Both equil. angles < 130°	−0.035	0.087	−0.019	0.109

### 10.3 Detailed torsion analysis for rotatable dihedrals using ADCO and CACO model potentials

The goal of this section is to repeat the analysis of Section 10.2 except using the ADCO and CACO model potentials in place of the ADDT and CADT model potentials. The key principle to keep in mind is that the CADT model potential is functionally equivalent to a full Fourier series expansion of the torsion potential as a function of the dihedral value *ϕ* subject to the constraint that232
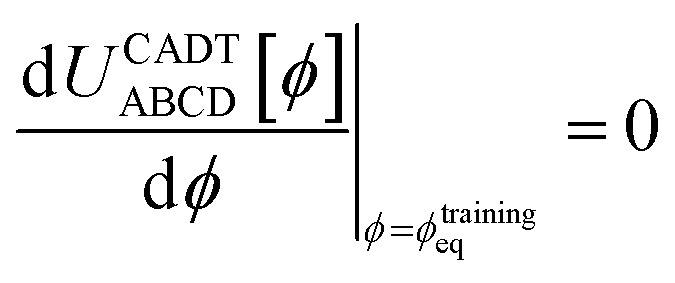


In stark contrast, the CACO model potential spans the subset of even functions of the dihedral value *ϕ* while omitting all odd-function contributions. A constraint analogous to eqn (232) is not imposed for the CACO model potential; however, if *sym*_value = 0 and the CACO model potential is untruncated then fitting the CACO model potential to a torsion scan curve should cause d*U*^CACO^_ABCD_[*ϕ*]/d*ϕ*|^training^_*ϕ*=*ϕ*eq_ to asymptotically approach zero.

**Fig. 11:**
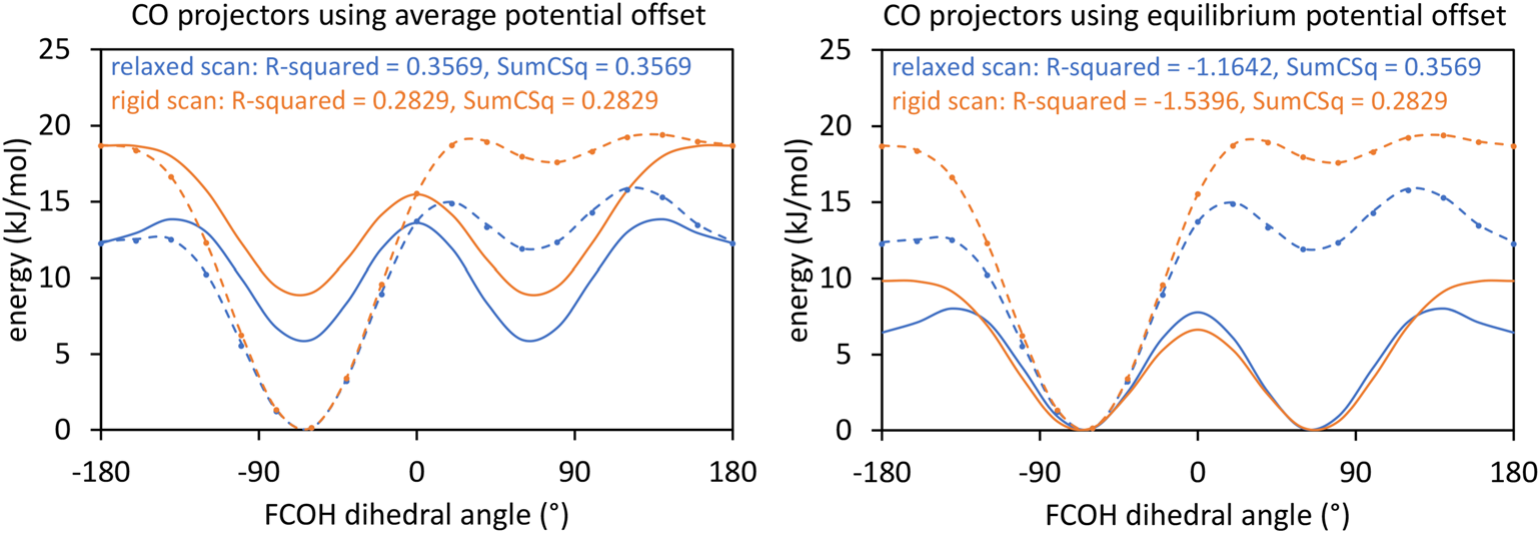
When *sym*_value > 0, then the CO projectors do not provide a good description of the dihedral scan curve. This figure compares model fits using the DT projectors (dashed lines) to CO projectors using the average potential offset (solid lines in lefthand panel) and CO projectors using the equilibrium potential offset (solid lines in righthand panel) for the S enantiomer of C(OH)ClFH. The filled circles show the QM-computed (CCSD/def2-TZVPD) values. Bond angles and bond lengths were held fixed to generate the results shown in orange. Bond angles and bond lengths were relaxed to generate the results shown in blue. When using the DT projectors, the *R*-squared values were 0.9995 (relaxed scan) and 0.9999 (rigid scan), and the SumCSq values using the DT projectors were 0.9999 (relaxed scan) and 1.0000 (rigid scan).

It directly follows that CO projectors (which are used by the CACO and ADCO model potentials) provide asymptotically close to functionally equivalent results to the DT projectors (which are used by the CADT and ADDT model potentials) for a torsion scan curve that has no odd-function contributions; that is, when *sym*_value = 0. When *sym*_value = 0, the SumCSq values from Table 3 computed using the DT projectors were nearly identical to the SumCSq values in Table 8 computed using the CO projectors. On the other hand, when *sym*_value ≠ 0 (*e.g.*, *sym*_value = 0.847 for the FCOH dihedral in the C(OH)ClFH molecule) then the SumCSq value using the CO projectors was much smaller than the SumCSq value using the DT projectors. When *sym*_value = 0, the model fits using the CO projectors were nearly identical to those using the DT projectors; this is visually illustrated by comparing the torsion scan curves in Fig. 8 (using the DT projectors) to those in ESI Fig. S1† (using the CO projectors). However, the CO projectors performed markedly worse than the DT projectors when *sym*_value ≠ 0 (*e.g.*, for the FCOH dihedral in the C(OH)ClFH molecule as shown in Fig. 11).

**Table 8:** Torsion mode analysis using the CO projectors for 13 molecules at the CCSD/def2-TZVPD level of theory. For the results without parentheses, all bond angles and bond lengths were held fixed at values taken from the ground-state structure, and the dihedral value was rigidly scanned to generate the energy curves. Where available, results shown inside parentheses allowed the bond angles and bond lengths to relax as the constrained dihedral value was scanned to generate the energy curves. For HNCO, HNCS, HONC, and HSNC, results in square brackets allowed the bond lengths to relax while the bond angles were held rigid

Molecule	Atoms in dihedral	*ϕ*_eq_ (°)	QM torsion barrier (kJ mol^−1^)	QM torsion norm (kJ mol^−1^)	*sym*_value	*c*_1_	*c*_2_	*c*_3_	*c*_4_	SumCSq
(CClFH)_2_	HCCH	180.0 (180.0)	73.15 (39.98)	19.40 (10.42)	0.000 (0.000)	0.5943 (0.5058)	0.3795 (0.2707)	0.7048 (0.8123)	0.0690 (0.0807)	0.9987 (0.9955)
C(OH)ClFH	FCOH	−64.70 (−64.70)	19.39 (15.78)	6.55 (4.72)	0.847 (0.802)	−0.3577 (−0.2859)	0.3475 (0.3551)	0.1848 (0.3860)	−0.0092 (−0.0090)	0.2829 (0.3569)
Ethane	HCCH	180.0 (180.0)	12.65 (12.10)	4.47 (4.28)	0.000 (0.000)	0.0000 (0.0000)	0.0000 (0.0000)	1.0000 (0.9996)	0.0000 (0.0000)	1.0000 (0.9992)
FSSF	FSSF	87.42 (87.42)	212.0 (129.6)	69.23 (43.80)	0.000 (0.000)	−0.0908 (0.0103)	0.9771 (0.9943)	0.0101 (0.1011)	0.1797 (−0.0035)	0.9953 (0.9990)
Glyoxal	OCCO	180.0 (180.0)	25.41 (24.86)	8.48 (8.52)	0.000 (0.000)	0.8090 (0.8215)	−0.5842 (−0.5658)	0.0599 (0.0033)	0.0255 (0.0694)	0.9999 (0.9999)
H_2_O_2_	HOOH	111.1 (111.1)	35.75 (31.56)	11.99 (10.68)	0.000 (0.000)	0.8339 (0.8390)	0.5495 (0.5430)	0.0500 (0.0361)	0.0094 (0.0001)	1.0000 (1.0000)
HNCO	HNCO	180.0 [180.0]	14.86 [14.78]	5.25 [5.23]	0.000 [0.000]	1.0000 [1.0000]	0.0011 [−0.0004]	0.0004 [0.0003]	0.0000 [0.0000]	1.0000 [1.0000]
HNCS	HNCS	180.0 [180.0]	8.19 [8.15]	2.90 [2.88]	0.000 [0.000]	1.0000 [1.0000]	0.0043 [0.0030]	0.0002 [0.0002]	0.0000 [0.0000]	1.0000 [1.0000]
HONC	HONC	180.0 [180.0]	2.54 [2.54]	0.903 [0.902]	0.000 [0.000]	0.9950 [0.9950]	−0.1001 [−0.1003]	0.0001 [0.0002]	0.0000 [0.0000]	1.0000 [1.0000]
HSNC	HSNC	180.0 [180.0]	1.89 [1.89]	0.675 [0.673]	0.000 [0.000]	0.9914 [0.9914]	−0.1307 [−0.1310]	0.0000 [0.0002]	0.0000 [−0.0001]	1.0000 [1.0000]
IF_3_ClOH	ClIOH	180.0 (180.0)	8.95 (2.70)	2.91 (0.70)	0.000 (0.000)	0.7753 (0.4091)	−0.6228 (−0.2565)	0.0930 (0.2593)	−0.0488 (−0.8356)	0.9999 (0.9987)
N_2_O_2_	ONNO	0.00 (0.00)	26.13 (18.99)	8.40 (5.38)	0.000 (0.000)	−0.7431 (−0.3967)	−0.5981 (−0.8543)	−0.2981 (−0.3347)	0.0254 (−0.0272)	0.9994 (1.0000)
PF_4_OH	FPOH	180.0 (180.0)	10.20 (4.46)	3.34 (1.69)	0.000 (0.000)	0.0000 (0.0000)	0.0000 (0.0000)	0.9862 (0.9922)	0.0000 (0.0000)	0.9727 (0.9844)

Using the trigonometric identities in eqn (26) and (27), the DT projectors for modes 1 to 4 can be re-written as

233 *P*^DT^_*n*_[*ϕ*] = −cos[*n*(*ϕ* − *ϕ*^training^_eq_)] = −cos[*nϕ*^training^_eq_]cos[*nϕ*] − sin[*nϕ*^training^_eq_]sin[*nϕ*]

Inserting this into eqn (164) gives234
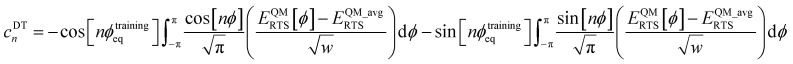
When *sym*_value = 0, the odd-function components of the torsion potential are zero, which means that235
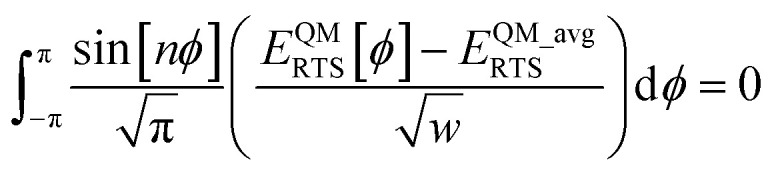
so that eqn (234) reduces in this case to236
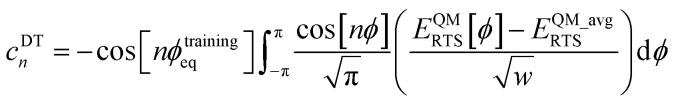


Combining eqn (145), (164), and (236) gives

237 *Δ*_*n*_ = *c*^DT^_*n*_ + *c*^CO^_*n*_cos[*nϕ*^training^_eq_]

equals zero when *sym*_value = 0. This is numerically confirmed for rigid torsion scans and relaxed (or partly relaxed) torsion scans of 12 molecules in Table S1 of the ESI.†

The torsion offset potential for the ADCO model potential can be computed by substituting *ϕ* = *ϕ*^training^_eq_ into the ADCO model potential:238
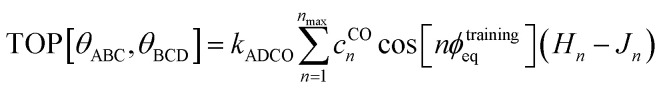
Comparing this to the TOP using the ADDT model potential239
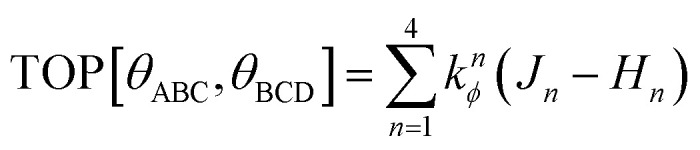
shows the two are equal iff

240 *k*^*n*^_*ϕ*_ = −*k*_ADCO_*c*^CO^_*n*_cos[*nϕ*^training^_eq_]

for *n* = 1 to 4. When *sym*_value = 0, then *Δ*_*n*_ = 0 so eqn (240) holds in this case. The ADCO model potential and eqn (240) do not apply when *sym*_value ≠ 0.

The results shown in Table S1 of the ESI† confirm that eqn (240) is satisfied for all 12 molecules having *sym*_value = 0. Accordingly, the angle-scan curves shown in Fig. 9 are unchanged when replacing the ADDT model potential with the ADCO model potential for those molecules having *sym*_value = 0. This includes all molecules shown in Fig. 9 except C(OH)ClFH (for which *sym*_value = 0.847). In summary, the ADCO model potential gives angle-scan curves and TOP identical to those of the ADDT model potential for 
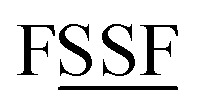
, 
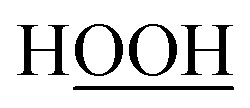
, 
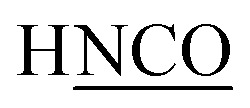
, 
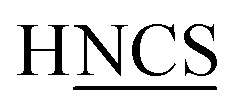
, 
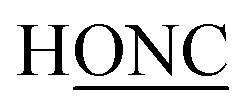
, 
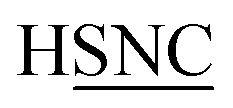
, 
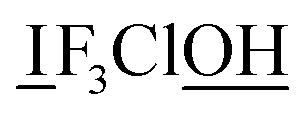
, 
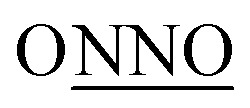
, and 
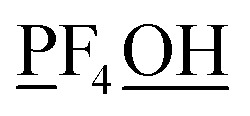
.

ESI Table S2† lists ADCO and CACO model predictions for four molecules (HNCO, HNCS, HONC, and HSNC) that have at least one equilibrium bond angle ≥ 130°. ESI Table S2† re-analyzes the systems and constrained bond angles that were previously analyzed using DT projectors in Tables 4 and 5. Although the mode coefficients using CO projectors were different from those using the DT projectors, the SumCSq values (*i.e.*, SumCSq = 1.0000) and predicted norms for each of these four molecules using the CO projectors were identical to those using the DT projectors.

Table 9 lists ADCO and CACO model predictions for five molecules (FSSF, H_2_O_2_, IF_3_ClOH, N_2_O_2_, and PF_4_OH) that have both equilibrium bond angles < 130° and *sym*_value = 0. For these five molecules, the ADCO and CACO predicted norms in Table 9 are identical to the ADDT and CADT predicted norms, respectively, in Table 6.

**Table 9:** Comparison of QM-computed (CCSD/def2-TZVPD) and ADCO model-predicted torsion norms for several molecules having both included equilibrium bond angles < 130° and *sym*_value = 0. *ϕ*_min_ is the dihedral value that has the lowest energy. The column labeled ‘QM norm ratio’ shows the ratio of the QM torsion norm for the constrained bond angle to the fully relaxed torsion norm. CACO and ADCO model predictions are listed in the last five columns. Δ*θ*_constr_ = *θ*_constrained_ − *θ*_eq_

Molecule	Δ*θ*_constr_ (°)	*ϕ*_min_ (°)	QM torsion norm (kJ mol^−1^)	QM norm ratio	CACO predicted *ϕ*_min_ (°)	CACO predicted norm (kJ mol^−1^)	ADCO predicted *ϕ*_min_ (°)	ADCO predicted norm (kJ mol^−1^)	ADCO predicted norm ratio
FSSF	0 (SSF)	±87.42	43.80	1.00	±85.9	43.8	±85.9	43.8	1.00
FSSF	−10 (SSF)	±86.73	39.83	0.91	±85.9	43.8	±85.5	51.4	1.18
FSSF	+10 (SSF)	±87.72	60.04	1.37	±85.9	43.8	±89.3	35.0	0.80
H_2_O_2_	0 (OOH)	±111.1	10.68	1.00	±111.3	10.7	±111.3	10.7	1.00
H_2_O_2_	−10 (OOH)	±120.8	14.45	1.35	±111.3	10.7	±120.8	11.9	1.11
H_2_O_2_	+10 (OOH)	±103.5	8.41	0.79	±111.3	10.7	±103.5	9.15	0.86
IF_3_ClOH	0 (IOH)	180.0	0.70	1.00	180.0	0.70	180.0	0.70	1.00
IF_3_ClOH	−10 (IOH)	180.0	1.21	1.74	180.0	0.70	180.0	0.86	1.23
IF_3_ClOH	+10 (IOH)	180.0	0.55	0.78	180.0	0.70	180.0	0.51	0.73
N_2_O_2_	0 (NNO)	0.00	5.38	1.00	0.00	5.38	0.00	5.38	1.00
N_2_O_2_	−10 (NNO)	0.00	7.79	1.45	0.00	5.38	0.00	6.50	1.21
N_2_O_2_	+10 (NNO)	0.00	3.55	0.66	0.00	5.38	0.00	4.20	0.78
PF_4_OH	0 (POH)	180 (−60, 60)^a^	1.69	1.00	180 (−60, 60)^a^	1.67^b^	180 (−60, 60)^a^	1.67^b^	1.00
PF_4_OH	−10 (POH)	180 (−60, 60)^a^	2.04	1.21	180 (−60, 60)^a^	1.67^b^	180 (−60, 60)^a^	2.11	1.26
PF_4_OH	+10 (POH)	180 (−60, 60)^a^	1.33	0.79	180 (−60, 60)^a^	1.67^b^	180 (−60, 60)^a^	1.19	0.71

a Due to the 3-fold symmetry of this molecule, there are chemically equivalent minima at *ϕ* = 60°, 180°, and −60°.

b For PF_4_OH, the model-fitted fully relaxed norm from the four fitted CO dihedral modes is 1.67 kJ mol^−1^ compared to 1.69 kJ mol^−1^ for a complete modal expansion; this reflects the SumCSq value of 0.98 as listed in Table 8.

For each value of the constrained bond angle in Table 9 and ESI Table S2,† the ADCO predicted *ϕ*_min_ value was computed by calculating the full 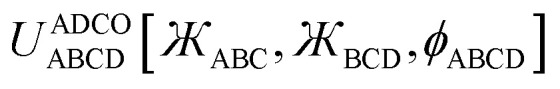 curve over the range −180° < *ϕ*_ABCD_ ≤ 180° in 0.01° increments and identifying which value of *ϕ*_ABCD_ produced the minimum value of 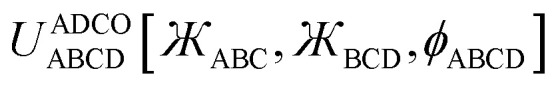. The CACO predicted *ϕ*_min_ value remains constant as the bond angle changes, because the CACO model potential is not a function of bond angle. The CACO and ADCO predicted *ϕ*_min_ values are equal at the optimized bond angle value (*i.e.*, at Δ*θ*_constr_ = 0).

In Table 9 and ESI Table S2,† the ADCO predicted torsion norm was computed as241
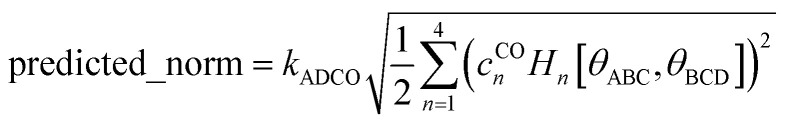
The factor of 
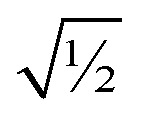
 appears in eqn (241), because the root-mean-squared value of cosine functions is 
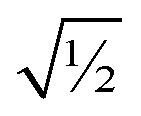
. Table 10 summarizes the mean log_10_ error (MLE) and mean unsigned log_10_ error (MULE) for the ADCO and CACO model predicted torsion norms. When (*θ*^eq^_ABC_ or *θ*^eq^_BCD_) ≥ 130°, the ADCO model was much more accurate than the CACO model. When (*θ*^eq^_ABC_ and *θ*^eq^_BCD_) < 130°, both the ADCO and CACO models performed acceptably and had similar accuracy to each other.

**Table 10:** Summary statistics comparing the ADCO model potential to the CACO model potential for molecules having *sym*_value = 0. MLE = mean log_10_ error; MULE = mean unsigned log_10_ error

Dataset	ADCO model		CACO model	
	MLE	MULE	MLE	MULE
An equil. angle ≥ 130°	0.098	0.098	−0.647	0.647
Both equil. angles < 130°	−0.039	0.086	−0.022	0.128

As shown in Fig. 10, the ADCO model correctly predicted trends for changes in *c*^CO^_*n*_ values as the constrained bond angle changed in the HONC and HSNC molecules. The predicted coefficients were computed using242
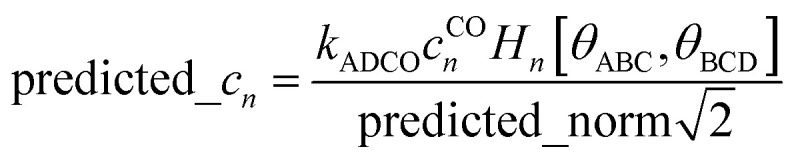


In summary, when *sym*_value = 0 then the ADDT and ADCO models provide nearly functionally equivalent results to each other if all seven ADDT modes and all four ADCO modes are included in the model potential. If some of the less important (but nonzero) modes are removed during smart selection so that the model is restricted to the more important ADDT or ADCO modes, then this introduces additional approximation that can make model results slightly different for the ADDT and ADCO model potentials. When *sym*_value = 0, then the CADT and CACO models provide nearly functionally equivalent results to each other if all seven CADT modes and all four CACO modes are included in the model potential. If some of the less important (but nonzero) modes are removed during smart selection so that the model is restricted to the more important CADT or CACO modes, then this introduces additional approximation that can make model results slightly different for the CADT and CACO model potentials. Of course, the ADCO and CACO model potentials are not good approximations if *sym*_value differs substantially from zero.

### 10.4 Slip torsion and the indispensable torsion offset potential

**Fig. 12:**
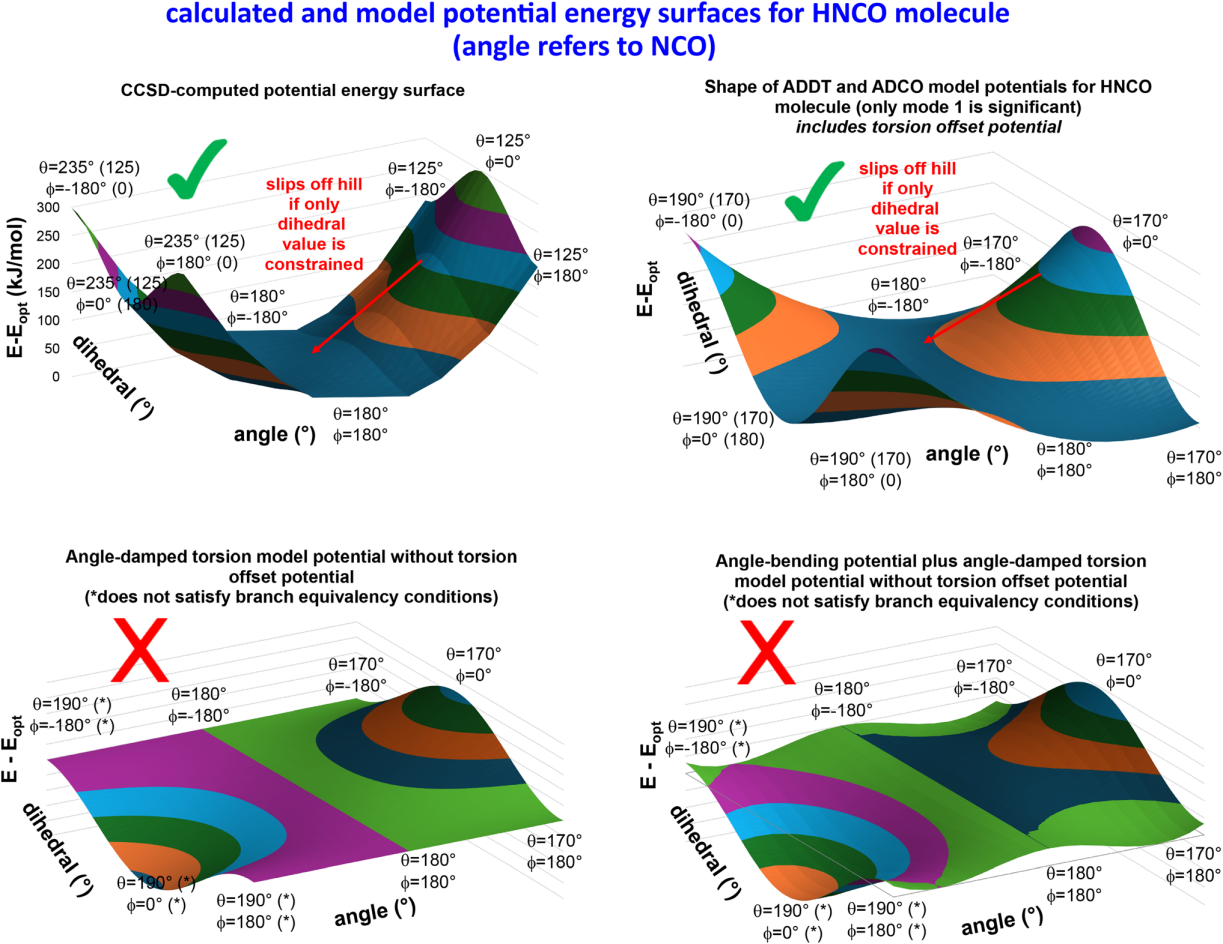
Calculated and model potential energy surfaces of the HNCO molecule demonstrating that it is absolutely critical to include the torsion offset potential. This potential energy surface also illustrates the ‘slip torsion’ phenomenon. The CCSD-calculated potential energy surface (upper left panel) matches the ADDT and ADCO model potential energy surfaces (upper right panel) which include the mode 1 term 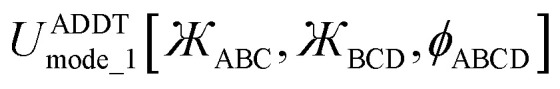 or 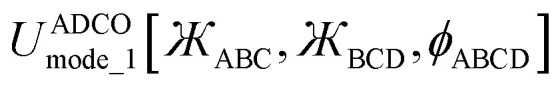 as the model potential. Both the CCSD-calculated and the ADDT and ADCO model potentials satisfy the angle-dihedral coordinate branch equivalency condition. For example, in the upper left panel the point marked (*θ*, *ϕ*) = (235°,-180°) is a branch replicate of the point marked (*θ*, *ϕ*) = (125°,0°). For comparison, the two lower panels show model potentials that do not include the torsion offset potential. The lower left panel shows the same potential model as the upper right panel, except the lower left panel excludes the torsion offset potential. This omission destroys the angle-dihedral coordinate branch equivalency and makes the model useless, because it is physically inconsistent. As shown in the lower right panel, adding an angle-bending potential does not rectify the physical inconsistency created by omitting the torsion offset potential.

By comparing model potential energy surfaces to the CCSD-calculated potential energy surface, Fig. 12 shows that including the torsion offset potential is absolutely required to describe angle-damped dihedral torsions. The CCSD-calculated potential energy surface used the underlying quantum chemistry data for the constrained torsion scans at NCO angle = 125°, 140°, 155°, and 165° listed in Table 4, plus the energy for linear (180°) bond angle. The model potential energy surfaces were generated using the torsion parameters listed in Tables 3 and 8 for the partly relaxed torsion scan. (For the lower right panel in Fig. 12, the added angle-bending potential used a greatly exaggerated value of *k*_angle_ = 2000 kJ mol^−1^ with my new angle-bending potential to make the effect of including an angle-bending potential more visible.) The CCSD potential energy surface was interpolated between data points to make it appear smoother. The model potential energy surfaces were computed in 1°(angle) and 5°(dihedral) increments. Agreement between the CCSD-calculated and ADDT/ADCO model potential energy surfaces was excellent. As shown in Fig. 12, potential energy surfaces omitting the torsion offset potential had incorrect shapes, and this makes them bad models. This conclusion still holds when any conceivable kind of angle-bending potential is included without including the torsion offset potential.

For this molecule, the ADDT and ADCO model potentials happened to coincide with each other for the following reasons. As shown in Table 3, the ADDT projection coefficients *c*_i_ are zero for all of the sine modes in this molecule. Since *ϕ*_eq_ = 180° for this molecule, it directly follows that cos[*m*(*ϕ* − *ϕ*_eq_)] = (−1)^*m*^cos[*mϕ*], and this means the coefficient for cosine mode *m* in this molecule has the same magnitude but not necessarily the same sign for the ADDT and ADCO model potentials. Examining Tables 3 and 8, mode 1 dominates (*i.e.*, abs[*c*_1_] = 1.0000) for the partly relaxed torsion scan. For mode 2, abs[*c*_2_] = 0.0004 is below the smart selection thresholds for the ADDT and ADCO model potentials. The remaining ADDT and ADCO modes have smaller magnitude coefficients that make them negligible.

In some materials, the angle-damped dihedral torsion (which includes the torsion offset potential) gives rise to a new physical phenomenon called ‘slip torsion’. This is illustrated in Fig. 12 for the HNCO molecule. In this molecule, the optimized dihedral value is *ϕ*_eq_ = 180°. Consider a geometry optimization in which the HNCO dihedral value is constrained but all other geometric parameters are allowed to relax. When the HNCO dihedral is constrained to a value of *ϕ* between −90° and +90°, the molecule is situated on the side of a potential energy hill. The molecule's energy can be lowered by sliding down the hill in the direction of increasing bond angle, *θ*_NCO_. When the molecule slides down as far as *θ*_NCO_ = 180°, then the HNCO dihedral value for this non-equilibrium linear bond angle becomes undefined and the constrained geometry calculation crashes (*i.e.*, abruptly terminates due to undefined dihedral value). True to form, this is exactly what happened when attempting to calculate fully relaxed torsion scans for the HNCO, HNCS, HOCN, and HSNC molecules. For these molecules, constraining only the dihedral value produced converged results when −180° < *ϕ* < −90° and 90° < *ϕ* ≤ 180°, while the calculations did not converge when −90° < *ϕ* < 90°. As explained in Section 10.2, converged results were achieved by performing partly relaxed dihedral scans in which both bond angles were constrained in addition to constraining the dihedral's value.

### 10.5 Potential energy surfaces for linear dihedrals

For five molecules with linear dihedrals, CCSD/def2-TZVPD calculations were performed at a series of constrained bond angles and constrained dihedral values to generate potential energy surfaces. These constrained geometry optimizations were performed in Gaussian 16 software using the opt = modredundant method. For the calculations used in this subsection, the bond lengths were not constrained. For the three molecules containing single-linear dihedrals, the angle for which *θ*_eq_ ≠ π was not constrained, while the other angle was constrained to 130°, 145°, and 160°. For the two molecules containing double-linear dihedrals, the two bond angles were constrained to all combinations of *θ*_ABC_,*θ*_BCD_ ∈ {150°,160°,170°}. Dihedrals were constrained to a series of symmetry unique values to generate data for the full range −180° < *ϕ* ≤ 180° at each constrained bond angle. Finally, the relative energy difference (*E*^el^_*μ*_ − *E*^el^_opt_) was computed for each geometry *μ*, where *E*^el^_opt_ is the QM-computed electronic energy of the molecule's fully unconstrained ground-state geometry.

The Generalized Reduced Gradient (GRG) solver in Excel was used to optimize the force constants of the ADLD model potential plus the Manz angle-bending potential. These force constants were optimized to maximize the R-squared value (see eqn (17)) using the following definitions243
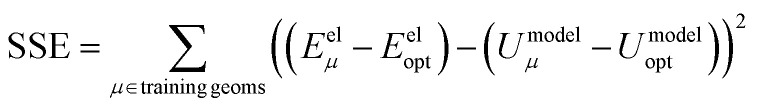
244
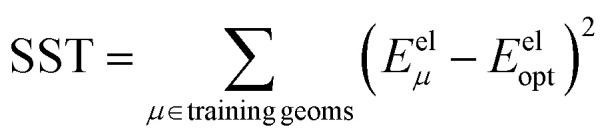
while constraining each optimized force constant to be non-negative. For the single-linear dihedrals, the Manz angle-bending potential was included only for the bond angle that was constrained to a series of different values. For the double-linear dihedrals, the Manz angle-bending potential was included for both bond angles, because these were constrained to a series of different values. Because the unconstrained optimized geometry of each molecule contained mirror planes, the odd-function (*i.e.*, sine modes) in the ADLD model potential have zero contributions and were not included in the fitting process. For the even-function (*i.e.*, cosine modes), the multiplicities (*i.e.*, *n* values) were chosen to match the n-fold rotational symmetry of the molecule's unconstrained optimized geometry. For example, H_3_CCN has a 3-fold rotational symmetry, while H_2_BCN and H_2_BNC have 2-fold rotational symmetry. Table 11 summarizes the optimized force constants and R-squared values. The R-squared values were >0.99 indicating superb fits of the ADLD model potential.

**Table 11:** Optimized ADLD force constants and *R*-squared values for five molecules having linear dihedrals. The number of unique geometries includes the optimized ground-state geometry plus displaced geometries

Formula	Number unique geometries in training set	Force constants optimized to nonzero values (kJ mol^−1^)	Force constants optimized to zero	*R*-squared
HCCH	61	*k* ^1^_LD5_ = 322.92, (both) *k*^Manz_bend^_CCH_ = 105.25	*k* ^1^_LD1_ = *k*^1^_LD2_ = *k*^1^_LD4_ = 0	0.9988
HCNO	91	*k* ^1^_LD5_ = 473.10, *k*^Manz_bend^_CNO_ = 329.99	*k* ^Manz_bend^_HCN_ = *k*^1^_LD1_ = *k*^1^_LD2_ = *k*^1^_LD4_ = 0	0.9974
H_3_CCN	22	*k* ^2^_LD5_ = 5.249, *k*^Manz_bend^_CCN_ = 194.90	*k* ^2^_LD4_ = 0	0.9993
H_2_BCN	22	*k* ^1^_LD2_ = 182.43, *k*^Manz_bend^_BCN_ = 98.14	*k* ^1^_LD1_ = 0	0.9967
H_2_BNC	22	*k* ^1^_LD2_ = 106.30, *k*^Manz_bend^_BNC_ = 61.50	*k* ^1^_LD1_ = 0	0.9971

**Fig. 13:**
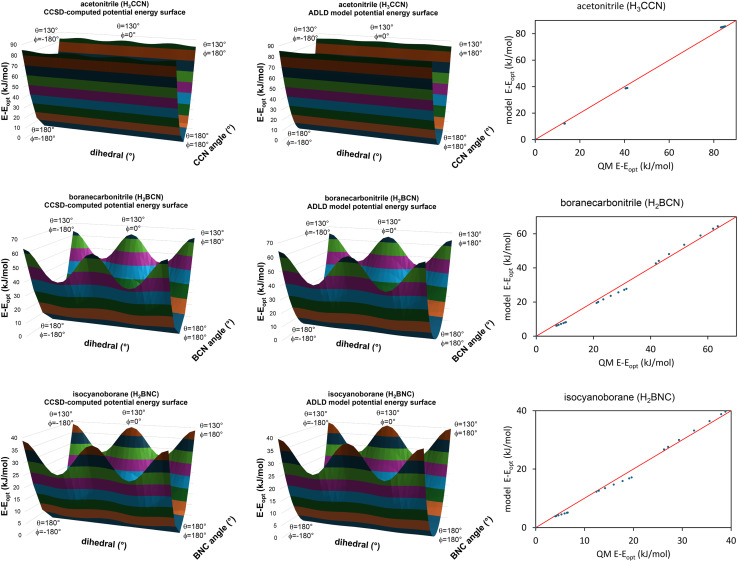
Comparison of QM-computed CCSD potential energy surfaces (lefthand panels) to ADLD model potential energy surfaces (middle panels) for three molecules having single-linear dihedrals. The righthand panels are parity plots comparing QM-computed CCSD energies to ADLD model potential energies for the computed datapoints for these three molecules. Note: these surface plots used linear interpolation between computed datapoints to create the appearance of smooth surfaces.

**Fig. 14:**
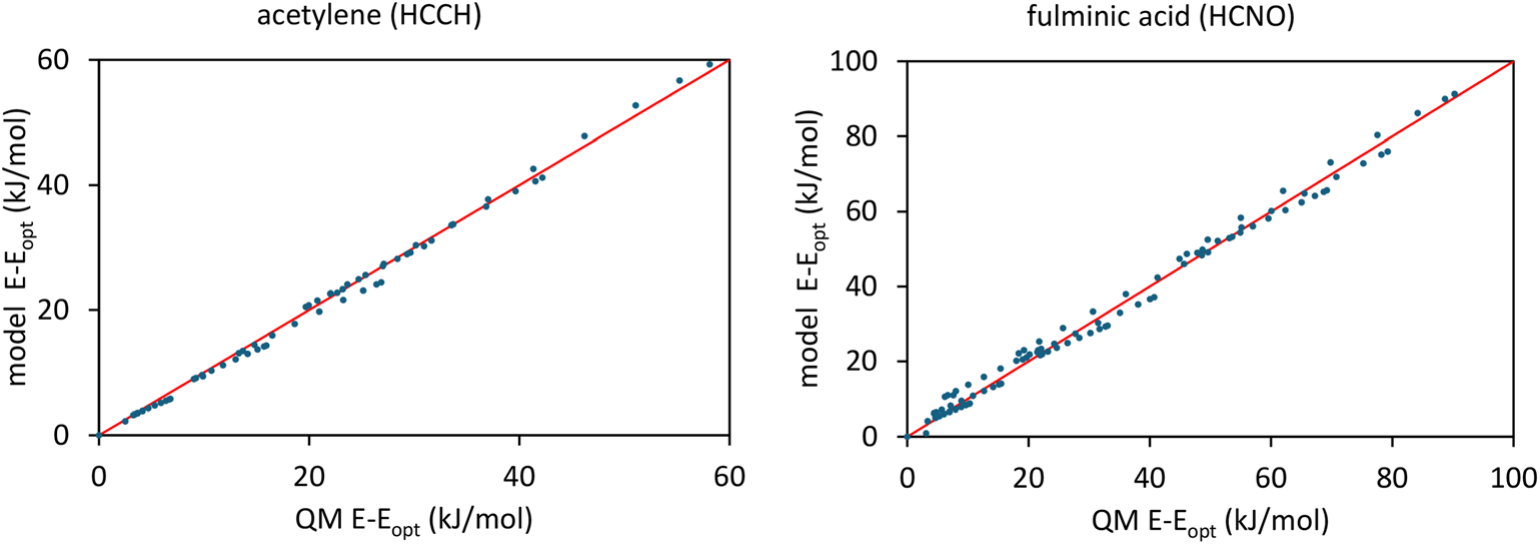
Parity plots comparing QM-computed CCSD energies to ADLD model potential energies for two molecules having double-linear dihedrals. This figure contains all of the computed datapoints used to generate the contour plots in Fig. 15.

**Fig. 15:**
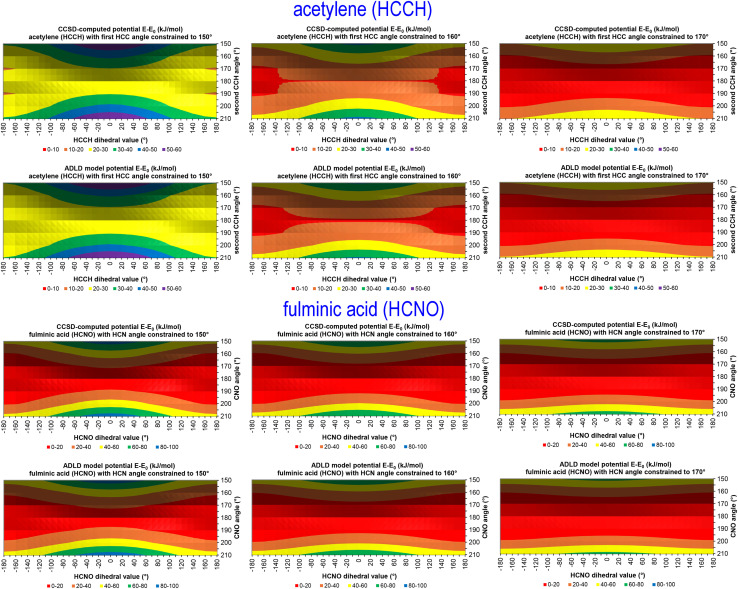
Comparison of QM-computed CCSD potential energy contour plots (first and third rows) to ADLD model potential energy contour plots (second and fourth rows) for two molecules having double-linear dihedrals. Note: these contour plots use interpolation between computed datapoints.

As shown in Fig. 13, the ADLD model potential energy surfaces closely reproduced the QM-computed CCSD potential energy surfaces for the three molecules with single-linear dihedrals. Fig. 13 also contains parity plots showing excellent agreement between the QM-computed CCSD energies and the ADLD model predicted energies for the computed datapoints. As shown in Fig. 14 and 15, the ADLD model potential closely reproduced the CCSD-computed energies for the two molecules with double-linear dihedrals. Overall, these results showed that my ADLD model potential (when including the Manz angle-bending potential) does an excellent job of describing potential energy surfaces for linear dihedrals.

In Fig. 15, each midline corresponds to one of the bond angles being constrained to 180° while the other bond angle was constrained to 150°, 160°, or 170°. The corresponding CCSD energy for each midline was computed in Gaussian16 (ref. 33) using the keyword opt = (ModRedundant,GIC) where GIC stands for Generalized Internal Coordinates. Since one of the angles was linear (*i.e.*, 180°) along this midline, its dihedral value was undefined. Using GIC allowed the constrained midline geometry during the CCSD calculation to be specified using constrained linear bend internal coordinates plus unconstrained bond lengths and constrained nonlinear bond angle. (These midlines were not included in the training dataset used to optimize the force constants.) For the ADLD model panels in Fig. 15, the energy along the corresponding midline was computed directly from the parameterized ADLD model potential.

### 10.6 Brief recap of results for metal–organic frameworks containing linear dihedrals

**Fig. 16:**
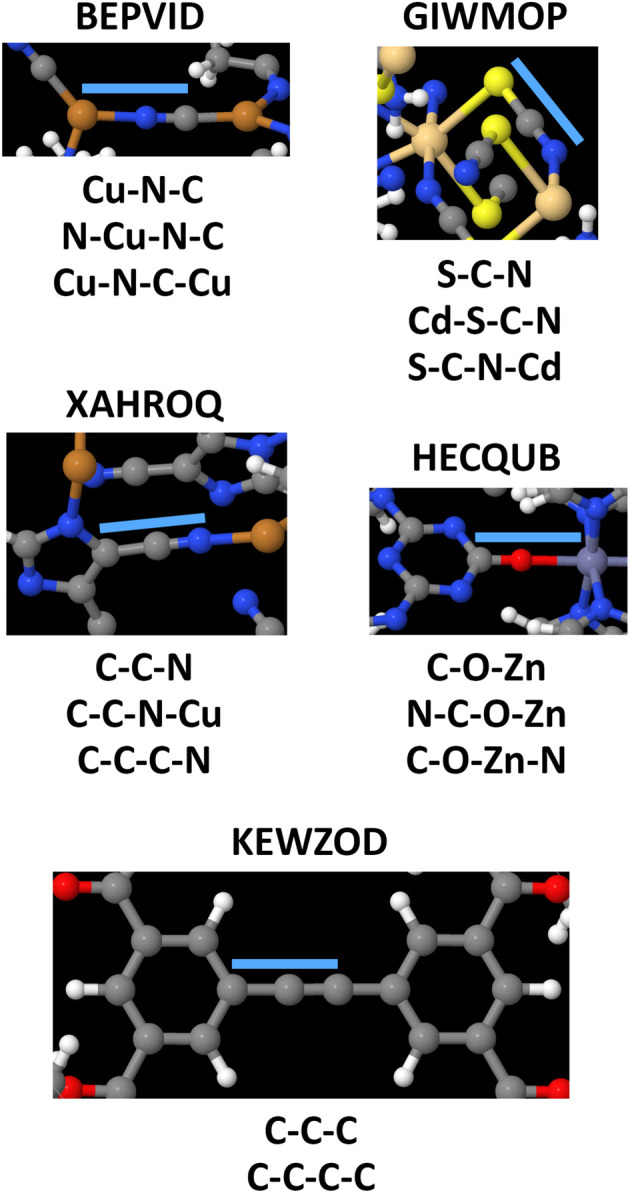
Some linear dihedrals in MOFs studied in ref. 18 and 19: BEPVID, GIWMOP, HECQUB, KEWZOD, and XAHROQ. In each panel, the blue line is adjacent to the linear bond angle. Below each panel are listed the atomic symbols comprising the linear bond angle and the associated after-pruning linear dihedrals.

In a companion article, 5 out of 116 MOFs studied contained after-pruning linear dihedrals.^18,19^ As shown in Fig. 16, these included: (a) accidental single-linear dihedrals (Case #3) in the MOFs BEPVID, GIWMOP, and XAHROQ, (b) a symmetry-induced single-linear dihedral (Case #1a) in HECQUB, and (c) both a symmetry-induced double-linear dihedral (Case # 2, which locally looks like Case # 2a) and a symmetry-induced single-linear dihedral (Case # 1, which locally looks like Case # 1a) in KEWZOD. In the companion article, the *n* = 2 *j* but not the *n* = (2 *j* – 1) modes shown in eqn (223) were derived using part of the theoretical analysis described in Section 9.1 above.^18^ The optimized flexibility models for those 5 MOFs included flexibility terms for the single-linear dihedrals and used the modes corresponding to the force constants *k*^1^_LD1_ and *k*^1^_LD2_ in eqn (223).^18,19^ For KEWZOD, the single-linear dihedral was included in the parameterized flexibility model, but the double-linear dihedral was not included in the parameterized flexibility model.

A linear dihedral has no torsion barrier at its equilibrium bond angle. For small deviations of the bond angle from its equilibrium value, the torsion barrier will be small in magnitude. Consequently, it is often not required to include torsion potentials for the linear dihedrals when constructing classical forcefields for complex materials. For the 5 MOFs having after-pruning linear dihedrals, flexibility models parameterized with and without including torsion potentials for the linear dihedrals produced essentially the same validation *R*-squared values.^18,19^ These *R*-squared values are listed in Table 12.

**Table 12:** Comparison of validation R-squared values for 5 MOFs containing after-pruning linear dihedrals. Values are listed with using individual [average] equilibrium values of internal coordinates. This data is taken from calculations (after pruning and without bond–bond cross terms) described in ref. 19. For KEWZOD, the torsion potential for linear dihedrals included the single-linear dihedral but not the double-linear dihedral

MOF refcode	Validation *R*-squared with torsion potential for linear dihedrals	Validation *R*-squared using no torsion potential for linear dihedrals
BEPVID	0.9392 [0.9356]	0.9392 [0.9356]
GIWMOP	0.9209 [0.9044]	0.9210 [0.9044]
HECQUB	0.9002 [0.9002]	0.9002 [0.9002]
KEWZOD	0.9208 [0.9194]	0.9208 [0.9194]
XAHROQ	0.9385 [0.9377]	0.9386 [0.9377]

### 10.7 Torsions in chiral chain-like molecules

The rigid (or relaxed) torsion scans used in ADDT, CADT, ADCO, or CACO torsion mode smart selection (see Section 7.2 above and ref. 18) are intended to be used when the rotatable group on one side of the rotatable middle bond is small enough that it does not sterically collide with the surrounding structure when rigidly rotated. Typical examples of small rotatable groups include methyl (–CH_3_), hydroxyl (–OH), amino (–NH_2_), nitro (–NO_2_), carboxyl (–C(O)OH), carboxylate (–CO_2_^−^), *etc.* substituent groups.

Sometimes rotatable dihedrals are contained in long chains comprising polymers, large biomolecules (*e.g.*, proteins, DNA, RNA, enzymes, fatty acids, polysaccharides, phospholipids, *etc.*), hydrocarbon chains (*e.g.*, petroleum), *etc.* A chain-like molecule often has many different conformers that are local ground-state structures in which all atom-in-material forces are zero. (Each conformer is a local energy minimum on the molecule's potential energy surface.) For example, a specific protein chain can often fold in different ways to form many different conformers. For these chain-like molecules, a rigid torsion scan of the entire chain may not be appropriate, because it may cause one part of the molecule's chain to sterically collide with another part of the molecule's chain. In these cases, a different strategy besides torsion scans of the full chain should be used to construct the training dataset for optimizing the chain's torsion force constants.

Two basic strategies could be suggested for optimizing the torsion force constants of chain-like molecules. The first strategy uses short pieces of the chain to separately optimize the torsion force constants for each type of rotatable middle bond. Each snippet of the chain should be capped with appropriate atoms; for example, a cut C–C single bond could be turned into a C–H termination. This strategy uses model molecules that are small enough so that rigid torsion scans can be performed without encountering any steric clashes of the rotating group. The reference energy for each torsion scan geometry would be computed using a high-level quantum chemistry method. If the molecule is small enough, a QM-computed relaxed torsion scan could be performed (instead of a rigid torsion scan) without causing large changes (*i.e.*, large relaxations) in the unconstrained internal coordinate values (*i.e.*, bond lengths, angle values, and unconstrained other dihedral values). This rigid (or relaxed) torsion scan allows smart mode selection (see Section 7.2) to be used successfully. The model molecule should be large enough to capture the relevant chemical environment around the corresponding target middle bond.

The second strategy employs the entire full chain without invoking model molecules. One could generate a training set containing many chain configurations. For example, molecular dynamics or Monte Carlo simulations could be used to generate an ensemble containing many chain configurations. The energy of each chain configuration would then be computed using a high-level quantum chemistry method. These quantum-mechanically-computed energies would then be used in the training set used to optimize the ADDT, CADT, ADCO, CACO, and/or ADLD torsion force constants. Symmetry properties could be used to eliminate some of the unimportant torsion modes prior to force-constant optimization. For example, the 3-fold rotation symmetry of methyl groups implies corresponding torsion modes of the form cos[3*j*(*ϕ* − *ϕ*_eq_)] and sin[3*j*(*ϕ* − *ϕ*_eq_)] for ADDT or CADT model potentials or cos[3*jϕ*] for ADCO or CACO model potentials, where *j* is a whole number. Similarly, the 2-fold rotation symmetry of amino (–NH_2_), nitro (–NO_2_), and carboxylate (–CO_2_^−^) groups implies corresponding torsion modes of the form cos[2*j*(*ϕ* − *ϕ*_eq_)] and sin[2*j*(*ϕ* − *ϕ*_eq_)] for ADDT or CADT model potentials or cos[2*jϕ*] for ADCO or CACO model potentials, where *j* is a whole number.

Most biomolecules contain many chiral centers. Chiral centers lack mirror-image (reflection) symmetry.

Fig. 11 shows a dramatic failure of the cosine-only model potential for the relaxed and rigid torsion scan curves of the FCOH dihedral in the *S* enantiomer of the chiral molecule C(OH)ClFH. As shown in Fig. 8, my CADT and ADDT model potentials gave *R*-squared values of 0.9995 (relaxed torsion scan) and 0.9999 (rigid torsion scan) for this same dihedral.

**Fig. 17:**
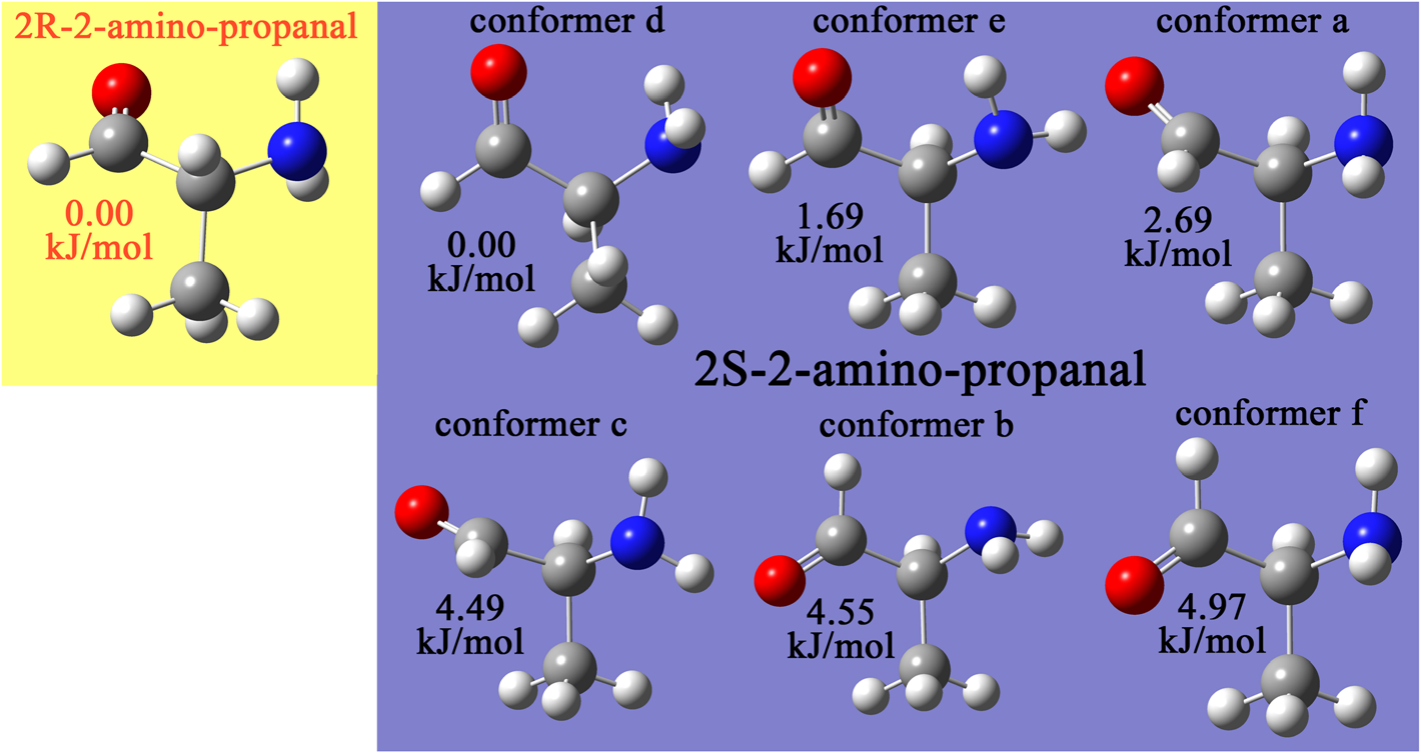
Various conformers of the 2S-2-amino-propanal molecule as computed using the B3LYP + D3BJ/def2-TZVPD level of theory. The computed relative energy (in kJ mol^−1^) is listed next to each conformer. For comparison, the lowest energy conformer of the 2*R* enantiomer is also shown.

As a more complex example, here we study the 2*S*-2-amino-propanal molecule. This molecule contains a chiral center. ‘2*S*’ indicates the specific enantiomer studied here. (The 2 in ‘2*S*’ indicates the chain position of the S chiral center.) Although this molecule only contains three rotatable middle bonds, it is still large enough to form many different conformers. The various conformers shown in Fig. 17 were generated by the following procedure. First, the three rotatable dihedrals (*i.e.*, OCCN, HNCC, and HCCH) were systematically rotated to a series of different values to generate a set of 24 starting structures. Beginning with each starting structure, geometry optimization was then performed in Gaussian 16 to compute equilibrium structures in which all atom-in-material forces are zero. I computed these using the B3LYP + D3BJ/def2-TZVPD (ref. 34 and 36–39) level of theory. Next, a frequency calculation was performed on each distinct equilibrium structure to identify whether it is a local ground state (*i.e.*, local energy minimum) or a saddle point (*e.g.*, transition state). Fig. 17 shows the resulting conformers that had all real-valued non-negative frequencies.

For comparison, Fig. 17 also shows the lowest energy conformer of the 2*R* enantiomer. The 2*R* enantiomer has the same number of conformers as the 2*S* enantiomer, such that each 2*R* conformer is a mirror image of and has the same energy as the corresponding 2*S* conformer.

**Fig. 18:**
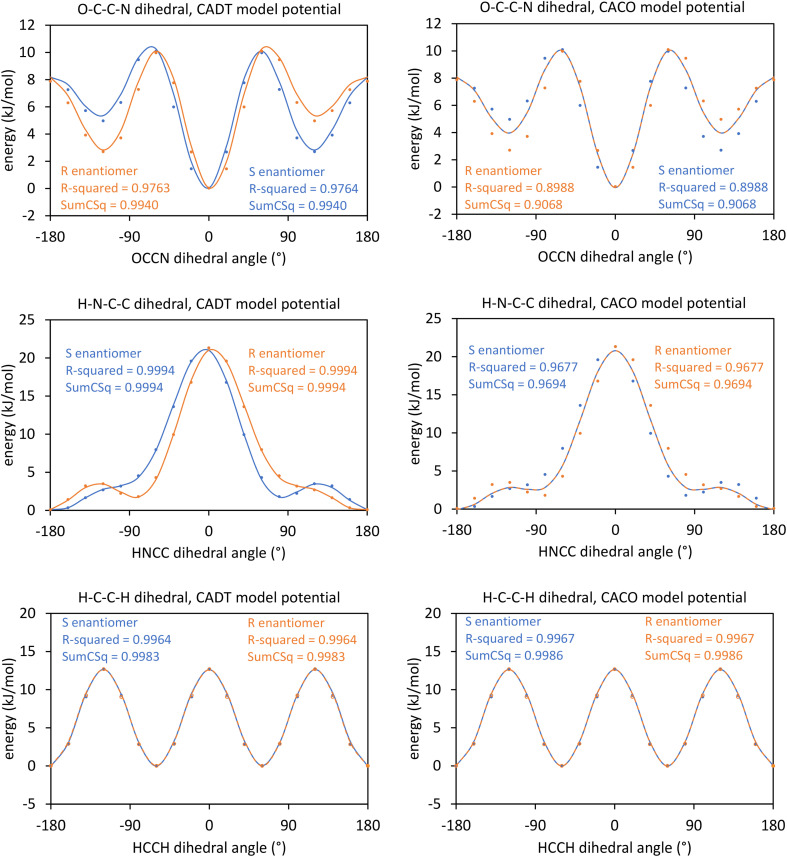
Better performance of the CADT model potential (left panels) compared to the CACO torsion model potential (right panels). Results are shown here for three rotatable dihedrals in the *R* (orange points and orange curves) and S (blue points and blue curves) enantiomers of the 2-amino-propanal molecule. The filled circles show the QM-computed (B3LYP + D3BJ/def2-TZVPD) energies for the dihedral scan curve in which all geometric parameters were relaxed except the constrained dihedral. The solid lines show the fitted model potentials including CADT modes 1–7 (left panels) and CACO modes 1–4 (right panels).

As shown in Fig. 18, the CADT model potential accurately reproduces the distinct mirror-image torsion scan curves for the *R* and *S* enantiomers. As described in Sections 3–5 above, both the ADDT and CADT model potentials achieve this in a way that allows mirror-image dihedral instances to be included within the same dihedral type. When using the ADDT and CADT model potentials, both the *R* and *S* enantiomers are described by the same dihedral types and torsion force constant values. In stark contrast, the cosine-only torsion model potential (*i.e.*, eqn (202) and (150)) cannot provide distinct mirror-image torsion scan curves for the *R* and *S* enantiomers. Depending on the specific situation, this limitation of the cosine-only torsion model potential (*i.e.*, eqn (202) and (150)) may result in any of the following: (a) negligible change in the *R*-squared value (*e.g.*, rotation of a methyl group as shown in the lower panels of Fig. 18), (b) a moderate reduction in the *R*-squared value (*e.g.*, rotation of the HNCC or OCCN dihedrals in 2-amino-propanal as shown in the middle and top panels of Fig. 18), or (c) a huge catastrophic reduction in the *R*-squared value (*e.g.*, rotation of the FCOH dihedral in the C(OH)ClFH molecule as shown in Fig. 11).

The computed *sym*_value (see eqn (159)) for these three dihedrals was 0.299 for the OCCN dihedral, 0.174 for the HNCC dihedral, and 0.014 for the HCCH dihedral. Using these *sym*_values, the equilibrium bond angle values, and the flowchart shown in Fig. 6, the CADT model potential is recommended for all three of these dihedrals. For the CADT model, the following modes were smart selected using the cutoff values recommended in Section 7.2: modes 3, 5, and 7 for the HCCH dihedral; modes 1, 2, 3, 5, 6, and 7 for the HNCC dihedral, and modes 1, 2, 3, 4, 5, and 6 for the OCCN dihedral. For the CACO model, the following modes were smart selected using the cutoff value of 0.001 recommended in Section 7.2: mode 3 for the HCCH dihedral; modes 1, 2, 3, and 4 for the HNCC dihedral, and modes 1, 2, 3, and 4 for the OCCN dihedral.

For this molecule, different sets of torsion force constants were optimized by fitting models to a training dataset. The training dataset contained the following QM-computed (B3LYP + D3BJ/def2-TZVPD) geometries and energies for the *S* enantiomer: (a) the six conformers shown in Fig. 17, (b) the symmetry-unique geometries for the relaxed torsion scan curves shown in Fig. 18 (*i.e.*, 18 geometries for OCCN dihedral, 18 geometries for HNCC dihedral, and 6 geometries for HCCH dihedral), and (c) 30 geometries in which the 3 dihedral values were chosen using a uniform random number generator. For (c), the geometries were relaxed in Gaussian 16 (using the B3LYP + D3BJ/def2-TZVPD level of theory) keeping the three randomized dihedral values constrained but allowing the bond lengths and angles to relax. This training dataset contained a total of 78 geometries.

Torsion force constants were optimized in Matlab by minimizing the following loss function:245
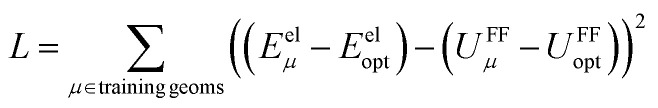

246 *U*^FF^_*μ*_ = *U*^bonded^_*μ*_ + *U*^nonbonded^_*μ*_

247 *U*^nonbonded^_*μ*_ = *U*^intracluster_nonbonded^_*μ*_ + *U*^intercluster_nonbonded^_*μ*_

where *μ* is the geometry number in the training dataset. *E*^el^_*μ*_ and *E*^el^_*opt*_ are the QM-computed electronic energies of training geometry *μ* and the fully optimized ground-state geometry, respectively. *U*^FF^_*μ*_ and *U*^FF^_opt_ are the forcefield model's potential energies of training geometry *μ* and the fully optimized ground-state geometry, respectively. Since the forcefield was trained on an isolated bonded cluster (*i.e.*, an isolated molecule), there were no intercluster interactions in the training dataset geometries: *U*^intercluster_nonbonded^_*μ*_ = 0.

Six variations of the forcefield model were constructed from two different torsion models and three different nonbonded interaction models: 2 (torsion models) × 3 (nonbonded interaction models) = 6 forcefield models. The two torsion models were: (a) the CADT model potential with smart selected modes and (b) the CACO torsion model potential using smart selected modes. Since the bond angles and bond lengths were relaxed in all training and validation geometries, dihedral torsions were the only bonded interactions included in the six forcefield models. This was chosen to directly compare the CADT model to the CACO torsion model.

The three nonbonded interaction models were: (i) no nonbonded interactions (*i.e.*, all nonbonded interactions set to zero), (ii) electrostatic and Lennard-Jones interactions between all atoms except first-, second-, and third-neighbors (*i.e.*, excluding self, 1-2, 1-3, and 1-4 nonbonded interactions), and (iv) electrostatic and Lennard-Jones interactions between all atoms except first- and second-neighbors (*i.e.*, excluding self, 1-2, and 1-3 nonbonded interactions). Nonbonded electrostatic interactions were modeled with an atom-centered point charge model computed using the DDEC6 (ref. 40 and 41) method. Lennard-Jones parameters and combining rules were taken from the Universal Force Field (UFF).^42^ These were used as inputs for Manz's interaction separation ansatz following the case in which a cutoff distance is not used for the nonbonded interactions.^20^ Following the notation of ref. 20, this gives:248
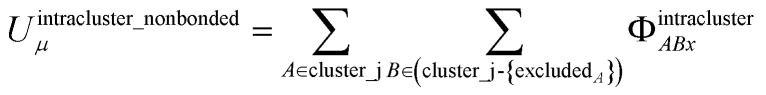
249
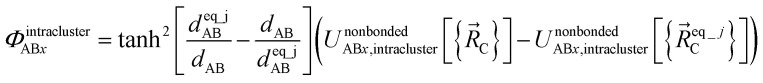
250
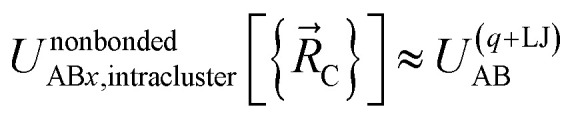
251
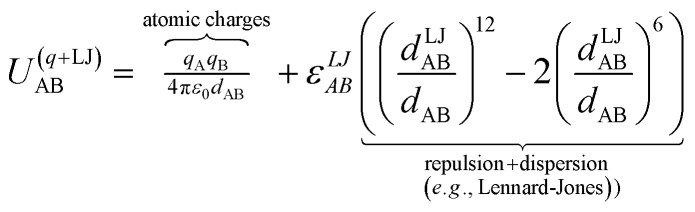
where 
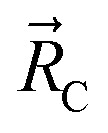
 is the nuclear position of atom C, cluster_j is the specific molecule, *d*^eq_j^_AB_ is the equilibrium distance between atoms A and B in the optimized ground state of isolated cluster_*j*, {excluded_A_} is the set of excluded nonbonded interactions (*e.g.*, self, 1–2, 1–3, and optionally 1–4 interactions), *ε*^LJ^_AB_ is the Lennard-Jones well-depth, and *d*^LJ^_AB_ is the Lennard-Jones reference distance.

The validation dataset contained 30 geometries in which the 3 dihedral values were chosen using a uniform random number generator. These geometries were relaxed in Gaussian 16 (using the B3LYP + D3BJ/def2-TZVPD level of theory) keeping the three randomized dihedral values constrained but allowing the bond lengths and angles to relax. These 30 validation geometries were generated independently (*i.e.*, using different random numbers) from the 30 randomized geometries used in the training dataset.

R-squared values were computed for the training and validation datasets using eqn (17) with the following definitions from ref. 20:252
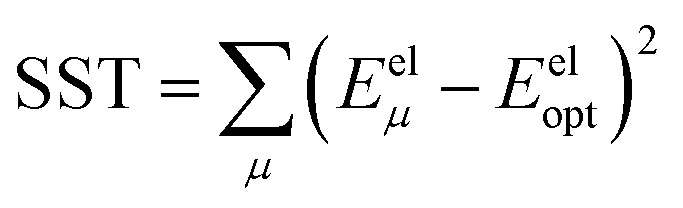
253
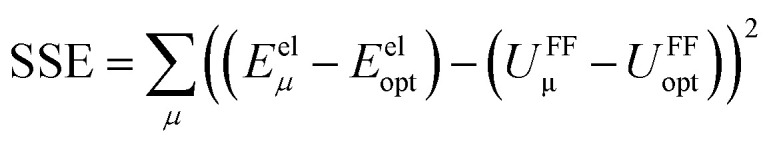


Minimizing the loss function in eqn (245) gives the same optimized force constant values as maximizing *R*-squared training. The root-mean-squared-error (RMSE) was defined as254
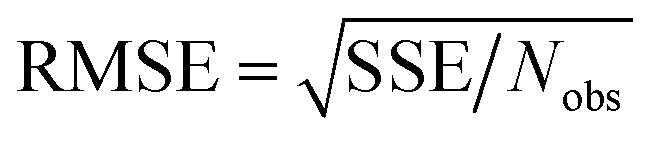
where *N*_obs_ is the total number of observation datapoints (*i.e.*, number of ‘*y* values’) in the (training or validation) dataset used to compute SSE. (In cases where the training or validation dataset contains atom-in-material forces and/or total energies, a total energy or atom-in-material force component counts as a ‘*y* value’.^18^)

Tables S3 and S4 of the ESI† list the optimized torsion force constant values. The Matlab codes and output results are found in the supporting data zip archive of the ESI.† As shown in Table 13, the CADT model potential performed better overall than the CACO torsion model potential for this molecule.

**Table 13:** Performance of six different forcefield models for describing dihedral torsions in the chiral 2-amino-propanal molecule

Torsion model	Nonbonded interactions included	*R*-squared training	RMSE training (eV)	*R*-squared validation	RMSE validation (eV)
CADT	None	(0.9424)^a^	(0.0326)^a^	(0.9227)^a^	(0.0651)^a^
CADT	Except 1-2, 1-3, 1-4	0.9317	0.0355	0.9444	0.0552
CADT	Except 1–2, 1–3	0.9259	0.0370	0.9427	0.0560
CACO	None	(0.9034)^a^	(0.0423)^a^	(0.9451)^a^	(0.0548)^a^
CACO	Except 1–2, 1–3, 1–4	0.7803	0.0637	0.8806	0.0809
CACO	Except 1–2, 1–3	0.8542	0.0519	0.9170	0.0674

a The results with all nonbonded interactions excluded are listed here for comparison purposes only. Since this molecule contains several 1–5 pairs (*i.e.*, fourth neighbors), it would actually not be appropriate to use a forcefield for this molecule that neglected all nonbonded interactions. Please see the main text for a discussion of this issue.

Although the two forcefield models that omitted all nonbonded interactions also appeared to perform well for this small molecule, the following point should be kept in mind. For long flexible chains, including nonbonded interactions is mandatory during classical molecular dynamics and Monte Carlo simulations. If all nonbonded interactions are excluded during such atomistic simulations, then different parts of a long flexible chain could occupy the same spatial local volume. This behavior would be physically unreasonable, because it violates the Pauli exclusion principle. To prevent such unphysical behavior, the forcefield must include short-range repulsion that causes nonbonded atoms to repel each other at short distances. For example, the Lennard-Jones potential includes a short-range repulsion that prevents two non-bonded atoms from occupying the same spatial local volume. Examining Table 13, for the forcefields including some nonbonded interactions, the CADT model performed significantly better than the CACO model for this molecule.

The same force constant values, *R*-squared values, and RMSE values would have resulted if some or all of the geometries in either the training or validation datasets were swapped for their mirror images. (The ADDT, ADCO, CADT, CACO, and ADLD model potentials are constructed to achieve this property.) Accordingly, changing *S* to *R* enantiomers by taking mirror image geometries would not have altered any of the results. Of course, for those mirror-image geometries, one is required to use the correct dihedral equilibrium value (*ϕ*_eq_) for each corresponding dihedral instance in that enantiomer. Taking a mirror image (*e.g.*, changing *S* to *R* enantiomer) has the following effect:

255 *ϕ*^eq^_ABCD_[*R* enantiomer] = π − mod[(*ϕ*^eq^_ABCD_[*S* enantiomer] + π),2π]

256 *ϕ*_ABCD_[*R* enantiomer] = π − mod[(*ϕ*_ABCD_[*S* enantiomer] + π),2π]

In eqn (255) and (256), the mod function ensures that −π < *ϕ*_ABCD_[*R* enantiomer] ≤ π.

### 10.8 Comparison of vibrational frequencies computed from forcefield models to experimental data

Forcefields were optimized for the isocyanic acid (HNCO), hydrogen peroxide (HOOH), and acetylene (HCCH) molecules. These molecules were chosen, because they allow each of the five dihedral torsion model potentials (*i.e.*, ADCO, ADDT, CACO, CADT, and ADLD) to be tested, and the computed vibrational frequencies were compared to experimental data. No cross terms were included in these forcefields, because computational tests showed they were not required to achieve good performance. The small size of these molecules greatly simplified the forcefield parameterization and testing. Since these molecules contained no further than third-neighbors, all intracluster nonbonded interactions were excluded.

For simplicity, the harmonic bond stretch potential was used:

257 *U*^harmonic_stretch^_AB_ = ½*k*_AB_(*d*_AB_ − *d*^eq^_AB_)^2^

Prior work showed the Manz stretch potential better models bond stretch anharmonicity.^20^ However, this involves an additional non-empirical parameter (*i.e.*, the quantum-mechanically-computed exponent 
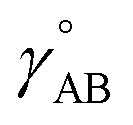
).^20^ Since the normal mode frequencies were computed here within the harmonic oscillator approximation which neglects bond stretch anharmonicities, the simpler harmonic bond stretch potential was used in these optimized forcefields.

Vibrational frequencies were computed using the procedure described in a prior publication: “For each flexibility model, normal vibrational mode analysis within the harmonic oscillator approximation was performed by diagonalizing the mass-weighted Hessian (MWH) matrix expressed in Cartesian coordinates:258
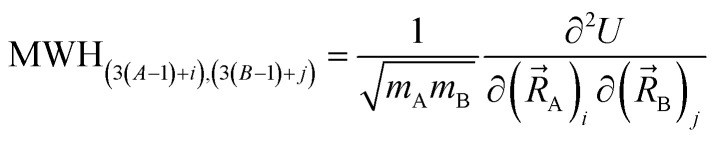
where *m*_A_ is the mass of atom A. Here, 
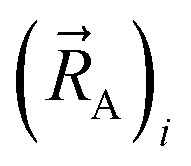
 for *i* ∈ {1,2,3} denotes the *X*, *Y*, or *Z* component of the nuclear position 
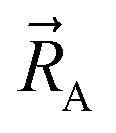
. The second derivatives can be computed either analytically or numerically; here, they were computed numerically using the central finite difference approximation. The eigenvalues {*λ*_*i*_} of the MWH matrix are related to the normal mode frequencies {freq_*i*_} *via*:^43^259
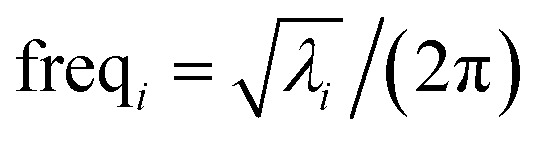


Each normal mode frequency was converted to wavenumber by dividing by the speed of light, *c*. Each eigenvector 
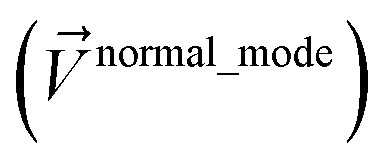
 of the MWH matrix is the corresponding normal mode's mass-weighted differential displacement vector:260
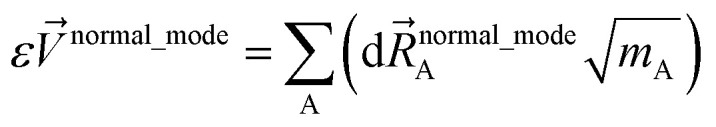
for infinitesimal |*ε*|.

For linear molecules, five of the MWH eigenvalues are zero; these correspond to molecular rotation (2 modes) and center-of-mass translation (3 modes). For nonlinear molecules, six of the MWH eigenvalues are zero; these correspond to molecular rotation (3 modes) and center-of-mass translation (3 modes).”^20^

For HNCO, the training dataset included 145 geometries: (a) the optimized ground-state geometry (1 geometry), (b) a rigid torsion scan in 20° increments over the range 180° < *ϕ* ≤ 180° (17 geometries outside the optimized geometry), (c) a partly relaxed torsion scan (in which bond angles were constrained but bond lengths were relaxed) in 20° increments over the range 180° < *ϕ* ≤ 180° (17 geometries outside the optimized geometry), (d) all possible combinations of bond lengths being displaced by −0.05, 0.00, and/or +0.05 Å (26 geometries outside the optimized geometry), (e) a rigid HNC angle scan in 5° increments over the range 30° ≤ Δ*θ*_HNC_ ≤ 30° (12 geometries outside the optimized geometry), (f) a relaxed HNC angle scan in 5° increments over the range 30° ≤ Δ*θ*_HNC_ ≤ 30° (12 geometries outside the optimized geometry), (g) a rigid NCO angle scan in 2° increments over the range 150° ≤ *θ*_NCO_ ≤ 180° (16 geometries), (h) a relaxed NCO angle scan in 2° increments over the range 150° ≤ *θ*_NCO_ ≤ 164° (8 geometries), (i) a torsion scan in 20° increments over the range 180° < *ϕ* ≤ 180° in which *θ*_NCO_ was constrained to 155° while all other geometric parameters were relaxed (18 geometries), and (j) a torsion scan in 20° increments over the range 180° < *ϕ* ≤ 180° in which *θ*_NCO_ was constrained to 165° while all other geometric parameters were relaxed (18 geometries).

In this subsection, the force constants were optimized using least-squares fitting in Matlab to minimize the cost function shown in eqn (245), where the forcefield contained no intracluster nonbonded interactions as explained above. The training *R*-squared and RMSE values were computed using eqn (17) and (252)–(254).

Table 14 summarizes the optimized force constants and training dataset statistics for the HNCO molecule using forcefields with ADCO or ADDT dihedral torsion model potentials. A bend potential for the NCO angle is not needed and was not used, because this angle scan curve is completely described by the HNCO torsion offset potential as clearly shown in Fig. 9. For this molecule, the QM-computed dihedral torsion potential energy surface contains no odd-function contributions (*i.e.*, *U*[*ϕ*] = *U*[−*ϕ*]). For ADCO and ADDT, the smart mode selection criteria described in Section 7 above were used. As shown in Table 14, the forcefields using the ADCO and ADDT model potentials performed extremely well and yielded nearly identical results for this molecule.

**Table 14:** Training dataset performance for isocyanic acid (HNCO) forcefields using harmonic bond stretch, Manz angle-bend, and ADCO or ADDT dihedral torsion model potentials. The QM-computed (CCSD/def2-TZVPD) equilibrium values were 1.00578 Å (HN), 1.21144 Å (NC), 1.16029 Å (CO), 123.57915° (HNC), 172.98777° (NCO), and 180.000° (HNCO)

	*k*_stretch_ (eV Å^−2^)	*k*_bend_ (eV)	*k*_torsion_ (eV)	Training RMSE (eV)	Training *R*-squared	*ϕ*_opt_ (°)
Forcefield *w*. ADCO	45.10 (HN), 56.60 (NC), 104.46 (CO)	1.696 (HNC)	0.05818, (*c*_*i*_ = [0.999999, 0.001117, 0.000000, 0.000000])	0.0283	0.9880	180.000°
Forcefield *w*. ADDT	45.10 (HN), 56.61 (NC), 104.46 (CO)	1.696 (HNC)	0.05817 (mode 1)	0.0283	0.9881	180.000°

Table 15 summarizes the validation dataset results for this molecule. The vibrational frequencies computed using each forcefield were in good agreement with the experimentally-measured frequencies.

**Table 15:** Validation dataset performance for isocyanic acid (HNCO) forcefields using harmonic bond stretch, Manz angle-bend, and ADCO or ADDT dihedral torsion model potentials. This validation dataset compares the normal-mode vibrational frequencies computed from the forcefield to experimentally-measured frequencies (in wavenumber, cm^−1^). The percent error relative to experiment is shown in parentheses. Modes 4 and 6 are two different in-plane bends, and each of these modes involves changes in both the HNC and CNO angles

freq→	1 (HN stretch)	2 (asymmetric NCO stretch)	3 (symmetric NCO stretch)	4 (in-plane bend)	5 (torsion)	6 (in-plane bend)
Experiment (ref. 44 and 45)	3538	2269	1322	777	656	577
Forcefield *w*. ADCO	3616 (+2%)	2279 (0%)	1160 (−12%)	789 (+2%)	574 (−13%)	542 (−6%)
Forcefield *w*. ADDT	3616 (+2%)	2279 (0%)	1161 (−12%)	789 (+2%)	574 (−13%)	542 (−6%)

For HOOH, the training dataset included 132 geometries: (a) the optimized ground-state geometry and its mirror image (2 geometries), (b) a rigid torsion scan in 20° increments over the range 180° < *ϕ* ≤ 180° (18 geometries), (c) a relaxed torsion scan in 20° increments over the range 180° < *ϕ* ≤ 180° (18 geometries), (d) a torsion scan in 20° increments over the range 180° < *ϕ* ≤ 180° in which Δ*θ*_HOO_ was constrained to −10° while all other geometric parameters were relaxed (18 geometries), (e) a torsion scan in 20° increments over the range 180° < *ϕ* ≤ 180° in which Δ*θ*_HOO_ was constrained to +10° while all other geometric parameters were relaxed (18 geometries), (f) all symmetry-unique combinations of bond lengths being displaced by −0.14, 0.00, and/or +0.14 Å (17 geometries outside the optimized geometry), (g) mirror images of the structures from (*f*) (17 geometries), (h) a rigid HOO angle scan in 10° increments over the range 80° ≤ *θ*_NCO_ ≤ 130° (6 geometries), (i) a relaxed HOO angle scan in 10° increments over the range 80° ≤ *θ*_NCO_ ≤ 130° (6 geometries), and (j) mirror images of the structures from (*h*) and (i) (12 geometries).

Table 16 summarizes the optimized force constants and training dataset statistics for the HOOH molecule using forcefields with CACO or CADT dihedral torsion model potentials. For this molecule, the QM-computed dihedral torsion potential energy surface contains no odd-function contributions (*i.e.*, *U*[*ϕ*] = *U*[−*ϕ*]). For CACO and CADT, the smart mode selection criteria described in Section 7 above were used. Except for one key distinction, the forcefields using CACO and CADT model potentials yielded similar results. The key distinction is that CACO strictly yielded *U*[*ϕ*] = *U*[−*ϕ*] giving *ϕ*_opt_ = ±111.47° which is close to but not identical to the QM-computed value of ±111.0568°. On the other hand, CADT reproduced *ϕ*_opt_ = +111.0568° on the positive side but yielded a slightly asymmetric potential *U*[*ϕ*] ≅ *U*[−*ϕ*] so that on the negative side *ϕ*_opt_ was −104.71° instead of −111.0568°. Both forcefields yielded training R-squared > 0.95.

**Table 16:** Training dataset performance for hydrogen peroxide (HOOH) forcefields using harmonic bond stretch, Manz angle-bend, and CACO or CADT dihedral torsion model potentials. The QM-computed (CCSD/def2-TZVPD) equilibrium values were 0.9666 Å (HO), 1.4378 Å (OO), 100.8215° (HOO), and 111.0568° (HOOH)

	*k*_stretch_ (eV Å^−2^)	*k*_bend_ (eV)	*k*_torsion_ (eV)	Training RMSE (eV)	Training *R*-squared	*ϕ*_opt_ (°)
Forcefield *w*. CACO	50.71 (HO), 31.81 (OO)	5.683 (HOO)	0.1552, (*c*_*i*_ = [0.833919, 0.549530, 0.050017, 0.009368])	0.1194	0.9530	±111.47
Forcefield *w*. CADT	50.96 (HO), 31.85 (OO)	5.760 (HOO)	0.04466 (mode 1), 0.06460 (mode 2), −0.00888 (mode 3), −0.11657 (mode 5), 0.04935 (mode 6), −0.05040 (mode 7)	0.1192	0.9531	−104.71, +111.0568

Table 17 summarizes the validation dataset results for this molecule. The vibrational frequencies computed using each forcefield were in good agreement with the experimentally-measured frequencies. For this molecule, the experimental reference frequency for each vibrational mode was taken as the median of the experimental values compiled from various sources as listed in the NIST Chemistry Webbook.^46^ Please see the ESI† for calculation of these median experimental values.

**Table 17:** Validation dataset performance for hydrogen peroxide (HOOH) forcefields using harmonic bond stretch, Manz angle-bend, and CACO or CADT dihedral torsion model potentials. This validation dataset compares the normal-mode vibrational frequencies computed from the forcefield to experimentally-measured frequencies (in wavenumber, cm^−1^). The percent error relative to experiment is shown in parentheses

freq→	1 (asymmetric OH stretch)	2 (symmetric OH stretch)	3 (symmetric HOO bend)	4 (asymmetric HOO bend)	5 (OO stretch)	6 (torsion)
Experiment	3613	3587	1389	1271	869	371
Forcefield *w*. CACO	3815 (+6%)	3814 (+6%)	1421 (+2%)	1349 (+6%)	981 (+13%)	388 (+5%)
Forcefield *w*. CADT	3824 (+6%)	3823 (+7%)	1430 (+3%)	1358 (+7%)	982 (+13%)	378 (+2%)

For HCCH, the training dataset included 78 geometries: (a) the optimized ground-state geometry (1 geometry), (b) all symmetry-unique combinations of bond lengths being displaced by −0.14, 0.00, and/or +0.14 Å (17 geometries outside the optimized geometry), (c) torsion scans in 20° increments over the range 0 ≤ *ϕ* ≤ 180° in which each HCC angle was constrained to 150, 160, or 170° while all other geometric parameters were relaxed (60 geometries). For (c), the range −180° < *ϕ* < 0 was not required, because due to symmetry *U*[*ϕ*] = *U*[−*ϕ*]. For (c), due to the molecular symmetry, there were six pairs of symmetry-unique constrained bond angles: (*θ*_1_, *θ*_2_) = (150°, 150°), (160°, 150°), (170°, 150°), (160°, 160°), (170°, 160°), and (170°, 170°).

Table 18 summarizes the optimized force constants and training dataset statistics for the acetylene molecule using a forcefield with the ADLD model potential. Since this molecule contains a double-linear dihedral, the *n* = 2(*j* = 1) − 1 = 1 modes form the dominant contribution. Due to the mirror plane, the sine torsion modes do not contribute, and thus *k*^1^_LD3_ = *k*^1^_LD6_ = 0. As shown in Table 11, for this molecule *k*^1^_LD5_ > 0 is significant while *k*^1^_LD1_ = *k*^1^_LD2_ = *k*^1^_LD4_ = 0. Consequently, *k*^1^_LD5_ was the only ADLD mode included in the parameterized forcefield. This forcefield yielded training *R*-squared > 0.95.

**Table 18:** Training dataset performance for acetylene forcefields using harmonic bond stretch, Manz angle-bend, and ADLD torsion model potentials. The QM-computed (CCSD/def2-TZVPD) equilibrium values were 1.06358 Å (HC), 1.20183 Å (CC), and 180.000° (HCC)

	*k*_stretch_ (eV Å^−2^)	*k*_bend_ (eV)	*k*_torsion_ (eV)	Training RMSE (eV)	Training *R*-squared
Forcefield *w*. ADLD	41.91 (HC), 111.34 (CC)	1.045 (HCC)	3.400 (*k*^1^_LD5_)	0.1307	0.9681

Table 19 summarizes the validation dataset results for this molecule. For both HCCH and DCCD, the vibrational frequencies computed using the forcefield were in excellent agreement with the experimentally-measured frequencies. Both HCCH and DCCD used the same forcefield parameters from Table 18. The change in frequencies from HCCH to DCCD arose solely due to the different masses of D *versus* H atoms.

**Table 19:** Validation dataset performance for acetylene (HCCH) and deuterated acetylene (DCCD) forcefields using harmonic bond stretch, Manz angle-bend, and ADLD torsion model potentials. This validation dataset compares the normal-mode vibrational frequencies computed from the forcefield to experimentally-measured frequencies (in wavenumber, cm^−1^). The percent error relative to experiment is shown in parentheses. Due to the molecular symmetry, mode 5 has the same frequency as mode 4, and mode 7 has the same frequency as mode 6

Molecule	freq→	1 (symmetric CH stretch)	2 (asymmetric CH stretch)	3 (CC stretch)	4, 5 (bowl)	6, 7 (wave)
HCCH	Experiment (ref. 47)	3372	3295	1974	730	599
HCCH	Forcefield *w*. ADLD	3588 (+6%)	3501 (+6%)	2106 (+7%)	701 (−4%)	641 (+7%)
DCCD	Experiment (ref. 47)	2704	2431	1766	538	504
DCCD	Forcefield *w*. ADLD	2855 (+6%)	2571 (+6%)	1872 (+6%)	534 (−1%)	515 (+2%)

## 11. Conclusions

Dihedral torsion model potentials can be categorized into five classes. Class A (aka ‘dihedral-only’) torsion potentials depend exclusively on the dihedral value (*e.g.*, *ϕ*_ABCD_) with no explicit dependence on the bond lengths or bond angles. Class B (aka ‘angle-damped’) torsion potentials depend exclusively on the dihedral value (*e.g.*, *ϕ*_ABCD_) and the two contained bond angle values (*i.e.*, *θ*_ABC_ and *θ*_BCD_) with no explicit dependence on the bond lengths. Class C (aka ‘distance-damped’) torsion potentials depend exclusively on the dihedral value (*e.g.*, *ϕ*_ABCD_) and the three contained bond lengths (*i.e.*, *R*_AB_, *R*_BC_, and *R*_CD_) with no explicit dependence on the bond angles. Class D (aka ‘fully-damped’) torsion potentials depend exclusively on the dihedral value (*e.g.*, *ϕ*_ABCD_), the two contained bond angle values (*i.e.*, *θ*_ABC_ and *θ*_BCD_), and the three contained bond lengths (*i.e.*, *R*_AB_, *R*_BC_, and *R*_CD_). Class E contains all of the miscellaneous torsion potentials that do not fit into any of the first four classes.

The most important new developments in this article pertain to the Class B (‘angle-damped’) dihedral torsion potentials. First, the combined angle-dihedral coordinate branch equivalency conditions and mathematical constraints were derived and used to construct a model of the angle-damping factors. Second, using the concept of ‘completing the squares’ these angle-damping factors were used to construct series expansions defining the ADDT, ADCO, and ADLD model potentials. These new dihedral torsion model potentials require only a small number of terms to achieve excellent accuracy, high computational efficiency, and continuous derivatives of all orders with respect to atom-in-material displacements. They capture correct dynamics across a wide range of bond angles including the limiting value of *θ* = π. In contrast, most previously used dihedral–torsion model potentials have either a derivative discontinuity or incorrect dynamics when the bond angle reaches *θ* = π.

To properly resolve the derivative discontinuity at *θ* = π, I showed the torsion term must depend on both the bond angles and dihedral value. Of particular interest, this gives rise to a new torsion-derived angle-bending energy term called the torsion offset potential (TOP). I showed the TOP gives rise in some materials to the unusual physical phenomenon of slip torsion.

The CADT and CACO torsion model potentials apply only to torsions for which it is energetically inaccessible for any contained bond angle to reach *θ* = π. Because neither contained bond angle can energetically reach *θ* = π, this allows the derivative continuity condition for *θ* = π to be relaxed for such torsions. These two Class A torsion model potentials approximate the torsion barrier height as constant as the contained bond angles change values. As a less computationally expensive and simpler option compared to the ADDT and ADCO model potentials, the CADT and CACO model potentials can be used when the equilibrium values of both contained bond angles are <130°.

I derived a new orthonormal representation of the independent rotatable torsion modes that facilitates automated identification of which particular torsion modes contribute significantly for each dihedral type. This smart selection enables insignificant torsion modes to be excluded from the subsequent forcefield parameterization process. This makes the parameterized forcefields both compact and accurate.

These torsion model potentials cover the following situations:

(1) The new angle-damped dihedral torsion (ADDT) model potential is preferred when neither contained equilibrium bond angle is linear (*i.e.*, (*θ*^eq^_ABC_ and *θ*^eq^_BCD_) ≠ 180°), at least one of the contained equilibrium bond angles is ≥ 130° (*i.e.*, (*θ*^eq^_ABC_ or *θ*^eq^_BCD_) ≥ 130°), and the dihedral torsion potential contains some odd-function contributions (*i.e.*, *U*[*ϕ*] ≠ *U*[−*ϕ*]).

(2) The new angle-damped cosine only (ADCO) model potential is preferred when neither contained equilibrium bond angle is linear (*i.e.*, (*θ*^eq^_ABC_ and *θ*^eq^_BCD_) ≠ 180°), at least one of the contained equilibrium bond angles is ≥ 130° (*i.e.*, (*θ*^eq^_ABC_ or *θ*^eq^_BCD_) ≥ 130°), and the dihedral torsion potential contains no odd-function contributions (*i.e.*, *U*[*ϕ*] = *U*[−*ϕ*]).

(3) The new constant amplitude dihedral torsion (CADT) model potential is preferred when neither contained equilibrium bond angle is linear (*i.e.*, (*θ*^eq^_ABC_ and *θ*^eq^_BCD_) ≠ 180°), both contained equilibrium bond angles are < 130° (*i.e.*, (*θ*^eq^_ABC_ and *θ*^eq^_BCD_) < 130°), and the dihedral torsion potential contains some odd-function contributions (*i.e.*, *U*[*ϕ*] ≠ *U*[−*ϕ*]).

(4) The constant amplitude cosine only (CACO) model potential is preferred when neither contained equilibrium bond angle is linear (*i.e.*, (*θ*^eq^_ABC_ and *θ*^eq^_BCD_) ≠ 180°), both contained equilibrium bond angles are <130° (*i.e.*, (*θ*^eq^_ABC_ and *θ*^eq^_BCD_) < 130°), and the dihedral torsion potential contains no odd-function contributions (*i.e.*, *U*[*ϕ*] = *U*[−*ϕ*]).

(5) The new angle-damped linear dihedral (ADLD) model potential is preferred when at least one contained equilibrium bond angle is linear (*i.e.*, (*θ*^eq^_ABC_ or *θ*^eq^_BCD_) = 180°).

The combination of dihedral pruning;^18,19^ classifying each dihedral type as non-rotatable, rotatable, hindered, or linear;^18,19^ selecting a ADDT, CADT, ADCO, CACO, or ADLD model potential for each dihedral type; and torsion mode smart selection results in an extremely computationally efficient, accurate, numerically stable, and versatile treatment of dihedral torsion in classical forcefields.

Analytic first derivatives and forces associated with the ADDT, CADT, ADCO, CACO, and ADLD potentials are derived in the ESI.† These analytic formulas are useful for computing forces during classical molecular dynamics simulations. As summarized in Section S11 of the ESI,† these analytic derivative and force formulas were rigorously checked *via* extensive comparisons to values computed numerically using the central finite-difference approximation. The ESI† zip archive contains software code that implements these analytic derivative and force formulas and compares them to values computed numerically using the central finite-difference approximation.

In Section 10, these five torsion model potentials were extensively tested for selected molecules and compared to high-level quantum chemistry calculations or experimental data. In Section 10.2, detailed torsion modal analysis using the ADDT and CADT model potentials was performed for 13 molecules that have two nonlinear equilibrium bond angles: (CHFCl)_2_, C(OH)ClFH, ethane, FSSF, glyoxal, H_2_O_2_, HNCO, HNCS, HONC, HSNC, IF_3_ClOH, N_2_O_2_, and PF_4_OH. Torsion modal analysis was performed for all of these molecules using rigid bonds and angles, and for some of these molecules also using relaxed bonds and angles. As shown in Table 3 and Fig. 8, for a torsion model containing only the first seven ADDT/CADT orthonormal modes, the model showed superb fit (R-squared > 0.93) to the QM-computed CCSD/def2-TZVPD results.

For 
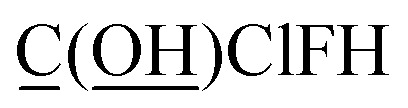
, 
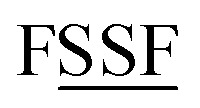
, 
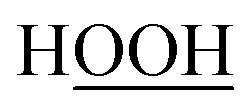
, 
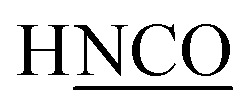
, 
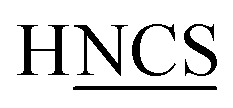
, 
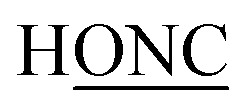
, 
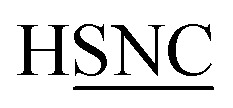
, 
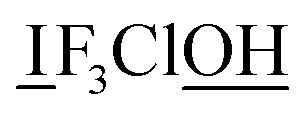
, 
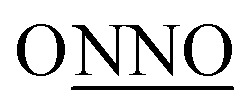
, and 
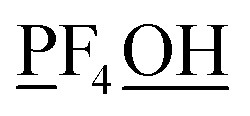
, angle-bending curves were QM-computed and compared in Fig. 9 to the sum of the Manz angle-bending potential plus the new torsion offset potential and found to be in excellent agreement. For 
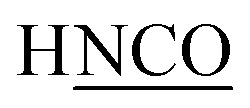
, 
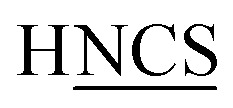
, 
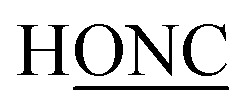
, 
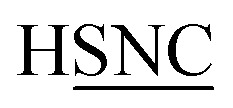
, excellent agreement was achieved without using any adjustable parameters. For the other six molecules, excellent agreement was achieved using only one adjustable parameter, *k*_angle_, which is the angle-bending force constant. The other parameters, which were not adjusted when preparing these angle-bending curves, were taken directly from the torsion modal analysis (see Table 3) for each molecule.

For HNCO, HNCS, HONC, HSNC, C(OH)ClFH, FSSF, H_2_O_2_, IF_3_ClOH, N_2_O_2_, and PF_4_OH, torsion modal analysis was performed at different constrained bond angles and compared to predictions of the ADDT and CADT model potentials. As summarized in Table 7, the ADDT model potential is a more accurate predictor of the torsion barrier height than the CADT model potential when the equilibrium value of at least one of the contained bond angles is ≥130°. In addition to predicting changes in the torsion barrier height, ADDT also predicts changes in the modal coefficients (see Fig. 10) and *ϕ*_min_ as the constrained bond angle varies. When the equilibrium values of both contained bond angles are <130°, the ADDT model has similar overall accuracy to the CADT model; however, the CADT model is preferred in this case due to its greater simplicity.

Section 10.3 performs an analogous torsion modal analysis using the ADCO and CACO model potentials for those molecules containing no odd-function contributions (*i.e.*, *U*[*ϕ*] = *U*[−*ϕ*] which makes *sym*_value = 0). The ADCO and CACO model potentials provided results similar to (but not necessarily identical to) the ADDT and CADT model potentials for these systems. As summarized in Table 10, the ADCO model potential is a more accurate predictor of the torsion barrier height than the CACO model potential when the equilibrium value of at least one of the contained bond angles is ≥130°. Section 10.3 also demonstrated (see Fig. 11) that the cosine-only model torsion potential fails when *sym*_value is substantially larger than zero.

Section 10.4 proves the torsion offset potential is indispensable. Specifically, the CCSD-computed potential energy surface for the HNCO molecule can only be reproduced when the torsion offset potential is included. This molecule exhibits slip torsion.

Section 10.5 analyzed in detail three molecules (*i.e.*, H_3_CCN, H_2_BCN, and H_2_BNC) having single-linear dihedrals plus two molecules (*i.e.*, HCCH and HCNO) having double-linear dihedrals. For each of these five molecules, the Manz bend and ADLD force constants were optimized to a training dataset containing the optimized geometry plus numerous geometries having constrained bond angle(s) and/or constrained dihedral values. In all cases, the training *R*-squared was >0.995, and this indicates superb model performance. The CCSD-computed and ADLD model potential energy surfaces were nearly identical.

Section 10.6 briefly recaps flexibility model performance for five MOFs containing linear dihedrals as described in a companion article.^18,19^ Since the torsion barrier is zero when the bond angle is linear, excellent performance was obtained even when the linear dihedral torsions were omitted from the flexibility models.

Section 10.7 studies a chiral molecule containing multiple adjacent rotatable dihedrals leading to many local ground-state conformations. This type of example was included to examine coupling between multiple adjacent rotatable dihedrals. Also, this molecule is large enough to examine the effects of different intracluster nonbonded interaction models. Using Manz's bonded/nonbonded interaction separation ansatz,^20^ the training and validation R-squared values depended only weakly on the specific choice of intracluster nonbonded interaction model. Comparisons were also made between the CADT and a cosine-only torsion model potential. Some of the prior literature used a cosine-only torsion model potential even for molecules where it does not strictly apply because *U*[*ϕ*] ≠ *U*[−*ϕ*] which makes *sym*_value > 0. Although this approximation sometimes yields reasonable results, I recommend that cosine-only torsion potentials such as ADCO and CACO be used only when *U*[*ϕ*] = *U*[−*ϕ*] which makes *sym*_value = 0. The ADDT and CADT model potentials are preferred when *U*[*ϕ*] ≠ *U*[−*ϕ*]. A key advantage of the ADDT and CADT model potentials is that a single set of force constant values simultaneously describes both mirror images (*e.g.*, enantiomers) of a chiral center. This was clearly demonstrated for the *S* and *R* enantiomers of the chiral 2-amino-propanal molecule.

Section 10.8 compared vibrational frequencies computed from forcefield models to experimental data for the HNCO, HOOH, and HCCH molecules. These molecules were chosen to provide examples including the ADCO, ADDT, CACO, CADT, and ADLD torsion potentials. Full flexibility models including bond stretches, angle bends, and dihedral torsions were parameterized. For each molecule, the training dataset *R*-squared was >0.95. Vibrational frequencies computed from the parameterized forcefields using the harmonic oscillator approximation were in excellent agreement with the previously published experimentally-measured vibrational frequencies.

In summary, all of these computational tests showed that the new theory of angle-damped dihedral torsion introduced in this article is a remarkable success.

## Data availability

Optimized geometries of molecules, selected data analysis spreadsheets, and selected Matlab codes and results are included as part of the ESI.†

## Conflicts of interest

There are no conflicts of interest to declare.

## Supplementary Material

RA-015-D4RA08960J-s001

RA-015-D4RA08960J-s002
