# Supplementary material for: Dihedral–torsion model potentials that include angle-damping factors
Source: RSC Adv. 2025 Mar 7;15(10):7257–306. doi: 10.1039/d4ra08960j (PMC11886958; doi:10.1039/d4ra08960j)
Supplement: RA-015-D4RA08960J-s002 [file RA-015-D4RA08960J-s002.pdf]

Supplementary Information (SI) for RSC Advances.
This journal is © The Royal Society of Chemistry 2025

Electronic Supplementary Information for

Dihedral-Torsion Model Potentials that Include Angle-Damping Factors

Thomas A. Manz

Chemical & Materials Engineering, New Mexico State University, Las Cruces, NM 88001
Email: tmanz@nmsu.edu

Contents

1. Gradients of the kangal

2. Gradients of the directed dihedral

3. Derivatives of the angle-damping factors

4. Analytic formulas for first derivatives and forces of the CADT potential

5. Analytic formulas for first derivatives and forces of the CACO potential

6. Analytic formulas for first derivatives and forces of the ADDT potential

7. Analytic formulas for first derivatives and forces of the ADCO potential

8. Selectivity rule for single-linear dihedrals

9. Analytic formulas for first derivatives and forces of the ADLD potential

10. Supplementary tables and supplementary figure

11. Validation of analytic derivative and force formulas

12. Derivation of torsion scan R-squared formulae

S1. Gradients of the kangal

I derived gradients of the kangal by first noting

$$\vec{\nabla}_G ж_{ABC} = \vec{\nabla}_G \cos\left[\theta_{ABC}/2\right] = \vec{\nabla}_G \sqrt{\frac{1+\cos\left[\theta_{ABC}\right]}{2}}$$
$$= \left(\frac{1}{4\sqrt{\frac{1+\cos\left[\theta_{ABC}\right]}{2}}}\right)\vec{\nabla}_G \cos\left[\theta_{ABC}\right] = \frac{1}{4ж_{ABC}}\vec{\nabla}_G \cos\left[\theta_{ABC}\right] \tag{S1}$$

$$\cos\left[\theta_{ABC}\right] = \hat{R}_{BA} \bullet \hat{R}_{BC} = 2\left(ж_{ABC}\right)^2 - 1 \tag{S2}$$

Then, I then substituted the formulas for $\vec{\nabla}_G \cos\left[\theta_{ABC}\right]$ from ref S1 into eqn (S1) to obtain

$$\vec{\nabla}_A ж_{ABC} = \frac{\left(\hat{R}_{BC} - \left(\hat{R}_{BA} \bullet \hat{R}_{BC}\right)\hat{R}_{BA}\right)}{4ж_{ABC} R_{BA}} = \frac{\left(\hat{R}_{BC} + \left(2\left(ж_{ABC}\right)^2 - 1\right)\hat{R}_{AB}\right)}{4ж_{ABC} R_{BA}} \tag{S3}$$

$$\vec{\nabla}_C ж_{ABC} = \frac{-\left(\hat{R}_{AB} + \left(2\left(ж_{ABC}\right)^2 - 1\right)\hat{R}_{BC}\right)}{4ж_{ABC} R_{BC}} \tag{S4}$$

$$\vec{\nabla}_B ж_{ABC} = -\left(\vec{\nabla}_A ж_{ABC} + \vec{\nabla}_C ж_{ABC}\right) \tag{S5}$$

which are expressed using the bond vector convention shown in eqn (S9)–(S11) above.

The analogous formulas for kangal BCD are:

$$\vec{\nabla}_B ж_{BCD} = \frac{\left(\hat{R}_{CD} - \left(\hat{R}_{CB} \bullet \hat{R}_{CD}\right)\hat{R}_{CB}\right)}{4ж_{BCD} R_{CB}} = \frac{\left(\hat{R}_{CD} + \left(2\left(ж_{BCD}\right)^2 - 1\right)\hat{R}_{BC}\right)}{4ж_{BCD} R_{BC}} \tag{S6}$$

$$\vec{\nabla}_D ж_{BCD} = \frac{-\left(\hat{R}_{BC} + \left(2\left(ж_{BCD}\right)^2 - 1\right)\hat{R}_{CD}\right)}{4ж_{BCD} R_{CD}} \tag{S7}$$

$$\vec{\nabla}_C ж_{BCD} = -\left(\vec{\nabla}_B ж_{BCD} + \vec{\nabla}_D ж_{BCD}\right) \tag{S8}$$

S2. Gradients of the directed dihedral

In this article, we use the following convention for the bond vectors:

$$\vec{R}_{AB} = \vec{R}_B - \vec{R}_A = -\vec{R}_{BA} \tag{S9}$$

$$R_{AB} = \left\|\vec{R}_{AB}\right\| = \sqrt{\vec{R}_{AB} \bullet \vec{R}_{AB}} = R_{BA} \tag{S10}$$

$$\hat{R}_{AB} = \vec{R}_{AB} / R_{AB} = -\hat{R}_{BA} \tag{S11}$$

Note that

$$\vec{R}_{AC} = \vec{R}_C - \vec{R}_A = \vec{R}_{AB} + \vec{R}_{BC} \tag{S12}$$

Swope and Ferguson[S1] derived the gradients of the directed dihedral. I converted these into the bond vector convention adopted here (see eqn (S9)–(S11)) and expressed them as vector cross products to get the following formulas. These formulas are mathematically equivalent to—yet expressed much more concisely than—the formulas presented by Swope and Ferguson. First, define

$$\vec{t} = -\left(\vec{R}_{AB} \times \vec{R}_{BC}\right) \quad (S13)$$

$$\vec{u} = -\left(\vec{R}_{BC} \times \vec{R}_{CD}\right) \quad (S14)$$

$$t = \sqrt{\vec{t} \bullet \vec{t}} \quad (S15)$$

$$\hat{t} = \vec{t}/t \quad (S16)$$

$$u = \sqrt{\vec{u} \bullet \vec{u}} \quad (S17)$$

$$\hat{u} = \vec{u}/u \quad (S18)$$

Second, define the following vectors

$$\vec{\Xi} = \frac{\left(\hat{t} \times \hat{R}_{BC}\right)}{t} \quad (S19)$$

$$\vec{\wp} = -\frac{\left(\hat{u} \times \hat{R}_{BC}\right)}{u} \quad (S20)$$

Finally, the gradients of the directed dihedral are given by

$$\vec{\nabla}_A \phi_{ABCD} = \vec{R}_{BC} \times \vec{\Xi} \quad (S21)$$

$$\vec{\nabla}_B \phi_{ABCD} = -\left(\vec{R}_{AC} \times \vec{\Xi}\right) + \left(\vec{R}_{CD} \times \vec{\wp}\right) \quad (S22)$$

$$\vec{\nabla}_C \phi_{ABCD} = \left(\vec{R}_{AB} \times \vec{\Xi}\right) - \left(\vec{R}_{BD} \times \vec{\wp}\right) \quad (S23)$$

$$\vec{\nabla}_D \phi_{ABCD} = \vec{R}_{BC} \times \vec{\wp} \quad (S24)$$

S3. Derivatives of the angle-damping factors

The kangals are related to the bond angles via

$$ж_{ABC} = \cos\left[\theta_{ABC}/2\right] \quad (S25)$$

$$ж_{ABC}^{eq} = \cos\left[\theta_{ABC}^{eq}/2\right] \quad (S26)$$

$$ж_{BCD} = \cos\left[\theta_{BCD}/2\right] \quad (S27)$$

$$ж_{BCD}^{eq} = \cos\left[\theta_{BCD}^{eq}/2\right] \quad (S28)$$

As derived in the main text, the angle-damping factors have the forms

$$f_n^{ABC} = \frac{\tanh\left[KP_n\left[ж_{ABC}\right]\right]}{tanh\_K} \quad (S29)$$

$$f_{n\_eq}^{ABC} = \frac{\tanh\left[KP_n\left[ж_{ABC}^{eq}\right]\right]}{tanh\_K} \quad (S30)$$

with

$$K = \frac{x_{root}}{\sqrt{½}\,(5/8)} = 2.815891616117388\ldots \quad (S31)$$

$$tanh\_K = \tanh\left[K\right] = 0.992861208914406 \quad (S32)$$

The polynomials are:

$$P_1\left[ж\right] = \left(ж + 3(ж)^3\right)/4 \quad (S33)$$

$$P_2\left[ж\right] = \left(3(ж)^2 + (ж)^4\right)/4 \quad (S34)$$

$$P_3\left[ж\right] = \left(6(ж)^3 - 3(ж)^5 + (ж)^7\right)/4 \quad (S35)$$

$$P_4\left[ж\right] = \left(10(ж)^4 - 9(ж)^6 + 3(ж)^8\right)/4 \quad (S36)$$

Explicit formulas are not given here for the higher-order polynomials (i.e., $P_{n\geq 5}[ж]$), because the corresponding modes almost always have negligible contributions to the dihedral's potential energy. These higher-order polynomials would have the form:

$$P_{n\geq 5}[ж] \propto (ж)^{n} + \text{h.o.t.} \tag{S37}$$

where h.o.t. means higher-order terms. Iff n is odd, then all the powers of $ж$ contributing to $P_n[ж]$ must be odd. Iff n is even, then all the powers of $ж$ contributing to $P_n[ж]$ must be even.

For n ≥ 1:

$$\frac{df_n^{ABC}}{dж_{ABC}} = \frac{K}{\tanh\_K}\left(1-\left(\left(f_n^{ABC}\right)\tanh\_K\right)^2\right)P_n'\left[ж_{ABC}\right] \tag{S38}$$

$$P_1'[ж] = \left(1+9(ж)^2\right)/4 \tag{S39}$$

$$P_2'[ж] = \left(3ж+2(ж)^3\right)/2 \tag{S40}$$

$$P_3'[ж] = \left(18(ж)^2-15(ж)^4+7(ж)^6\right)/4 \tag{S41}$$

$$P_4'[ж] = \left(20(ж)^3-27(ж)^5+12(ж)^7\right)/2 \tag{S42}$$

For n = 0:

$$P_0[ж] = 1 \tag{S43}$$

$$f_0^{ABC} = f_0^{BCD} = 1 \tag{S44}$$

$$\frac{df_0^{ABC}}{dж_{ABC}} = \frac{df_0^{BCD}}{dж_{BCD}} = 0 \tag{S45}$$

S4. Analytic formulas for first derivatives and forces of the CADT potential

The first derivative of the CADT potential is

$$\frac{dU_{ABCD}^{CADT}[\phi]}{d\phi} = \sum_{n=1}^{4} nk_\phi^n \sin\left[n\left(\phi-\phi_{eq}\right)\right] + S_{instance}\sum_{n=1}^{4} nb_\phi^n \cos\left[n\left(\phi-\phi_{eq}\right)\right] \tag{S46}$$

The force exerted on atom $G \in \{A,B,C,D\}$ is

$$\vec{F}_G^{dihedral\_ABCD} = -\frac{dU_{ABCD}^{CADT}[\phi_{ABCD}]}{d\phi_{ABCD}}\vec{\nabla}_G\phi_{ABCD} \tag{S47}$$

where $\vec{\nabla}_G\phi_{ABCD}$ is computed as described in Section S2. If $G \notin \{A,B,C,D\}$, then $\vec{F}_G^{dihedral\_ABCD} = 0$.

S5. Analytic formulas for first derivatives and forces of the CACO potential

The first derivative of the CACO potential is

$$\frac{dU_{ABCD}^{CACO}[\phi]}{d\phi} = -k_{CACO}\sum_{n=1}^{4} nc_n^{CO}\sin[n\phi] \tag{S48}$$

The force exerted on atom $G \in \{A,B,C,D\}$ is

$$\vec{F}_G^{dihedral\_ABCD} = -\frac{dU_{ABCD}^{CACO}[\phi_{ABCD}]}{d\phi_{ABCD}}\vec{\nabla}_G\phi_{ABCD} \tag{S49}$$

where $\vec{\nabla}_G\phi_{ABCD}$ is computed as described in Section S2. If $G \notin \{A,B,C,D\}$, then $\vec{F}_G^{dihedral\_ABCD} = 0$.

S6. Analytic formulas for first derivatives and forces of the ADDT potential

After parameterization, the ADDT potential can be equivalently rewritten as

$$U_{ABCD}^{ADDT}\left[ж_{ABC},ж_{BCD},\phi_{ABCD}\right]-U_{ABCD}^{ADDT}\left[ж_{ABC}^{eq},ж_{BCD}^{eq},\phi_{ABCD}^{eq}\right]=$$

$$\sum_{n=1}^{4}\frac{k_\phi^n}{4}\left(\left(\frac{f_n^{ABC}f_{\lfloor n/2\rfloor\_eq}^{ABC}}{f_{n\_eq}^{ABC}f_{\lfloor n/2\rfloor}^{ABC}}\right)^2+\left(\frac{f_{\lfloor n/2\rfloor}^{ABC}}{f_{\lfloor n/2\rfloor\_eq}^{ABC}}\right)^2\right)\left(\left(\frac{f_n^{BCD}f_{\lfloor n/2\rfloor\_eq}^{BCD}}{f_{n\_eq}^{BCD}f_{\lfloor n/2\rfloor}^{BCD}}\right)^2+\left(\frac{f_{\lfloor n/2\rfloor}^{BCD}}{f_{\lfloor n/2\rfloor\_eq}^{BCD}}\right)^2\right) \quad \text{(S50)}$$

$$+\sum_{n=1}^{4}\left(H_n\left[ж_{ABC},ж_{BCD}\right]\left(-k_\phi^n\cos\left[n(\phi-\phi_{eq})\right]+S_{instance}b_\phi^n\sin\left[n(\phi-\phi_{eq})\right]\right)\right)$$

where

$$H_n\left[ж_{ABC},ж_{BCD}\right]=\frac{f_n^{ABC}f_n^{BCD}}{f_{n\_eq}^{ABC}f_{n\_eq}^{BCD}} \quad \text{(S51)}$$

$$b_\phi^1=\frac{3}{\sqrt{10}}k_\phi^5+\frac{1}{\sqrt{15}}k_\phi^7 \quad \text{(S52)}$$

$$b_\phi^2=\frac{2}{\sqrt{5}}k_\phi^6-\frac{1}{\sqrt{15}}k_\phi^7 \quad \text{(S53)}$$

$$b_\phi^3=\frac{-1}{\sqrt{10}}k_\phi^5+\frac{3}{\sqrt{15}}k_\phi^7 \quad \text{(S54)}$$

$$b_\phi^4=\frac{-1}{\sqrt{5}}k_\phi^6-\frac{2}{\sqrt{15}}k_\phi^7 \quad \text{(S55)}$$

The following limits, which retain the leading term in the Taylor series expansion, should be used to avoid division by zero in all equations containing the ratios $f_n^{ABC}/f_{\lfloor n/2\rfloor}^{ABC}$ and/or $f_n^{BCD}/f_{\lfloor n/2\rfloor}^{BCD}$ (e.g., eqn (S50), (S61)–(S62), (S117)–(S118), and others) as one or both bond angles approach linearity:

$$\lim_{ж_{ABC}\to 0}\frac{f_n^{ABC}}{f_{\lfloor n/2\rfloor}^{ABC}}=\lim_{ж_{ABC}\to 0}\frac{P_n\left[ж_{ABC}\right]}{P_{\lfloor n/2\rfloor}\left[ж_{ABC}\right]}=\mu_{\lfloor n/2\rfloor}^{n}\left(ж_{ABC}\right)^{n-\lfloor n/2\rfloor} \quad \text{(S56)}$$

$$\lim_{ж_{BCD}\to 0}\frac{f_n^{BCD}}{f_{\lfloor n/2\rfloor}^{BCD}}=\lim_{ж_{BCD}\to 0}\frac{P_n\left[ж_{BCD}\right]}{P_{\lfloor n/2\rfloor}\left[ж_{BCD}\right]}=\mu_{\lfloor n/2\rfloor}^{n}\left(ж_{BCD}\right)^{n-\lfloor n/2\rfloor} \quad \text{(S57)}$$

$$\mu_1^2=3,\quad \mu_1^3=6,\quad \mu_2^4=10/3 \quad \text{(S58)}$$

I recommend using the leading order expansion shown in eqn (S56) iff $ж_{ABC}<0.001$. I recommend using the leading order expansion shown in eqn (S57) iff $ж_{BCD}<0.001$. For example, if $ж_{ABC}<0.001$ then f_4^{ABC}/f_2^{ABC} to leading order equals $(10/3)(ж_{ABC})^2$.

If the forcefield model includes the Manz angle-bending potential for bond angles ABC and BCD, then neither included bond angle can energetically reach a value of zero.[S3] This follows from the fact that the Manz angle-bending potential has infinite energy as the bond angle's value approaches 0.[S3] Because of this infinite energy, there is a 100% probability of rejecting such a structure during Monte Carlo sampling and also a 0% probably that such a structure can be reached during a constant-energy (e.g., NVE ensemble) classical molecular dynamics simulation. Accordingly, structures having $\theta_{ABC}=0$ and/or $\theta_{BCD}=0$ can simply be assigned infinite total potential energy and safely rejected.

If the forcefield model does not include the Manz angle-bending potential for bond angle ABC or for bond angle BCD, then depending on the potential energy model used it may or may

not be feasible for the structure to energetically reach $\theta_{ABC}=0$ and/or $\theta_{BCD}=0$. *Case A*: If the forcefield model assigns an extremely high (but finite) energy to all structures having $\theta_{ABC}=0$ and/or $\theta_{BCD}=0$, this still means it is extremely unlikely to reach any such structures even a single time during the course of a typical classical molecular dynamics simulation. It also means there is a high probability that none of these structures will be accepted even a single time during the course of a typical Monte Carlo simulation. Accordingly, structures having $\theta_{ABC}=0$ and/or $\theta_{BCD}=0$ can simply be categorized as having 'too high energy to accept' and rejected straight away during a Monte Carlo simulation trial move. Consequently, it is not necessary to explicitly compute a dihedral potential energy for such structures. Case B: If the nonreactive forcefield model assigns a thermally accessible energy to any structures having $\theta_{ABC}=0$ and/or $\theta_{BCD}=0$, this means the nonreactive forcefield model is inaccurate. Recall that $\theta_{ABC}=0$ means atom A is bonded to atom B, and atom B is bonded to atom C. As explained in the companion article, bond angles and associated dihedrals that are part of 3-membered rings are not used to construct angle-bending and dihedral torsion terms in the forcefield and are replaced with Urey-Bradley terms in the forcefield.[S2] Therefore, the inclusion of dihedral ABCD in the forcefield implies that atom C is not bonded to atom A in this forcefield model. If $\theta_{ABC}=0$, this means the atoms are ordered in a line as A-C-B or C-A-B or that atoms A and C have the same nuclear position. Within the non-reactive forcefield approximation, it is not feasible to have a linear A-C-B geometry in which atom A is bonded to B but not to C, or to have a linear C-A-B geometry in which atom C is bonded to B but not to A. Consequently, a nonreactive forcefield model that assign a thermally accessible energy to any structure having $\theta_{ABC}=0$ and/or $\theta_{BCD}=0$ should be flagged as 'unreliable' and modified or replaced to correct this problem.

This means $0<\theta_{ABC}\le\pi$ and $0<\theta_{BCD}\le\pi$ in all thermally accessible structures for a reliable forcefield model. This gives rise to the following three cases.

Case # 1: When neither included bond angle is linear (i.e., $0<\theta_{ABC}<\pi$ and $0<\theta_{BCD}<\pi$)

If $G\notin\{A,B,C,D\}$, then $\vec{F}_G^{\text{dihedral\_ABCD}}=0$. Using the differentiation chain rule, the force exerted on atom $G\in\{A,B,C,D\}$ can be computed as follows:

$$\vec{F}_G^{\text{dihedral\_ABCD}} = -\frac{\partial U_{ABCD}^{ADDT}\left[ж_{ABC},ж_{BCD},\phi_{ABCD}\right]}{\partial ж_{ABC}}\vec{\nabla}_G ж_{ABC} - \frac{\partial U_{ABCD}^{ADDT}\left[ж_{ABC},ж_{BCD},\phi_{ABCD}\right]}{\partial ж_{BCD}}\vec{\nabla}_G ж_{BCD} - \frac{\partial U_{ABCD}^{ADDT}\left[ж_{ABC},ж_{BCD},\phi_{ABCD}\right]}{\partial \phi_{ABCD}}\vec{\nabla}_G \phi_{ABCD}$$

(S59)

If $G\notin\{A,B,C\}$, then $\vec{\nabla}_G ж_{ABC}=0$. If $G\in\{A,B,C\}$, then $\vec{\nabla}_G ж_{ABC}$ is computed as shown in Section S1 above. If $G\notin\{B,C,D\}$, then $\vec{\nabla}_G ж_{BCD}=0$. If $G\in\{B,C,D\}$, then $\vec{\nabla}_G ж_{BCD}$ is computed as shown in Section S1 above. If $G\notin\{A,B,C,D\}$, then $\vec{\nabla}_G \phi_{ABCD}=0$. If $G\in\{A,B,C,D\}$, then $\vec{\nabla}_G \phi_{ABCD}$ is computed as shown in Section S2 above.

The first-order partial derivatives of the ADDT potential are:

$$\frac{\partial U_{ABCD}^{ADDT}\left[ж_{ABC},ж_{BCD},\phi_{ABCD}\right]}{\partial \phi_{ABCD}} = \sum_{n=1}^{4} n\left(H_n\left[ж_{ABC},ж_{BCD}\right]\left(k_\phi^n \sin\left[n(\phi-\phi_{eq})\right] + S_{instance} b_\phi^n \cos\left[n(\phi-\phi_{eq})\right]\right)\right) \quad (S60)$$

$$\frac{\partial U_{ABCD}^{ADDT}\left[ж_{ABC}, ж_{BCD}, \phi_{ABCD}\right]}{\partial ж_{ABC}} =$$

$$\sum_{n=1}^{4}\frac{k_\phi^n}{2}\left(\left(\frac{f_n^{ABC}\left(f_{\lfloor n/2\rfloor\_eq}^{ABC}\right)^2}{\left(f_{n\_eq}^{ABC}\right)^2\left(f_{\lfloor n/2\rfloor}^{ABC}\right)^2}\right)\left(\frac{df_n^{ABC}}{dж_{ABC}}-\frac{f_n^{ABC}}{f_{\lfloor n/2\rfloor}^{ABC}}\frac{df_{\lfloor n/2\rfloor}^{ABC}}{dж_{ABC}}\right)+\left(\frac{f_{\lfloor n/2\rfloor}^{ABC}}{\left(f_{\lfloor n/2\rfloor\_eq}^{ABC}\right)^2}\right)\left(\frac{df_{\lfloor n/2\rfloor}^{ABC}}{dж_{ABC}}\right)\right)\left(\left(\frac{f_n^{BCD}f_{\lfloor n/2\rfloor\_eq}^{BCD}}{f_{n\_eq}^{BCD}f_{\lfloor n/2\rfloor}^{BCD}}\right)^2+\left(\frac{f_{\lfloor n/2\rfloor}^{BCD}}{f_{\lfloor n/2\rfloor\_eq}^{BCD}}\right)^2\right) \quad (S61)$$

$$+\sum_{n=1}^{4}\left(\left(\frac{df_n^{ABC}}{dж_{ABC}}\right)\left(\frac{f_n^{BCD}}{f_{n\_eq}^{ABC}f_{n\_eq}^{BCD}}\right)\left(-k_\phi^n\cos\left[n(\phi-\phi_{eq})\right]+S_{instance}b_\phi^n\sin\left[n(\phi-\phi_{eq})\right]\right)\right)$$

$$\frac{\partial U_{ABCD}^{ADDT}\left[ж_{ABC}, ж_{BCD}, \phi_{ABCD}\right]}{\partial ж_{BCD}} =$$

$$\sum_{n=1}^{4}\frac{k_\phi^n}{2}\left(\left(\frac{f_n^{BCD}\left(f_{\lfloor n/2\rfloor\_eq}^{BCD}\right)^2}{\left(f_{n\_eq}^{BCD}\right)^2\left(f_{\lfloor n/2\rfloor}^{BCD}\right)^2}\right)\left(\frac{df_n^{BCD}}{dж_{BCD}}-\frac{f_n^{BCD}}{f_{\lfloor n/2\rfloor}^{BCD}}\frac{df_{\lfloor n/2\rfloor}^{BCD}}{dж_{BCD}}\right)+\left(\frac{f_{\lfloor n/2\rfloor}^{BCD}}{\left(f_{\lfloor n/2\rfloor\_eq}^{BCD}\right)^2}\right)\left(\frac{df_{\lfloor n/2\rfloor}^{BCD}}{dж_{BCD}}\right)\right)\left(\left(\frac{f_n^{ABC}f_{\lfloor n/2\rfloor\_eq}^{ABC}}{f_{n\_eq}^{ABC}f_{\lfloor n/2\rfloor}^{ABC}}\right)^2+\left(\frac{f_{\lfloor n/2\rfloor}^{ABC}}{f_{\lfloor n/2\rfloor\_eq}^{ABC}}\right)^2\right) \quad (S62)$$

$$+\sum_{n=1}^{4}\left(\left(\frac{df_n^{BCD}}{dж_{BCD}}\right)\left(\frac{f_n^{ABC}}{f_{n\_eq}^{ABC}f_{n\_eq}^{BCD}}\right)\left(-k_\phi^n\cos\left[n(\phi-\phi_{eq})\right]+S_{instance}b_\phi^n\sin\left[n(\phi-\phi_{eq})\right]\right)\right)$$

Case # 2: When both included bond angles are linear (i.e., $\theta_{ABC}=\pi$ and $\theta_{BCD}=\pi$)

In this case, it can readily be shown

$$\vec{F}_G^{dihedral\_ABCD}=0 \quad (S63)$$

for each and every atom G in the material. The proof is as follows. When both $\theta_{ABC}=\pi$ and $\theta_{BCD}=\pi$, it follows from eqn (S29), (S31)–(S36) that $f_n^{ABC}=0$ and $f_n^{BCD}=0$ for n =1 to 4. We can compute the force on each atom using the finite displacement method employing a small (i.e., infinitesimal) displacement. For example, the force on atom G in the x direction can be computed as:

$$F_{G,x}^{dihedral\_ABCD}=\lim_{\Delta X_G\to 0}-\frac{U_{ABCD}^{ADDT}\left[\left\{\vec{R}_{H\neq G},\left(\vec{R}_G^0+\Delta X_G\right)\right\}\right]-U_{ABCD}^{ADDT}\left[\left\{\vec{R}_{H\neq G},\vec{R}_G^0\right\}\right]}{\Delta X_G}=-\frac{\partial U_{ABCD}^{ADDT}}{\partial X_G} \quad (S64)$$

Clearly such a finite displacement will change the value of f_n^{ABC} by either no amount or by some infinitesimal amount proportional to $(\Delta X_G)^{p[n]}$ for p[n] ≥ 1. Also, such a finite displacement will change the value of f_n^{BCD} by either no amount or by some infinitesimal amount proportional to $(\Delta X_G)^{q[n]}$ for q[n] ≥ 1. As shown in eqn (S50), each non-constant term in U_{ABCD}^{ADDT} is proportional to second-order or higher products of angle-damping functions such that in the displaced geometry $\left\{\vec{R}_{H\neq G},\left(\vec{R}_G^0+\Delta X_G\right)\right\}$, these angle-damping function products will either retain a value of zero or acquire a value proportional to $(\Delta X_G)^{t\geq 2}$. This means that $U_{ABCD}^{ADDT}\left[\left\{\vec{R}_{H\neq G},\left(\vec{R}_G^0+\Delta X_G\right)\right\}\right]-U_{ABCD}^{ADDT}\left[\left\{\vec{R}_{H\neq G},\vec{R}_G^0\right\}\right]$ is a polynomial of ΔX_G such that the leading term has a power ≥ 2:

$$U_{ABCD}^{ADDT}\left[\left\{\vec{R}_{H\neq G},\left(\vec{R}_G^0+\Delta X_G\right)\right\}\right]-U_{ABCD}^{ADDT}\left[\left\{\vec{R}_{H\neq G},\vec{R}_G^0\right\}\right]\propto\left(\Delta X_G\right)^{t\geq 2}+\text{h.o.t.} \quad (S65)$$

where the higher-order terms (h.o.t.) have exponents higher than t. Substituting eqn (S65) into (S64) and taking the limit as $\Delta X_G\to 0$ yields $F_{G,x}^{dihedral\_ABCD}=0$. Because exactly the same argument also holds for $\Delta Y_G\to 0$ and $\Delta Z_G\to 0$, this proves eqn (S63).

Case # 3: When only one of the included bond angles is linear (i.e., $\theta_{ABC} = \pi$ xor $\theta_{BCD} = \pi$)

Case # 3a: $\theta_{ABC} = \pi$ **and** $0 < \theta_{BCD} < \pi$

In this case, $ж_{ABC} = 0$, $0 < ж_{BCD} < 1$, $f_n^{ABC} = 0$, and $0 < f_n^{BCD} < 1$ for n = 1 to 4. Since ϕ_{ABCD}^{eq} can exist only if $\theta_{ABC}^{eq} \neq \pi$ and $\theta_{BCD}^{eq} \neq \pi$, it follows that $0 < f_{n\_eq}^{ABC} < 1$ and $0 < f_{n\_eq}^{BCD} < 1$ for n = 1 to 4.

We can compute the force on each atom using the finite displacement method employing a small (i.e., infinitesimal) displacement. Displacing any atom $G \notin \{A, B, C, D\}$ does not change dihedral potential's value; therefore,

$$\vec{F}_{G \notin \{A,B,C,D\}}^{dihedral\_ABCD} = 0 \qquad (S66)$$

Depending on the displacement direction, an extremely small finite displacement of atom A, B, or C could either leave the value of $ж_{ABC}$ unchanged at zero or change it to a value proportional to the displacement length, $\Delta\ell$. In this context, the phrase "value proportional to the displacement length, $\Delta\ell$" specifically means that if the extremely small displacement length is doubled along the same direction for the same atom then the total change in $ж_{ABC}$ also doubles. Examining eqn (S29) and (S33)–(S36), this means the value of f_n^{ABC} either remains unchanged at zero or it is changed to a value proportional to $(\Delta\ell)^n$.

The force of the displaced atom projected onto the displacement direction can be computed as

$$\vec{F}_{displaced\_atom}^{dihedral\_ABCD} \bullet \hat{d} = \lim_{\Delta\ell \to 0} -\frac{U_{ABCD}^{ADDT}[displaced\_geom] - U_{ABCD}^{ADDT}[orig\_geom]}{\Delta\ell} \qquad (S67)$$

where $\hat{d}$ is the direction along which the displaced_atom is displaced and $\Delta\ell$ is the displacement distance. Examining eqn (S67), any terms in the numerator which happen to be proportional to $(\Delta\ell)^{t>1}$ do not contribute to the force. Any terms in the numerator which happen to be proportional to $\Delta\ell$ can contribute to the force, because they are divided by a factor of $\Delta\ell$ in the denominator. As already stated above, upon extremely small finite displacement the value of $f_{n\geq 2}^{ABC}$ either remains unchanged or changes proportional to $(\Delta\ell)^{n\geq 2}$. Therefore, no potential energy terms proportional to $\left(f_{n\geq 2}^{ABC}\right)^{q>(1/n)}$ contribute to the force in Case # 3a. For the same reason, any potential energy terms contributing to U_{ABCD}^{ADDT} that are proportional to $\left(f_1^{ABC}\right)^{q>1}$ do not contribute to the force in Case # 3a. Stripping those negligible terms from the potential energy (eqn (S50)) results in only the following terms surviving for extremely small finite displacements:

$$U_{ABCD}^{ADDT}[displaced\_geom] - U_{ABCD}^{ADDT}[orig\_geom] = \frac{k_\phi^1}{4\left(f_{1\_eq}^{BCD}\right)^2}\left(\left(\underset{\sim}{f}_1^{BCD}\right)^2 - \left(f_1^{BCD}\right)^2\right) + \left(\frac{\underset{\sim}{f}_1^{ABC}\underset{\sim}{f}_1^{BCD}}{f_{1\_eq}^{ABC} f_{1\_eq}^{BCD}}\right)\left(-k_\phi^1 \cos\left[\underset{\sim}{\phi} - \phi_{eq}\right] + S_{instance} b_\phi^1 \sin\left[\underset{\sim}{\phi} - \phi_{eq}\right]\right)$$

(S68)

where orig_geom is the original geometry having $\theta_{ABC} = \pi$. In eqn (S68), $\underset{\sim}{f}_1^{ABC}$, $\underset{\sim}{f}_1^{BCD}$, and $\underset{\sim}{\phi}$ are the values computed for the displaced geometry, while f_1^{BCD} is for the original geometry.

If $k_\phi^1 = S_{instance} b_\phi^1 = 0$, then it directly follows that $\vec{F}_A^{dihedral\_ABCD} = \vec{F}_B^{dihedral\_ABCD} = \vec{F}_C^{dihedral\_ABCD} = \vec{F}_D^{dihedral\_ABCD} = 0$. Otherwise, we proceed as follows. Using the trigonometric identity

$$\cos[\alpha - \beta] = \cos[\alpha]\cos[\beta] + \sin[\alpha]\sin[\beta] \qquad (S69)$$

we can rewrite

$$\left(-k_{\phi}^{1}\cos\left[\underset{\sim}{\phi}-\phi_{eq}\right]+S_{instance}b_{\phi}^{1}\sin\left[\underset{\sim}{\phi}-\phi_{eq}\right]\right)=-\rho\cos\left[\underset{\sim}{\phi}-\Psi\right] \quad (S70)$$

where

$$\rho=+\sqrt{\left(k_{\phi}^{1}\right)^{2}+\left(S_{instance}b_{\phi}^{1}\right)^{2}} \quad (S71)$$

$$\Psi=\phi_{eq}+\arg\left[\frac{k_{\phi}^{1}-S_{instance}b_{\phi}^{1}\sqrt{-1}}{\rho}\right]=\phi_{eq}+\mathrm{atan2}\left[\left(-S_{instance}b_{\phi}^{1}\right),k_{\phi}^{1}\right] \quad (S72)$$

The argument function, $\arg\left[a+b\sqrt{-1}\right]$, is the phase angle of the complex number $a+b\sqrt{-1}$. This gives

$$U_{ABCD}^{ADDT}[\text{displaced\_geom}]-U_{ABCD}^{ADDT}[\text{orig\_geom}]=-\left(\frac{\underset{\sim}{f}_{1}^{ABC}\underset{\sim}{f}_{1}^{BCD}}{f_{1\_eq}^{ABC}f_{1\_eq}^{BCD}}\right)\rho\cos\left[\underset{\sim}{\phi}-\Psi\right]+\frac{k_{\phi}^{1}}{4\left(f_{1\_eq}^{BCD}\right)^{2}}\left(\left(\underset{\sim}{f}_{1}^{BCD}\right)^{2}-\left(f_{1}^{BCD}\right)^{2}\right) \quad (S73)$$

<u>Let's now consider the displacement of atom A.</u> Since $\underset{\sim}{f}_{1}^{ABC}$ depends only on $ж_{ABC}$ for the displaced structure and not on $\underset{\sim}{\phi}$, it follows from eqn (S73) that the largest projected force magnitude is achieved by choosing a displacement direction that maximizes $\left|\cos\left[\underset{\sim}{\phi}-\Phi\right]\right|$. In other words, the projected force is largest in magnitude for $\underset{\sim}{\phi}=\Phi$ and $\underset{\sim}{\phi}=\Phi+\pi$. These correspond to two displacements in opposite directions that give rise to projected forces equal in magnitude with opposite signs. Displacing atom A does not alter f_{n}^{BCD}; thus, $\underset{\sim}{f}_{1}^{BCD}=f_{1}^{BCD}$. Making these substitutions into eqn (S73) gives the following for small finite displacement parallel to $\vec{F}_{A}^{dihedral\_ABCD}$:

$$U_{ABCD}^{ADDT}[\text{displaced\_geom}]-U_{ABCD}^{ADDT}[\text{orig\_geom}]=-\rho\left(\frac{\underset{\sim}{f}_{1}^{ABC}f_{1}^{BCD}}{f_{1\_eq}^{ABC}f_{1\_eq}^{BCD}}\right) \quad (S74)$$

Substituting this into eqn (S67) gives

$$F_{A}^{dihedral\_ABCD}=\left(\frac{\rho f_{1}^{BCD}}{f_{1\_eq}^{ABC}f_{1\_eq}^{BCD}}\right)\left(\lim_{\Delta\ell\to0}\left(\frac{\underset{\sim}{f}_{1}^{ABC}}{\Delta\ell}\right)\right) \quad (S75)$$

Let $\varepsilon_{ABC}=\pi-\underset{\sim}{\theta}_{ABC}$ be the small (infinitesimal) angle produced upon displacing atom A perpendicular to the bond vector $\vec{R}_{BA}$. Then, the displaced distance is given by

$$\Delta\ell\approx\varepsilon_{ABC}R_{BA} \quad (S76)$$

The kangal in the displaced geometry is

$$\underset{\sim}{ж}_{ABC}=\cos\left[\underset{\sim}{\theta}_{ABC}/2\right]=\cos\left[\left(\pi-\varepsilon_{ABC}\right)/2\right]=\sin\left[\varepsilon_{ABC}/2\right]\approx\varepsilon_{ABC}/2 \quad (S77)$$

Examining eqn (S29) and (S33),

$$\lim_{\underset{\sim}{ж}_{ABC}\to\text{infinitesimal}}\underset{\sim}{f}_{1}^{ABC}=\frac{\tanh\left[K\underset{\sim}{ж}_{ABC}/4\right]}{\text{tanh\_K}}=\frac{K\underset{\sim}{ж}_{ABC}}{4\text{tanh\_K}}=\frac{K\varepsilon_{ABC}}{8\text{tanh\_K}} \quad (S78)$$

Substituting eqn (S76) and (S78) into (S75) gives

$$F_{A}^{dihedral\_ABCD}=\left(\frac{\rho f_{1}^{BCD}}{f_{1\_eq}^{ABC}f_{1\_eq}^{BCD}}\right)\left(\frac{K}{8\text{tanh\_K}}\right)\frac{1}{R_{BA}} \quad (S79)$$

The direction of this force is computed as follows. First, we compute a unit vector $\hat{v}$ that is perpendicular to $\vec{R}_{BC}$ that is in the same plane as $\vec{R}_{CD}$ and $\vec{R}_{BC}$:

$$\vec{v}=\vec{R}_{CD}-\hat{R}_{BC}\left(\vec{R}_{CD}\bullet\hat{R}_{BC}\right) \quad (S80)$$

$$\hat{v}=\vec{v}/\left\|\vec{v}\right\| \quad (S81)$$

Using eqn (S14) and (S18), we compute the unit vector $\hat{u}$ that is perpendicular to both $\vec{R}_{BC}$ and $\vec{R}_{CD}$. It follows from basic geometry that the direction of the force is then given by

$$\hat{F}_{A}^{dihedral\_ABCD} = \hat{v}\cos[\Psi] + \hat{u}\sin[\Psi] \qquad (S82)$$

Finally, the magnitude from eqn (S79) and the direction from eqn (S82) are combined to give the force vector:

$$\vec{F}_{A}^{dihedral\_ABCD} = \left(\frac{\rho f_{1}^{BCD}}{f_{1\_eq}^{ABC} f_{1\_eq}^{BCD}}\right)\left(\frac{K}{8tanh\_K}\right)\frac{1}{R_{BA}}\left(\hat{v}\cos[\Psi] + \hat{u}\sin[\Psi]\right) \qquad (S83)$$

<u>Let's now consider the displacement of atom D</u>. Since $f_{n\geq 1}^{ABC}$ depends only on the positions of atoms A, B, and C, its value is not changed by a displacement of atom D. Thus, we have $\underset{\sim}{f}_{n\geq 1}^{ABC} = f_{n\geq 1}^{ABC} = 0$. Substituting this into the ADDT potential (eqn (S50)) shows that the only surviving non-zero term is $\frac{k_{\phi}^{1}}{4}\left(\left(\frac{\underset{\sim}{f}_{1}^{BCD}}{f_{1\_eq}^{BCD}}\right)^{2} + 1\right)$. Therefore, in this case

$$\vec{F}_{D}^{dihedral\_ABCD} = -\frac{\partial U_{ABCD}^{ADDT}[\text{ж}_{ABC}, \text{ж}_{BCD}, \phi_{ABCD}]}{\partial \text{ж}_{BCD}}\vec{\nabla}_{D}\text{ж}_{BCD} = -\frac{k_{\phi}^{1} f_{1}^{BCD}}{2\left(f_{1\_eq}^{BCD}\right)^{2}}\frac{df_{1}^{BCD}}{d\text{ж}_{BCD}}\vec{\nabla}_{D}\text{ж}_{BCD} \qquad (S84)$$

where $df_{1}^{BCD}/d\text{ж}_{BCD}$ is computed from eqn (S38) and $\vec{\nabla}_{D}\text{ж}_{BCD}$ is computed from eqn (S7).

<u>Let's now consider the displacement of atom C</u>. For atom C, we separate changes in the potential energy and the force into two parts. We re-write eqn (S73) as

$$U_{ABCD}^{ADDT}[displaced\_geom] - U_{ABCD}^{ADDT}[orig\_geom] = \Delta U_{part\_1} + \Delta U_{part\_2} \qquad (S85)$$

$$\Delta U_{part\_1} = -\left(\frac{\underset{\sim}{f}_{1}^{ABC}\underset{\sim}{f}_{1}^{BCD}}{f_{1\_eq}^{ABC} f_{1\_eq}^{BCD}}\right)\rho\cos\left[\underset{\sim}{\phi} - \Psi\right] \qquad (S86)$$

$$\Delta U_{part\_2} = \frac{k_{\phi}^{1}}{4\left(f_{1\_eq}^{BCD}\right)^{2}}\left(\left(\underset{\sim}{f}_{1}^{BCD}\right)^{2} - \left(f_{1}^{BCD}\right)^{2}\right) \qquad (S87)$$

$$\vec{F}_{C}^{dihedral\_ABCD} = \vec{F}_{C}^{part\_1} + \vec{F}_{C}^{part\_2} \qquad (S88)$$

$\vec{F}_{C}^{part\_1}$ and $\vec{F}_{C}^{part\_2}$ projected onto the displacement direction can be computed as

$$\vec{F}_{C}^{part\_1}\bullet\hat{d} = \lim_{\Delta\ell\to 0} -\frac{\Delta U_{part\_1}}{\Delta\ell} = \lim_{\Delta\ell\to 0}\left(\frac{\underset{\sim}{f}_{1}^{ABC}\underset{\sim}{f}_{1}^{BCD}}{f_{1\_eq}^{ABC} f_{1\_eq}^{BCD}}\right)\frac{\rho\cos\left[\underset{\sim}{\phi} - \Psi\right]}{\Delta\ell} \qquad (S89)$$

$$\vec{F}_{C}^{part\_2}\bullet\hat{d} = \lim_{\Delta\ell\to 0} -\frac{\Delta U_{part\_2}}{\Delta\ell} = \lim_{\Delta\ell\to 0} -\left(\frac{k_{\phi}^{1}}{4\left(f_{1\_eq}^{BCD}\right)^{2}}\right)\frac{\left(\left(\underset{\sim}{f}_{1}^{BCD}\right)^{2} - \left(f_{1}^{BCD}\right)^{2}\right)}{\Delta\ell} \qquad (S90)$$

where $\hat{d}$ is the direction along which the displaced_atom is displaced and $\Delta\ell$ is the displacement distance. Using the definition of the first derivative, eqn (S90) can be re-written as

$$\vec{F}_{C}^{part\_2} = -\left(\frac{k_{\phi}^{1}}{4\left(f_{1\_eq}^{BCD}\right)^{2}}\right)\frac{\partial\left(\left(f_{1}^{BCD}\right)^{2}\right)}{\partial\text{ж}_{BCD}}\vec{\nabla}_{C}\text{ж}_{BCD} = -\frac{k_{\phi}^{1} f_{1}^{BCD}}{2\left(f_{1\_eq}^{BCD}\right)^{2}}\left(\frac{df_{1}^{BCD}}{d\text{ж}_{BCD}}\right)\vec{\nabla}_{C}\text{ж}_{BCD} \qquad (S91)$$

Since $\underset{\sim}{f}_{1}^{ABC}$ depends only on ж_{ABC} for the displaced structure and not on $\underset{\sim}{\phi}$, it follows from eqn (S89) that the largest projected force magnitude of part_1 is achieved by choosing a displacement direction that maximizes $\left|\cos\left[\underset{\sim}{\phi} - \Phi\right]\right|$. In other words, the projected force in eqn (S89) is largest in magnitude for $\underset{\sim}{\phi} = \Phi$ and $\underset{\sim}{\phi} = \Phi + \pi$. These correspond to two displacements in opposite directions that give rise to projected forces equal in magnitude with opposite signs.

We have the following for infinitesimal displacement away from a linear ABC angle:

$$\underset{\sim}{f}_1^{ABC} = \underset{0}{f_1^{ABC}} + df_1^{ABC} = df_1^{ABC} \tag{S92}$$

$$\underset{\sim}{f}_1^{BCD} = f_1^{BCD} + df_1^{BCD} \tag{S93}$$

$$\underset{\sim}{f}_1^{ABC}\underset{\sim}{f}_1^{BCD} = df_1^{ABC}\left(f_1^{BCD} + df_1^{BCD}\right) = df_1^{ABC}f_1^{BCD} + \underbrace{df_1^{ABC}df_1^{BCD}}_{\text{negligible}} \approx \underset{\sim}{f}_1^{ABC}f_1^{BCD} \tag{S94}$$

The term marked as negligible can be neglected, because it is the product of two infinitesimal changes. Substituting eqn (S94) into (S89) gives

$$F_C^{part\_1} = \left(\frac{\rho f_1^{BCD}}{f_{1\_eq}^{ABC}f_{1\_eq}^{BCD}}\right)\left(\lim_{\Delta\ell\to 0}\left(\frac{\underset{\sim}{f}_1^{ABC}}{\Delta\ell}\right)\right) \tag{S95}$$

Let $\varepsilon_{ABC} = \pi - \underset{\sim}{\theta}_{ABC}$ be the small (infinitesimal) angle produced upon displacing atom C perpendicular to the bond vector $\vec{R}_{BC}$. Then, the displaced distance is given by

$$\Delta\ell \approx \varepsilon_{ABC}R_{BC} \tag{S96}$$

The kangal in the displaced geometry is

$$\underset{\sim}{ж}_{ABC} = \cos\left[\underset{\sim}{\theta}_{ABC}/2\right] = \cos\left[\left(\pi - \varepsilon_{ABC}\right)/2\right] = \sin\left[\varepsilon_{ABC}/2\right] \approx \varepsilon_{ABC}/2 \tag{S97}$$

Examining eqn (S29) and (S33),

$$\lim_{ж_{ABC}\to\text{infinitesimal}} \underset{\sim}{f}_1^{ABC} = \frac{\tanh\left[K\underset{\sim}{ж}_{ABC}/4\right]}{\tanh\_K} = \frac{K\underset{\sim}{ж}_{ABC}}{4\tanh\_K} = \frac{K\varepsilon_{ABC}}{8\tanh\_K} \tag{S98}$$

Substituting eqn (S96) and (S98) into (S95) gives

$$F_C^{part\_1} = \left(\frac{\rho f_1^{BCD}}{f_{1\_eq}^{ABC}f_{1\_eq}^{BCD}}\right)\left(\frac{K}{8\tanh\_K}\right)\frac{1}{R_{BC}} \tag{S99}$$

The direction of $\vec{F}_C^{part\_1}$ is computed as follows. Using eqn (S80) and (S81), we compute the unit vector $\hat{v}$ that is perpendicular to $\vec{R}_{BC}$ that is in the same plane as $\vec{R}_{CD}$ and $\vec{R}_{BC}$. Using eqn (S14) and (S18), we compute the unit vector $\hat{u}$ that is perpendicular to both $\vec{R}_{BC}$ and $\vec{R}_{CD}$. It follows from basic geometry that the direction of the force is then given by

$$\hat{F}_C^{part\_1} = \hat{v}\cos[\Psi] + \hat{u}\sin[\Psi] \tag{S100}$$

The magnitude from eqn (S99) and the direction from eqn (S100) are combined to give:

$$\vec{F}_C^{part\_1} = \left(\frac{\rho f_1^{BCD}}{f_{1\_eq}^{ABC}f_{1\_eq}^{BCD}}\right)\left(\frac{K}{8\tanh\_K}\right)\frac{1}{R_{BC}}\left(\hat{v}\cos[\Psi] + \hat{u}\sin[\Psi]\right) \tag{S101}$$

Finally, eqn (S91) and (S101) are inserted into eqn (S88) to give:

$$\vec{F}_C^{dihedral\_ABCD} = \left(\frac{\rho f_1^{BCD}}{f_{1\_eq}^{ABC}f_{1\_eq}^{BCD}}\right)\left(\frac{K}{8\tanh\_K}\right)\frac{1}{R_{BC}}\left(\hat{v}\cos[\Psi] + \hat{u}\sin[\Psi]\right) - \frac{k_\phi^1 f_1^{BCD}}{2\left(f_{1\_eq}^{BCD}\right)^2}\left(\frac{df_1^{BCD}}{dж_{BCD}}\right)\vec{\nabla}_C ж_{BCD} \tag{S102}$$

<u>Let's now consider the displacement of atom B.</u> Because U_{ABCD}^{ADDT} is invariant to a rigid translation of atoms A, B, C, and D, it follows that the associated forces sum to zero:

$$\vec{F}_A^{dihedral\_ABCD} + \vec{F}_B^{dihedral\_ABCD} + \vec{F}_C^{dihedral\_ABCD} + \vec{F}_D^{dihedral\_ABCD} = 0 \tag{S103}$$

Rearranging gives

$$\vec{F}_B^{dihedral\_ABCD} = -\left(\vec{F}_A^{dihedral\_ABCD} + \vec{F}_C^{dihedral\_ABCD} + \vec{F}_D^{dihedral\_ABCD}\right) \tag{S104}$$

Case # 3b: $0<\theta_{ABC}<\pi$ **and** $\theta_{BCD}=\pi$

If we make the appropriate substitutions of atoms, this case is analogous to Case # 3a and has the following equations:

$$\rho = +\sqrt{\left(k_{\phi}^{1}\right)^{2}+\left(S_{instance}b_{\phi}^{1}\right)^{2}} \tag{S105}$$

$$\Psi = \phi_{eq} + \arg\left[\frac{k_{\phi}^{1}-S_{instance}b_{\phi}^{1}\sqrt{-1}}{\rho}\right] = \phi_{eq} + \text{atan2}\left[\left(-S_{instance}b_{\phi}^{1}\right),k_{\phi}^{1}\right] \tag{S106}$$

Define $\hat{w}$ as a unit vector that is perpendicular to $\vec{R}_{BC}$ that is in the same plane as $\vec{R}_{AB}$ and $\vec{R}_{BC}$:

$$\vec{w} = \vec{R}_{BA} - \left(\vec{R}_{BA}\bullet\hat{R}_{BC}\right)\hat{R}_{BC} \tag{S107}$$

$$\hat{w} = \vec{w}/\|\vec{w}\| \tag{S108}$$

Using eqn (S13) and (S16), define $\hat{t}$ as a unit vector that is perpendicular to both $\vec{R}_{BC}$ and $\vec{R}_{AB}$.

The forces are then given by

$$\vec{F}_{G\notin\{A,B,C,D\}}^{dihedral\_ABCD} = 0 \tag{S109}$$

$$\vec{F}_{A}^{dihedral\_ABCD} = -\frac{\partial U_{ABCD}^{ADDT}\left[ж_{ABC},ж_{BCD},\phi_{ABCD}\right]}{\partial ж_{ABC}}\vec{\nabla}_{A}ж_{ABC} = -\frac{k_{\phi}^{1}f_{1}^{ABC}}{2\left(f_{1\_eq}^{ABC}\right)^{2}}\frac{df_{1}^{ABC}}{dж_{ABC}}\vec{\nabla}_{A}ж_{ABC} \tag{S110}$$

$$\vec{F}_{D}^{dihedral\_ABCD} = \left(\frac{\rho f_{1}^{ABC}}{f_{1\_eq}^{ABC}f_{1\_eq}^{BCD}}\right)\left(\frac{K}{8\tanh\_K}\right)\frac{1}{R_{CD}}\left(\hat{w}\cos[\Psi]-\hat{t}\sin[\Psi]\right) \tag{S111}$$

$$\vec{F}_{B}^{dihedral\_ABCD} = \left(\frac{\rho f_{1}^{ABC}}{f_{1\_eq}^{ABC}f_{1\_eq}^{BCD}}\right)\left(\frac{K}{8\tanh\_K}\right)\frac{1}{R_{BC}}\left(\hat{w}\cos[\Psi]-\hat{t}\sin[\Psi]\right) - \frac{k_{\phi}^{1}f_{1}^{ABC}}{2\left(f_{1\_eq}^{ABC}\right)^{2}}\frac{df_{1}^{ABC}}{dж_{ABC}}\vec{\nabla}_{B}ж_{ABC} \tag{S112}$$

Because U_{ABCD}^{ADDT} is invariant to a rigid translation of atoms A, B, C, and D, it follows that the associated forces sum to zero:

$$\vec{F}_{A}^{dihedral\_ABCD} + \vec{F}_{B}^{dihedral\_ABCD} + \vec{F}_{C}^{dihedral\_ABCD} + \vec{F}_{D}^{dihedral\_ABCD} = 0 \tag{S113}$$

Rearranging this equation gives

$$\vec{F}_{C}^{dihedral\_ABCD} = -\left(\vec{F}_{A}^{dihedral\_ABCD} + \vec{F}_{B}^{dihedral\_ABCD} + \vec{F}_{D}^{dihedral\_ABCD}\right) \tag{S114}$$

S7. Analytic formulas for first derivatives and forces of the ADCO potential

If the forcefield model includes the Manz angle-bending potential for bond angles ABC and BCD, then neither included bond angle can energetically reach a value of zero.[S3] This follows from the fact that the Manz angle-bending potential has infinite energy as the bond angle's value approaches 0.[S3] Because of this infinite energy, there is a 100% probability of rejecting such a structure during Monte Carlo sampling and also a 0% probably that such a structure can be reached during a constant-energy (e.g., NVE ensemble) classical molecular dynamics simulation. Accordingly, structures having $\theta_{ABC}=0$ and/or $\theta_{BCD}=0$ can simply be assigned infinite total potential energy and safely rejected.

If the forcefield model does not include the Manz angle-bending potential for bond angle ABC or for bond angle BCD, then depending on the potential energy model used it may or may not be feasible for the structure to energetically reach $\theta_{ABC}=0$ and/or $\theta_{BCD}=0$. *Case A*: If the forcefield model assigns an extremely high (but finite) energy to all structures having $\theta_{ABC}=0$ and/or $\theta_{BCD}=0$, this still means it is extremely unlikely to reach any such structures even a single

time during the course of a typical classical molecular dynamics simulation. It also means there is a high probability that none of these structures will be accepted even a single time during the course of a typical Monte Carlo simulation. Accordingly, structures having $\theta_{ABC}=0$ and/or $\theta_{BCD}=0$ can simply be categorized as having 'too high energy to accept' and rejected straight away during a Monte Carlo simulation trial move. Consequently, it is not necessary to explicitly compute a dihedral potential energy for such structures. Case B: If the nonreactive forcefield model assigns a thermally accessible energy to any structures having $\theta_{ABC}=0$ and/or $\theta_{BCD}=0$, this means the nonreactive forcefield model is inaccurate. Recall that $\theta_{ABC}=0$ means atom A is bonded to atom B, and atom B is bonded to atom C. As explained in the companion article, bond angles and associated dihedrals that are part of 3-membered rings are not used to construct angle-bending and dihedral torsion terms in the forcefield and are replaced with Urey-Bradley terms in the forcefield.[S2] Therefore, the inclusion of dihedral ABCD in the forcefield implies that atom C is not bonded to atom A in this forcefield model. If $\theta_{ABC}=0$, this means the atoms are ordered in a line as A-C-B or C-A-B or that atoms A and C have the same nuclear position. Within the non-reactive forcefield approximation, it is not feasible to have a linear A-C-B geometry in which atom A is bonded to B but not to C, or to have a linear C-A-B geometry in which atom C is bonded to B but not to A. Consequently, a nonreactive forcefield model that assign a thermally accessible energy to any structure having $\theta_{ABC}=0$ and/or $\theta_{BCD}=0$ should be flagged as 'unreliable' and modified or replaced to correct this problem.

This means $0<\theta_{ABC}\le\pi$ and $0<\theta_{BCD}\le\pi$ in all thermally accessible structures for a reliable forcefield model. This gives rise to the following three cases.

Case # 1: When neither included bond angle is linear (i.e., $0<\theta_{ABC}<\pi$ and $0<\theta_{BCD}<\pi$)

If $G\notin\{A,B,C,D\}$, then $\vec{F}_G^{\text{dihedral\_ABCD}}=0$. Using the differentiation chain rule, the force exerted on atom $G\in\{A,B,C,D\}$ can be computed as follows:

$$\vec{F}_G^{\text{dihedral\_ABCD}}=-\frac{\partial U_{ABCD}^{ADCO}[ж_{ABC},ж_{BCD},\phi_{ABCD}]}{\partial ж_{ABC}}\vec{\nabla}_G ж_{ABC}-\frac{\partial U_{ABCD}^{ADCO}[ж_{ABC},ж_{BCD},\phi_{ABCD}]}{\partial ж_{BCD}}\vec{\nabla}_G ж_{BCD}-\frac{\partial U_{ABCD}^{ADCO}[ж_{ABC},ж_{BCD},\phi_{ABCD}]}{\partial \phi_{ABCD}}\vec{\nabla}_G \phi_{ABCD}$$

(S115)

If $G\notin\{A,B,C\}$, then $\vec{\nabla}_G ж_{ABC}=0$. If $G\in\{A,B,C\}$, then $\vec{\nabla}_G ж_{ABC}$ is computed as shown in Section S1 above. If $G\notin\{B,C,D\}$, then $\vec{\nabla}_G ж_{BCD}=0$. If $G\in\{B,C,D\}$, then $\vec{\nabla}_G ж_{BCD}$ is computed as shown in Section S1 above. If $G\notin\{A,B,C,D\}$, then $\vec{\nabla}_G \phi_{ABCD}=0$. If $G\in\{A,B,C,D\}$, then $\vec{\nabla}_G \phi_{ABCD}$ is computed as shown in Section S2 above.

The first-order partial derivatives of the ADCO potential are:

$$\frac{\partial U_{ABCD}^{ADCO}[ж_{ABC},ж_{BCD},\phi_{ABCD}]}{\partial \phi_{ABCD}}= -k_{ADCO}\sum_{n=1}^{4} n c_n^{CO} H_n[ж_{ABC},ж_{BCD}]\sin[n\phi] \quad \text{(S116)}$$

$$\frac{\partial U_{ABCD}^{ADCO}[ж_{ABC}, ж_{BCD}, \phi_{ABCD}]}{\partial ж_{ABC}} =$$

$$-\frac{k_{ADCO}}{2}\sum_{n=1}^{4} c_n^{CO}\cos\left[n\phi_{eq}^{training}\right]\left(\left(\frac{f_n^{ABC}\left(f_{\lfloor n/2\rfloor\_eq}^{ABC}\right)^2}{\left(f_{n\_eq}^{ABC}\right)^2\left(f_{\lfloor n/2\rfloor}^{ABC}\right)^2}\right)\left(\frac{df_n^{ABC}}{dж_{ABC}} - \frac{f_n^{ABC}}{f_{\lfloor n/2\rfloor}^{ABC}}\frac{df_{\lfloor n/2\rfloor}^{ABC}}{dж_{ABC}}\right) + \left(\frac{f_{\lfloor n/2\rfloor}^{ABC}}{\left(f_{\lfloor n/2\rfloor\_eq}^{ABC}\right)^2}\right)\left(\frac{df_{\lfloor n/2\rfloor}^{ABC}}{dж_{ABC}}\right)\right)\left(\left(\frac{f_n^{BCD} f_{\lfloor n/2\rfloor\_eq}^{BCD}}{f_{n\_eq}^{BCD} f_{\lfloor n/2\rfloor}^{BCD}}\right)^2 + \left(\frac{f_{\lfloor n/2\rfloor}^{BCD}}{f_{\lfloor n/2\rfloor\_eq}^{BCD}}\right)^2\right)$$

$$+ \quad k_{ADCO}\sum_{n=1}^{4} c_n^{CO}\left(\frac{df_n^{ABC}}{dж_{ABC}}\right)\left(\frac{f_n^{BCD}}{f_{n\_eq}^{ABC} f_{n\_eq}^{BCD}}\right)\cos[n\phi]$$

(S117)

$$\frac{\partial U_{ABCD}^{ADCO}[ж_{ABC}, ж_{BCD}, \phi_{ABCD}]}{\partial ж_{BCD}} =$$

$$-\frac{k_{ADCO}}{2}\sum_{n=1}^{4} c_n^{CO}\cos\left[n\phi_{eq}^{training}\right]\left(\left(\frac{f_n^{BCD}\left(f_{\lfloor n/2\rfloor\_eq}^{BCD}\right)^2}{\left(f_{n\_eq}^{BCD}\right)^2\left(f_{\lfloor n/2\rfloor}^{BCD}\right)^2}\right)\left(\frac{df_n^{BCD}}{dж_{BCD}} - \frac{f_n^{BCD}}{f_{\lfloor n/2\rfloor}^{BCD}}\frac{df_{\lfloor n/2\rfloor}^{BCD}}{dж_{BCD}}\right) + \left(\frac{f_{\lfloor n/2\rfloor}^{BCD}}{\left(f_{\lfloor n/2\rfloor\_eq}^{BCD}\right)^2}\right)\left(\frac{df_{\lfloor n/2\rfloor}^{BCD}}{dж_{BCD}}\right)\right)\left(\left(\frac{f_n^{ABC} f_{\lfloor n/2\rfloor\_eq}^{ABC}}{f_{n\_eq}^{ABC} f_{\lfloor n/2\rfloor}^{ABC}}\right)^2 + \left(\frac{f_{\lfloor n/2\rfloor}^{ABC}}{f_{\lfloor n/2\rfloor\_eq}^{ABC}}\right)^2\right)$$

$$+ \quad k_{ADCO}\sum_{n=1}^{4} c_n^{CO}\left(\frac{df_n^{BCD}}{dж_{BCD}}\right)\left(\frac{f_n^{ABC}}{f_{n\_eq}^{ABC} f_{n\_eq}^{BCD}}\right)\cos[n\phi]$$

(S118)

Case # 2: When both included bond angles are linear (i.e., $\theta_{ABC} = \pi$ and $\theta_{BCD} = \pi$)

In this case, it can readily be shown

$$\vec{F}_G^{dihedral\_ABCD} = 0 \qquad \text{(S119)}$$

for each and every atom G in the material. The proof is as follows. When both $\theta_{ABC} = \pi$ and $\theta_{BCD} = \pi$, it follows from eqn (S29), (S31)–(S36) that $f_n^{ABC} = 0$ and $f_n^{BCD} = 0$ for n =1 to 4. We can compute the force on each atom using the finite displacement method employing a small (i.e., infinitesimal) displacement. For example, the force on atom G in the x direction can be computed as:

$$F_{G,x}^{dihedral\_ABCD} = \lim_{\Delta X_G \to 0} -\frac{U_{ABCD}^{ADCO}\left[\left\{\vec{R}_{H\neq G}, \left(\vec{R}_G^0 + \Delta X_G\right)\right\}\right] - U_{ABCD}^{ADCO}\left[\left\{\vec{R}_{H\neq G}, \vec{R}_G^0\right\}\right]}{\Delta X_G} = -\frac{\partial U_{ABCD}^{ADCO}}{\partial X_G} \qquad \text{(S120)}$$

Clearly such a finite displacement will change the value of f_n^{ABC} by either no amount or by some infinitesimal amount proportional to $(\Delta X_G)^{p[n]}$ for p[n] ≥ 1. Also, such a finite displacement will change the value of f_n^{BCD} by either no amount or by some infinitesimal amount proportional to $(\Delta X_G)^{q[n]}$ for q[n] ≥ 1. Each non-constant term in U_{ABCD}^{ADCO} is proportional to second-order or higher products of angle-damping functions such that in the displaced geometry $\left\{\vec{R}_{H\neq G}, \left(\vec{R}_G^0 + \Delta X_G\right)\right\}$, these angle-damping function products will either retain a value of zero or acquire a value proportional to $(\Delta X_G)^{t\geq 2}$. This means that $U_{ABCD}^{ADCO}\left[\left\{\vec{R}_{H\neq G}, \left(\vec{R}_G^0 + \Delta X_G\right)\right\}\right] - U_{ABCD}^{ADCO}\left[\left\{\vec{R}_{H\neq G}, \vec{R}_G^0\right\}\right]$ is a polynomial of ΔX_G such that the leading term has a power ≥ 2:

$$U_{ABCD}^{ADCO}\left[\left\{\vec{R}_{H\neq G}, \left(\vec{R}_G^0 + \Delta X_G\right)\right\}\right] - U_{ABCD}^{ADCO}\left[\left\{\vec{R}_{H\neq G}, \vec{R}_G^0\right\}\right] \propto \left(\Delta X_G\right)^{t\geq 2} + \text{h.o.t.} \qquad \text{(S121)}$$

where the higher-order terms (h.o.t.) have exponents higher than t. Substituting eqn (S121) into (S120) and taking the limit as $\Delta X_G \to 0$ yields $F_{G,x}^{dihedral\_ABCD} = 0$. Because exactly the same argument also holds for $\Delta Y_G \to 0$ and $\Delta Z_G \to 0$, this proves eqn (S119).

Case # 3: When only one of the included bond angles is linear (i.e., $\theta_{ABC} = \pi$ xor $\theta_{BCD} = \pi$)

Case # 3a: $\theta_{ABC} = \pi$ **and** $0 < \theta_{BCD} < \pi$

In this case, $ж_{ABC} = 0$, $0 < ж_{BCD} < 1$, $f_n^{ABC} = 0$, and $0 < f_n^{BCD} < 1$ for n = 1 to 4. Since ϕ_{ABCD}^{eq} can exist only if $\theta_{ABC}^{eq} \neq \pi$ and $\theta_{BCD}^{eq} \neq \pi$, it follows that $0 < f_{n\_eq}^{ABC} < 1$ and $0 < f_{n\_eq}^{BCD} < 1$ for n = 1 to 4.

We can compute the force on each atom using the finite displacement method employing a small (i.e., infinitesimal) displacement. Displacing any atom $G \notin \{A, B, C, D\}$ does not change dihedral potential's value; therefore,

$$\vec{F}_{G \notin \{A,B,C,D\}}^{dihedral\_ABCD} = 0 \tag{S122}$$

Depending on the displacement direction, an extremely small finite displacement of atom A, B, or C could either leave the value of $ж_{ABC}$ unchanged at zero or change it to a value proportional to the displacement length, $\Delta\ell$. In this context, the phrase "value proportional to the displacement length, $\Delta\ell$" specifically means that if the extremely small displacement length is doubled along the same direction for the same atom then the total change in $ж_{ABC}$ also doubles. Examining eqn (S29) and (S33)–(S36), this means the value of f_n^{ABC} either remains unchanged at zero or it is changed to a value proportional to $(\Delta\ell)^n$.

The force of the displaced atom projected onto the displacement direction can be computed as

$$\vec{F}_{displaced\_atom}^{dihedral\_ABCD} \bullet \hat{d} = \lim_{\Delta\ell \to 0} -\frac{U_{ABCD}^{ADCO}[displaced\_geom] - U_{ABCD}^{ADCO}[orig\_geom]}{\Delta\ell} \tag{S123}$$

where $\hat{d}$ is the direction along which the displaced_atom is displaced and $\Delta\ell$ is the displacement distance. Examining eqn (S123), any terms in the numerator which happen to be proportional to $(\Delta\ell)^{t>1}$ do not contribute to the force. Any terms in the numerator which happen to be proportional to $\Delta\ell$ can contribute to the force, because they are divided by a factor of $\Delta\ell$ in the denominator. As already stated above, upon extremely small finite displacement the value of $f_{n\geq 2}^{ABC}$ either remains unchanged or changes proportional to $(\Delta\ell)^{n\geq 2}$. Therefore, no potential energy terms proportional to $\left(f_{n\geq 2}^{ABC}\right)^{q>(1/n)}$ contribute to the force in Case # 3a. For the same reason, any potential energy terms contributing to U_{ABCD}^{ADCO} that are proportional to $\left(f_1^{ABC}\right)^{q>1}$ do not contribute to the force in Case # 3a. Stripping those negligible terms from the ADCO potential energy results in only the following term surviving for extremely small finite displacements:

$$U_{ABCD}^{ADCO}[displaced\_geom] - U_{ABCD}^{ADCO}[orig\_geom] = k_{ADCO} c_1^{CO} \left(\left(\frac{\underset{\sim}{f}_1^{ABC} \underset{\sim}{f}_1^{BCD}}{f_{1\_eq}^{ABC} f_{1\_eq}^{BCD}} \right) \cos[\underset{\sim}{\phi}] - \frac{1}{4\left(f_{1\_eq}^{BCD}\right)^2} \left(\left(\underset{\sim}{f}_1^{BCD}\right)^2 - \left(f_1^{BCD}\right)^2 \right) \cos\left[\phi_{eq}^{training}\right] \right)$$

(S124)

where orig_geom is the original geometry having $\theta_{ABC} = \pi$. In eqn (S124), $\underset{\sim}{f}_1^{ABC}$, $\underset{\sim}{f}_1^{BCD}$, and $\underset{\sim}{\phi}$ are the values computed for the displaced geometry.

If $k_{ADCO} c_1^{CO} = 0$, then it directly follows that $\vec{F}_A^{dihedral\_ABCD} = \vec{F}_B^{dihedral\_ABCD} = \vec{F}_C^{dihedral\_ABCD} = \vec{F}_D^{dihedral\_ABCD} = 0$. Otherwise, we proceed as follows. Let

$$\rho = \left| k_{ADCO} c_1^{CO} \right| \tag{S125}$$

$$\Psi = \begin{cases} \pi & \text{if } \left(k_{ADCO} c_1^{CO}\right) \geq 0 \\ 0 & \text{if } \left(k_{ADCO} c_1^{CO}\right) < 0 \end{cases} \tag{S126}$$

Using these variable substitutions, eqn (S124) is rewritten as

$$U_{ABCD}^{ADCO}[\text{displaced\_geom}] - U_{ABCD}^{ADCO}[\text{orig\_geom}] = -\left(\frac{\underset{\sim}{f}_1^{ABC}\underset{\sim}{f}_1^{BCD}}{f_{1\_eq}^{ABC} f_{1\_eq}^{BCD}}\right)\left(\rho\cos\left[\underset{\sim}{\phi} - \Psi\right]\right) - \frac{k_{ADCO} c_1^{CO} \cos\left[\phi_{eq}^{training}\right]}{4\left(f_{1\_eq}^{BCD}\right)^2}\left(\left(\underset{\sim}{f}_1^{BCD}\right)^2 - \left(f_1^{BCD}\right)^2\right) \tag{S127}$$

Since the right-hand sides of eqn (S73) and (S127) have analogous forms, we can reuse the force expressions derived in Section S6 for the ADDT potential and adopt them to the ADCO potential by making the appropriate variable substitutions. Additionally, we note that eqn (S125) and (S126) combine to give

$$\rho\cos[\Psi] = -k_{ADCO} c_1^{CO} \tag{S128}$$

From eqn (S83), (S84), (S102), and (S104) this gives

$$\vec{F}_A^{dihedral\_ABCD} = -k_{ADCO} c_1^{CO}\left(\frac{f_1^{BCD}}{f_{1\_eq}^{ABC} f_{1\_eq}^{BCD}}\right)\left(\frac{K}{8\tanh\_K}\right)\frac{1}{R_{BA}}\hat{v} \tag{S129}$$

$$\vec{F}_D^{dihedral\_ABCD} = -\frac{\partial U_{ABCD}^{ADCO}[ж_{ABC}, ж_{BCD}, \phi_{ABCD}]}{\partial ж_{BCD}}\vec{\nabla}_D ж_{BCD} = k_{ADCO} c_1^{CO} \cos\left[\phi_{eq}^{training}\right]\left(\frac{f_1^{BCD}}{2\left(f_{1\_eq}^{BCD}\right)^2}\right)\frac{df_1^{BCD}}{dж_{BCD}}\vec{\nabla}_D ж_{BCD} \tag{S130}$$

$$\vec{F}_C^{dihedral\_ABCD} = -k_{ADCO} c_1^{CO}\left(\frac{f_1^{BCD}}{f_{1\_eq}^{ABC} f_{1\_eq}^{BCD}}\right)\left(\frac{K}{8\tanh\_K}\right)\frac{1}{R_{BC}}\hat{v} + k_{ADCO} c_1^{CO} \cos\left[\phi_{eq}^{training}\right]\left(\frac{f_1^{BCD}}{2\left(f_{1\_eq}^{BCD}\right)^2}\right)\frac{df_1^{BCD}}{dж_{BCD}}\vec{\nabla}_C ж_{BCD} \tag{S131}$$

$$\vec{F}_B^{dihedral\_ABCD} = -\left(\vec{F}_A^{dihedral\_ABCD} + \vec{F}_C^{dihedral\_ABCD} + \vec{F}_D^{dihedral\_ABCD}\right) \tag{S132}$$

where $\hat{v}$ is computed from eqn (S81).

Case # 3b: $0 < \theta_{ABC} < \pi$ **and** $\theta_{BCD} = \pi$

If we make the appropriate substitutions of atoms, this case is analogous to Case # 3a and has the following equations for the atom-in-material forces:

$$\vec{F}_{G \notin \{A,B,C,D\}}^{dihedral\_ABCD} = 0 \tag{S133}$$

$$\vec{F}_A^{dihedral\_ABCD} = k_{ADCO} c_1^{CO} \cos\left[\phi_{eq}^{training}\right]\left(\frac{f_1^{ABC}}{2\left(f_{1\_eq}^{ABC}\right)^2}\right)\frac{df_1^{ABC}}{dж_{ABC}}\vec{\nabla}_A ж_{ABC} \tag{S134}$$

$$\vec{F}_D^{dihedral\_ABCD} = -k_{ADCO} c_1^{CO}\left(\frac{f_1^{ABC}}{f_{1\_eq}^{ABC} f_{1\_eq}^{BCD}}\right)\left(\frac{K}{8\tanh\_K}\right)\frac{1}{R_{CD}}\hat{w} \tag{S135}$$

$$\vec{F}_B^{dihedral\_ABCD} = -k_{ADCO} c_1^{CO}\left(\frac{f_1^{ABC}}{f_{1\_eq}^{ABC} f_{1\_eq}^{BCD}}\right)\left(\frac{K}{8\tanh\_K}\right)\frac{1}{R_{BC}}\hat{w} + k_{ADCO} c_1^{CO} \cos\left[\phi_{eq}^{training}\right]\left(\frac{f_1^{ABC}}{2\left(f_{1\_eq}^{ABC}\right)^2}\right)\frac{df_1^{ABC}}{dж_{ABC}}\vec{\nabla}_B ж_{ABC} \tag{S136}$$

$$\vec{F}_C^{dihedral\_ABCD} = -\left(\vec{F}_A^{dihedral\_ABCD} + \vec{F}_B^{dihedral\_ABCD} + \vec{F}_D^{dihedral\_ABCD}\right) \tag{S137}$$

where $\hat{w}$ is computed from eqn (S108).

S8. Selectivity rule for single-linear dihedrals

For single-linear dihedrals, which have exactly one included linear equilibrium bond angle (i.e., $\pi - \theta_{ABC}^{eq} < \varepsilon$ xor $\pi - \theta_{BCD}^{eq} < \varepsilon$), the n = (2(j=1) – 1) = 1 terms are always eliminated (i.e., their force constants are zero). This selectivity rule is proved as follows. First, we note the following requirement on the potential energy:

$$U_{ABCD}^{ADLD}\left[Ж_{ABC}^{eq}, Ж_{BCD}^{eq}, \phi_{ABCD}\right] = 0 \text{ if either } Ж_{ABC}^{eq} = 0 \text{ or } Ж_{BCD}^{eq} = 0 \tag{S138}$$

Eqn (S138) arises because the dihedral scan energy curve is flat if one of the bond angles is linear and the relative potential energy is zero at the equilibrium geometry. For a single-linear dihedral, evaluating eqn (S141) at the equilibrium bond angles gives:

$$U_{ABCD}^{ADLD}\left[Ж_{ABC}^{eq},Ж_{BCD}^{eq},\phi_{ABCD}\right]=\begin{cases}\frac{1}{2}\left(f_1^{ABC}\right)^2\left(k_{LD4}^1+k_{LD5}^1\right) & \text{if } \left(f_{1\_eq}^{ABC}\neq 0 \text{ and } f_{1\_eq}^{BCD}=0\right)\\ \frac{1}{2}\left(f_1^{BCD}\right)^2\left(k_{LD4}^1+k_{LD5}^1\right) & \text{if } \left(f_{1\_eq}^{ABC}=0 \text{ and } f_{1\_eq}^{BCD}\neq 0\right)\end{cases} \quad \text{(S139)}$$

Combining eqn (S138) and (S139) shows that

$$\left(k_{LD4}^1+k_{LD5}^1\right)=0 \quad \text{(S140)}$$

As explained in Section 9.1 of the main text, $k_{LD4}^j\geq 0$ and $k_{LD5}^j\geq 0$. Inserting these constraints into eqn (S140) gives $k_{LD4}^1=0$ and $k_{LD5}^1=0$ for single-linear dihedrals. Second, we note the following requirement on the potential energy's first derivatives. For a single-linear dihedral, the potential energy reaches a minimum and hence its first derivatives (specifically, $\partial U_{ABCD}^{ADLD}/\partial ж_{ABC}$, $\partial U_{ABCD}^{ADLD}/\partial ж_{BCD}$, and $\partial U_{ABCD}^{ADLD}/\partial\phi_{ABCD}$) must be zero when $(Ж_{ABC},Ж_{BCD})=\left(Ж_{ABC}^{eq},Ж_{BCD}^{eq}\right)$. Examining eqn (S143)–(S145), this requires that $k_{LD4}^1=k_{LD5}^1=k_{LD6}^1=0$ for all single-linear dihedrals.

S9. Analytic formulas for first derivatives and forces of the ADLD potential

As explained in this article's main text, my angle-damped-linear-dihedral (ADLD) potential

$$\begin{aligned}U_{ABCD}^{ADLD}\left[Ж_{ABC},Ж_{BCD},\phi_{ABCD}\right]&=\sum_{j=1}^{\infty}\left(U_{2j}^{ADLD}+U_{2j-1}^{ADLD}\right)=\\ &\sum_{j=1}^{\infty}\left(f_j^{ABC}\right)^2\left(f_j^{BCD}\right)^2\left(k_{LD1}^j\left(1-\cos\left[2j\phi_{ABCD}\right]\right)+k_{LD2}^j\left(1+\cos\left[2j\phi_{ABCD}\right]\right)+k_{LD3}^jS_{instance}\sin\left[2j\phi_{ABCD}\right]\right)\\ &+\sum_{j=1}^{\infty}f_j^{ABC}f_{j-1}^{ABC}f_j^{BCD}f_{j-1}^{BCD}\left(\left(-k_{LD4}^j+k_{LD5}^j\right)\cos\left[(2j-1)\phi_{ABCD}\right]+k_{LD6}^jS_{instance}\sin\left[(2j-1)\phi_{ABCD}\right]\right)\\ &+\frac{1}{2}\sum_{j=1}^{\infty}\left(\left(f_j^{ABC}\right)^2\left(f_{j-1}^{BCD}\right)^2+\left(f_{j-1}^{ABC}\right)^2\left(f_j^{BCD}\right)^2\right)\left(k_{LD4}^j+k_{LD5}^j\right)\end{aligned} \quad \text{(S141)}$$

applies to dihedrals in which one or both of the contained equilibrium bond angles is linear (i.e., equal to π within a tolerance). I now derive the forces for this ADLD potential.

Case # A: When neither included bond angle is linear (i.e., $0<\theta_{ABC}<\pi$ and $0<\theta_{BCD}<\pi$)

If $G\notin\{A,B,C,D\}$, then $\vec{F}_G^{dihedral\_ABCD}=0$. Using the differentiation chain rule, the force exerted on atom $G\in\{A,B,C,D\}$ can be computed as follows:

$$\vec{F}_G^{dihedral\_ABCD}=-\frac{\partial U_{ABCD}^{ADLD}\left[ж_{ABC},ж_{BCD},\phi_{ABCD}\right]}{\partial ж_{ABC}}\vec{\nabla}_G ж_{ABC}-\frac{\partial U_{ABCD}^{ADLD}\left[ж_{ABC},ж_{BCD},\phi_{ABCD}\right]}{\partial ж_{BCD}}\vec{\nabla}_G ж_{BCD}-\frac{\partial U_{ABCD}^{ADLD}\left[ж_{ABC},ж_{BCD},\phi_{ABCD}\right]}{\partial\phi_{ABCD}}\vec{\nabla}_G\phi_{ABCD}$$

(S142)

If $G\notin\{A,B,C\}$, then $\vec{\nabla}_G ж_{ABC}=0$. If $G\in\{A,B,C\}$, then $\vec{\nabla}_G ж_{ABC}$ is computed as shown in Section S1 above. If $G\notin\{B,C,D\}$, then $\vec{\nabla}_G ж_{BCD}=0$. If $G\in\{B,C,D\}$, then $\vec{\nabla}_G ж_{BCD}$ is computed as shown in Section S1 above. If $G\notin\{A,B,C,D\}$, then $\vec{\nabla}_G\phi_{ABCD}=0$. If $G\in\{A,B,C,D\}$, then $\vec{\nabla}_G\phi_{ABCD}$ is computed as shown in Section S2 above.

The required partial derivatives of the ADLD potential are as follows:

$$\begin{aligned}&\frac{\partial U_{ABCD}^{ADLD}[ж_{ABC},ж_{BCD},\phi_{ABCD}]}{\partial ж_{ABC}}=\\&2\sum_{j=1}^{\infty}f_j^{ABC}\left(\frac{df_j^{ABC}}{dж_{ABC}}\right)\left(f_j^{BCD}\right)^2\left(k_{LD1}^j\left(1-\cos[2j\phi_{ABCD}]\right)+k_{LD2.}^j\left(1+\cos[2j\phi_{ABCD}]\right)+k_{LD3}^jS_{instance}\sin[2j\phi_{ABCD}]\right)\\&+\sum_{j=1}^{\infty}\left(f_j^{ABC}\left(\frac{df_{j-1}^{ABC}}{dж_{ABC}}\right)+f_{j-1}^{ABC}\left(\frac{df_j^{ABC}}{dж_{ABC}}\right)\right)f_j^{BCD}f_{j-1}^{BCD}\left(\left(-k_{LD4}^j+k_{LD5}^j\right)\cos\left[(2j-1)\phi_{ABCD}\right]+k_{LD6}^jS_{instance}\sin\left[(2j-1)\phi_{ABCD}\right]\right)\\&+\sum_{j=1}^{\infty}\left(f_j^{ABC}\left(\frac{df_j^{ABC}}{dж_{ABC}}\right)\left(f_{j-1}^{BCD}\right)^2+f_{j-1}^{ABC}\left(\frac{df_{j-1}^{ABC}}{dж_{ABC}}\right)\left(f_j^{BCD}\right)^2\right)\left(k_{LD4}^j+k_{LD5}^j\right)\end{aligned}\qquad (S143)$$

$$\begin{aligned}&\frac{\partial U_{ABCD}^{ADLD}[ж_{ABC},ж_{BCD},\phi_{ABCD}]}{\partial ж_{BCD}}=\\&2\sum_{j=1}^{\infty}\left(f_j^{ABC}\right)^2f_j^{BCD}\left(\frac{df_j^{BCD}}{dж_{BCD}}\right)\left(k_{LD1}^j\left(1-\cos[2j\phi_{ABCD}]\right)+k_{LD2.}^j\left(1+\cos[2j\phi_{ABCD}]\right)+k_{LD3}^jS_{instance}\sin[2j\phi_{ABCD}]\right)\\&+\sum_{j=1}^{\infty}f_j^{ABC}f_{j-1}^{ABC}\left(f_j^{BCD}\left(\frac{df_{j-1}^{BCD}}{dж_{BCD}}\right)+f_{j-1}^{BCD}\left(\frac{df_j^{BCD}}{dж_{BCD}}\right)\right)\left(\left(-k_{LD4}^j+k_{LD5}^j\right)\cos\left[(2j-1)\phi_{ABCD}\right]+k_{LD6}^jS_{instance}\sin\left[(2j-1)\phi_{ABCD}\right]\right)\\&+\sum_{j=1}^{\infty}\left(\left(f_j^{ABC}\right)^2f_{j-1}^{BCD}\left(\frac{df_{j-1}^{BCD}}{dж_{BCD}}\right)+\left(f_{j-1}^{ABC}\right)^2f_j^{BCD}\left(\frac{df_j^{BCD}}{dж_{BCD}}\right)\right)\left(k_{LD4}^j+k_{LD5}^j\right)\end{aligned}\qquad (S144)$$

$$\begin{aligned}&\frac{\partial U_{ABCD}^{ADLD}[ж_{ABC},ж_{BCD},\phi_{ABCD}]}{\partial \phi_{ABCD}}=\\&\sum_{j=1}^{\infty}2j\left(f_j^{ABC}\right)^2\left(f_j^{BCD}\right)^2\left(\left(k_{LD1}^j-k_{LD2.}^j\right)\sin[2j\phi_{ABCD}]+k_{LD3}^jS_{instance}\cos[2j\phi_{ABCD}]\right)\\&+\sum_{j=1}^{\infty}(2j-1)f_j^{ABC}f_{j-1}^{ABC}f_j^{BCD}f_{j-1}^{BCD}\left(\left(k_{LD4}^j-k_{LD5}^j\right)\sin\left[(2j-1)\phi_{ABCD}\right]+k_{LD6}^jS_{instance}\cos\left[(2j-1)\phi_{ABCD}\right]\right)\end{aligned}\qquad (S145)$$

Derivatives of the angle-damping factors are presented above in Section S3.

Case # B: When both included bond angles are linear (i.e., $\theta_{ABC}=\pi$ and $\theta_{BCD}=\pi$)

In this case, it can readily be shown

$$\vec{F}_G^{dihedral\_ABCD}=0 \qquad (S146)$$

for each and every atom G in the material. The proof is as follows. When both $\theta_{ABC}=\pi$ and $\theta_{BCD}=\pi$, it follows from eqn (S29), (S31)–(S36) that $f_n^{ABC}=0$ and $f_n^{BCD}=0$ for n ≥ 1. We can compute the force on each atom using the finite displacement method employing a small (i.e., infinitesimal) displacement. For example, the force on atom G in the x direction can be computed as:

$$F_{G,x}^{dihedral\_ABCD}=\lim_{\Delta X_G\to 0}-\frac{U_{ABCD}^{ADLD}\left[\left\{\vec{R}_{H\neq G},\left(\vec{R}_G^0+\Delta X_G\right)\right\}\right]-U_{ABCD}^{ADLD}\left[\left\{\vec{R}_{H\neq G},\vec{R}_G^0\right\}\right]}{\Delta X_G}=-\frac{\partial U_{ABCD}^{ADLD}}{\partial X_G} \qquad (S147)$$

Clearly such a finite displacement will change the value of f_n^{ABC} by either no amount or by some infinitesimal amount proportional to $(\Delta X_G)^{p[n]}$ for p[n] ≥ 1. Also, such a finite displacement will change the value of f_n^{BCD} by either no amount or by some infinitesimal amount proportional to $(\Delta X_G)^{q[n]}$ for q[n] ≥ 1. As shown in eqn (S141), each non-constant term in U_{ABCD}^{ADLD} is proportional to (a sum of) second-order or higher-order product(s) of some angle-damping factors having n ≥ 1. (For j = 1 in eqn (S141), the angle-damping terms $\left(f_j^{ABC}\right)^2\left(f_j^{BCD}\right)^2$, $f_j^{ABC}f_{j-1}^{ABC}f_j^{BCD}f_{j-1}^{BCD}$, and

$\left(\left(f_j^{ABC}\right)^2\left(f_{j-1}^{BCD}\right)^2+\left(f_{j-1}^{ABC}\right)^2\left(f_j^{BCD}\right)^2\right)$ become $\left(f_1^{ABC}\right)^2\left(f_1^{BCD}\right)^2$, $f_1^{ABC}f_1^{BCD}$, and $\left(\left(f_1^{ABC}\right)^2+\left(f_1^{BCD}\right)^2\right)$, respectively, which are sums of second-order or higher-order products of angle-damping functions having $n \geq 1$. *Note:* $\left(f_1^{ABC}\right)^2 = f_1^{ABC}f_1^{ABC}$ and $f_1^{ABC}f_1^{BCD}$ are second-order products.) Accordingly, $U_{ABCD}^{ADLD}\left[\left\{\vec{R}_{H\neq G},\left(\vec{R}_G^0+\Delta X_G\right)\right\}\right]-U_{ABCD}^{ADLD}\left[\left\{\vec{R}_{H\neq G},\vec{R}_G^0\right\}\right]$ is a polynomial of ΔX_G such that the leading term has a power ≥ 2:

$$U_{ABCD}^{ADLD}\left[\left\{\vec{R}_{H\neq G},\left(\vec{R}_G^0+\Delta X_G\right)\right\}\right]-U_{ABCD}^{ADLD}\left[\left\{\vec{R}_{H\neq G},\vec{R}_G^0\right\}\right] \propto \left(\Delta X_G\right)^{t\geq 2}+\text{h.o.t.} \quad \text{(S148)}$$

where the higher-order terms (h.o.t.) have exponents higher than t. Substituting eqn (S148) into (S147) and taking the limit as $\Delta X_G \to 0$ yields $F_{G,x}^{dihedral\_ABCD}=0$. Because exactly the same argument also holds for $\Delta Y_G \to 0$ and $\Delta Z_G \to 0$, this proves eqn (S146).

Case # C: When only one of the included bond angles is linear (i.e., $\theta_{ABC}=\pi$ xor $\theta_{BCD}=\pi$)

Case # C1: Single-linear dihedral (i.e., $\theta_{ABC}^{eq}=\pi$ xor $\theta_{BCD}^{eq}=\pi$) with ($\theta_{ABC}=\pi$ xor $\theta_{BCD}=\pi$)

For reasons explained in Cases # C2 and # C3 below, only the n = (2(j=1) – 1) = 1 terms contribute to the atom-in-material force $\vec{F}_G^{dihedral\_ABCD}$ when one of the included bond angles is linear (i.e., $\theta_{ABC}=\pi$ xor $\theta_{BCD}=\pi$). As explained in Section S8 above, the n = (2(j=1) – 1) = 1 terms are zero for single-linear dihedrals. Therefore, the atom-in-material force is zero in this case

$$\vec{F}_G^{dihedral\_ABCD}=0 \quad \text{(S149)}$$

where G is any atom.

Case # C2: Double-linear dihedral (i.e., $\theta_{ABC}^{eq}=\pi$ and $\theta_{BCD}^{eq}=\pi$) with ($\theta_{ABC}=\pi$ and $0<\theta_{BCD}<\pi$)

In this case, $ж_{ABC}=0$, $0<ж_{BCD}<1$, $f_{n\geq 1}^{ABC}=0$, $0<f_{n\geq 1}^{BCD}<1$, and $f_0^{ABC}=f_0^{BCD}=1$. We can compute the force on each atom using the finite displacement method employing a small (i.e., infinitesimal) displacement. Displacing any atom $G \notin \{A,B,C,D\}$ does not change the values of $ж_{ABC}$ and $ж_{BCD}$; consequently, $f_{n\geq 1}^{ABC}=0$ remains and $\left\{f_n^{BCD}\right\}$ are unchanged. Examining eqn (S141), there is no change in U_{ABCD}^{ADLD} for such a displacement, and thus

$$\vec{F}_{G\notin\{A,B,C,D\}}^{dihedral\_ABCD}=0 \quad \text{(S150)}$$

Depending on the displacement direction, an extremely small finite displacement of atom A, B, or C could either leave the value of $ж_{ABC}$ unchanged at zero or change it to a value proportional to the displacement length, $\Delta\ell$. In this context, the phrase "value proportional to the displacement length, $\Delta\ell$" specifically means that if the extremely small displacement length is doubled along the same direction for the same atom then the total change in $ж_{ABC}$ also doubles. Examining eqn (S29) and (S33)–(S37), this means the value of f_n^{ABC} either remains unchanged at zero or it is changed to a value proportional to $(\Delta\ell)^n$.

The force of the displaced atom projected onto the displacement direction can be computed as

$$\vec{F}_{displaced\_atom}^{dihedral\_ABCD}\bullet\hat{d}=\lim_{\Delta\ell\to 0}-\frac{U_{ABCD}^{ADLD}[\text{displaced\_geom}]-U_{ABCD}^{ADLD}[\text{orig\_geom}]}{\Delta\ell} \quad \text{(S151)}$$

where $\hat{d}$ is the direction along which the displaced_atom is displaced and $\Delta\ell$ is the displacement distance. Examining eqn (S151), any terms in the numerator which happen to be proportional to $(\Delta\ell)^{t>1}$ do not contribute to the force. Any terms in the numerator which happen to be proportional

to $\Delta\ell$ can contribute to the force, because they are divided by a factor of $\Delta\ell$ in the denominator. As already stated above, upon extremely small finite displacement the value of $f_{n\geq 2}^{ABC}$ either remains unchanged or changes proportional to $(\Delta\ell)^{n\geq 2}$. Therefore, no potential energy terms proportional to $\left(f_{n\geq 2}^{ABC}\right)^{q>(1/n)}$ contribute to the force in Case # C2. For the same reason, any potential energy terms contributing to U_{ABCD}^{ADLD} that are proportional to $\left(f_1^{ABC}\right)^{q>1}$ do not contribute to the force in Case # C2. Stripping those negligible terms from the potential energy (eqn (S141)) results in only the following terms surviving for extremely small finite displacements:

$$U_{ABCD}^{ADLD}[\text{displaced\_geom}] - U_{ABCD}^{ADLD}[\text{orig\_geom}] = \underset{\sim}{f}_1^{ABC}\underset{\sim}{f}_1^{BCD}\left(\left(-k_{LD4}^1 + k_{LD5}^1\right)\cos\left[\underset{\sim}{\phi}\right] + k_{LD6}^1 S_{instance}\sin\left[\underset{\sim}{\phi}\right]\right) + \frac{1}{2}\left(\left(\underset{\sim}{f}_1^{BCD}\right)^2 - \left(f_1^{BCD}\right)^2\right)\left(k_{LD4}^1 + k_{LD5}^1\right) \tag{S152}$$

where orig_geom is the original geometry having $\theta_{ABC} = \pi$. In eqn (S152), $\underset{\sim}{f}_1^{ABC}$, $\underset{\sim}{f}_1^{BCD}$, and $\underset{\sim}{\phi}$ are the values computed for the displaced geometry, while f_1^{BCD} is for the original geometry.

If $k_{LD4}^1 = k_{LD5}^1 = k_{LD6}^1 = 0$, then it directly follows that $\vec{F}_A^{dihedral\_ABCD} = \vec{F}_B^{dihedral\_ABCD} = \vec{F}_C^{dihedral\_ABCD} = 0$. Otherwise, we proceed as follows. Using the trigonometric identity

$$\cos[\alpha - \beta] = \cos[\alpha]\cos[\beta] + \sin[\alpha]\sin[\beta] \tag{S153}$$

we can rewrite

$$\left(\left(-k_{LD4}^1 + k_{LD5}^1\right)\cos\left[\underset{\sim}{\phi}\right] + k_{LD6}^1 S_{instance}\sin\left[\underset{\sim}{\phi}\right]\right) = -\rho\cos\left[\underset{\sim}{\phi} - \Psi\right] \tag{S154}$$

where

$$\rho = +\sqrt{\left(-k_{LD4}^1 + k_{LD5}^1\right)^2 + \left(S_{instance}k_{LD6}^1\right)^2} \tag{S155}$$

$$\Psi = \arg\left[\frac{\left(k_{LD4}^1 - k_{LD5}^1\right) - k_{LD6}^1 S_{instance}\sqrt{-1}}{\rho}\right] = \text{atan2}\left[\left(-k_{LD6}^1 S_{instance}\right), \left(k_{LD4}^1 - k_{LD5}^1\right)\right] \tag{S156}$$

The argument function, $\arg\left[a + b\sqrt{-1}\right]$, is the phase angle of the complex number $a + b\sqrt{-1}$. This gives

$$U_{ABCD}^{ADLD}[\text{displaced\_geom}] - U_{ABCD}^{ADLD}[\text{orig\_geom}] = -\underset{\sim}{f}_1^{ABC}\underset{\sim}{f}_1^{BCD}\rho\cos\left[\underset{\sim}{\phi} - \Psi\right] + \frac{1}{2}\left(\left(\underset{\sim}{f}_1^{BCD}\right)^2 - \left(f_1^{BCD}\right)^2\right)\left(k_{LD4}^1 + k_{LD5}^1\right) \tag{S157}$$

<u>Let's now consider the displacement of atom A.</u> Since $\underset{\sim}{f}_1^{ABC}$ depends only on $ж_{ABC}$ for the displaced structure and not on $\underset{\sim}{\phi}$, it follows from eqn (S157) that the largest projected force magnitude is achieved by choosing a displacement direction that maximizes $\left|\cos\left[\underset{\sim}{\phi} - \Phi\right]\right|$. In other words, the projected force is largest in magnitude for $\underset{\sim}{\phi} = \Phi$ and $\underset{\sim}{\phi} = \Phi + \pi$. These correspond to two displacements in opposite directions that give rise to projected forces equal in magnitude with opposite signs. Displacing atom A does not alter f_n^{BCD}; thus, $\underset{\sim}{f}_1^{BCD} = f_1^{BCD}$. Making these substitutions into eqn (S157) gives the following for small finite displacement parallel to $\vec{F}_A^{dihedral\_ABCD}$:

$$U_{ABCD}^{ADLD}[\text{displaced\_geom}] - U_{ABCD}^{ADLD}[\text{orig\_geom}] = -\rho\underset{\sim}{f}_1^{ABC}f_1^{BCD} \tag{S158}$$

Substituting this into eqn (S151) gives

$$F_A^{dihedral\_ABCD} = \rho f_1^{BCD}\left(\lim_{\Delta\ell\to 0}\left(\frac{\underset{\sim}{f}_1^{ABC}}{\Delta\ell}\right)\right) \tag{S159}$$

Let $\varepsilon_{ABC} = \pi - \underset{\sim}{\theta}_{ABC}$ be the small (infinitesimal) angle produced upon displacing atom A perpendicular to the bond vector $\vec{R}_{BA}$. Then, the displaced distance is given by

$$\Delta\ell \approx \varepsilon_{ABC} R_{BA} \qquad \text{(S160)}$$

The kangal in the displaced geometry is

$$\underset{\sim}{ж}_{ABC} = \cos\left[\underset{\sim}{\theta}_{ABC}/2\right] = \cos\left[\left(\pi - \varepsilon_{ABC}\right)/2\right] = \sin\left[\varepsilon_{ABC}/2\right] \approx \varepsilon_{ABC}/2 \qquad \text{(S161)}$$

Examining eqn (S29) and (S33),

$$\lim_{\underset{\sim}{ж}_{ABC} \to \text{infinitesimal}} \underset{\sim}{f}_1^{ABC} = \frac{\tanh\left[K\underset{\sim}{ж}_{ABC}/4\right]}{\text{tanh\_K}} = \frac{K\underset{\sim}{ж}_{ABC}}{4\text{tanh\_K}} = \frac{K\varepsilon_{ABC}}{8\text{tanh\_K}} \qquad \text{(S162)}$$

Substituting eqn (S160) and (S162) into (S159) gives

$$F_A^{\text{dihedral\_ABCD}} = \rho f_1^{BCD}\left(\frac{K}{8\text{tanh\_K}}\right)\frac{1}{R_{BA}} \qquad \text{(S163)}$$

The direction of this force is computed as follows. Using eqn (S80) and (S81), we compute the unit vector $\hat{v}$ that is perpendicular to $\vec{R}_{BC}$ that is in the same plane as $\vec{R}_{CD}$ and $\vec{R}_{BC}$. Using eqn (S14) and (S18), we compute the unit vector $\hat{u}$ that is perpendicular to both $\vec{R}_{BC}$ and $\vec{R}_{CD}$. It follows from basic geometry that the direction of the force is then given by

$$\hat{F}_A^{\text{dihedral\_ABCD}} = \hat{v}\cos[\Psi] + \hat{u}\sin[\Psi] \qquad \text{(S164)}$$

Finally, the magnitude from eqn (S163) and the direction from eqn (S164) are combined to give the force vector:

$$\vec{F}_A^{\text{dihedral\_ABCD}} = \rho f_1^{BCD}\left(\frac{K}{8\text{tanh\_K}}\right)\frac{1}{R_{BA}}\left(\hat{v}\cos[\Psi] + \hat{u}\sin[\Psi]\right) \qquad \text{(S165)}$$

<u>Let's now consider the displacement of atom D</u>. Since $f_{n\geq1}^{ABC}$ depends only on the positions of atoms A, B, and C, its value is not changed by a displacement of atom D. Thus, we have $\underset{\sim}{f}_{n\geq1}^{ABC} = f_{n\geq1}^{ABC} = 0$. Substituting this into the ADLD potential (eqn (S141)) shows that the only surviving non-zero term is $½\left(f_1^{BCD}\right)^2\left(k_{LD4}^1 + k_{LD5}^1\right)$. Therefore, in this case

$$\vec{F}_D^{\text{dihedral\_ABCD}} = -\frac{\partial U_{ABCD}^{ADLD}\left[ж_{ABC}, ж_{BCD}, \phi_{ABCD}\right]}{\partial ж_{BCD}}\vec{\nabla}_D ж_{BCD} = -\left(k_{LD4}^1 + k_{LD5}^1\right) f_1^{BCD}\left(\frac{df_1^{BCD}}{dж_{BCD}}\right)\vec{\nabla}_D ж_{BCD} \qquad \text{(S166)}$$

where $df_1^{BCD}/dж_{BCD}$ is given by eqn (S38) and $\vec{\nabla}_D ж_{BCD}$ is given by eqn (S7).

<u>Let's now consider the displacement of atom C</u>. For atom C, we separate changes in the potential energy and the force into two parts. We re-write eqn (S152) as

$$U_{ABCD}^{ADLD}[\text{displaced\_geom}] - U_{ABCD}^{ADLD}[\text{orig\_geom}] = \Delta U_{\text{part\_1}} + \Delta U_{\text{part\_2}} \qquad \text{(S167)}$$

$$\Delta U_{\text{part\_1}} = -\underset{\sim}{f}_1^{ABC}\underset{\sim}{f}_1^{BCD}\rho\cos\left[\underset{\sim}{\phi} - \Psi\right] \qquad \text{(S168)}$$

$$\Delta U_{\text{part\_2}} = \frac{1}{2}\left(\left(\underset{\sim}{f}_1^{BCD}\right)^2 - \left(f_1^{BCD}\right)^2\right)\left(k_{LD4}^1 + k_{LD5}^1\right) \qquad \text{(S169)}$$

$$\vec{F}_C^{\text{dihedral\_ABCD}} = \vec{F}_C^{\text{part\_1}} + \vec{F}_C^{\text{part\_2}} \qquad \text{(S170)}$$

$\vec{F}_C^{\text{part\_1}}$ and $\vec{F}_C^{\text{part\_2}}$ projected onto the displacement direction can be computed as

$$\vec{F}_C^{\text{part\_1}} \bullet \hat{d} = \lim_{\Delta\ell \to 0} -\frac{\Delta U_{\text{part\_1}}}{\Delta\ell} = \lim_{\Delta\ell \to 0}\frac{\underset{\sim}{f}_1^{ABC}\underset{\sim}{f}_1^{BCD}\rho\cos\left[\underset{\sim}{\phi} - \Psi\right]}{\Delta\ell} \qquad \text{(S171)}$$

$$\vec{F}_C^{\text{part\_2}} \bullet \hat{d} = \lim_{\Delta\ell \to 0} -\frac{\Delta U_{\text{part\_2}}}{\Delta\ell} = \lim_{\Delta\ell \to 0} -\left(k_{LD4}^1 + k_{LD5}^1\right)\frac{\left(\left(\underset{\sim}{f}_1^{BCD}\right)^2 - \left(f_1^{BCD}\right)^2\right)}{2\Delta\ell} \qquad \text{(S172)}$$

where $\hat{d}$ is the direction along which the displaced_atom is displaced and $\Delta\ell$ is the displacement distance. Using the definition of the first derivative, eqn (S172) can be re-written as

$$\vec{F}_C^{part\_2} = -\frac{\partial\left(½\left(k_{LD4}^1 + k_{LD5}^1\right)\left(f_1^{BCD}\right)^2\right)}{\partial ж_{BCD}}\vec{\nabla}_C ж_{BCD} = -\left(k_{LD4}^1 + k_{LD5}^1\right) f_1^{BCD}\left(\frac{df_1^{BCD}}{dж_{BCD}}\right)\vec{\nabla}_C ж_{BCD} \quad \text{(S173)}$$

Since $\underset{\sim}{f}_1^{ABC}$ depends only on $ж_{ABC}$ for the displaced structure and not on $\underset{\sim}{\phi}$, it follows from eqn (S171) that the largest projected force magnitude of part_1 is achieved by choosing a displacement direction that maximizes $\left|\cos\left[\underset{\sim}{\phi} - \Phi\right]\right|$. In other words, the projected force in eqn (S171) is largest in magnitude for $\underset{\sim}{\phi} = \Phi$ and $\underset{\sim}{\phi} = \Phi + \pi$. These correspond to two displacements in opposite directions that give rise to projected forces equal in magnitude with opposite signs.

We have the following for infinitesimal displacement away from a linear ABC angle:

$$\underset{\sim}{f}_1^{ABC} = \underset{0}{f_1^{ABC}} + df_1^{ABC} = df_1^{ABC} \quad \text{(S174)}$$

$$\underset{\sim}{f}_1^{BCD} = f_1^{BCD} + df_1^{BCD} \quad \text{(S175)}$$

$$\underset{\sim}{f}_1^{ABC}\underset{\sim}{f}_1^{BCD} = df_1^{ABC}\left(f_1^{BCD} + df_1^{BCD}\right) = df_1^{ABC} f_1^{BCD} + \underbrace{df_1^{ABC} df_1^{BCD}}_{\text{negligible}} \approx \underset{\sim}{f}_1^{ABC} f_1^{BCD} \quad \text{(S176)}$$

The term marked as negligible can be neglected, because it is the product of two infinitesimal changes. Substituting eqn (S176) into (S171) gives

$$F_C^{part\_1} = \rho f_1^{BCD} \lim_{\Delta\ell \to 0} \frac{\underset{\sim}{f}_1^{ABC}}{\Delta\ell} \quad \text{(S177)}$$

Let $\varepsilon_{ABC} = \pi - \underset{\sim}{\theta}_{ABC}$ be the small (infinitesimal) angle produced upon displacing atom C perpendicular to the bond vector $\vec{R}_{BC}$. Then, the displaced distance is given by

$$\Delta\ell \approx \varepsilon_{ABC} R_{BC} \quad \text{(S178)}$$

The kangal in the displaced geometry is

$$\underset{\sim}{ж}_{ABC} = \cos\left[\underset{\sim}{\theta}_{ABC}/2\right] = \cos\left[\left(\pi - \varepsilon_{ABC}\right)/2\right] = \sin\left[\varepsilon_{ABC}/2\right] \approx \varepsilon_{ABC}/2 \quad \text{(S179)}$$

Examining eqn (S29) and (S33),

$$\lim_{ж_{ABC} \to \text{infinitesimal}} \underset{\sim}{f}_1^{ABC} = \frac{\tanh\left[K\underset{\sim}{ж}_{ABC}/4\right]}{\text{tanh\_K}} = \frac{K\underset{\sim}{ж}_{ABC}}{4\text{tanh\_K}} = \frac{K\varepsilon_{ABC}}{8\text{tanh\_K}} \quad \text{(S180)}$$

Substituting eqn (S178) and (S180) into (S177) gives

$$F_C^{part\_1} = \rho f_1^{BCD}\left(\frac{K}{8\text{tanh\_K}}\right)\frac{1}{R_{BC}} \quad \text{(S181)}$$

The direction of $\vec{F}_C^{part\_1}$ is computed as follows. Using eqn (S80) and (S81), we compute the unit vector $\hat{v}$ that is perpendicular to $\vec{R}_{BC}$ that is in the same plane as $\vec{R}_{CD}$ and $\vec{R}_{BC}$. Using eqn (S14) and (S18), we compute the unit vector $\hat{u}$ that is perpendicular to both $\vec{R}_{BC}$ and $\vec{R}_{CD}$. It follows from basic geometry that the direction of the force is then given by

$$\hat{F}_C^{part\_1} = \hat{v}\cos[\Psi] + \hat{u}\sin[\Psi] \quad \text{(S182)}$$

The magnitude from eqn (S181) and the direction from eqn (S182) are combined to give:

$$\vec{F}_C^{part\_1} = \rho f_1^{BCD}\left(\frac{K}{8\text{tanh\_K}}\right)\frac{1}{R_{BC}}\left(\hat{v}\cos[\Psi] + \hat{u}\sin[\Psi]\right) \quad \text{(S183)}$$

Finally, eqn (S173) and (S183) are inserted into eqn (S170) to give:

$$\vec{F}_{C}^{dihedral\_ABCD} = \rho f_1^{BCD}\left(\frac{K}{8tanh\_K}\right)\frac{1}{R_{BC}}\left(\hat{v}\cos[\Psi]+\hat{u}\sin[\Psi]\right)-\left(k_{LD4}^1+k_{LD5}^1\right)f_1^{BCD}\left(\frac{df_1^{BCD}}{dж_{BCD}}\right)\vec{\nabla}_C ж_{BCD} \quad (S184)$$

<u>Let's now consider the displacement of atom B.</u> Because U_{ABCD}^{ADLD} is invariant to a rigid translation of atoms A, B, C, and D, it follows that the associated forces sum to zero:

$$\vec{F}_{A}^{dihedral\_ABCD}+\vec{F}_{B}^{dihedral\_ABCD}+\vec{F}_{C}^{dihedral\_ABCD}+\vec{F}_{D}^{dihedral\_ABCD}=0 \quad (S185)$$

Rearranging this equation gives

$$\vec{F}_{B}^{dihedral\_ABCD}=-\left(\vec{F}_{A}^{dihedral\_ABCD}+\vec{F}_{C}^{dihedral\_ABCD}+\vec{F}_{D}^{dihedral\_ABCD}\right) \quad (S186)$$

Case # C3: Double-linear dihedral (i.e., $\theta_{ABC}^{eq}=\pi$ and $\theta_{BCD}^{eq}=\pi$) with ($0<\theta_{ABC}<\pi$ and $\theta_{BCD}=\pi$)

If we make the appropriate substitutions of atoms, this case is analogous to Case # C2 and has the following equations:

$$\rho=+\sqrt{\left(-k_{LD4}^1+k_{LD5}^1\right)^2+\left(S_{instance}k_{LD6}^1\right)^2} \quad (S187)$$

$$\Psi=\arg\left[\frac{\left(k_{LD4}^1-k_{LD5}^1\right)-k_{LD6}^1 S_{instance}\sqrt{-1}}{\rho}\right]=atan2\left[\left(-k_{LD6}^1 S_{instance}\right),\left(k_{LD4}^1-k_{LD5}^1\right)\right] \quad (S188)$$

Using eqn (S107) and (S108), define $\hat{w}$ as a unit vector that is perpendicular to $\vec{R}_{BC}$ that is in the same plane as $\vec{R}_{AB}$ and $\vec{R}_{BC}$. Using eqn (S13) and (S16), define $\hat{t}$ as a unit vector that is perpendicular to both $\vec{R}_{BC}$ and $\vec{R}_{AB}$.

The forces are then given by

$$\vec{F}_{G\notin\{A,B,C,D\}}^{dihedral\_ABCD}=0 \quad (S189)$$

$$\vec{F}_{A}^{dihedral\_ABCD}=-\frac{\partial U_{ABCD}^{ADLD}[ж_{ABC},ж_{BCD},\phi_{ABCD}]}{\partial ж_{ABC}}\vec{\nabla}_A ж_{ABC}=-\left(k_{LD4}^1+k_{LD5}^1\right)f_1^{ABC}\left(\frac{df_1^{ABC}}{dж_{ABC}}\right)\vec{\nabla}_A ж_{ABC} \quad (S190)$$

$$\vec{F}_{D}^{dihedral\_ABCD}=\rho f_1^{ABC}\left(\frac{K}{8tanh\_K}\right)\frac{1}{R_{CD}}\left(\hat{w}\cos[\Psi]-\hat{t}\sin[\Psi]\right) \quad (S191)$$

$$\vec{F}_{B}^{dihedral\_ABCD}=\rho f_1^{ABC}\left(\frac{K}{8tanh\_K}\right)\frac{1}{R_{BC}}\left(\hat{w}\cos[\Psi]-\hat{t}\sin[\Psi]\right)-\left(k_{LD4}^1+k_{LD5}^1\right)f_1^{ABC}\left(\frac{df_1^{ABC}}{dж_{ABC}}\right)\vec{\nabla}_B ж_{ABC} \quad (S192)$$

Because U_{ABCD}^{ADLD} is invariant to a rigid translation of atoms A, B, C, and D, it follows that the associated forces sum to zero:

$$\vec{F}_{A}^{dihedral\_ABCD}+\vec{F}_{B}^{dihedral\_ABCD}+\vec{F}_{C}^{dihedral\_ABCD}+\vec{F}_{D}^{dihedral\_ABCD}=0 \quad (S193)$$

Rearranging this equation gives

$$\vec{F}_{C}^{dihedral\_ABCD}=-\left(\vec{F}_{A}^{dihedral\_ABCD}+\vec{F}_{B}^{dihedral\_ABCD}+\vec{F}_{D}^{dihedral\_ABCD}\right) \quad (S194)$$

S10. Supplementary tables and supplementary figure

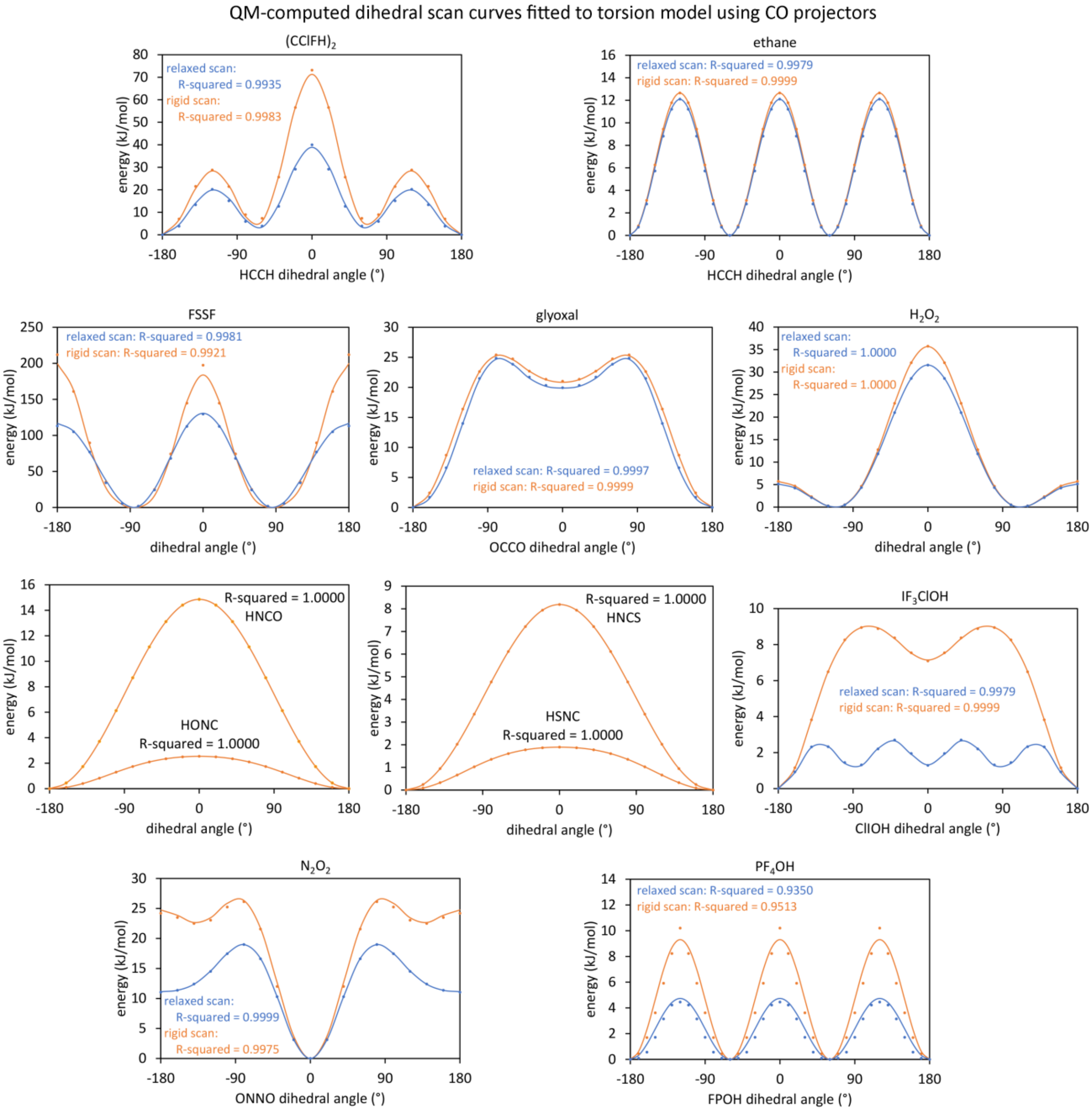

Figure S1: Example dihedral scan energy curves in twelve molecules for which the dihedral has sym_value = 0. The y-axis plots the energy relative to the low energy conformation. The filled circles show the QM-computed (CCSD/def2-TZVPD) values. The solid lines show the fitted model potential of eqn (158)–(159) of the main text using the CO projectors (eqn (144)–(145) of the main text) with the parameters from Table 8 of the main text. Bond angles and bond lengths were held fixed to generate the results shown in orange. Bond angles and bond lengths were relaxed to generate the results (where available) shown in blue. The SumCSq values are listed in Table 8 of the main text.

Table S1: Calculated value of Δ_n in 12 molecules for which the dihedral has sym_value = 0. This value being zero (within a round-off error) shows that if sym_value = 0 then the torsion offset potential (TOP) is the same using the CO projectors as when using the DT projectors. Values outside parentheses are for rigid torsion scan. Values inside parentheses are for relaxed torsion scan. For HNCO, HNCS, HONC, and HSNC, the values inside square brackets relaxed the bond lengths but not the bond angles.

**molecule**	**dihedral**	**mode 1**	**mode 2**	**mode 3**	**mode 4**
$(CClFH)_2$	HCCH	0.0000 (0.0000)	0.0000 (0.0000)	0.0000 (0.0000)	0.0000 (0.0000)
ethane	HCCH	0.0000 (0.0000)	0.0000 (0.0000)	0.0000 (0.0000)	0.0000 (0.0000)
FSSF	FSSF	0.0000 (0.0000)	0.0000 (-0.0001)	0.0000 (0.0000)	0.0000 (0.0001)
glyoxal	OCCO	0.0000 (0.0000)	0.0000 (0.0000)	0.0000 (0.0000)	0.0000 (0.0000)
H_2O_2	HOOH	0.0000 (0.0000)	0.0001 (0.0000)	0.0000 (0.0000)	0.0000 (0.0000)
HNCO	HNCO	0.0000 [0.0000]	0.0000 [0.0000]	0.0000 [0.0000]	0.0000 [0.0000]
HNCS	HNCS	0.0000 [0.0000]	0.0000 [0.0000]	0.0000 [0.0000]	0.0000 [0.0000]
HONC	HONC	0.0000 [0.0000]	0.0000 [0.0000]	0.0000 [0.0000]	0.0000 [0.0000]
HSNC	HSNC	0.0000 [0.0000]	0.0000 [0.0000]	0.0000 [0.0000]	0.0000 [0.0000]
IF_3ClOH	ClIOH	0.0000 (0.0000)	0.0000 (0.0000)	0.0000 (0.0000)	0.0000 (0.0000)
N_2O_2	ONNO	0.0000 (0.0000)	0.0000 (0.0000)	0.0000 (0.0000)	0.0000 (0.0000)
PF_4OH	FPOH	0.0000 (0.0000)	0.0000 (0.0000)	0.0000 (0.0000)	0.0000 (0.0000)

Table S2: QM-computed torsion mode coefficients using the CO projectors for four molecules that have at least one equilibrium bond angle ≥ 130° and sym_value=0. ADCO and CACO model predictions are listed in the last four columns.

molecule	θ_{constr} (°)	c_1	c_2	c_3	c_4	SumCSq	ADCO predicted ϕ_{min} (°)	ADCO predicted norm (kJ/mol)	ADCO predicted norm ratio	CACO prediction equal to optimized norm (kJ/mol)
HNCO	125.0	0.9994	-0.0143	0.0308	0.0011	1.0000	180.0	63.59	12.17	5.23
HNCO	140.0	0.9999	-0.0070	0.0119	0.0012	1.0000	180.0	38.98	7.46	5.23
HNCO	155.0	1.0000	-0.0035	0.0035	0.0000	1.0000	180.0	20.77	3.98	5.23
HNCO	165.0	1.0000	-0.0019	0.0012	-0.0001	1.0000	180.0	11.47	2.20	5.23
HNCS	165.0	1.0000	0.0040	-0.0004	-0.0005	1.0000	180.0	8.11	2.81	2.88
HONC	125.0	0.8816	-0.4718	0.0089	-0.0022	1.0000	180.0	12.04	13.35	0.90
HONC	140.0	0.8985	-0.4389	0.0072	-0.0003	1.0000	180.0	7.47	8.29	0.90
HONC	155.0	0.9438	-0.3304	0.0026	-0.0003	1.0000	180.0	3.91	4.33	0.90
HONC	165.0	0.9764	-0.2160	0.0010	-0.0002	1.0000	180.0	2.12	2.35	0.90
HSNC	125.0	0.6597	-0.7514	-0.0107	-0.0059	1.0000	180.0	10.48	15.58	0.67
HSNC	140.0	0.7672	-0.6414	0.0039	-0.0013	1.0000	180.0	6.44	9.57	0.67
HSNC	155.0	0.8847	-0.4661	0.0031	-0.0001	1.0000	180.0	3.27	4.87	0.67
HSNC	165.0	0.9530	-0.3029	0.0014	0.0000	1.0000	180.0	1.74	2.58	0.67

Table S3: Optimized CADT force constants (eV) for 2-amino-propanal

**dihedral**	**CADT mode**	**forcefield excluding all nonbonded interactions**	**forcefield excluding 1-2, 1-3, and 1-4 nonbonded interactions**	**forcefield excluding 1-2 and 1-3 nonbonded interactions**
HCCH	3	0.05317	0.05348	0.04623
HCCH	5	-0.00408	-0.00800	-0.01830
HCCH	7	-0.01057	-0.00879	-0.01204
HNCC	1	0.04068	0.00035	0.01026
HNCC	2	-0.01234	0.00098	-0.00305
HNCC	3	0.03508	0.02841	0.02631
HNCC	5	0.00435	0.00564	0.00068
HNCC	6	-0.00197	0.00271	0.00078
HNCC	7	-0.02341	-0.02326	-0.01986
OCCN	1	-0.01171	-0.06225	-0.03731
OCCN	2	0.03442	0.05246	0.03708
OCCN	3	0.03211	0.03201	0.03580
OCCN	4	0.00492	0.00351	0.00479
OCCN	5	0.01193	0.02250	0.01539
OCCN	6	-0.00226	-0.00829	-0.00459

Table S4: Optimized CACO torsion force constants k_{CACO} (eV) and mode coefficients c_n (dimensionless) for 2-amino-propanal

**dihedral**	**constant**	**forcefield excluding all nonbonded interactions**	**forcefield excluding 1-2, 1-3, and 1-4 nonbonded interactions**	**forcefield excluding 1-2 and 1-3 nonbonded interactions**
HCCH	k_{CACO}	0.06262	0.06173	0.05598
HCCH	c_1	0.000000	0.000000	0.000000
HCCH	c_2	0.000000	0.000000	0.000000
HCCH	c_3	0.999286	0.999286	0.999286
HCCH	c_4	0.000000	0.000000	0.000000
HNCC	k_{CACO}	0.05899	0.00765	0.01867
HNCC	c_1	0.848580	0.848580	0.848580
HNCC	c_2	0.420023	0.420023	0.420023
HNCC	c_3	0.269624	0.269624	0.269624
HNCC	c_4	0.013052	0.013052	0.013052
OCCN	k_{CACO}	0.04780	0.02939	0.03534
OCCN	c_1	-0.172963	-0.172963	-0.172963
OCCN	c_2	-0.377461	-0.377461	-0.377461
OCCN	c_3	-0.848237	-0.848237	-0.848237
OCCN	c_4	-0.122084	-0.122084	-0.122084

S11. Validation of analytic derivatives and forces formulas

All of the analytic derivatives and force formulas presented in Sections S1 to S9 above were validated through comparisons to numeric forces and derivatives computed using the central finite difference approximation. The Matlab codes and their output results for these tests are found in the test_derivatives_and_forces subfolder of the enclosed ESI zip archive. The following were explicitly tested by comparing results from the analytic formulas to those computed using the central finite difference approximation:

- Gradients of the kangal $ж_{\mathrm{ABC}}$ when $ж_{\mathrm{ABC}} > 0$ (i.e., when $0 < \theta_{\mathrm{ABC}} < \pi$)
- Gradients of the kangal $ж_{\mathrm{BCD}}$ when $ж_{\mathrm{BCD}} > 0$ (i.e., when $0 < \theta_{\mathrm{BCD}} < \pi$)
- Gradients of the dihedral ϕ_{ABCD} when $(\theta_{\mathrm{ABC}} \text{ and } \theta_{\mathrm{BCD}}) \neq \pi$
- Atom-in-material forces for the CADT model potential when $(\theta_{\mathrm{ABC}} \text{ and } \theta_{\mathrm{BCD}}) \neq \pi$ and $(\theta^{\mathrm{eq}}_{\mathrm{ABC}} \text{ and } \theta^{\mathrm{eq}}_{\mathrm{BCD}}) \neq \pi$
- Atom-in-material forces for the CACO model potential when $(\theta_{\mathrm{ABC}} \text{ and } \theta_{\mathrm{BCD}}) \neq \pi$ and $(\theta^{\mathrm{eq}}_{\mathrm{ABC}} \text{ and } \theta^{\mathrm{eq}}_{\mathrm{BCD}}) \neq \pi$
- Atom-in-material forces for the ADDT model potential when $(\theta^{\mathrm{eq}}_{\mathrm{ABC}} \text{ and } \theta^{\mathrm{eq}}_{\mathrm{BCD}}) \neq \pi$ for each of the following cases:
    - Case #1: When neither included bond angle is linear (i.e., $(0 < \theta_{\mathrm{ABC}} < \pi)$ and $(0 < \theta_{\mathrm{BCD}} < \pi)$)
    - Case #2: When both included bond angles are linear (i.e., $(\theta_{\mathrm{ABC}} = \pi)$ and $(\theta_{\mathrm{BCD}} = \pi)$)
    - Case #3: When only one of the included bond angles is linear (i.e., $(\theta_{\mathrm{ABC}} = \pi)$ xor $(\theta_{\mathrm{BCD}} = \pi)$)
        - Case # 3a: $(\theta_{\mathrm{ABC}} = \pi)$ and $(0 < \theta_{\mathrm{BCD}} < \pi)$
        - Case # 3b: $(0 < \theta_{\mathrm{ABC}} < \pi)$ and $(\theta_{\mathrm{BCD}} = \pi)$
- Atom-in-material forces for the ADCO model potential when $(\theta^{\mathrm{eq}}_{\mathrm{ABC}} \text{ and } \theta^{\mathrm{eq}}_{\mathrm{BCD}}) \neq \pi$ for each of the following cases:
    - Case #1: When neither included bond angle is linear (i.e., $(0 < \theta_{\mathrm{ABC}} < \pi)$ and $(0 < \theta_{\mathrm{BCD}} < \pi)$)
    - Case #2: When both included bond angles are linear (i.e., $(\theta_{\mathrm{ABC}} = \pi)$ and $(\theta_{\mathrm{BCD}} = \pi)$)
    - Case #3: When only one of the included bond angles is linear (i.e., $(\theta_{\mathrm{ABC}} = \pi)$ xor $(\theta_{\mathrm{BCD}} = \pi)$)
        - Case # 3a: $(\theta_{\mathrm{ABC}} = \pi)$ and $(0 < \theta_{\mathrm{BCD}} < \pi)$
        - Case # 3b: $(0 < \theta_{\mathrm{ABC}} < \pi)$ and $(\theta_{\mathrm{BCD}} = \pi)$
- Atom-in-material forces for the ADLD model potential for each of the following cases:
    - Case # A: When neither included bond angle is linear (i.e., $(0 < \theta_{\mathrm{ABC}} < \pi)$ and $(0 < \theta_{\mathrm{BCD}} < \pi)$)
    - Case # B: When both included bond angles are linear (i.e., $(\theta_{\mathrm{ABC}} = \pi)$ and $(\theta_{\mathrm{BCD}} = \pi)$)

- Case # C: When only one of the included bond angles is linear (i.e., $(\theta_{ABC} = \pi)$ xor $(\theta_{BCD} = \pi)$)
  - Case # C1: Single-linear dihedral (i.e., $(\theta_{ABC}^{eq} = \pi)$ xor $(\theta_{BCD}^{eq} = \pi)$) with ($(\theta_{ABC} = \pi)$ xor $(\theta_{BCD} = \pi)$)
  - Case # C2: Double-linear dihedral (i.e., $(\theta_{ABC}^{eq} = \pi)$ and $(\theta_{BCD}^{eq} = \pi)$) with ($(\theta_{ABC} = \pi)$ and $(0 < \theta_{BCD} < \pi)$)
  - Case # C3: Double-linear dihedral (i.e., $(\theta_{ABC}^{eq} = \pi)$ and $(\theta_{BCD}^{eq} = \pi)$) with ($(0 < \theta_{ABC} < \pi)$ and $(\theta_{BCD} = \pi)$)

Each test set included more than 100 distinct geometries that sampled all degrees of freedom. A full coverage of all degrees of freedom was rigorously ensured by generating test geometries via independent random displacements of all atoms in the molecule along the XYZ directions across a test dataset that spanned various values of the bond lengths, bond angles, and dihedral value. All test geometries were subsequently rigidly rotated by multiplying their XYZ coordinates by a random rotation matrix. All dihedral force constants were set to independent random values between -0.5 and +0.5 using the uniform real-valued random number generator (rand[1] – 0.5) in Matlab, except force constants for the ADLD cosine modes were set to independent random non-negative values between 0.0 and +1.0. For the ADDT and CADT potentials, $S_{instance}$ was randomly selected to be -1, 0, or +1. Ten runs were performed for each model potential. Each run generated new sets of atom-in-material XYZ random displacements, random rotation matrix, random dihedral force constants, and where applicable random $S_{instance}$ values. For the ADDT, ADCO, and ADLD model potentials, geometries were included to cover each of the distinct cases enumerated in the bulleted list above.

The analytic derivatives and forces formulas were validated by computing norm[numeric_values_array – analytic_values_array] and max[abs[numeric_values_array – analytic_values_array]] and comparing these to norm[numeric_values_array] and norm[analytic_values_array]. The results for each run of each model potential showed values computed using the analytic formulas were in excellent agreement with the numerically-computed gradients and forces, when using a finite-difference step size of 0.0001. The analytically-computed and numerically-computed forces almost always differed by less than one part per million and still agreed closely when their difference was somewhat higher than this. This clearly demonstrates the analytic derivatives and forces formulas presented in Sections S1 to S9 above are correct.

These enclosed computer codes serve the critically important function of being a reference implementation that programmers can translate into different computer languages. Programmers can then check the results of their translated routines against the results of this reference implementation.

S.12 Derivation of torsion scan R-squared formulae

For a rotatable dihedral, the potential energy along a torsion scan curve can be modeled by projecting onto an orthonormal basis of independent torsion modes:

$$\int_{-\pi}^{\pi} \left(\frac{P_m^{DT}[\phi]}{\sqrt{\pi}} \right) \left(\frac{P_n^{DT}[\phi]}{\sqrt{\pi}} \right) d\phi = \delta_{m,n}^{Kronecker} \quad \text{(S195)}$$

$$\int_{-\pi}^{\pi} \left(\frac{P_m^{CO}[\phi]}{\sqrt{\pi}} \right) \left(\frac{P_n^{CO}[\phi]}{\sqrt{\pi}} \right) d\phi = \delta_{m,n}^{Kronecker} \quad \text{(S196)}$$

"According to the Sampling Theorem that was developed by several pioneers of signal transmission, a continuous signal can be transmitted and recovered without aliasing (also called wrap-around or folding) errors if it is sampled at least as frequently as twice the highest frequency component of the signal.[S4-S6] This minimum sampling rate is called the Nyquist rate.[S4,S5,S7,S8] This means eqn (S195) and (S196) can be equivalently rewritten as a sum over an equally spaced grid of ϕ values

$$\left(\frac{2}{T}\right)\sum_{j=1}^{T} P_m^{DT}[\phi]P_n^{DT}[\phi] = \delta_{m,n}^{Kronecker} \tag{S197}$$

$$\left(\frac{2}{T}\right)\sum_{j=1}^{T} P_m^{CO}[\phi]P_n^{CO}[\phi] = \delta_{m,n}^{Kronecker} \tag{S198}$$

The maximum frequency of the product of two sine and/or cosine functions equals the sum of their frequencies. Thus, to achieve correct sampling for projectors containing sine and cosine components up to multiplicity n_{max} (e.g., $\cos[n_{max}\phi]$, $\cos\left[n_{max}\left(\phi-\phi_{eq}\right)\right]$, $\sin\left[n_{max}\left(\phi-\phi_{eq}\right)\right]$) eqn (S197) and (S198) hold exactly if we choose any $T \geq 4n_{max}$. For $n_{max} = 4$, this means T can be chosen as any whole number ≥ 16.

Let $E_{RTS}^{QM}[\phi]$ be the QM-computed energy of the material along the torsion scan curve for rotatable dihedral ABCD. This torsion scan curve is conducted using T dihedral values equally spaced over the range $(-\pi,\pi]$. Let $E_{RTS}^{QM\_avg}$ be the average value

$$E_{RTS}^{QM\_avg} = \frac{1}{T}\sum_{j=1}^{T} E_{RTS}^{QM}\left[\phi_j\right] \tag{S199}$$

and w the self-overlap integral

$$w = \int_{-\pi}^{\pi}\left(E_{RTS}^{QM}[\phi]-E_{RTS}^{QM\_avg}\right)^2 d\phi \approx \left(\frac{2\pi}{T}\right)\sum_{j=1}^{T}\left(E_{RTS}^{QM}\left[\phi_j\right]-E_{RTS}^{QM\_avg}\right)^2 \tag{S200}$$

The 'QM torsion norm' is the root-mean-squared value of $\left(E_{RTS}^{QM}[\phi]-E_{RTS}^{QM\_avg}\right)$:

$$\text{torsion\_norm}_{QM} = \sqrt{\left(\frac{1}{T}\right)\sum_{j=1}^{T}\left(E_{RTS}^{QM}\left[\phi_j\right]-E_{RTS}^{QM\_avg}\right)^2} = \sqrt{\frac{w}{2\pi}} \tag{S201}$$

Using a complete set of DT projectors, the QM potential for the torsion scan curve can be expanded in terms of the orthogonal basis set as

$$E_{RTS}^{QM}[\phi] = E_{RTS}^{QM\_avg} + \sqrt{w}\sum_{m=1}^{\infty} c_m^{DT}\frac{P_m^{DT}[\phi]}{\sqrt{\pi}} \tag{S202}$$

Since the CO projectors are even functions of ϕ, the CO projectors can provide a complete expansion of the QM potential along the torsion scan curve iff the following symmetry descriptor

$$\text{sym\_value} = \frac{1}{2}\sqrt{\frac{\sum_{j=1}^{T}\left(E_{RTS}^{QM}\left[\phi_j\right]-E_{RTS}^{QM}\left[-\phi_j\right]\right)^2}{\sum_{j=1}^{T}\left(E_{RTS}^{QM}\left[\phi_j\right]-E_{RTS}^{QM\_avg}\right)^2}} \tag{S203}$$

equals zero. Iff sym_value equals zero, then in this case the QM potential can be expanded using the CO projectors as

$$E_{RTS}^{QM}[\phi] = E_{RTS}^{QM\_avg} + \sqrt{w}\sum_{m=1}^{\infty} c_m^{CO}\frac{P_m^{CO}[\phi]}{\sqrt{\pi}} \tag{S204}$$

The model potential for the torsion scan curve can be expanded in terms of the orthogonal basis set as

$$E_{RTS}^{model}[\phi] = E_{RTS}^{model\_avg} + \sqrt{w}\sum_{m=1}^{m_{max}} c_m \frac{P_m[\phi]}{\sqrt{\pi}} \quad (S205)$$

$$E_{RTS}^{model}[\phi] - E_{RTS}^{model}\left[\phi_{eq}^{training}\right] = \sqrt{w}\sum_{m=1}^{m_{max}} c_m \frac{G_m[\phi]}{\sqrt{\pi}} \quad (S206)$$

where

$$E_{RTS}^{model\_avg} = \frac{1}{T}\sum_{j=1}^{T} E_{RTS}^{model}\left[\phi_j\right] \quad (S207)$$

For the ADDT and CADT model potentials, $P_m = P_m^{DT}$ is used in eqn (S205) and (S208), and $G_m = G_m^{DT}$ is used in eqn (S206). For the ADCO and CACO model potentials, $P_m = P_m^{CO}$ is used in eqn (S205) and (S208), and $G_m = G_m^{CO}$ is used in eqn (S206). The coefficients are given by

$$c_m = \int_{-\pi}^{\pi} \frac{P_m[\phi]}{\sqrt{\pi}}\left(\frac{E_{RTS}^{QM}[\phi] - E_{RTS}^{QM\_avg}}{\sqrt{w}}\right)d\phi \approx \left(\frac{2\pi}{T}\right)\sum_{j=1}^{T}\frac{P_m[\phi_j]}{\sqrt{\pi}}\left(\frac{E_{RTS}^{QM}\left[\phi_j\right] - E_{RTS}^{QM\_avg}}{\sqrt{w}}\right) \quad (S208)$$

For the torsion modes included in the model potential, the expansion coefficients $\{c_m\}$ are the same for the QM and model potentials along the torsion scan curve. The model potential neglects some of the less important (i.e., negligible) torsion modes while the QM potential is formally expanded (see eqn (S202)) as a nontruncated sum over all possible torsion modes.

The 'model torsion norm' is the root-mean-squared value of $\left(E_{RTS}^{model}\left[\phi_j\right] - E_{RTS}^{model\_avg}\right)$:

$$torsion\_norm_{model} = \sqrt{\left(\frac{1}{T}\right)\sum_{j=1}^{T}\left(E_{RTS}^{model}\left[\phi_j\right] - E_{RTS}^{model\_avg}\right)^2} \quad (S209)$$

Substituting eqn (S205) into (S209) and making use of the orthonormality condition in eqn (S197) or (S198) yields

$$torsion\_norm_{model} = \sqrt{\frac{w}{2\pi}\sum_{m=1}^{m_{max}}\left(c_m{}^2\right)} \quad (S210)$$

Defining

$$SumCSq = \sum_{m=1}^{m_{max}}\left(c_m{}^2\right) \quad (S211)$$

eqn (S201), (S210), and (S211) combine to give

$$torsion\_norm_{model} = torsion\_norm_{QM}\sqrt{SumCSq} \quad (S212)$$

Inserting eqn (S202) into eqn (S201) and making use of eqn (S197) or (S198) gives

$$torsion\_norm_{QM} = \sqrt{\frac{w}{2\pi}\sum_{m=1}^{\infty}\left(\left(c_m^{DT}\right)^2\right)} = \sqrt{\frac{w}{2\pi}} \quad (S213)$$

which shows that the full set of mode coefficients for the normalized QM potential along the torsion scan curve satisfies

$$\sum_{m=1}^{\infty}\left(\left(c_m^{DT}\right)^2\right) = 1 \quad (S214)$$

Since $\{c_m\}$ are the coefficients for projection onto an orthonormal basis set, it follows that

$$0 \le SumCSq \le 1 \quad (S215)$$

SumCSq value can be interpreted as the fraction of the QM torsion scan curve that is recovered by the model potential. Iff $\mathrm{SumCSq} \approx 1$ and $E_{RTS}^{model\_avg} \approx E_{RTS}^{QM\_avg}$, then the model potential provides an adequate approximation of the QM potential along the torsion scan curve:

$$E_{RTS}^{model}[\phi] \approx E_{RTS}^{QM}[\phi] - E_{RTS}^{QM}\left[\phi_{eq}^{training}\right] \quad \text{(S216)}$$

Here, we consider the general case in which $E_{RTS}^{model\_avg}$ may potentially be different in value than $E_{RTS}^{QM\_avg}$. This leads to three scenarios. Scenario #1: This scenario chooses the value of $E_{RTS}^{model\_avg}$ such that

$$E_{RTS}^{model}\left[\phi_{eq}^{training}\right] = 0 \quad \text{(S217)}$$

which makes the left and right sides of eqn (S216) exactly equal to each other at the point $\phi = \phi_{eq}^{training}$. For the ADDT and CADT model potentials, this scenario corresponds to the choice

$$E_{RTS}^{model\_avg} = \frac{\sqrt{w}}{\sqrt{\pi}} \sum_{m=1}^{m_{max}} c_m^{DT} G_m^{DT,avg} \quad \text{(S218)}$$

For the ADCO and CACO model potentials, this scenario corresponds to the choice

$$E_{RTS}^{model\_avg} = -\frac{\sqrt{w}}{\sqrt{\pi}} \sum_{n=1}^{n_{max}} c_n^{CO} \cos\left[n\phi_{eq}^{training}\right] \quad \text{(S219)}$$

Scenario #2: This scenario chooses the value of $E_{RTS}^{model\_avg}$ such that

$$E_{RTS}^{model\_avg} = E_{RTS}^{QM\_avg} - E_{RTS}^{QM}\left[\phi_{eq}^{training}\right] \quad \text{(S220)}$$

which makes the averages of the left and right sides of eqn (S216) exactly equal to each other. Using a complete set of orthonormal DT projectors to expand the QM potential along the torsion scan curve yields the untruncated expansion:

$$E_{RTS}^{QM\_avg} - E_{RTS}^{QM}\left[\phi_{eq}^{training}\right] = \frac{\sqrt{w}}{\sqrt{\pi}} \sum_{m=1}^{\infty} c_m^{DT} G_m^{DT,avg} \quad \text{(S221)}$$

Scenario #3: This scenario (denoted as "other") encompasses any situations in which $E_{RTS}^{model\_avg}$ is chosen to satisfy any criteria different from Scenarios #1 and #2 described above.

Comparing eqn (S218), (S220), and (S221), the difference in $E_{RTS}^{model\_avg}$ for scenarios #1 and #2 using DT projectors is

$$E_{RTS}^{model\_avg}[\text{Scenario \#2}] - E_{RTS}^{model\_avg}[\text{Scenario \#1}] = \frac{\sqrt{w}}{\sqrt{\pi}} \sum_{m=(m_{max}+1)}^{\infty} c_m^{DT} G_m^{DT,avg} \quad \text{(S222)}$$

Examining eqn (S222), $E_{RTS}^{model\_avg}$ exactly coincides for the two scenarios if the modal coefficients c_m are zero for all values of $m > m_{max}$. If the modal coefficients c_m^{DT} are nearly zero for all values of $m > m_{max}$, then $E_{RTS}^{model\_avg}$ approximately coincides for the two scenarios.

The situation is slightly more complicated for the CO projectors than for the DT projectors. If sym_value (see eqn (S203)) equals zero, then in this case the QM potential can be expanded as

$$E_{RTS}^{QM\_avg} - E_{RTS}^{QM}\left[\phi_{eq}^{training}\right] = -\frac{\sqrt{w}}{\sqrt{\pi}} \sum_{n=1}^{\infty} c_n^{CO} \cos\left[n\phi_{eq}^{training}\right] \quad \text{(S223)}$$

Comparing eqn (S219), (S220), and (S223), the difference in $E_{RTS}^{model\_avg}$ for scenarios #1 and #2 using CO projectors when sym_value = 0 is

$$E_{RTS}^{model\_avg}[\text{Scenario \#2}] - E_{RTS}^{model\_avg}[\text{Scenario \#1}] = -\frac{\sqrt{w}}{\sqrt{\pi}} \sum_{n=(n_{max}+1)}^{\infty} c_n^{CO} \cos\left[n\phi_{eq}^{training}\right] \quad \text{(S224)}$$

When sym_value ≠ 0, eqn (S223) and (S224) do not apply. If sym_value is large, then the CO projectors might give a large difference in $E_{RTS}^{model\_avg}$ value between scenarios #1 and #2.

The definition for R-squared is

$$\text{R-squared} = R^2 = 1 - \frac{SSE}{SST} \tag{S225}$$

For a torsion scan curve, we define the sum of squares total (SST) as

$$SST = \sum_{j=1}^{T}\left(E_{RTS}^{QM}\left[\phi_j\right] - E_{RTS}^{QM\_avg}\right)^2 \tag{S226}$$

and the sum of squared errors (SSE) as

$$SSE = \sum_{j=1}^{T}\left(E_{RTS}^{QM}\left[\phi_j\right] - E_{RTS}^{QM}\left[\phi_{eq}^{training}\right] - E_{RTS}^{model}\left[\phi_j\right]\right)^2 \tag{S227}$$

This SSE can be rewritten as

$$SSE = \sum_{j=1}^{T}\left(\left(E_{RTS}^{QM}\left[\phi_j\right] - E_{RTS}^{QM\_avg}\right) + \left(E_{RTS}^{QM\_avg}\left[\phi_j\right] - E_{RTS}^{QM}\left[\phi_{eq}^{training}\right] - E_{RTS}^{model\_avg}\right) - \left(E_{RTS}^{model}\left[\phi_j\right] - E_{RTS}^{model\_avg}\right)\right)^2$$

(S228)

Expanding this square yields

$$\begin{aligned}
SSE = &\sum_{j=1}^{T}\left(E_{RTS}^{QM}\left[\phi_j\right] - E_{RTS}^{QM_avg}\right)^2 \\
&+\sum_{j=1}^{T}\left(E_{RTS}^{QM_avg} - E_{RTS}^{QM}\left[\phi_{eq}^{training}\right] - E_{RTS}^{model_avg}\right)^2 \\
&+\sum_{j=1}^{T}\left(E_{RTS}^{model}\left[\phi_j\right] - E_{RTS}^{model_avg}\right)^2 \\
&+2\sum_{j=1}^{T}\left(E_{RTS}^{QM}\left[\phi_j\right] - E_{RTS}^{QM_avg}\right)\left(E_{RTS}^{QM_avg} - E_{RTS}^{QM}\left[\phi_{eq}^{training}\right] - E_{RTS}^{model_avg}\right) \\
&-2\sum_{j=1}^{T}\left(E_{RTS}^{QM}\left[\phi_j\right] - E_{RTS}^{QM_avg}\right)\left(E_{RTS}^{model}\left[\phi_j\right] - E_{RTS}^{model_avg}\right) \\
&-2\sum_{j=1}^{T}\left(E_{RTS}^{QM_avg} - E_{RTS}^{QM}\left[\phi_{eq}^{training}\right] - E_{RTS}^{model_avg}\right)\left(E_{RTS}^{model}\left[\phi_j\right] - E_{RTS}^{model_avg}\right)
\end{aligned} \tag{S229}$$

The first term in this expansion is SST (see eqn (S226)). Other terms in this expansion can be rewritten as follows. Since $\left(E_{RTS}^{QM\_avg} - E_{RTS}^{QM}\left[\phi_{eq}^{training}\right] - E_{RTS}^{model\_avg}\right)$ is a constant, from eqn (S199) it follows that

$$\sum_{j=1}^{T}\left(E_{RTS}^{QM}\left[\phi_j\right] - E_{RTS}^{QM\_avg}\right)\left(E_{RTS}^{QM\_avg} - E_{RTS}^{QM}\left[\phi_{eq}^{training}\right] - E_{RTS}^{model\_avg}\right) = 0 \tag{S230}$$

and from eqn (S207) it follows that

$$\sum_{j=1}^{T}\left(E_{RTS}^{QM\_avg} - E_{RTS}^{QM}\left[\phi_{eq}^{training}\right] - E_{RTS}^{model\_avg}\right)\left(E_{RTS}^{model}\left[\phi_j\right] - E_{RTS}^{model\_avg}\right) = 0 \tag{S231}$$

Combining eqn (S209), (S212), (S201), and (S226) gives

$$\sum_{j=1}^{T}\left(E_{RTS}^{model}\left[\phi_j\right] - E_{RTS}^{model\_avg}\right)^2 = (SST)(SumCSq) \tag{S232}$$

Using eqn (S205) gives

$$\sum_{j=1}^{T}\left(E_{RTS}^{QM}\left[\phi_j\right] - E_{RTS}^{QM\_avg}\right)\left(E_{RTS}^{model}\left[\phi_j\right] - E_{RTS}^{model\_avg}\right) = \sum_{j=1}^{T}\left(E_{RTS}^{QM}\left[\phi_j\right] - E_{RTS}^{QM\_avg}\right)\left(\sqrt{w}\sum_{m=1}^{m_{max}} c_m \frac{P_m[\phi]}{\sqrt{\pi}}\right) \tag{S233}$$

Using eqn (S208), eqn (S233) is rewritten as

$$\sum_{j=1}^{T}\left(E_{RTS}^{QM}\left[\phi_j\right]-E_{RTS}^{QM\_avg}\right)\left(E_{RTS}^{model}\left[\phi_j\right]-E_{RTS}^{model\_avg}\right)=\frac{Tw}{2\pi}\sum_{m=1}^{m_{max}}\left(c_m{}^2\right) \quad (S234)$$

From eqn (S201) and (S226), it follows that

$$SST=\frac{Tw}{2\pi} \quad (S235)$$

Substituting eqn (S211) and (S235) into (S234) gives

$$\sum_{j=1}^{T}\left(E_{RTS}^{QM}\left[\phi_j\right]-E_{RTS}^{QM\_avg}\right)\left(E_{RTS}^{model}\left[\phi_j\right]-E_{RTS}^{model\_avg}\right)=\left(SST\right)\left(SumCSq\right) \quad (S236)$$

Now, substituting eqn (S226), (S230)–(S232), and (S236) into (S229) gives

$$SSE=SST\left(1-SumCSq\right)+\sum_{j=1}^{T}\left(E_{RTS}^{QM\_avg}-E_{RTS}^{QM}\left[\phi_{eq}^{training}\right]-E_{RTS}^{model\_avg}\right)^2 \quad (S237)$$

For a torsion scan curve, eqn (S237) is exact.

Substituting eqn (S237) into (S225) gives

$$\text{R-squared}=R^2=SumCSq-\frac{T}{SST}\left(E_{RTS}^{QM\_avg}-E_{RTS}^{QM}\left[\phi_{eq}^{training}\right]-E_{RTS}^{model\_avg}\right)^2 \quad (S238)$$

Examining eqn (S238),

$$\text{R-squared}\le SumCSq \quad (S239)$$

For Scenario #2, combining eqn (S220) and (S238) shows that

$$\text{R-squared}=R^2=SumCSq \quad (S240)$$

Eqn (S240) holds in Scenario #2 irrespective of whether DT or CO projectors are used and irrespective of the value of sym_value, but of course the value of SumCSq (and hence of R-squared) is impacted by which projectors are used.

For Scenario #1 using DT projectors, inserting eqn (S220) and (S222) into (S238) gives

$$\text{R-squared}=R^2=SumCSq-\frac{T}{SST}\left(\frac{\sqrt{w}}{\sqrt{\pi}}\sum_{m=\left(m_{max}+1\right)}^{\infty}c_m^{DT}G_m^{DT,avg}\right)^2 \quad (S241)$$

Inserting eqn (S235) into (S241) gives

$$\text{R-squared}=R^2=SumCSq-2\left(\sum_{m=\left(m_{max}+1\right)}^{\infty}c_m^{DT}G_m^{DT,avg}\right)^2 \quad (S242)$$

Thus when using Scenario #1, R-squared is close to SumCSq when c_m^{DT} for every omitted torsion mode is close to zero. Iff sym_value = 0, then an analogous derivation using the CO projectors yields

$$\text{R-squared}=R^2=SumCSq-2\left(\sum_{n=\left(n_{max}+1\right)}^{\infty}c_n^{CO}\cos\left[n\phi_{eq}^{training}\right]\right)^2 \quad (S243)$$

When computing the R-squared value as described above, all T geometries along the torsion scan curve had equal observation weights. In classical molecular dynamics or Monte Carlo simulations employing the NPT, NVT, μPT, or μVT thermodynamic ensembles, the lower-energy geometries should appear more often (i.e., have higher observation weights) than higher-energy geometries. When every geometry along the torsion scan curve has equal observation weights, scenario #2 described above has a higher R-squared value (see eqn (S240)) than scenario #1 (see eqn (S242)). Scenario #1 (see eqn (S217)) exactly matches the relative energy of the QM and

model potentials at the training dataset's optimized ground-state geometry. For this reason, scenario #1 typically performs better than scenario #2 when employing the NPT, NVT, μPT, or μVT thermodynamic ensembles, because scenario #1 gives smaller errors than scenario #2 for the lower-energy geometries that receive relatively higher observation weights in such ensembles. For this reason, my ADDT, CADT, ADCO, and CACO model potentials are typically constructed according to scenario #1 rather than according to scenario #2. Except where otherwise indicated, the ADDT, CADT, ADCO, and CACO model potentials were constructed according to scenario #1 throughout this work.

References

S1. W.C. Swope and D.M. Ferguson, "Alternative expressions for energies and forces due to angle bending and torsional energy," *J. Comput. Chem.*, 1992, **13**, 585-594, doi: 10.1002/jcc.540130508.

S2. R. Ghanavati, C. Escobosa, and T.A. Manz, "An automated protocol to construct flexibility parameters for classical forcefields: applications to metal-organic frameworks," *RSC Adv.*, 2024, **14**, 22714-22762, doi: 10.1039/d4ra01859a.

S3. T.A. Manz, "A formally exact theory to construct nonreactive forcefields using linear regression to optimize bonded parameters," *RSC Adv.*, 2024, **14**, 33345-33383, doi: 10.1039/d4ra01861c.

S4. P.L. Butzer, M.M. Dodson, P.J.S.G. Ferreira, J.R. Higgins, O. Lange and P. Seidler, "Herbert Raabe's work in multiplex signal transmission and his development of sampling methods," *Signal Process.*, 2010, **90**, 1436–1455, doi: 10.1016/j.sigpro.2009.11.018.

S5. C.E. Shannon, "Communication in the presence of noise," *Proc. IEEE*, 1998, **86**, 447–457, doi: 10.1109/JPROC.1998.659497.

S6. H.D. Luke, "The origins of the sampling theorem," *IEEE Commun. Mag.*, 1999, **37**, 106–108, doi: 10.1109/35.755459.

S7. H. Nyquist, "Certain topics in telegraph transmission theory," *Proc. IEEE*, 2002, **90**, 280–305, doi: 10.1109/5.989875.

S8. N. Gabaldon Limas and T. A. Manz, "Introducing DDEC6 atomic population analysis: part 4. Efficient parallel computation of net atomic charges, atomic spin moments, bond orders, and more," *RSC Adv.*, 2018, **8**, 2678-2707, doi: 10.1039/c7ra11829e.
